# Comparative genomics and multiomics analyses reveal the evolution and physiological basis of rubber biosynthesis in *Hevea* species

**DOI:** 10.1093/gigascience/giaf115

**Published:** 2025-10-10

**Authors:** Nyok-Sean Lau, Emiko Okubo-Kurihara, Yuko Makita, Fetrina Oktavia, Tomoko Kuriyama, Yukio Kurihara, Hidefumi Hamasaki, Yuki Nakamura, Mitsutaka Kadota, Osamu Nishimura, Shigehiro Kuraku, Ahmad Sofiman Othman, Minami Matsui

**Affiliations:** RIKEN Center for Sustainable Resource Science, Yokohama 230-0045, Japan; Centre for Chemical Biology, Universiti Sains Malaysia, Bayan Lepas 11900 Penang, Malaysia; RIKEN Center for Sustainable Resource Science, Yokohama 230-0045, Japan; Department of Biology, Keio University, Yokohama 223-8522, Japan; RIKEN Center for Sustainable Resource Science, Yokohama 230-0045, Japan; Mebashi Institute of Technology, Maebashi, Gunma 371-0816, Japan; Indonesian Rubber Research Institute, Sembawa, Banyuasin 30953, Indonesia; RIKEN Center for Sustainable Resource Science, Yokohama 230-0045, Japan; RIKEN Center for Sustainable Resource Science, Yokohama 230-0045, Japan; RIKEN Center for Sustainable Resource Science, Yokohama 230-0045, Japan; Plant Lipid Research Team, RIKEN Center for Sustainable Resource Science, Yokohama 230-0045, Japan; Laboratory for Phyloinformatics, RIKEN Center for Biosystems Dynamics Research (BDR), Kobe 650-0047, Japan; Laboratory for Phyloinformatics, RIKEN Center for Biosystems Dynamics Research (BDR), Kobe 650-0047, Japan; Laboratory for Phyloinformatics, RIKEN Center for Biosystems Dynamics Research (BDR), Kobe 650-0047, Japan; Centre for Chemical Biology, Universiti Sains Malaysia, Bayan Lepas 11900 Penang, Malaysia; School of Biological Sciences, Universiti Sains Malaysia, Minden 11800 Penang, Malaysia; RIKEN Center for Sustainable Resource Science, Yokohama 230-0045, Japan; Yokohama City University, Kihara Institute for Biological Research, Totsuka, Yokohama 244-0813, Kanagawa, Japan

**Keywords:** *Hevea* species, comparative genomics, proteomics, lipidomics, rubber biosynthesis, latex metabolism

## Abstract

**Background:**

There are multiple species within the *Hevea* genus, each exhibiting distinct characteristics, but many remain underexplored due to their lower latex productivity. While *Hevea brasiliensis* is the primary source of natural rubber, other *Hevea* species represent valuable gene pools that could be leveraged in breeding programs to enhance latex yield, biosynthesis efficiency, and the physicochemical properties of latex. With increasing interest in enhancing natural rubber traits, these lesser-known species are being revisited for their underexplored genetic diversity.

**Results:**

In this study, we performed a pangene analysis of 6 *Hevea* species and varieties, integrating proteomic and lipidomic data to investigate genetic and metabolic variation related to rubber biosynthesis and latex composition. The pangene analysis revealed conserved and expanded ATP-related functions, underscoring ATP’s role in latex production. The proteomic data identified key enzymes involved in rubber biosynthesis and differentially abundant proteins related to latex regeneration, suggesting that regeneration capacity may influence yield efficiency. Lipidomic profiling uncovered species-specific lipid compositions associated with membrane dynamics and rubber particle stability, which may contribute to latex properties.

**Conclusions:**

These findings provide valuable insights into *Hevea*’s genomic and metabolic diversity, supporting future breeding programs aimed at improving natural rubber production and its performance in various applications.

## Introduction

Natural rubber stands as an indispensable natural polymer, integral to the manufacture of diverse industrial products, owing to its unparalleled physical properties. Despite extensive research, no synthetic polymer has yet been developed that can replicate the physical attributes of natural rubber. Although more than 2,500 plant species are known to biosynthesize natural rubber [1, 2], commercial natural rubber production depends exclusively on latex derived from the Pará rubber tree, *Hevea brasiliensis* Muell. Arg. The genus *Hevea*, belonging to the family Euphorbiaceae, comprises 11 species, including *H. brasiliensis, Hevea bethamiana, Hevea camporum, Hevea camargoana, Hevea guianensis, Hevea microphylla, Hevea nitida, Hevea pauciflora, Hevea paludosa, Hevea rigidifolia*, and *Hevea spruceana* [3, 4]. Although the genus is diverse, *H. brasiliensis* is the primary source of natural rubber, accounting for over 90% of global production, estimated at 14 million tons annually according to the website of the Food and Agriculture Organization of the United Nations. Originally from the Amazon basin, *H. brasiliensis* has seen its cultivation shift mainly to Southeast Asia since Henry Wickham introduced rubber tree seeds to the region in 1876 [5]. As a result, global rubber production is now largely dependent on a limited genetic pool from a small collection of seeds. Over the past decades, global rubber production has increased due to the expansion of cultivated areas and a significant increase in productivity, with genetic improvement playing a crucial role.

Natural rubber, a *cis*-1,4-polyisoprene polymer, is synthesised and stored in specialized phloem cells known as laticifers, specifically on the surfaces of rubber particles [6]. The elongation of rubber chains is catalyzed by rubber transferase, a member of the *cis*-prenyltransferase (CPT) family, through the sequential addition of isopentenyl diphosphate (IPP) to prenyl groups [7]. A CPT-like (CPTL) protein is thought to be associated with the rubber transferase complex, where it interacts with and activates CPT [8, 9]. In plants, IPP is synthesized via the cytosolic mevalonate (MVA) and plastidic methylerythritol (MEP) pathways, with evidence suggesting the MVA pathway as the primary source for natural rubber production in *H. brasiliensis* [10]. The synthesis of natural rubber is also supported by rubber elongation factors (REFs) and small rubber particle proteins (SRPPs), the 2 most abundant proteins on rubber particles, which are essential for rubber particle stability and rubber biosynthesis [11, 12]. Latex coagulation, a crucial process for rubber production, is mediated by lutoid-derived proteins such as hevamine/chitinase, β-1,3-glucanase, and hevein, which are released upon lutoid disruption during tapping and promote rubber particle aggregation [13]. Latex biosynthesis is regulated by hormonal signaling pathways, particularly ethylene and jasmonates, which can be produced in response to wounding or applied exogenously. Jasmonates are known to play a role in secondary laticifer differentiation, while ethylene has been shown to prolong the duration of latex flow [14, 15]. Understanding the genetic basis of the rubber biosynthetic pathway is integral to optimizing rubber yield and improving production efforts.

Although multiple genome assemblies have been published for *H. brasiliensis*, genomic analyses of other *Hevea* species have only recently become available [16–23]. Genetic information beyond *H. brasiliensis* remains limited, restricting the gene space accessible for comparative studies and breeding. To help address this gap and improve our understanding of diversity within the genus, we performed a pangene analysis of 5 *Hevea* species or varieties alongside the *H. brasiliensis* reference genome. Our comparative genomic analyses, integrated with proteomics and lipidomics data, enabled the exploration of key agronomic traits, including core components of rubber biosynthesis, factors influencing its capacity, and lipid composition relevant to latex properties. This multiomics approach offers a broader view of molecular variation related to rubber biosynthesis and latex traits in *Hevea*, providing a useful resource to inform future efforts in genetic improvement and species conservation.

## Results

### *Hevea* species genome sequences as a resource for diversity studies

**Figure 1:**
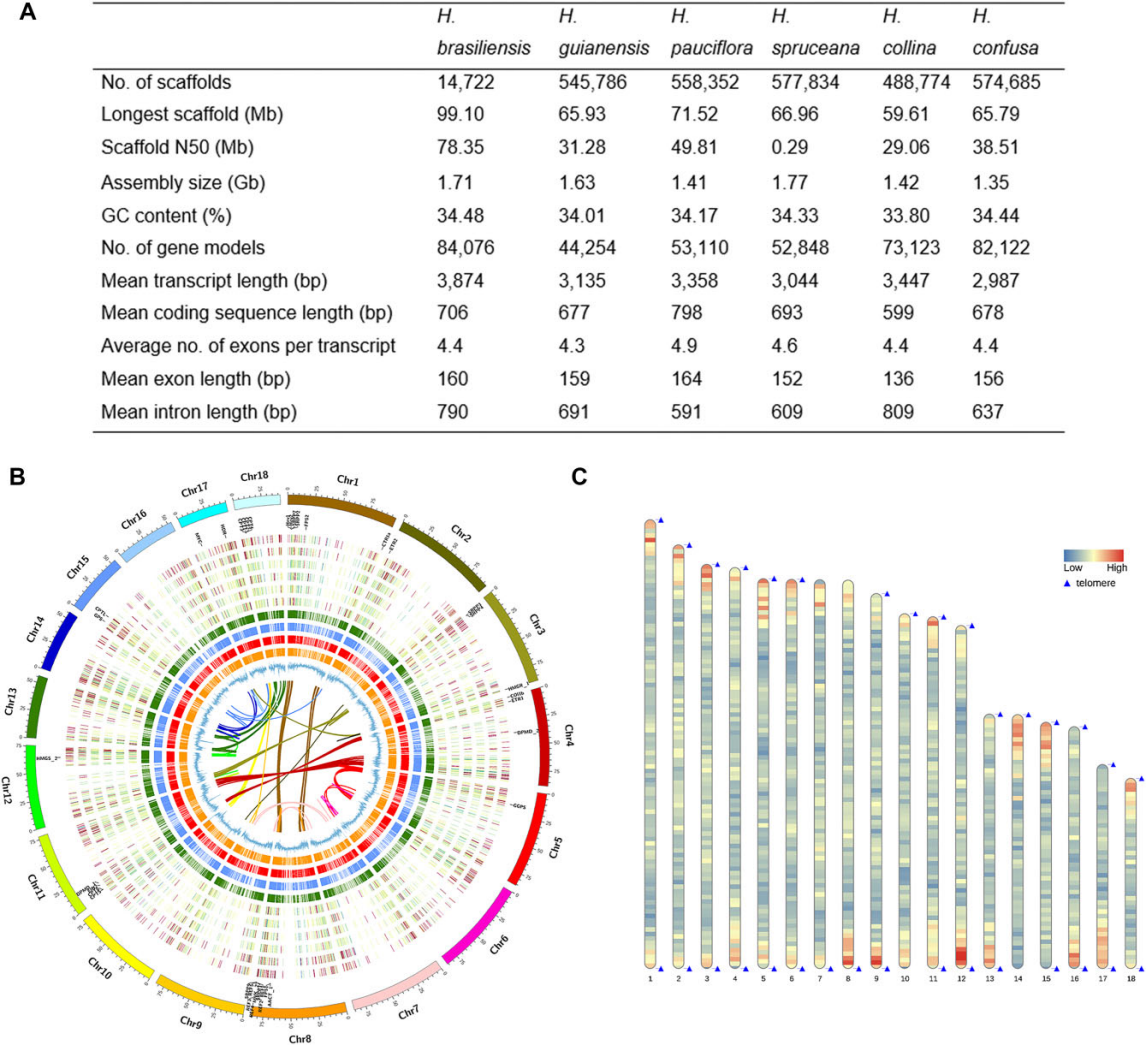
Genome assembly and annotation of *Hevea* species. (A) Summary of genome assembly and annotation statistics across *Hevea* species. (B) Circos plot showing the chromosomal organization of *Hevea brasiliensis* (outermost ring) and comparative features across *Hevea* species. From outside to inside: proteome abundance in *H. confusa, H. collina, H. spruceana, H. guianensis*, and *H. brasiliensis*, with blue indicating high abundance and red indicating low abundance; rubber biosynthesis-related genes are labeled; orthologous gene relationships between *H. brasiliensis* and *A. thaliana, M. esculenta, R. communis*, and *J. curcas*; GC content across chromosomes; and the innermost links represent gene duplications within *H. brasiliensis*. (C) Chromosomal distribution of telomeric regions in *H. brasiliensis*, with telomeric regions marked by blue triangles and gene density represented by a heatmap, where red indicates gene-rich regions and blue indicates gene-poor regions.

We previously reported a draft genome sequence of *H. brasiliensis* RRIM 600, a historically important clone widely planted during the early expansion of rubber cultivation in East Asia [22] (Supplementary Text S1). To support comparative analyses, RRIM600 was selected for high-quality genome reconstruction. A chromosome-level genome was constructed using a hybrid approach integrating PacBio SMRT and Hi-C sequencing (Supplementary Table S1). The initial PacBio assembly, based on 169.94 Gb of reads, yielded a 1.71-Gb genome with an N50 of 550.35 Kb (Supplementary Table S2). Refinement with Hi-C reads (105-fold coverage) produced a final assembly of 1.71 Gb, with a scaffold N50 of 78.35 Mb (Fig. 1A, Supplementary Text S2, and Supplementary  Table S3). More than 78% of the assembly was anchored onto 18 pseudo-chromosomes, covering 91% of the estimated 1.88-Gb genome size (Fig. 1B, Supplementary Figs. S1–S2, and Supplementary Table S4). BUSCO assessment showed 98.7% completeness, and over 90% of PacBio Iso-seq transcripts aligned to the assembly, indicating high contiguity and sequence quality (Supplementary Text S3, Supplementary Fig. S3, and Supplementary Tables S5–S6). Centromeric regions were identified in all 18 chromosomes, while telomeric sequences were detected at both ends of most chromosomes, with only 1 telomeric end identified in 4 chromosomes (Fig. 1C, Supplementary Tables S7–S8). To explore genomic diversity in *Hevea*, we sequenced 5 additional *Hevea: H. guianensis, H. pauciflora, H. spruceana, H. confusa* (*H. pauciflora* var. *confusa*), and *H. collina* (*H. guianensis* var. *collina*) (Supplementary Text S4). Their genomes were *de novo* assembled using 56.9× to 95.6× Illumina reads and synteny scaffolded with *H. brasiliensis* as reference. Assembly sizes ranged from 1.35 to 1.77 Gb, with N50 values of 0.29 to 38.51 Mb and GC content between 33.80% and 34.48% (Supplementary Fig. S4).

Annotation using the MAKER pipeline predicted an average of ∼65,000 high-confidence gene models, with *H. brasiliensis* containing the most (84,076) and *H. guianensis* the fewest (44,254). Predicted protein-coding genes were mapped to functional databases, including NCBI NR, SwissProt, TrEMBL, InterPro, Gene Ontology (GO), and Kyoto Encyclopedia of Genes and Genomes (KEGG) (Supplementary Table S9). The gene models averaged 3,044 to 3,874 bp in transcript length, 599 to 798 bp in coding sequence length, and 4 exons per gene (Supplementary Fig. S5). Noncoding RNA genes, including transfer RNAs (tRNAs), ribosomal RNAs (rRNAs), microRNAs (miRNAs), and small nucleolar RNAs (snoRNAs), were also identified (Supplementary Table S10). Repeat annotation using *de novo* and homology-based approaches revealed variability across *Hevea* genomes. *H. brasiliensis* exhibited the highest repeat content (74.1%), while *H. collina* had the lowest (33.3%) (Supplementary Table S11). Long terminal repeats (LTRs) dominated, constituting 31.21–70.54% of the genomes, with Gypsy elements being particularly abundant (31.45–54.92%). In contrast, long interspersed nuclear elements (LINEs) accounted for only 1.04–1.60%, and DNA transposons ranged from 0.79–1.94%. The Gypsy-to-Copia ratio was consistently high across species. Divergence analysis of transposable elements based on Kimura distances revealed a peak divergence rate of around 6 for *H. pauciflora* and 9–10 for other species, with the Gypsy superfamily being the major component of the peak (Supplementary Fig. S6).

### Comparative genomics of *Hevea* species

**Figure 2:**
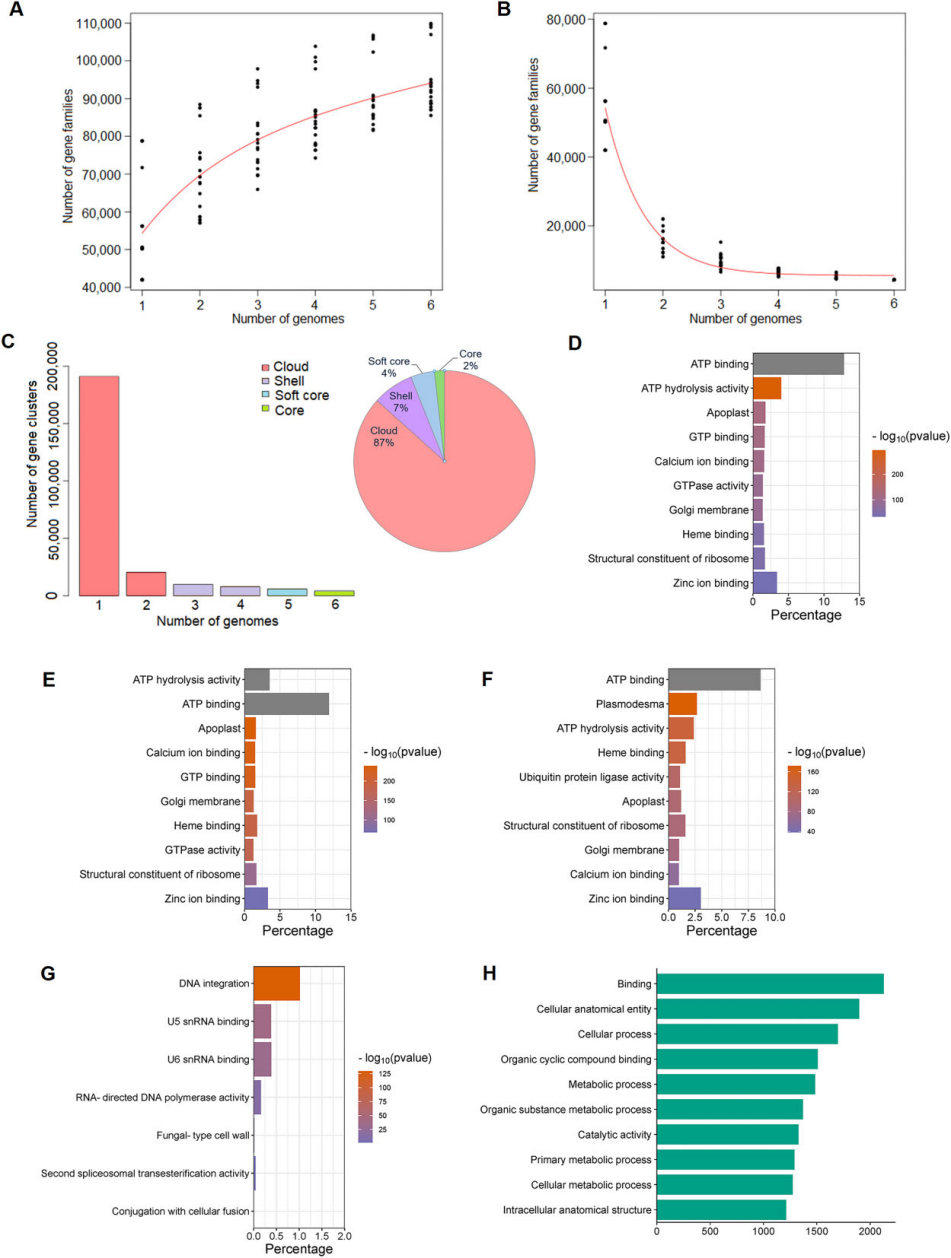
Pangene analysis of *Hevea*. Simulations of the (A) increase in the pangene size and (B) decrease in the core gene size. (C) Composition of the *Hevea* pangene. Gene Ontology (GO) enrichment of the (D) core, (E) soft core, (F) shell, and (G) cloud genes relative to the pangene. (H) GO annotation of the *H. brasiliensis*–specific genes.

To study the genomic landscape of the *Hevea* genus, we conducted comparative analyses on the assembled genomes of *H. brasiliensis, H. guianensis, H. pauciflora, H. spruceana, H. collina*, and *H. confusa* (Fig. 2A–C). Orthologous gene analysis classified all genes from these 6 genomes into 239,660 families. Our findings revealed that 2% of the gene families were present in the core cluster, 4% in the soft core, 7% in the shell, and 87% in the cloud clusters. The core and soft-core clusters harbored highly conserved genes shared across all analyzed genomes, while the shell and cloud clusters contained flexible genes that may serve as reservoirs of genetic variability contributing to adaptive potential. GO enrichment analysis of the core and soft-core gene clusters identified significantly enriched GO terms, with the largest gene members included in adenosine triphosphate (ATP) binding, ATP hydrolysis activity, and apoplast (Fig. 2D, E, Supplementary Tables S12–S13). In the shell gene cluster, enriched pathways also encompassed ATP binding and ATP hydrolysis activity, but also plasmodesma (Fig. 2F, Supplementary Table S14). Functional annotation of the cloud genes revealed significant enrichment of GO terms associated with DNA integration, U5 small nuclear RNA (snRNA) binding, and U6 snRNA binding (Fig. 2G, Supplementary Table S15).

Modeling of pangene size showed that the total number of gene families increased with the addition of each new genome, indicating that *Hevea* genomes exhibit genetic variation, and the analyzed genomes may not fully capture the diversity of the genus. Furthermore, species-specific genes in *H. brasiliensis* were annotated, revealing an abundance of pathways related to binding, cellular anatomical structures, and cellular processes (Fig. 2H). These fundamental function genes have likely evolved independently to support the distinct biological characteristics of *H. brasiliensis*.

**Figure 3:**
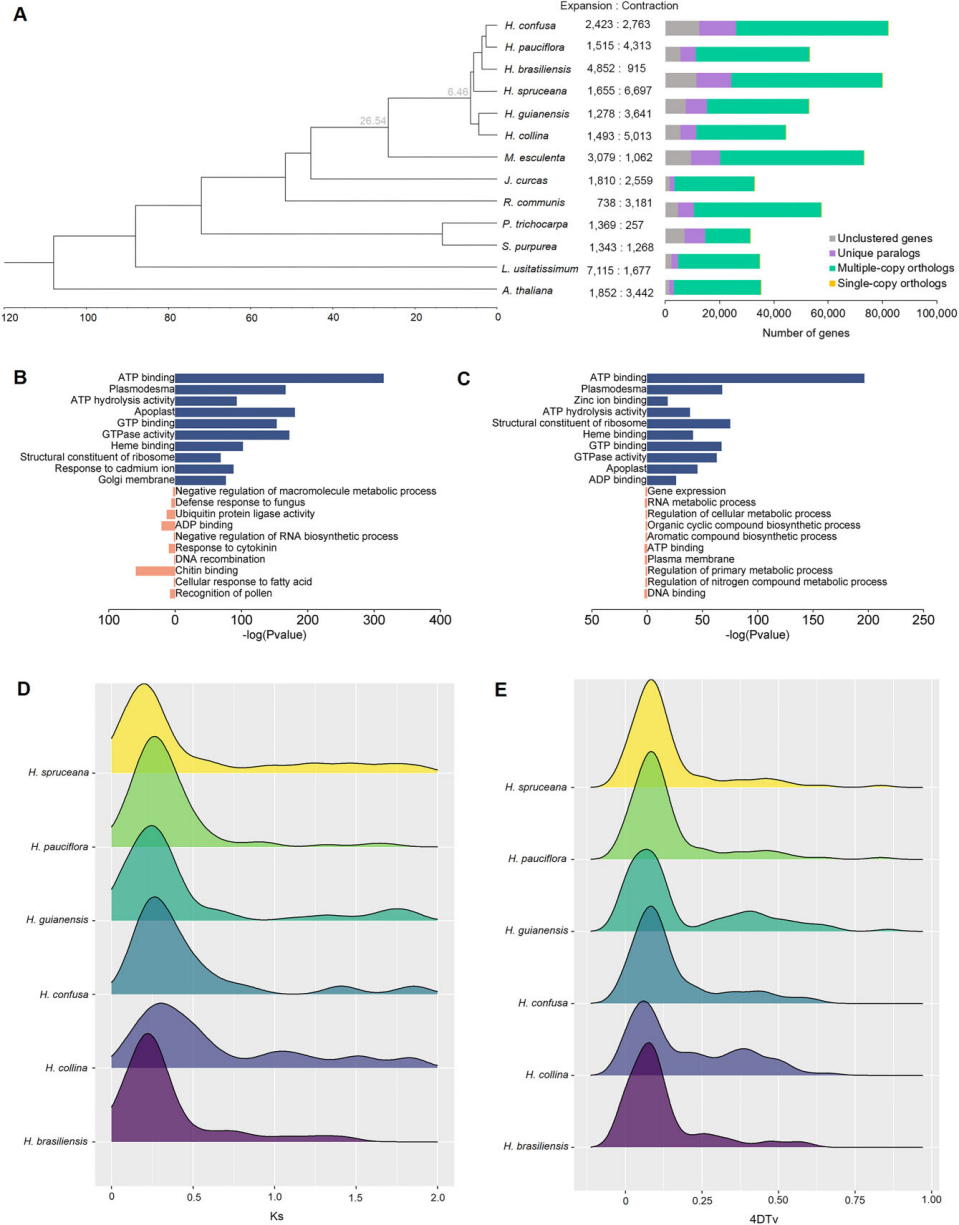
Comparative genomics of *Hevea* species. (A) Maximum likelihood phylogeny inferred from single-copy orthologs. The column on the right represents the number of expanded and contracted gene families. Bar chart shows the distribution of single-copy, multiple-copy, and unique orthologs in the 11 plant species. Gene Ontology enrichment of genes expanded (blue) and contracted (pink) in (B) *Hevea* species and (C) *H. brasiliensis*. The distribution of (D) *K*_s_ and (E) 4DTv values of the syntenic gene pairs in the *H. brasiliensis, H. guianensis, H. pauciflora, H. collina*, and *H. confusa* genomes.

To trace the evolutionary history of *Hevea*, gene family clustering was performed using gene sets from 13 plant species, including 6 *Hevea*, 6 representative Malpighiales species, and the outgroup *Arabidopsis thaliana*. This analysis identified 1,156 single-copy orthologs shared between *Hevea* and the other plants, which were used for phylogenetic reconstruction and divergence time estimation (Fig. 3A). Our results indicate that all *Hevea* species share a common ancestor around 6.46 million years ago (Mya), with *Hevea* diverging from *Manihot esculenta* approximately 26.54 Mya. *H. brasiliensis* exhibited high conservation of orthologous genes with *M. esculenta, Ricinus communis*, and *Jatropha curcas*, particularly with *M. esculenta* (Fig. 1B). The phylogenomic analysis revealed 2 distinct clades within *Hevea*: one contains *H. brasiliensis, H. spruceana, H. pauciflora*, and its variety *H. confusa*; the other comprises *H. guianensis* and its variety *H. collina*.

To investigate changes in gene family size during evolution, we analyzed expansions and contractions of gene families in *Hevea* relative to the most recent common ancestor of the 13 plant species, as well as in *H. brasiliensis* relative to the common ancestor of *Hevea* (Fig. 3A). Expanded gene families in *Hevea* species were enriched for functions related to ATP binding, plasmodesma, and ATP hydrolysis activity (Fig. 3B, Supplementary Table S16). In *H. brasiliensis*, expanded gene families also showed enrichment in ATP binding, plasmodesma, and zinc ion binding (Fig. 3C, Supplementary Table S17). Conversely, contracted gene families in *Hevea* species exhibited an overrepresentation of functions associated with the negative regulation of macromolecule metabolic process, defense response to fungus, and ubiquitin protein ligase activity (Supplementary Table S18). In *H. brasiliensis*, contracted gene families were overrepresented with gene expression, RNA metabolic process, and the regulation of cellular metabolic process (Supplementary Table S19).

For dating the whole-genome duplication event in *Hevea*, a genomic analysis based on self-comparison of *H. brasiliensis, H. guianensis, H. pauciflora, H. spruceana, H. collina*, and *H. confusa* paralogous genes was carried out. All *Hevea* genomes exhibited a major left peak in the distributions of synonymous substitutions per synonymous site (*K*_s_) and transversions at 4-fold degenerate site (4DTv) frequency, indicating a recent species-specific duplication (Fig. 3D, E). *K*_s_ estimates for paralogs in *H. brasiliensis, H. guianensis, H. pauciflora, H. spruceana, H. collina*, and *H. confusa* suggested that this *Hevea*-specific duplication event may date back to ∼12.3–28.7 Mya. Additionally, a minor right peak in the 4DTv distribution and a right smooth peak in the *K*_s_ distribution correspond to the whole-genome triplication event across core eudicots.

### Evolution of rubber biosynthesis potential in *Hevea* species

**Figure 4:**
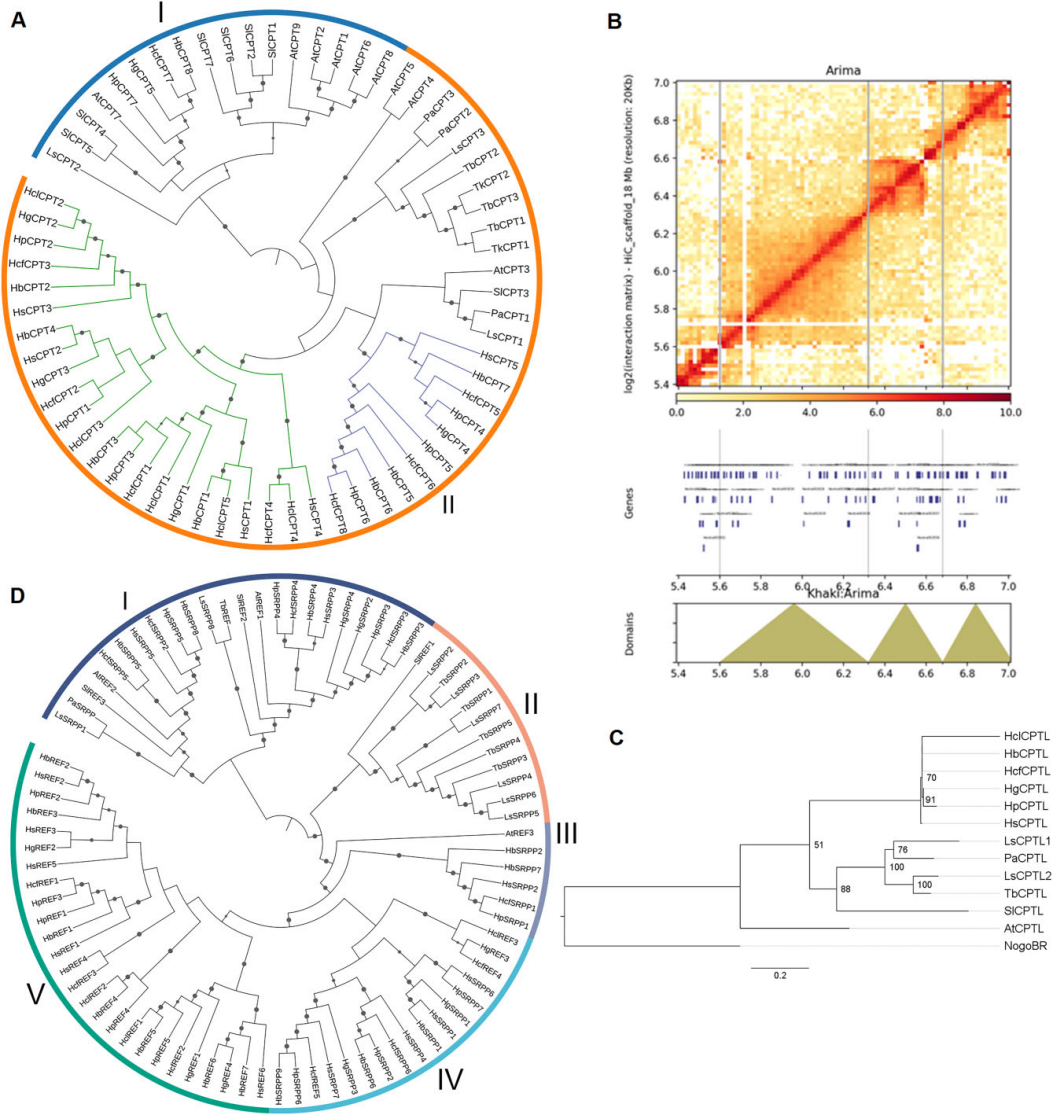
Analysis of rubber biosynthesis-related genes. (A) Maximum likelihood tree of CPT amino acid sequences from *Hevea* and other plants. (B) Hi-C plot showing TAD-likes structure for CPT1–4 on chromosome 18. (C) Maximum likelihood tree of CPTL nucleotide sequences from *Hevea* and other plants. (D) Maximum likelihood tree of REF and SRPP amino acid sequences from *Hevea* and other plants. At, *A. thaliana*; Hb, *H. brasiliensis*; Hcf, *H. confusa*; Hcl, *H. collina*; Hg, *H. guianensis*; Hp, *H. pauciflora*; Hs, *H. spruceana*; Ls, *L. sativa*; Pa, *P. argentatum*; Sl, *S. lycopersicum*; and Tb, *T. brevicorniculatum*.

**Figure 5:**
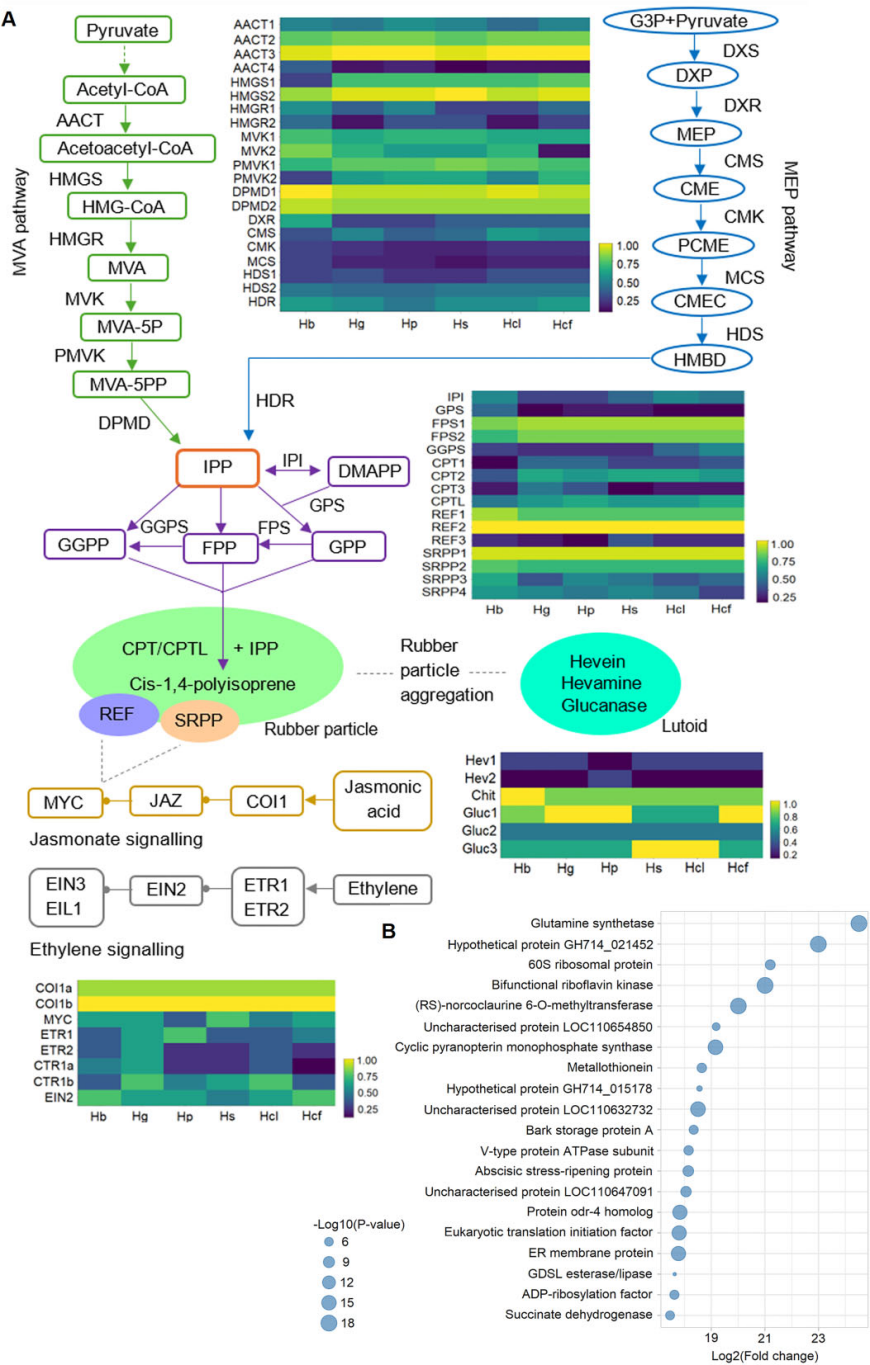
Rubber biosynthesis pathway and proteomic profiles in *Hevea* species. Heatmap showing log_2_-transformed average protein abundance across 3 biological replicates per sample, with color coding at which yellow indicates high abundance and blue signifies low abundance. MVA pathway: AACT, HMGS, HMGR, MVK, MVA-5-p, MVA-5-pp, PMK, DPMD. MEP pathway: G3P, DXS, DXP, DXS, DXR, MEP, CMS, CME, CMK, PCME, MCS, CMEC, HDS, HMBD, HDR. Formation of isoprenoid precursors: IPP, IPI, DMAPP, GPS, GGPP, FPS, GGPS, GPP. Rubber particle-associated proteins: CPT, REF, SRPP, CPTL. Jasmonic acid signaling: COI1, JAZ, MYC. Ethylene signaling: ETR, CTR1, EIN2/3, EIL1. (B) Differentially abundant proteins between *H. brasiliensis* and other *Hevea* species samples. Bubble plot shows proteins with significantly different abundance levels, where bubble size is proportional to statistical significance value.

The biosynthesis of natural rubber in *Hevea* involves a network of metabolic pathways and key proteins. Genes linked to rubber biosynthesis were identified through genome annotation across *Hevea* species, and their activity in latex was confirmed by proteomics (Figs. 4–5, Supplementary Text S5). The relative abundances of proteins in latex were quantified using data-independent acquisition coupled with high-resolution mass spectrometry (Supplementary Text S6 and Supplementary Table S20).

### IPP monomer supplying pathways

At the core of this process are the pathways that supply IPP, the fundamental building block for natural rubber. IPP is produced via 2 compartmentalized metabolic routes: the MVA pathway in the cytosol and the MEP pathway in the plastids. In *Hevea*, 14–18 genes are annotated in the MVA pathway, while 14–22 are associated with the MEP pathway (Supplementary Table S21). Proteomic analysis identified 14 of the 18 MVA pathway proteins in latex but only 7 from the MEP pathway (Fig. 5A, Supplementary Table S22). Notably, MEP pathway proteins were detected at much lower levels, and 1-deoxy-D-xylulose 5-phosphate synthase (DXS), which catalyzes the first MEP pathway step, was absent from the latex proteome. In the MVA pathway, all 6 key enzymes, acetyl-CoA acetyltransferase (AACT), 3-hydroxy-3-methylglutaryl-coenzyme A synthase (HMGS), HMG-CoA reductase (HMGR), mevalonate kinase (MVK), phosphomevalonate kinase (PMVK), and mevalonate diphosphate decarboxylase (DPMD), were detected in latex, represented by 2 to 4 isoforms. AACT3, HMGS1, and DPMD were among the most abundant isoforms.

During rubber elongation, IPP and its isomer, dimethylallyl diphosphate, undergo sequential condensations to form geranyl pyrophosphate (GPP), farnesyl pyrophosphate (FPP), and geranylgeranyl pyrophosphate (GGPP), catalyzed by *trans*-prenyltransferases such as geranyl pyrophosphate synthase (GPS), farnesyl pyrophosphate synthase (FPS), and geranylgeranyl pyrophosphate synthase (GGPS). These short-chain prenyl pyrophosphates serve as initiators for further IPP condensation, leading to the production of high molecular weight natural rubber. Among these enzymes, FPS was the most abundant in latex, indicating its role as the major *trans*-prenyltransferase in *Hevea*, as its product, FPP, has been identified as the main initiator of natural rubber biosynthesis [24].

### Rubber particle−associated proteins

The key enzymes responsible for natural rubber biosynthesis, CPTs, were annotated in *Hevea* genomes: CPT1–8 in *H. brasiliensis*, CPT1–5 in *H. guianensis*, CPT1–7 in *H. pauciflora*, CPT1–5 in *H. spruceana*, CPT1–5 in *H. collina*, and CPT1–8 in *H. confusa* (Fig. 4A, Supplementary Table S23). Chromosome 18 emerged as a hotspot for CPT genes, with several clustered together, suggesting coordinated regulation, supported by the observation of topologically associating domain structures for CPT1–4 in the *H. brasiliensis* leaf Hi-C analysis (Fig. 4B. Supplementary Fig. S7). Comparative analyses of CPT orthologs across multiple species, including *Linum usitatissimum, P. trichocarpa*, and *Salix purpurea* from Malpighiales, and *J. curcas, M. esculenta, R. communis*, and *Hevea* species from Euphorbiaceae, revealed a higher CPT gene count in Euphorbiaceae (average of 7) compared to non-Euphorbiaceae Malpighiales (average of 4), indicating a lineage-specific duplication event in Euphorbiaceae (Supplementary Table S24).

Phylogenetic analysis, including rubber-producing species such as *Taraxacum koksaghyz, Taraxacum brevicorniculatum*, and *Parthenium argentatum*, revealed 2 CPT clades: clade I, comprising chloroplastic CPTs, and clade II, consisting of cytosolic CPTs, consistent with previous studies (Fig. 5A) [8]. Within clade II, specific *Hevea* CPTs formed subclades distinct from other rubber-producing plants. Proteomic analysis of *Hevea* latex showed that CPT1–3 from clade II were exclusively expressed in latex, reinforcing their role in rubber biosynthesis. Multiple alignment of CPT sequences identified conserved regions I–V characteristic of known CPT structure in *Hevea* [25] (Supplementary Fig. S8).

The rubber transferase complex, comprising CPTs and CPT-like (CPTL) proteins, has been characterized in *Hevea* and other rubber-producing species, where CPTLs anchor, activate, and stabilize CPTs on rubber particles without directly catalyzing polymerization [8, 9, 26, 27]. Genome annotation and proteomic detection confirm CPTLs are present across *Hevea* species beyond *H. brasiliensis*, supporting their involvement in rubber biosynthesis. Congruent with earlier observations, *Hevea* CPTLs exhibit low homology to CPTs, lacking conserved CPT motifs [26] (Supplementary Fig. S9). Phylogenetic analysis showed *Hevea* CPTLs forming a distinct cluster from those in *P. argentatum, T. brevicorniculatum, L. sativa, S. lycopersicum*, and *A. thaliana* (Fig. 4C).

REF and SRPP proteins, which are abundant in rubber particles, contribute positively to rubber biosynthesis by stabilizing particle structure and preventing coagulation, with REF levels in latex correlating with rubber content [9, 11, 12, 28]. Genome annotation revealed an average of 12 REF/SRPP genes across *Hevea*, with *H. brasiliensis* containing the most (16) and *H. collina* the fewest (7) (Supplementary Table S25). Non-*Hevea* species in Malpighiales and Euphorbiaceae have an average of 4 REF/SRPP genes. Phylogenetic analysis delineated REF/SRPP genes into 5 evolutionary branches, with distinct clusters for *Hevea* genes, particularly in branches I, III, IV, and V (Fig. 4D). Sequence alignments confirmed the presence of the REF domain and noted C-terminal differences between SRPP and REF, consistent with previous reports [12, 28] (Supplementary Fig. S10). Proteomic analysis identified *Hevea* REF1–3 and SRPP1–4 in latex, with REF2 and SRPP1 being the most abundant among latex biosynthesis-related proteins.

### Rubber particle aggregation

In addition to rubber particles, lutoids of vacuolar origin are major components of latex, containing proteins essential for latex coagulation. Upon tapping, latex flow alters turgor pressure, causing lutoid rupture and the release of hevamine/chitinase and β-1,3-glucanase—proteins that promote rubber particle aggregation and latex coagulation [13, 29]. Concurrently, hevein contributes to particle aggregation, ultimately plugging latex vessels and stopping flow. Our study demonstrates a high abundance of chitinase and β-1,3-glucanase in *Hevea* latex proteomes, consistent with their proposed role in rubber coagulation (Supplementary Table S26).

### Jasmonate and ethylene signaling in rubber biosynthesis

Tapping induces a wounding response in phloem tissues that activates the jasmonate and ethylene signaling pathways. Jasmonate signaling is crucial for laticifer differentiation and stimulates the expression of rubber biosynthesis-related genes (e.g., *FPS* and *SRPP*), leading to increased rubber yield [14]. The coronatine-insensitive 1 (COI1)–jasmonate ZIM-domain (JAZ)–MYC transcription factor module is central to this pathway: jasmonate perception leads to the degradation of JAZ repressors via the COI1 complex, releasing MYC2 and activating jasmonate-responsive genes [30]. Components of the COI1–JAZ–MYC2 regulatory module were annotated across *Hevea* species (Fig. 5A). Latex proteomes showed high levels of COI1 and the absence of JAZ, suggesting JAZ degradation and activation of jasmonate-responsive genes.

Ethylene treatment prolongs latex flow by promoting sucrose allocation, water transport, glycolysis, and C3 carbon fixation [15]. While key ethylene signaling components, ethylene receptors, constitutive triple response 1 (CTR1), and ethylene-insensitive 2/3, are annotated in *Hevea* genomes, their low abundance in latex proteomes (Fig. 5A) suggests that ethylene signaling is not the primary response to tapping-induced wounding.

### Differential proteomics reveal potential factors influencing high-capacity rubber biosynthesis

We next identified proteins differentially expressed between *H. brasiliensis* and other *Hevea* species latex (Fig. 5B, Supplementary Fig. S11, and Supplementary Table S27). Glutamine synthetase exhibited the largest fold change, increasing 24.51-fold in *H. brasiliensis* compared with other species. This observation aligns with a study on ethylene treatment, which suggests that the glutamine synthetase/glutamate synthase cycle may constitute the major pathway for protein synthesis during latex regeneration [31]. Metallothionein, enriched 18.65-fold in *H. brasiliensis*, was also among the top differentially expressed proteins. While thiol, ascorbate, and glutathione are major reactive oxygen species (ROS) scavengers in latex [32], metallothionein expression has also been associated with maintaining ROS homeostasis under oxidative stress during tapping and regeneration [33]. Bark storage protein, a nitrogen storage protein not previously linked to latex production, was upregulated by 18.34-fold. The eukaryotic translation initiation factor eIF5A, a member of the broader eIF family involved in protein synthesis initiation, was enriched 17.80-fold in *H. brasiliensis*. Additionally, proteins linked to rubber biosynthesis, including those involved in IPP formation, rubber biosynthesis, rubber particle aggregation, and hormone signaling, were analyzed for differential expression (Supplementary Table S28). Among these, only SRPP2 (2.19-fold), MVK2 (6.22-fold), AACT4 (10.74-fold), and CTR1a (12.4-fold) showed significant enrichment in *H. brasiliensis*.

### Lipidome profiling of *Hevea* species latex

**Figure 6:**
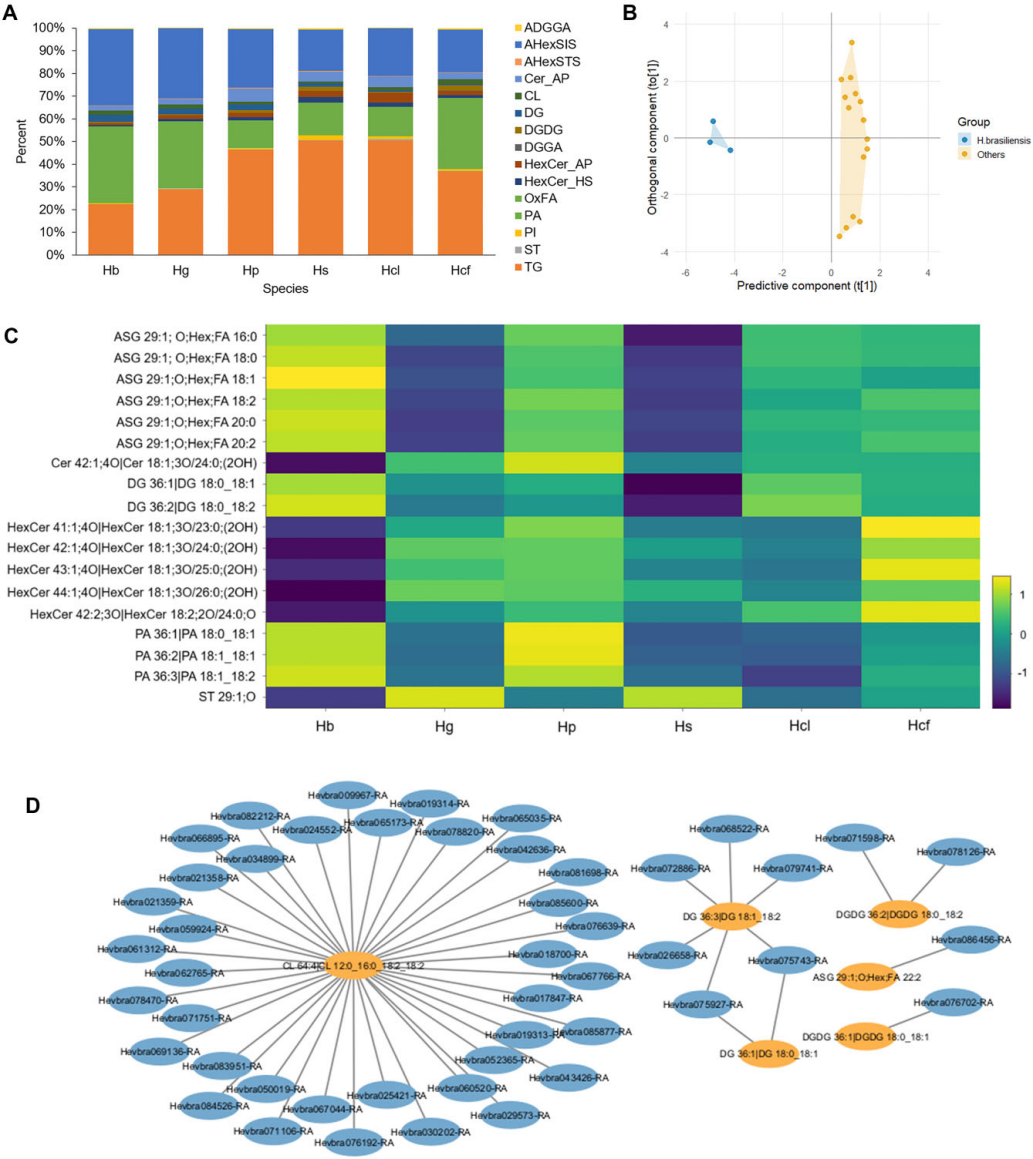
Lipidome profiles across *Hevea* species. (A) Bar plot showing the average composition of lipid classes in the *Hevea* latex lipidome across 3 biological replicates per sample. Hb, *H. brasiliensis*; Hg, *H. guianensis*; Hs, *H. spruceana*; Hcl, *H. collina*; Hcf, *H. confusa*. (B) Orthogonal partial least squares discriminant analysis score plot showing the separation between *H. brasiliensis* and other *Hevea* species samples. (C) Heatmap showing log_10_-transformed average lipid abundance (based on 3 biological replicates), with color coding at which yellow indicates high abundance and blue signifies low abundance. Only differential abundant lipids with VIP > 1 and *P* < 0.05 are shown. (D) Correlation network of proteome and lipidome profiles in *Hevea* latex. Nodes represent proteins (blue) and lipids (orange), and edges represent significant positive correlations (Pearson’s *r* > 0.75, *P* < 0.01). The network illustrates the top 50 most significant protein–lipid associations.

In addition to *cis*-polyisoprene, *Hevea* latex comprises proteins, lipids, and carbohydrates, with lipids known to influence natural rubber’s physical properties [34–36]. To explore this further, a targeted metabolomic analysis was performed to profile the lipid composition of latex from *Hevea* species within the same age range. The lipidomic profiles were analyzed using liquid chromatography–tandem mass spectrometry (LC-MS/MS), identifying 53 lipid molecules classified into 5 main categories: fatty acyls, glycerolipids, glycerophospholipids, sphingolipids, and sterol lipids, which were further divided into 16 classes (Supplementary Tables S29–S30). Triacylglycerol (TG), acylhexosyl sitosterol (AHexSIS), and phosphatidic acid (PA) were the most abundant lipid classes across species (Fig. 6A). *H. guianensis* and *H. confusa* exhibited lipid profiles similar to *H. brasiliensis*, characterized by high PA but lower TG. In contrast, *H. pauciflora, H. spruceana*, and *H. collina* showed a distinct profile with elevated TG and reduced PA. The lipid composition observed in this study differs from that reported previously [37], likely due to differences in sampling and processing protocols. While the prior study used inhibitors to prevent lipid degradation, our samples were collected and transported without chemical preservatives to reflect field-to-factory conditions.

A supervised orthogonal partial least squares discriminative analysis (OPLS-DA) was performed to compare the latex lipidomes of *H. brasiliensis* and other *Hevea* species. The OPLS-DA score plots showed clear separation between the 2 groups, with R²X(cum), R²Y(cum), and Q²(cum) values of 0.80, 0.97, and 0.801, respectively (Fig. 6B). Differential lipid species were identified using OPLS-DA and univariate analysis, selecting those with variable importance in projection (VIP) > 1 and *P* < 0.05. This analysis identified 18 differential lipid molecules, comprising 6 acylated steryl glucosides (ASGs), 1 ceramide (Cer), 2 diacylglycerols (DGs), 5 hexosylceramides (HexCers), 3 PAs, and 1 sterol lipid (ST) (Fig. 6C, Supplementary Table S31). Among these, DG 36:1, DG 36:2, and PA 36:1 exhibited the highest upregulated fold changes, whereas HexCer 41:1, HexCer 43:1, and HexCer 42:1 were the most downregulated in *H. brasiliensis* relative to other *Hevea* species.

To investigate potential functional relationships between proteins and lipids in *Hevea* latex, we performed a correlation analysis between proteome and lipidome profiles across species. Using Pearson correlation with a threshold of *r* > 0.75 and *P* < 0.01, a total of 828 significant positive protein–lipid associations were identified (Supplementary Table S32). From these, the top 50 highly correlated protein–lipid pairs were selected for network visualization using Cytoscape. Notably, several significant correlations involved cardiolipin (CL), a mitochondrial phospholipid, and mitochondrial-associated proteins such as NifU-like, succinate-CoA ligase, and glycine cleavage H. In addition, proteins involved in lipid-related pathways, such as long-chain acyl-CoA synthetase, squalene synthase-like, and shaggy-related protein kinase, showed significant correlations with digalactosyldiacylglycerol (DGDG) and diacylglycerol (DG). These correlations indicate potential coordination between lipid and protein abundance patterns, with several protein–lipid pairs showing strong associations that may reflect shared pathways or subcellular localization.

## Discussion

The genetic diversity among *Hevea* species is crucial for breeding and conservation, offering a reservoir of traits that could be harnessed to improve rubber yield, latex regeneration capacity, and physiological adaptability. However, the limited genetic information available for species other than *H. brasiliensis* poses challenges to fully understanding the evolutionary relationships and functional diversity within the genus. This highlights the importance of identifying shared and species-specific genes across *Hevea* species to better explore and utilize the genetic potential of this economically significant genus.

A pangene analysis of *H. brasiliensis, H. guianensis, H. pauciflora, H. spruceana, H. collina*, and *H. confusa* revealed conservation of ATP binding and ATP hydrolysis activity functions within core and soft-core gene clusters. Simultaneously, gene family size evolution analysis showed expansion in ATP binding and ATP hydrolysis activity gene families in *Hevea*. In *H. brasiliensis*, ATP plays a crucial role in latex production by functioning as a physiological regulator directly in the metabolic pathways and indirectly through the H⁺-ATPase activity. Efficient adenylate turnover supports latex regeneration and rubber yield by driving sucrose conversion to polyisoprene and regulating cytosolic pH [38]. While inorganic phosphorus is a known indicator in latex diagnosis [39], ATP levels in latex also correlate strongly with rubber yield [40, 41], implicating ATP in yield-associated physiological processes. Further evidence supporting the role of ATP in isoprenoid biosynthesis is provided by *T. brevicorniculatum*, a rubber-producing species that serves as a suitable model for functional studies related to latex production. In this species, overexpression of ATP-dependent enzymes in the MVA pathway (acetyl-CoA acetyltransferase and ATP-citrate lyase) resulted in increased accumulation of isoprenoid end products [42]. This prior finding supports the notion that ATP-dependent steps influence isoprenoid flux, consistent with the hypothesis that expansion of ATP-related genes in *Hevea* reflects adaptation to the energetic demands of latex biosynthesis.

The taxonomy of *Hevea* species has long relied on morphological classification, which has not been updated for some time [3, 43]. Morphological approaches are limited by geographic accessibility and confounded by convergent evolution and phenotypic plasticity. Sampling of native *Hevea* species, aside from *H. brasiliensis*, has historically been confined to the Amazon basin [44]. In this study, genomic resources beyond *H. brasiliensis* provide a more robust framework for delineating taxonomic relationships based on genetic relatedness. Phylogenomic analysis of single-copy orthologs revealed 2 distinct *Hevea* subgroups: one comprising *H. brasiliensis, H. pauciflora, H. confusa*, and *H. spruceana*, and the other containing *H. guianensis* and *H. collina*. The divergence between these subgroups, estimated at 6.46 Mya, predates the introduction of *H. brasiliensis* into cultivation outside its native range. This divergence may reflect ecological differentiation, as *H. guianensis* typically grows at higher elevations, while the others thrive in lowland areas [44]. To investigate potential molecular adaptations to this environment, we annotated genes involved in the cold stress regulatory pathway, focusing on the inducer of CBF expression (ICE)–C-repeat binding factor (CBF) transcriptional cascade (Supplementary Text S7). Comparative analysis revealed that *H. guianensis* possesses additional copies of *ICE* and *CBF* relative to *H. brasiliensis* (Supplementary Table S33). This gene expansion may reflect a genomic adaptation to cold stress, supporting the hypothesis that specific genetic changes underpin ecological differentiation.

Characterization of the genetic basis of latex biosynthesis across rubber-producing plants suggests polyphyletic evolution, with convergent mechanisms and enzymes emerging independently in distinct clades [45, 46]. Our phylogenetic analyses of rubber biosynthesis genes in *Hevea* support this, showing *Hevea* CPT genes formed a distinct clade compared to other rubber-producing plants. Similarly, the *Hevea* CPTL and REF/SRPP genes clustered separately in their respective phylogenies. Comparative analyses of CPT and REF/SRPP gene counts indicated that the Euphorbiaceae, including *Hevea*, have undergone CPT gene duplication, whereas REF/SRPP gene duplication occurred exclusively in *Hevea*. The *Hevea* CPT genes have expanded into 2 subclades, with CPT1–3 detected in the latex proteome, clustering within 1 subclade in the phylogeny. This observation suggests that the 2 *Hevea* CPT subclades may have diverged through neofunctionalization, with CPT1–3 likely playing a role in natural rubber metabolism. Similarly, *Hevea* REF/SRPP genes are separated into 3 subgroups, with REF1–3 and SRPP1–4 exclusively detected in the latex proteome. This phylogenetic divergence suggests functional specialization driven by selection, with the latex-specific REF/SRPP genes adapted for rubber particle function.

Proteomic analysis provides complementary insights into metabolic activity by revealing the presence and relative abundance of proteins. In this study, latex proteomics across *Hevea* highlighted key enzymes involved in rubber biosynthesis, spanning IPP formation, rubber chain elongation, particle aggregation, and hormone signaling. The results confirmed the MVA pathway as the primary IPP source, FPS as the main *trans*-prenyltransferase, and CPT1–3, REF1–3, and SRPP1–4 as essential for latex synthesis. Hevamine/chitinase and β-1,3-glucanase are implicated in the rubber particle aggregation, while jasmonate signaling is activated in response to tapping. These proteins represent core rubber biosynthesis components across *Hevea* species. To explore the factors contributing to enhanced rubber biosynthesis in *H. brasiliensis*, differential proteomics analysis revealed several key enriched proteins. These include glutamine synthetase, whose overexpression in *A. thaliana* promotes nitrogen assimilation [47], with nitrogen content positively correlated with latex yield in *H. brasiliensis* [48], suggesting a regulatory link to rubber biosynthesis. Metallothionein, another top-enriched protein, is encoded by a gene induced by ethephon and has been associated with ROS scavenging in tapping panel dryness [49]. The bark storage protein, implicated in nitrogen cycling in *J. curcas* [50], is speculated to support nitrogen demands associated with latex regeneration. The eukaryotic translation initiation factor eIF5A, also known as the rubber biosynthesis stimulator protein, was also enriched and has been shown to promote IPP incorporation into rubber *in vitro* [51]. Given the continuous harvesting of latex and the resulting demand to replenish carbon, nitrogen, and energy reserves, the observed enrichment of proteins involved in nitrogen metabolism, ROS scavenging, and protein synthesis could reflect an adaptive response supporting latex regeneration. These findings raise the hypothesis that the efficiency of rubber biosynthesis in *H. brasiliensis* may be influenced by the molecular capacity for latex regeneration. Among the 51 detected proteins linked to rubber biosynthesis, only SRPP2, MVK2, AACT4, and CTR1a were significantly enriched, suggesting that while the core biosynthetic pathway may be well conserved, latex production could be constrained by regeneration processes. Our proteomic data highlight the importance of latex renewal in sustaining high rubber yield and suggest that future improvements may benefit from targeting upstream physiological capacities rather than the biosynthetic steps alone.

Latex lipidomes further revealed distinct compositional features across *Hevea* species, with TG, AHexSIS, and PA identified as dominant lipid classes. TG functions as a lipid reservoir supporting regeneration [52], while AHexSIS modulates membrane fluidity [53], potentially influencing latex colloidal stability during storage or coagulation. PA, associated with membrane curvature and vesicle formation [54], may influence interparticle interaction during transport and processing. Latex from *H. brasiliensis* exhibited elevated levels of DG 36:1, DG 36:2, and PA 36:1 compared to other *Hevea* species. DG acts as a biosynthetic precursor and signaling molecule [55], and its enrichment suggests more active membrane turnover and stress adaptation. Likewise, higher PA levels may enhance colloidal stability and particle interactions during storage and processing. Since PA is also involved in plant stress signaling, particularly in response to wounding [56], its abundance may reflect an adaptive response to tapping-induced stress. Differences in rubber particle size between species [57] likely contribute to these compositional differences, affecting surface area–dependent lipid interactions and processing behavior.

In addition to compositional differences, correlation analysis between proteome and lipidome profiles provided insights into potential functional interactions. Notably, significant positive correlations were observed between the mitochondrial phospholipid cardiolipin and proteins of mitochondrial origin or function. For instance, NifU-like and succinate-CoA ligase, both implicated in mitochondrial iron–sulfur cluster assembly and energy metabolism [58, 59], showed robust correlations with cardiolipin. These associations reflect coordinated roles in mitochondrial activity in latex-producing cells. Additionally, proteins involved in lipid biosynthesis (long-chain acyl-CoA synthetase) [60], sterol and isoprenoid biosynthesis (squalene synthase-like) [61], and lipid-related signaling (shaggy-related protein kinase alpha) [62] showed significant correlations with glycerolipids, including diacylglycerol and digalactosyldiacylglycerol. These associations raise the possibility of coordinated roles in lipid remodeling, membrane biogenesis, or signaling processes that may contribute to rubber particle formation or function.

Together, the distinct lipid profiles of *Hevea*, alongside its protein–lipid interactions, may underpin adaptive metabolic features that support rubber stability, membrane integrity, and flow resilience, traits crucial for high-quality rubber production.

This study demonstrates how comparative multiomics can be applied to investigate the biological basis of agronomic traits such as latex production in *Hevea* species. By integrating genomic, proteomic, and lipidomic data, we highlight the genetic and biochemical diversity among *Hevea* and speculate on molecular features that may contribute to rubber yield and latex regeneration. Our integrative analyses support the hypothesis that latex productivity and quality in *Hevea* may be influenced by a combination of factors, including expanded ATP-related functions, differential protein abundance related to nitrogen balance, ROS homeostasis, and protein synthesis, as well as distinct lipid profiles associated with membrane remodeling and rubber particle stability. These insights open new avenues for understanding species-specific adaptations and lay the groundwork for targeted functional studies. Importantly, our findings provide a foundational resource to inform future research and breeding strategies aimed at improving rubber production.

## Methods

### DNA extraction and genome sequencing

The sequenced individuals of *H. brasiliensis* (NCBI:txid605986), *H. guianensis* (NCBI:txid882917), *H. pauciflora* (NCBI:txid1318717), *H. spruceana* (NCBI:txid1318718), *H. collina*, and *H. confusa* were maintained at the Rubber Research Institute of Indonesia (Supplementary Text S1). High molecular weight genomic DNA was extracted from young leaves of *H. brasiliensis* using the Genomic-tip 100/G kit (Qiagen). A PacBio library was prepared using the SMRTbell Express Template Prep Kit 2.0 (Pacific Biosciences) according to the manufacturer’s instructions. Sequencing was performed on a PacBio Sequel II with a SMRT Cell 8 M. Raw PacBio subreads were obtained from the SMRT Link v9.0 pipeline (RRID:SCR_002942). For Hi-C sequencing of *H. brasiliensis*, fresh young leaves were ground in liquid nitrogen and fixed with formaldehyde. The Hi-C library was constructed using the Arima Hi-C kit with DpnII and HinfI restriction enzymes, following Kadota et al. [63] (Supplementary Text S2). The library was sequenced on a HiSeq X Ten (Illumina). For short-read sequencing of *H. guianensis, H. pauciflora, H. spruceana, H. collina*, and *H. confusa*, genomic DNA was extracted from leaf tissues using a DNeasy Plant Mini Kit (Qiagen). Sequencing libraries were prepared using the TruSeq DNA Sample Preparation Kit (Illumina) and sequenced on a DNBSEQ platform at BGI-Shenzen (BGI Co. Ltd.).

### RNA sequencing

Total RNA was extracted from the bark, latex, leaf, and petiole of *H. brasiliensis* following the method previously published [64], using a CTAB buffer and lithium chloride precipitation. RNA was quantified using a NanoDrop spectrophotometer (ThermoFisher Scientific) and assessed with a Bioanalyzer 2100 (Agilent Technologies). For Iso-seq, cDNA synthesis was performed using the SMARTer PCR cDNA Synthesis Kit (Clontech). Size fractionation and selection were carried out using the BluePippin Size Selection System (Sage Science). SMRT libraries were prepared using the SMRTbell Express Template Prep Kit 2.0 (Pacific Biosciences) and sequenced on the PacBio Sequel II. Iso-seq data were analyzed using SMRT Link (v9.0), which incorporates read quality filtering, read clustering, consensus calling, and Quiver polishing steps to assemble the Iso-seq reads into high quality, full-length transcripts (Supplementary Text S3).

### Genome assembly and annotation

For *H. brasiliensis*, the long-read assembler Flye (v2.8.2) (RRID:SCR_017016) [65] was used for the assembly and polishing of PacBio reads with the parameter -pacbio-raw. The raw Hi-C reads were adapter and quality trimmed using TrimGalore (v0.6.0). The clean Hi-C reads were mapped to the PacBio assembly using Juicer pipeline (v1.6) (RRID:SCR_017226) [66]. Scaffolds were grouped, ordered, and orientated into pseudo-chromosomes using 3D-DNA (v.180922) (RRID:SCR_017227) with parameters i = 10,000 and r = 4. For *H. guianensis, H. pauciflora, H. spruceana, H. collina*, and *H. confusa*, Illumina reads were trimmed using Platanus_trim (v1.0.7) and assembled using Platanus (v1.2.4) [67]. The Platanus assemblies were synteny scaffolded with RagTag [68] (v2.1.0) using *H. brasiliensis* as reference.

Genome size was estimated based on *k*-mer counting using Jellyfish (v2.3.0) (RRID:SCR_005491). For *H. brasiliensis*, previously sequenced Illumina paired-end reads [22] were used, while for *H. guianensis, H. pauciflora, H. spruceana, H. collina*, and *H. confusa*, short reads generated in this study were analyzed. Genome size was estimated based on 25 *k*-mer with GenomeScope 2.0. Completeness of the genome assembly was assessed using BUSCO (v5) (RRID:SCR_015008) [69] against the Embryophyta odb10 database.

Telomeric and centromeric sequences in *H. brasiliensis* chromosomes were analyzed using quarTeT (v1.2.5). Within quarTeT, the TeloExplorer module, which utilizes the Telomere Identification Toolkit (tidk), was used for telomere detection. Meanwhile, the CentroMiner module incorporated transposable element annotations from the Extensive De Novo TE Annotator (EDTA) as a complementary approach for centromere identification.

Repetitive elements in the *Hevea* species genome were identified by RepeatMasker (v4.1.0) (RRID:SCR_012954) [70] using the *de novo* repeat library and known Viridiplantae repetitive sequences in Dfam and RepBase. The *de novo* repeat library was constructed using RepeatModeler, integrating RECON (v1.0.8), RepeatScout (v1.0.6), and Tandem Repeat Finder (v4.0.9). Genome annotation was performed using the MAKER (v3.01.03) (RRID:SCR_005309) [71] pipeline with *ab initio* gene predictions Augustus (v3.2.2), Snap (v2013-11-29), GeneMark-ES (v3.61), and Fgenesh (v8.0.0b). Iso-seq dataset and assembled transcripts from previously generated RNA-seq [72] were input to MAKER as expressed sequence tag evidence. The functional annotation of the protein-coding genes against NCBI NR, SwissProt, TrEMBL, and KEGG was performed with BLASTP at an *E*-value of 1e^−5^. GO terms and InterPro entries were assigned via OmicsBox (v3.1.9) and InterProScan (v5.52–86.0). Noncoding RNAs were annotated using INFERNAL (v1.1.4) against the Rfam database.

### Clustering of pangene

Pangene across *Hevea*, including *H. brasiliensis, H. guianensis, H. spruceana, H. collina*, and *H. confusa*, was analyzed using GET_HOMOLOGUES-EST (v3.6.2) (RRID:SCR_015705) [73]. All-against-all comparisons were performed using BLAST, followed by clustering with the OrthoMCL algorithm at an inflation value of 1.5. Genes were clustered into core, soft-core, shell, and cloud clusters based on orthogroup frequency. GO enrichment analysis for genes in each cluster was performed using the OmicsBox. The curves describing pangene and core gene sizes were fitted to the Tettelin model.

### Construction of gene families

We downloaded the protein sequences of *A. thaliana, L. usitatissimum, M. esculenta, O. sativa, P. trichocarpa, R. communis*, and *S. purpurea* from Phytozome 13 and the protein sequences of *J. curcas* from Ensembl Plants. Gene families based on all-against-all BLASTP alignment among the 13 plant species were constructed using OrthoFinder (v2.5.5) (RRID:SCR_017118) [74]. Single-copy orthologs for each species within a given orthogroup as analyzed by OrthoFinder were aligned using MAFFT (v7.520), and the resulting alignments were concatenated to create a super alignment matrix. Phylogenetic analysis on the concatenated alignment was conducted using IQ-TREE (v2.2.6) (RRID:SCR_017254) [75] with maximum likelihood and 1,000 ultrafast bootstraps, employing the JTTF + I + R6 model selected by ModelFinder according to the Bayesian information criterion. The phylogenetic tree was visualized using FigTree (v1.4.5). The divergence times in the phylogenetic tree were inferred using the least squares dating method implemented in IQ-TREE. For divergence time estimation, we calibrated the model using the divergence times between *A. thaliana* and *H. brasiliensis* (108 Mya), *L. usitatissimum* and *H. brasiliensis* (88 Mya), and *P. trichocarpa* and *H. brasiliensis* (72 Mya) obtained from the TimeTree database [76].

### Gene family size evolution

The gene family expansion and contraction across 13 plant species were inferred using CAFE5 (RRID:SCR_018924) [77], with the phylogenetic tree constructed by IQ-TREE based on single-copy orthologs as the input tree. The separate birth and death rates of gene families across the phylogeny were estimated using maximum likelihood, and the *P* value threshold of 0.01 was applied to identify significantly expanded or contracted gene families for a given species. These expanded and contracted gene families were subjected to enrichment analysis using OmicsBox with Fisher’s exact test.

### Genome duplication analysis

The genome-wide duplications in the *Hevea* species genomes were analyzed with Tree2GD (v1.0.40) [78]. All-against-all protein alignments were performed with Diamond (v2.1.9), and hierarchical orthogroups were predicted with PhyloMCL (v2.0). Gene trees were reconciled with a reference species tree using the default parameters in Tree2GD. Paralogous gene pairs for each species were identified, and their coding sequences were aligned using MUSCLE (v3.8.31) and pal2nal (v13). The *K*_s_ values of the gene pairs were calculated using KaKs_calculator (v2.0), and the 4DTv values were determined using the Perl script Calculate_4DTV_correction.pl from GitHub. The genome duplication events times of the *Hevea* species were estimated using the formula *T* = *K*_s_/2*r*, where the rate of synonymous substitutions per site per year (*r*) is 7.5 × 10^–9^.

### Proteome characterization

Proteins from the latex samples were extracted as detailed in Supplementary Text S6 and according to the protocol available at protocols.io [79]. Three biological replicates were collected and analyzed for each sample. The peptides were injected onto a 75-µm × 120-mm nanoLC column (Nikkyo Technos) at 50°C and then separated with a gradient (A = 0.1% formic acid [FA] in water, B = 0.1% FA in 80% acetonitrile) consisting of 0–50 min 8% B, 50–57 min 36% B, and 57–60 min 70% B using an UltiMate 3000 RSLCnano LC system (Thermo Fisher Scientific). The eluted peptides were analyzed on an Orbitrap Exploris 480 (Thermo Fisher Scientific) operated in positive ion mode with data-independent acquisition (DIA-MS). MS1 spectra were collected in the range of 495 to 745 *m/z* at a 15,000 resolution, and MS2 spectra were collected in the range of 200 *m/z* and above at a 30,000 resolution with normalized collision energy at 26%. Data were processed using DIA-NN (v1.8.1) (RRID:SCR_022865) [80] for protein and peptide identification and quantification. A spectral library was generated using deep learning–based spectral prediction, and the MS/MS data were searched against the *H. brasiliensis* annotated protein sequences. Parameters were set as follows: enzyme, trypsin; maximum missed cleavage site, 1; static modification, carbamidomethylation; and false discovery rate (FDR) thresholds for precursor and protein identification, 1%.

### Lipid extraction and LC-MS/MS analysis

Latex samples for lipidomic analysis were collected following the same method as for proteomic analysis (Supplementary Text S1), with 3 biological replicates per sample. To simulate real field-to-factory conditions, samples were transported without chemical preservation prior to lipid extraction and LC-MS/MS analysis.

In a previous study, phospholipase A inhibitors such as manoalide, ONO (2-[*p*-amylcinnamoyl]amino-4-chlorobenzoic acid), BEL (bromoenol lactone), and AACOCF3 (trifluoromethyl ketone), along with Triton X-100, were used to prevent lipid degradation [37].

In comparison, our sampling strategy aimed to capture a lipid profile representative of the natural processing timeline, which may differ from profiles obtained under controlled laboratory conditions.

A total of 100 µL of chloroform was added to 1 mg of latex samples, followed by shaking, sonication, and centrifugation. Methanol was then added to 50 µL of the supernatant, stirred, and centrifuged again, and the supernatant was collected. Lysophosphatidylcholine (18:1-d7) was added as an internal standard at a final concentration of 500 ng/mL, and the sample was analyzed using LC-MS/MS. Operational blanks (prepared without samples) and quality control samples (prepared by equally mixing aliquots from each sample solution) were analyzed in the same manner. LC-MS analysis was performed using an UltiMate 3000 BioRS system coupled to an LTQ Orbitrap XL mass spectrometer (Thermo Fisher Scientific). Chromatographic separation was achieved using an L-column3 C18 metal-free column (2.0 mm I.D. × 100 mm, 2-µm particle size; CERI). Data analysis, including peak detection, lipid species estimation within each class, and sample alignment, was conducted using MS-DIAL (v4.80). Lipid peaks were considered detected if they met the following criteria: peak height in quality control samples ≥10,000, peak area at least twice that of the operational blank, average signal-to-noise ratio ≥3, and consistent detection across all quality control measurements with a coefficient of variation <20%. The lipid content was determined relative to an internal standard by comparing the analyte peak area. OPLS-DA was performed using the R package ropls (v1.39.0) (RRID:SCR_016888) to estimate lipid differences between *H. brasiliensis* and other *Hevea* species. Lipid abundances were transformed using log_10_(x + 1) to reduce data skewness and accommodate zero values. VIP scores were obtained from ropls, and *P* values were calculated using a 2-sample *t*-test. Lipids with *P* < 0.05 and VIP > 1.0 were considered differentially abundant.

## Additional Files

**Supplementary Text S1**. Sample collection.

**Supplementary Text S2**. Genome analysis of *Hevea*.

**Supplementary Text S3**. Isoform sequencing.

**Supplementary Text S4**. Background information on the *Hevea* species.

**Supplementary Text S5**. Analysis of genes involved in rubber biosynthesis.

**Supplementary Text S6**. Protein extraction, identification, and quantitative analysis.

**Supplementary Text S7**. Analysis of *ICE-CBF-COR* genes in *Hevea* species.

**Supplementary Fig. S1**. Genome size estimation of *Hevea* species based on *k*-mer analysis. The distributions of unique 25 *k*-mer counts were calculated from paired-end short reads of *H. guianensis, H. pauciflora, H. spruceana, H. collina*, and *H. confusa* generated in this study, as well as from *H. brasiliensis* reads sequenced in a previous study [3].

**Supplementary Fig. S2**. Hi-C interaction heatmap of *H. brasiliensis* genome. The visualization was plotted in Juicebox (v1.11.08).

**Supplementary Fig. S3**. Assessment of genome completeness using BUSCO software.

**Supplementary Fig. S4**. Distribution of GC contents in *Hevea* species genomes. The GC contents were plotted using a 20-Kb sliding window.

**Supplementary Fig. S5**. Comparison of gene structure between *Hevea* species.

**Supplementary Fig. S6**. Age distribution of transposable elements in the *Hevea* species genomes. The Kimura divergence rates of transposable elements were estimated using the “calcDivergenceFromalign.pl” script from the RepeatMasker package, and the TE landscapes were visualized with “createRepeatLandscape.pl.”

**Supplementary Fig. S7**. Genomic clustering of CPT genes on chromosome 18 and REF and SRPP genes on chromosome 8 across *Hevea* species.

**Supplementary Fig. S8**. Multiple alignment of amino sequences of CPTs from *Hevea* species and other rubber-producing plants. Only regions containing conserved CPT domains are shown. Hb, *H. brasiliensis*; Hcf, *H. confusa*; Hcl, *H. collina*; Hg, *H. guianensis*; Hp, *H. pauciflora*; Hs, *H. spruceana*; Pa, *P. argentatum*; Tb, *T. brevicorniculatum*; and Tk, *T. koksaghyz*.

**Supplementary Fig. S9**. Multiple alignment comparing CPTLs from *Hevea* species, *P. argentatum*, and *T. brevicorniculatum* with *H. brasiliensis* CPT1. Only regions showing the conserved domains of *H. brasiliensis* CPT1 are presented. The arrow indicates the start of *H. brasiliensis* CPT1-conserved domains. Hb, *H. brasiliensis*; Hcf, *H. confusa*; Hcl, *H. collina*; Hg, *H. guianensis*; Hp, *H. pauciflora*; Hs, *H. spruceana*; Pa, *P. argentatum*; and Tb, *T. brevicorniculatum*.

**Supplementary Fig. S10**. Multiple alignment of amino sequences of REF/SRPP from *Hevea* species. Only regions containing conserved REF domains and variable C-terminals are shown. Hb, *H. brasiliensis*; Hcf, *H. confusa*; Hcl, *H. collina*; Hg, *H. guianensis*; Hp, *H. pauciflora*; and Hs, *H. spruceana*.

**Supplementary Fig. S11**. Volcano plot showing upregulated (red) and downregulated proteins (blue) in *H. brasiliensis*. The x-axis represents log_2_ fold change, and the y-axis shows −log10 of the *P* value.

**Supplementary Table S1**. Summary of genome sequencing data.

**Supplementary Table S2**. *H. brasiliensis* genome assembly statistics using different assembly and library construction approaches.

**Supplementary Table S3**. Statistics of Hi-C mapping of the *H. brasiliensis* genome.

**Supplementary Table S4**. Statistics of *H. brasiliensis* chromosome-scale pseudomolecules.

**Supplementary Table S5**. Evaluation of gene coverage using PacBio Iso-seq transcripts.

**Supplementary Table S6**. PacBio Iso-seq high-quality consensus isoform reads and annotation statistics across different tissue.

**Supplementary Table S7**. Centromeric regions identified in *H. brasiliensis* chromosomes using QuarTeT.

**Supplementary Table S8**. Telomeric regions identified in *H. brasiliensis* chromosomes using QuarTeT.

**Supplementary Table S9**. Number of gene models with homology or functional classification.

**Supplementary Table S10**. Noncoding RNA genes annotated in the *Hevea* species genomes.

**Supplementary Table S11**. Comparison of repeat composition among *Hevea* species genomes.

**Supplementary Table S12**. Top 20 Gene Ontology enrichment of core genes of the *Hevea* pangenome.

**Supplementary Table S13**. Top 20 Gene Ontology enrichment of soft-core genes of the *Hevea* pangenome.

**Supplementary Table S14**. Top 20 Gene Ontology enrichment of shell genes of the *Hevea* pangenome.

**Supplementary Table S15**. Top 20 Gene Ontology enrichment of cloud genes of the *Hevea* pangenome.

**Supplementary Table S16**. Top 20 Gene Ontology enrichment of expanded gene families in the *Hevea* species genome.

**Supplementary Table S17**. Top 20 Gene Ontology enrichment of expanded gene families in the *H. brasiliensis* genome.

**Supplementary Table S18**. Top 20 Gene Ontology enrichment of contracted gene families in the *Hevea* species genome.

**Supplementary Table S19**. Top 20 Gene Ontology enrichment of contracted gene families in the *H. brasiliensis* genome.

**Supplementary Table S20**. Top 20 most abundant proteins in the latex of *Hevea* species.

**Supplementary Table S21**. Number of genes encoding MVA and MEP pathways, as well as initiator synthesis proteins in *Hevea* species.

**Supplementary Table S22**. Abundance of rubber biosynthesis-related proteins in the latex of *Hevea* species.

**Supplementary Table S23**. Annotation of *cis*-prenyltranferase and *cis*-prenyltranferase-like gene families in *Hevea* species.

**Supplementary Table S24**. Number of orthologous genes encoding proteins related to latex biosynthesis in plant species.

**Supplementary Table S25**. Annotation of rubber elongation factor/small rubber particle protein gene families in *Hevea* species.

**Supplementary Table S26**. Number of genes encoding other rubber biosynthesis-related pathways in *Hevea* species.

**Supplementary Table S27**. Top 20 proteins with the highest fold change in the latex proteome of *H. brasiliensis* compared to other *Hevea* species samples.

**Supplementary Table S28**. Relative changes in the content of proteins involved in rubber biosynthesis in *H. brasiliensis* compared to non–*H. brasiliensis* samples.

**Supplementary Table S29**. Normalized LC-MS/MS peak areas of lipid metabolites detected in latex from *Hevea* species.

**Supplementary Table S30**. Categories, classes, and species of lipids detected in *Hevea* species latex.

**Supplementary Table S31**. Multivariate OPLS-DA analysis of lipids from *H. brasiliensis* and other *Hevea* species samples.

**Supplementary Table S32**. Protein–lipid correlations identified in latex proteome and lipidome analyses.

**Supplementary Table S33**. Annotation of ICE-CBF-COR genes in *Hevea* species.

giaf115_Supplemental_Files

giaf115_Authors_Response_To_Reviewer_Comments_Original_Submission

giaf115_GIGA-D-25-00195_Original_Submission

giaf115_GIGA-D-25-00195_Revision_1

giaf115_Reviewer_1_Report_Original_Submission Marcin Nowicki -- 6/15/2025

giaf115_Reviewer_2_Report_Original_Submission Quentin Cronk -- 6/26/2025

## Abbreviations

AACT: acetyl-CoA C-acetyltransferase; ADGGA: acyl diacylglyceryl glucuronide; AHexSIS: acylhexosyl sitosterol; AHexSTS: acylhexosyl stigmasterol; BUSCO: Benchmarking Universal Single-Copy Orthologs; Cer_AP: ceramide alpha-hydroxy fatty acid-phytospingosine; CL: cardiolipin; CME: 4-(cytidine 5′-diphospho)-2-C-methyl-D-erythritol; CMEC: 2-C-methyl-D-erythritol-2,4-cyclodiphosphate; CMK: 2-C-methyl-d-erythritol 4-phosphate kinase; CMS: 2-C-methyl-D-erythritol 4-phosphate cytidylyltransferase; COI1: coronatine-insensitive 1; CPT: *cis*-prenyltransferase; CPTL: CPT-like; CTR1: constitutive triple response 1; DG: diacylglycerol; DGDG: digalactosyldiacylglycerol; DGGA: diacylglyceryl glucuronide; DMAPP: dimethylallyl pyrophosphate; DPMD: diphosphomevalonate decarboxylase; DXP: 1-deoxy-d-xylulose 5-phosphate; DXR: DXP reductoisomerase; DXS: 1-deoxy-d-xylulose 5-phosphate synthase; EIL1: ethylene insensitive-like 1; EIN2/3: ethylene-insensitive 2/3; ETR: ethylene receptors; FPS: farnesyl pyrophosphate synthase; G3P: glyceraldehyde 3-phosphate; GGPP: geranylgeranyl pyrophosphate; GGPS: geranylgeranyl pyrophosphate synthase; GO: Gene Ontology; GPP: geranyl pyrophosphate; GPS: geranyl pyrophosphate synthase; HDR: 4-hydroxy-3-methylbut-2-enyl diphosphate reductase; HDS: 4-hydroxy-3-methylbut-2-enyl-diphosphate synthase; HMBD: 1-hydroxy-2-methyl-2-butenyl 4-diphosphate; HMGR: hydroxymethylglutaryl-CoA reductase; HMGS: hydroxymethylglutaryl-CoA synthase; IPI: isopentenyl diphosphate isomerase; IPP: isopentenyl diphosphate; JAZ: jasmonate ZIM-domain; KEGG: Kyoto Encyclopedia of Genes and Genomes; MCS: 2-C-methyl-D-erythritol 2,4-cyclodiphosphate synthase; MEP: 2-C-methyl-d-erythritol 4-phosphate; MVK: mevalonate kinase; MYC: MYC transcription factor; MVA-5-p: mevalonate-5-phopshate; MVA-5-pp: MVA 5-diphosphate; OxFA: oxidized fatty acid; PA: phosphatidic acid; PCME: 2-phospho-4-(cytidine 5′-diphospho)-2-C-methyl-D-erythritol; PI: phosphatidylinositol; PMK: phospho-MVA kinase; REF: rubber elongation factor; SRPP: small rubber particle protein; ST: sulfatide; TG: triacylglycerol.

## Data Availability

The data have been submitted under BioProject accession PRJNA1180688. A previously published RNA-seq dataset used in this study is available under BioProject accession PRJDB4387. Genome data are available in the DDBJ/EMBL/GenBank under accession numbers SRR31189420–SRR31189422 (*H. brasiliensis*), SRR31191093 (*H. guianensis*), SRR31191092 (*H. collina*), SRR31192722 (*H. pauciflora*), SRR31192721 (*H. confusa*), and SRR31192751 (*H. spruceana*). Iso-seq data are available under accession numbers SRR31201725 (latex), SRR31201726 (leaf), SRR31203224 (petiole), and SRR31203225 (bark). Proteomic data have been deposited in the ProteomeXchange repository under the accession number PXD057483. Lipidomic data have been submitted to the Metabolomics Workbench under accession ST003958. All additional supporting data are available in the *GigaScience* repository, GigaDB [81].

## References

[1] Metcalfe CR . Distribution of latex in the plant kingdom. Econ Bot. 1967;21:115–27. 10.1007/BF02897859.

[2] van Beilen JB, Poirier Y. Establishment of new crops for the production of natural rubber. Trends Biotechnol. 2007;25:522–29. 10.1016/j.tibtech.2007.08.009. 17936926

[3] Gonçalves PS, Cardoso M, Ortolani A. Origin, variability and domestication of *Hevea*—a review. Pesq Agropec Brasileira. 1990;25:135–56.

[4] Priyadarshan P, Goncalves PS. Use of *Hevea* gene pool in rubber tree (*Hevea brasiliensis* Muell.-Arg) breeding. Planter. 2002;78:123–38.

[5] Priyadarshan PM, Clément-Demange A. Breeding *Hevea* rubber: formal and molecular genetics. Adv Genet. 2004;52:51–115. 10.1016/s0065-2660(04)52003-5. 15522733

[6] Cornish K, Wood DF, Windle JJ. Rubber particles from four different species, examined by transmission electron microscopy and electron-paramagnetic-resonance spin labeling, are found to consist of a homogeneous rubber core enclosed by a contiguous, monolayer biomembrane. Planta. 1999;210:85–96. 10.1007/s004250050657. 10592036

[7] Takahashi S, Koyama T. Structure and function of *cis*-prenyl chain elongating enzymes. Chem Rec. 2006;6:194–205. 10.1002/tcr.20083. 16900467

[8] Lakusta AM, Kwon M, Kwon EG, et al. Molecular studies of the protein complexes involving *cis*-prenyltransferase in guayule (*Parthenium argentatum*), an alternative rubber-producing plant. Front Plant Sci. 2019;10:165. 10.3389/fpls.2019.00165. 30858856 PMC6397875

[9] Yamashita S, Yamaguchi H, Waki T, et al. Identification and reconstitution of the rubber biosynthetic machinery on rubber particles from *Hevea brasiliensis*. eLife. 2016;5:e19022. 10.7554/eLife.19022. 27790974 PMC5110245

[10] Chow K-S, Mat-Isa MN, Bahari A, et al. Metabolic routes affecting rubber biosynthesis in *Hevea brasiliensis* latex. J Exp Bot. 2012;63:1863–71. 10.1093/jxb/err363. 22162870 PMC3295384

[11] Dennis MS, Light DR. Rubber elongation factor from *Hevea brasiliensis*. Identification, characterization, and role in rubber biosynthesis. J Biol Chem. 1989;264:18608–617. 10.1016/S0021-9258(18)51510-6. 2681199

[12] Berthelot K, Lecomte S, Estevez Y, et al. *Hevea brasiliensis* REF (Hev b 1) and SRPP (Hev b 3): an overview on rubber particle proteins. Biochimie. 2014;106:1–9. 10.1016/j.biochi.2014.07.002. 25019490

[13] Wang X, Shi M, Wang D, et al. Comparative proteomics of primary and secondary lutoids reveals that chitinase and glucanase play a crucial combined role in rubber particle aggregation in *Hevea brasiliensis*. J Proteome Res. 2013;12:5146–59. 10.1021/pr400378c. 23991906

[14] Deng X, Guo D, Yang S, et al. Jasmonate signalling in the regulation of rubber biosynthesis in laticifer cells of rubber tree, *Hevea brasiliensis*. J Exp Bot. 2018;69:3559–71. 10.1093/jxb/ery169. 29726901

[15] Liu JP, Zhuang YF, Guo XL, et al. Molecular mechanism of ethylene stimulation of latex yield in rubber tree (*Hevea brasiliensis*) revealed by de novo sequencing and transcriptome analysis. BMC Genomics. 2016;17:257. 10.1186/s12864-016-2587-4. 27008913 PMC4806457

[16] Chao J, Wu S, Shi M, et al. Genomic insight into domestication of rubber tree. Nat Commun. 2023;14:4651. 10.1038/s41467-023-40304-y. 37532727 PMC10397287

[17] Cheng H, Song X, Hu Y, et al. Chromosome-level wild *Hevea brasiliensis* genome provides new tools for genomic-assisted breeding and valuable loci to elevate rubber yield. Plant Biotechnol J. 2023;21:1058–72. 10.1111/pbi.14018. 36710373 PMC10106855

[18] Pootakham W, Sonthirod C, Naktang C, et al. *De novo* hybrid assembly of the rubber tree genome reveals evidence of paleotetraploidy in *Hevea* species. Sci Rep. 2017;7:41457. 10.1038/srep41457. 28150702 PMC5288721

[19] Rahman AY, Usharraj AO, Misra BB, et al. Draft genome sequence of the rubber tree *hevea brasiliensis*. BMC Genomics. 2013;14:75. 10.1186/1471-2164-14-75. 23375136 PMC3575267

[20] Tang C, Yang M, Fang Y, et al. The rubber tree genome reveals new insights into rubber production and species adaptation. Nat Plants. 2016;2:16073. 10.1038/nplants.2016.73. 27255837

[21] Fang Y, Xiao X, Lin J, et al. Pan-genome and phylogenomic analyses highlight *Hevea* species delineation and rubber trait evolution. Nat Commun. 2024;15:7232. 10.1038/s41467-024-51031-3. 39174505 PMC11341782

[22] Lau NS, Makita Y, Kawashima M, et al. The rubber tree genome shows expansion of gene family associated with rubber biosynthesis. Sci Rep. 2016;6:28594. 10.1038/srep28594. 27339202 PMC5008842

[23] Liu J, Shi C, Shi CC, et al. The chromosome-based rubber tree genome provides new insights into spurge genome evolution and rubber biosynthesis. Mol Plant. 2020;13:336–50. 10.1016/j.molp.2019.10.017. 31838037

[24] Cornish K . Similarities and differences in rubber biochemistry among plant species. Phytochemistry. 2001;57:1123–34. 10.1016/S0031-9422(01)00097-8. 11430985

[25] Asawatreratanakul K, Zhang YW, Wititsuwannakul D, et al. Molecular cloning, expression and characterization of cDNA encoding cis-prenyltransferases from *Hevea brasiliensis*. A key factor participating in natural rubber biosynthesis. Eur J Biochem. 2003;270:4671–80. 10.1046/j.1432-1033.2003.03863.x. 14622254

[26] Epping J, van Deenen N, Niephaus E, et al. A rubber transferase activator is necessary for natural rubber biosynthesis in dandelion. Nat Plants. 2015;1:15048. 10.1038/nplants.2015.48.

[27] Niephaus E, Müller B, van Deenen N, et al. Uncovering mechanisms of rubber biosynthesis in *Taraxacum koksaghyz*—role of cis-prenyltransferase-like 1 protein. Plant J. 2019;100:591–609. 10.1111/tpj.14471. 31342578

[28] Oh SK, Kang H, Shin DH, et al. Isolation, characterization, and functional analysis of a novel cDNA clone encoding a small rubber particle protein from *Hevea brasiliensis*. J Biol Chem. 1999;274:17132–38. 10.1074/jbc.274.24.17132. 10358068

[29] Chao J, Chen Y, Wu S, et al. Comparative transcriptome analysis of latex from rubber tree clone CATAS8-79 and PR107 reveals new cues for the regulation of latex regeneration and duration of latex flow. BMC Plant Biol. 2015;15:104. 10.1186/s12870-015-0488-3. 25928745 PMC4410575

[30] Florez-Velasco N, Ramos VF, Magnitskiy S, et al. Ethylene and jasmonate as stimulants of latex yield in rubber trees (*Hevea brasiliensis*): molecular and physiological mechanisms. A systematic approximation review. Adv Agrochem. 2024;3:279–88. 10.1016/j.aac.2024.07.003.

[31] Pujade-Renaud V, Clement A, Perrot-Rechenmann C, et al. Ethylene-induced increase in glutamine synthetase activity and mRNA levels in *Hevea brasiliensis* latex cells. Plant Physiol. 1994;105:127–32. 10.1104/pp.105.1.127. 12232192 PMC159337

[32] Zhang Y, Leclercq J, Montoro P. Reactive oxygen species in *Hevea brasiliensis* latex and relevance to tapping panel dryness. Tree Physiol. 2017;37:261–69. 10.1093/treephys/tpw106. 27903918 PMC5928795

[33] Huang Y, Fang Y, Long X, et al. Characterization of the rubber tree metallothionein family reveals a role in mitigating the effects of reactive oxygen species associated with physiological stress. Tree Physiol. 2018;38:911–24. 10.1093/treephys/tpy003. 29425342

[34] Asghari Barzegar Z, Taghvaei Ganjali S, Malekzadeh M, et al. Correlations between lipid contents of natural rubber and tensile properties of natural rubber-based compound, using attenuated total reflection fourier transform infrared spectroscopy. Spectrosc Lett. 2023;56:1–13. 10.1080/00387010.2022.2153143.

[35] Yu H, Wang Q, Li J, et al. Effect of lipids on the stability of natural rubber latex and tensile properties of its films. J Rubber Res. 2017;20:213–22. 10.1007/BF03449153.

[36] Zhang BL, Huang HH, Wang YZ, et al. Study on molecular structure and property of highly purified natural rubber. J Anal Appl Pyrolysis. 2018;134:130–35. 10.1016/j.jaap.2018.05.018.

[37] Bae SW, Jung S, Choi SC, et al. Lipid composition of latex and rubber particles in *Hevea brasiliensis* and *Taraxacum kok-saghyz*. Molecules. 2020;5110:25.:10.3390/molecules25215110. 33153210 PMC7662343

[38] d’Auzac J, Jacob JL, Chrestin H. Physiology of rubber tree latex. Boca Raton, FL: CRC Press; 1989.;

[39] Chotiphan R, Vaysse L, Lacote R, et al. Can fertilization be a driver of rubber plantation intensification?. Ind Crops Prod. 2019;141:111813. 10.1016/j.indcrop.2019.111813.

[40] Sreelatha S, Simon SP, Jacob JJJRR. On the possibility of using ATP concentration in latex as an indicator of high yield in *Hevea brasiliensis*. J Rubb Res. 2004;7:71–78.

[41] Sreelatha S, Jacob J, Mercykutty VC, et al. ATP concentration in latex as an indicator for early evaluation of yield in *Hevea brasiliensis*. J Plant Crops. 2014;42:48–53.

[42] Pütter KM, van Deenen N, Unland K, et al. Isoprenoid biosynthesis in dandelion latex is enhanced by the overexpression of three key enzymes involved in the mevalonate pathway. BMC Plant Biol. 2017;17:88. 10.1186/s12870-017-1036-0. 28532507 PMC5441070

[43] Schultes RE . A brief taxonomic view of the genus Hevea. Kuala Lumpur: Malaysian Rubber Research and Development Board; 1990.

[44] Priyadarshan PM . Genetic resources. Biology of Hevea rubber. Cham, Switzerland: Springer; 2017:83–105. 10.1007/978-3-319-54506-6.

[45] Lin T, Xu X, Ruan J, et al. Genome analysis of *Taraxacum kok-saghyz* Rodin provides new insights into rubber biosynthesis. Natl Sci Rev. 2018;5:78–87. 10.1093/nsr/nwx101.

[46] Wuyun TN, Wang L, Liu H, et al. The hardy rubber tree genome provides insights into the evolution of polyisoprene biosynthesis. Mol Plant. 2018;11:429–42. 10.1016/j.molp.2017.11.014. 29229569

[47] Zhu C, Zhang G, Shen C, et al. Expression of bacterial glutamine synthetase gene in *Arabidopsis thaliana* increases the plant biomass and level of nitrogen utilization. Biologia (Bratisl). 2015;70:1586–96. 10.1515/biolog-2015-0183.

[48] An F, Xie G, Jiang J, et al. Nutrient conditions of rubber olantations and their relationship with latex yield under stimulated tapping system in Xishuangbanna. Chin J Trop Crop. 2005;26:1–6.

[49] Zhu J, Zhang Q, Wu R, et al. *HbMT2*, an ethephon-induced metallothionein gene from *Hevea brasiliensis* responds to H_2_O_2_ stress. Plant Physiol Biochem. 2010;48:710–15. 10.1016/j.plaphy.2010.04.004. 20471279

[50] Zhang MJ, Fu Q, Chen MS, et al. Characterization of the bark storage protein gene (*JcBSP*) family in the perennial woody plant *Jatropha curcas* and the function of *JcBSP1* in *Arabidopsis thaliana*. PeerJ. 2022;10:e12938. 10.7717/peerj.12938. 35186503 PMC8833228

[51] Yusof F, Chow K-S, Ward MA, et al. A stimulator protein of rubber biosynthesis from *Hevea brasiliensis* latex. J Rubber Res. 2000;3:232–49.

[52] Yang Y, Benning C. Functions of triacylglycerols during plant development and stress. Curr Opin Biotechnol. 2018;49:191–98. 10.1016/j.copbio.2017.09.003. 28987914

[53] Dufourc EJ . Sterols and membrane dynamics. J Chem Biol. 2008;1:63–77. 10.1007/s12154-008-0010-6. 19568799 PMC2698314

[54] Kooijman EE, Chupin V, de Kruijff B, et al. Modulation of membrane curvature by phosphatidic acid and lysophosphatidic acid. Traffic. 2003;4:162–74. 10.1034/j.1600-0854.2003.00086.x. 12656989

[55] Carrasco S, Mérida I. Diacylglycerol, when simplicity becomes complex. Trends Biochem Sci. 2007;32:27–36. 10.1016/j.tibs.2006.11.004. 17157506

[56] Testerink C, Munnik T. Phosphatidic acid: a multifunctional stress signaling lipid in plants. Trends Plant Sci. 2005;10:368–75. 10.1016/j.tplants.2005.06.002. 16023886

[57] Ong CW, Shamsul Bahri AR. Rubber particles: size, molecular weight and their distributions detected in wild *Hevea* species. J Biol Agric Healthc. 2016;6:98–103.

[58] Couturier J, Touraine B, Briat JF, et al. The iron-sulfur cluster assembly machineries in plants: current knowledge and open questions. Front Plant Sci. 2013;4:259. 10.3389/fpls.2013.00259. 23898337 PMC3721309

[59] Zhang Y, Fernie AR. The role of TCA cycle enzymes in plants. Adv Biol. 2023;7:e2200238. 10.1002/adbi.202200238. 37341441

[60] Zhao H, Kosma DK, Lü S. Functional role of long-chain acyl-coA synthetases in plant development and stress responses. Front Plant Sci. 2021;12:640996. 10.3389/fpls.2021.640996. 33828572 PMC8019973

[61] Unland K, Pütter KM, Vorwerk K, et al. Functional characterization of squalene synthase and squalene epoxidase in *Taraxacum koksaghyz*. Plant Direct. 2018;2:e00063. 10.1002/pld3.63. 31245726 PMC6508512

[62] Dornelas MC, Van Lammeren AA, Kreis M. *Arabidopsis thaliana* SHAGGY-related protein kinases (AtSK11 and 12) function in perianth and gynoecium development. Plant J. 2000;21:419–29. 10.1046/j.1365-313x.2000.00691.x. 10758494

[63] Kadota M, Nishimura O, Miura H, et al. Multifaceted Hi-C benchmarking: what makes a difference in chromosome-scale genome scaffolding? Gigascience. 2020;9:giz158. 10.1093/gigascience/giz158. PMC6952475 31919520

[64] Deng LH, Luo MW, Zhang CF, et al. Extraction of high-quality RNA from rubber tree leaves. Biosci Biotechnol Biochem. 2012;76:1394–96. 10.1271/bbb.120014. 22785464

[65] Kolmogorov M, Yuan J, Lin Y, et al. Assembly of long, error-prone reads using repeat graphs. Nat Biotechnol. 2019;37:540–46. 10.1038/s41587-019-0072-8. 30936562

[66] Durand NC, Shamim MS, Machol I, et al. Juicer provides a one-click system for analyzing loop-resolution hi-C experiments. Cell Syst. 2016;3:95–98. 10.1016/j.cels.2016.07.002. 27467249 PMC5846465

[67] Kajitani R, Toshimoto K, Noguchi H, et al. Efficient de novo assembly of highly heterozygous genomes from whole-genome shotgun short reads. Genome Res. 2014;24:1384–95. 10.1101/gr.170720.113. 24755901 PMC4120091

[68] Alonge M, Lebeigle L, Kirsche M, et al. Automated assembly scaffolding using RagTag elevates a new tomato system for high-throughput genome editing. Genome Biol. 2022;23:258. 10.1186/s13059-022-02823-7. 36522651 PMC9753292

[69] Manni M, Berkeley MR, Seppey M, et al. BUSCO update: novel and streamlined workflows along with broader and deeper phylogenetic coverage for scoring of eukaryotic, prokaryotic, and viral genomes. Mol Biol Evol. 2021;38:4647–54. 10.1093/molbev/msab199. 34320186 PMC8476166

[70] Tarailo-Graovac M, Chen N. Using RepeatMasker to identify repetitive elements in genomic sequences. Curr Protoc Bioinformatics. 2009;25:Unit 4.10. 10.1002/0471250953.bi0410s25. 19274634

[71] Campbell MS, Law M, Holt C, et al. MAKER-P: a tool kit for the rapid creation, management, and quality control of plant genome annotations. Plant Physiol. 2014;164:513–24. 10.1104/pp.113.230144. 24306534 PMC3912085

[72] Makita Y, Ng KK, Veera Singham G, et al. Large-scale collection of full-length cDNA and transcriptome analysis in *Hevea brasiliensis*. DNA Res. 2017;24:159–67. 10.1093/dnares/dsw056. 28431015 PMC5397604

[73] Contreras-Moreira B, Cantalapiedra CP, García-Pereira MJ, et al. Analysis of plant pan-genomes and transcriptomes with GET_HOMOLOGUES-EST, a clustering solution for sequences of the same species. Front Plant Sci. 2017;8:184. 10.3389/fpls.2017.00184. 28261241 PMC5306281

[74] Emms DM, Kelly S. OrthoFinder: phylogenetic orthology inference for comparative genomics. Genome Biol. 2019;20:238. 10.1186/s13059-019-1832-y. 31727128 PMC6857279

[75] Minh BQ, Schmidt HA, Chernomor O, et al. IQ-TREE 2: new models and efficient methods for phylogenetic inference in the genomic era. Mol Biol Evol. 2020;37:1530–34. 10.1093/molbev/msaa015. 32011700 PMC7182206

[76] Kumar S, Suleski M, Craig JM, et al. TimeTree 5: an expanded resource for species divergence times. Mol Biol Evol. 2022;39:msac174. 10.1093/molbev/msac174. 35932227 PMC9400175

[77] Mendes FK, Vanderpool D, Fulton B, et al. CAFE 5 models variation in evolutionary rates among gene families. Bioinformatics. 2021;36:5516–18. 10.1093/bioinformatics/btaa1022. 33325502

[78] Chen D, Zhang T, Chen Y, et al. Tree2GD: a phylogenomic method to detect large-scale gene duplication events. Bioinformatics. 2022;38:5317–21. 10.1093/bioinformatics/btac669. 36218394

[79] Lau N, Okubo-Kurihara E, Makita Y, et al. Sample preparation of Hevea brasiliensis for proteomic analysis by LC-MS/MS. protocols.io. 2025. 10.17504/protocols.io.ewov1mwr7vr2/v1. (assesed May 2025).

[80] Demichev V, Messner CB, Vernardis SI, et al. DIA-NN: neural networks and interference correction enable deep proteome coverage in high throughput. Nat Methods. 2020;17:41–44. 10.1038/s41592-019-0638-x. 31768060 PMC6949130

[81] Lau N, Okubo-Kurihara E, Makita Y, et al. Supporting data for “Comparative Genomics and Multiomics Analyses Reveal the Evolution and Physiological Basis of Rubber Biosynthesis in Hevea Species.” GigaScience Database. 2025. 10.5524/102755. PMC12512020 41070844

