# Supplementary material for: Comparative genomics and multiomics analyses reveal the evolution and physiological basis of rubber biosynthesis in Hevea species
Source: Gigascience. 2025 Oct 10;14:giaf115. doi: 10.1093/gigascience/giaf115 (PMC12512020; doi:10.1093/gigascience/giaf115)
Supplement: giaf115_Supplemental_Files [file giaf115_supplemental_files.zip › Supplementary Material_Figures_Comparative genomics Hevea.docx]

**Supplementary Materials**

**Supplementary Text S1. Sample collection.**

RRIM600 is a historically important *H. brasiliensis* clone that was widely used in early breeding programs and has contributed significantly to the genetic diversity of modern rubber tree cultivars [1]. It remains one of the most extensively studied clones and serves as a valuable reference for genetic and physiological research. We selected RRIM600 for genome assembly to represent its foundational role in *Hevea* breeding and to support future comparative and functional studies.

All samples used in this study were obtained from mature, seven-year-old *H. brasiliensis* and other *Hevea* species grown at the same location within the plantation of the Rubber Research Institute of Indonesia. Leaf samples were collected for genomic DNA sequencing, while bark, latex, leaf, and petiole samples were used for RNA sequencing. Latex samples were also harvested for proteomic and lipidomic analyses. To standardize latex sampling, each tree was tapped once on the same morning, and 2 mL of latex was collected immediately after tapping. The trees are maintained as a species collection for research purposes; they were not part of a commercial plantation and thus were not regularly tapped.

**Supplementary Text 2. Genome analysis of *Hevea*.**

The previously published genome assembly of *H. brasiliensis* RRIM600, generated with sequencing data from Illumina and PacBio platforms, comprised 189,316 scaffolds spanning 1.55 Gb, with a scaffold N50 of 67.24 Kb. In this study, we present a chromosome-scale assembly of *H. brasiliensis* for in-depth comparative analyses. The genome of *H. brasiliensis* was sequenced using PacBio and Hi-C approaches (Supplementary Table S1). The assembly of PacBio reads was attempted using Flye, wtdbg2 and Canu software (Supplementary Table S2). The assembly generated using Flye, which exhibited the longest N50 and a total scaffold size close to the genome size estimated by *k*-mer, was selected for subsequent analysis. The Hi-C library construction was attempted using either the iconHi-C protocol with the HindIII restriction enzyme [2] or the Arima Genomics Kit (Supplementary Table S3). The Arima Hi-C library exhibited a higher number of valid interaction read pairs. The observed difference is thought to be attributable to the difference in the frequency of restriction sites. Therefore, we consider the Arima kit as more suitable to *H. brasiliensis*, and the Arima H-C assembly was adopted for the final genome assembly of this species. The final assembly has a length of 1.71 Gb with a scaffold N50 of 78.35 Mb, representing an improvement in scaffold N50 value of compared to the previously published genome assembly of RRIM600 [3]. The statistics of the current RRIM600 assembly genome are comparable to the recently published genome assemblies of RY73397 [4], CATAS8-79 [5] and MT/VB/25A 57/8 [6].

We estimated genome heterozygosity across *Hevea* species using GenomeScope, revealing variation from relatively low levels (1.11% to 1.22) in *H. brasiliensis*, *H. pauciflora*, and *H. confusa* to higher levels (3.65% to 3.76) in *H. guianensis*, *H. spruceana*, and *H. collina*.

**Supplementary Text S3. Isoform sequencing.**

Isoform sequencing (Iso-Seq) was performed on *H. brasiliensis* to support genome annotation and provide expressed sequence tag evidence for MAKER analysis. For MAKER genome annotation, both previously published RNA-seq data [7] and Iso-Seq transcripts generated in this study were used. The transcriptomes of bark, latex, leaf, and petiole samples were previously sequenced using the Illumina platform. In this study, PacBio Iso-Seq was also performed on total RNA extracted from these tissues. The raw PacBio reads were processed with the IsoSeq3 bulk workflow (<https://isoseq.how/getting-started.html>, lima v2.9.0, isoseq v4.0.0, pbmm2 v1.13.1 , and pigeon v1.2.0) to generate full-length non-chimeric (FLNC) reads. These FLNC reads were mapped to the *H. brasiliensis* genome assembly to assess the completeness of gene coverage (Supplementary Table S5). Subsequently, the FLNC reads were clustered and polished to generate high-quality consensus isoforms. In total, 52,016, 37,595, 47,666, and 64,498 unique isoforms were identified from bark, latex, leaf, and petiole, respectively (Supplementary Table S6). Of these, 97.8–98.4% were annotated, 1.3–2.2% were classified as novel, and less than 0.22% were antisense of annotated genes. The majority of transcripts from each tissue exhibited only 1–2 isoforms per gene.

**Supplementary Text S4. Background information on the *Hevea* species.**

The genus *Hevea* and its type species *H. guianensis* were first described in 1775 by the French botanist Jean Baptiste Christophore Fusée Aublet. The name *Siphoni*a was introduced in 1779 by L.C. Richard as a synonym for *Hevea*, while H.F. Gmelin proposed *Caoutchoua* as a replacement for *Hevea* in 1781. In 1854, Betham proposed five new species within the genus. Schultes (1977) [8] reviewed the infrageneric classification of *Hevea* and *Siphoni*a, and recognised two subgenera, *Hevea* and *Microphylla*e. The number of recognised species of *Heve*a has changed over time, from 11 [9] to 24 species [10], then to eight [11], and finally to the present 11 species.

*Hevea guianensis* Aublet, which was first described in 1775, is the type species of the genus and likely represents one of the earliest concepts within *Hevea*. It has a large distribution range that spans the entire geographic region of the genus, including Brazil, Venezuela, Bolivia, French Guiana, Peru, Colombia, Suriname, and Ecuador [12]. It typically thrives in well-drained soils or at higher altitudes. *H. guianensis* are medium-sized trees with short shoots that suberect mature leaflets [13]. The trees are exploited for latex in the wild but are not cultivated.

The taxonomic history of *Hevea collina* has been subject to various interpretations by different botanists over the years [14]. Huber first described *H. collina* in 1909, noting the similarities to *H. guianensis*, but distinguishing them based on leaf differences, inflorescence location, and staminate buds shape. Pax accepted this classification in 1910 and placed it in the section Euhevea. Ducke initially considered *H. collina* to be a local variety of *H. guianensis*, but later regarded it as a synonym of *H. guianensis*.

*Hevea brasiliensis* (Willd. ex. A. de. Juss.) Muell.-Arg. was first named as *S. brasiliensis* in 1824 by Jussieu, who also published a plate with detailed drawings of the specimen. *H. brasiliensis* is native to regions south of the Amazon River, including Brazil, Bolivia, Ecuador, and Peru. This species thrives mainly in well-drained areas but can also grow in locations with occasional brief or light flooding, such as high riverbanks. *H. brasiliensis* are tall trees that undergo “winter”, with trunks that sometimes swell in the wild. *H. brasiliensis* is a highly valuable crop, presently cultivated worldwide for the production of natural rubber.

*Hevea pauciflora* (Spr. ex Bth.) Muell.-Arg. was described in 1854 based on the Spruce 2691 collection from the Uapés in Brazil [14]. This species is found north and west of the Amazon River, encompassing regions in Brazil, Guyana, and Peru [12]. It thrives in well-drained soils and rocky hillsides. *H. pauciflora* consists of small to medium-sized trees with short shoots and tough leaves [13]. This species has not been exploited for rubber due to the high resin content in its latex.

The initial recognition of the *H. pauciflora* variant, *H. confusa*, was made by Hemsley in 1989, based on collections from the Schomburgk brothers, Prestoe and Jenman in British Guiana. This classification was accepted by Huber and Baldwin. However, a subsequent review by Seibert, Ducke and Schultes considered *H. confusa* as a synonym of *H. pauciflora* var. coriacea [14].

*Hevea spruceana* (Bth.) Muell.- Arg, described by Bentham in 1854 and named in honour of Spruce, was based on material collected in the Amazon below Santarém [14]. This species lines the banks of the Amazon and is also found along the lower Madeira, the Rio Negro, and the lower reaches of other major tributaries [12]. It grows on the muddy soils of islands and riverbanks that are frequently inundated. *H. spruceana* consists of medium-sized trees with short shoots, and their trunks are sometimes swollen [13]. Similar to *H. pauciflora*, *H. spruceana* has not been exploited due to the high resin content in its latex.

**Supplementary Text S5. Analysis of genes involved in rubber biosynthesis.**

To annotate genes related to rubber biosynthesis in the *Hevea* species genome, homologous gene and protein sequences were retrieved from the previously published *H. brasiliensis* genome annotation, as well as from *H. brasiliensis* and *Arabidopsis thaliana* sequences from the NCBI database. The retrieved protein sequences were used as queries in a BLASTP search against the annotated proteins of *H. brasiliensis, H. guianensis, H. pauciflora, H. spruceana, H. collina*, and *H. confusa*. The best hits were filtered with an e-value cutoff of 1e^-5^, minimum of 70% identity and 70% coverage. In total, 72-110 candidate genes related to rubber biosynthesis were annotated across the *Hevea* species genomes, including 5-12 genes in initiator synthesis, 14-18 genes in the MVA pathway, 14-22 genes in the MEP pathway, 6-9 genes from the CPT/ CPTL family, 7-16 genes from the REF/ SRPP family, 10-16 genes associated with rubber particle aggregation, 5-6 genes involved in jasmonate signalling, and 9-16 genes involved in ethylene signalling. Phylogenetic analysis of CPT amino acid sequences from *Hevea* species and other plants including *Taraxacum koksaghyz*, *Taraxacum brevicorniculatum*, and *Parthenium argentatum* was performed using IQ-TREE (v2.2.6) with maximum likelihood and 1,000 ultrafast bootstraps, employing the LG+I+G4 model selected according to the Bayesian Information Criterion. A maximum likelihood tree for CPTL nucleotide sequences from *Hevea* and other species including *P. argentatum, T. brevicorniculatum, Lactuva sativa, Solanum lycopersicum,* and *A. thaliana* with the human NogoB receptor as an outgroup, was generated using 1,000 ultrafast bootstraps and the TPM2u+F+I model. Similarly, a maximum likelihood tree for REF and SRPP amino acid sequences from *Hevea* and other species such as *A. thaliana*, *P. argentatum, T. brevicorniculatum, L. sativa* and *S. lycopersicum* was constructed with 1,000 ultrafast bootstraps using the VT+G4 model. Multiple alignments of CPT amino acid sequences from *Hevea* species and other rubber-producing plants including *P. argentatum*, *T. brevicorniculatum*, and *T. koksaghyz*, were performed using the CLC genomics workbench (v23.0). Additionally, multiple alignments comparing CPTL nucleotide sequences from *Hevea* species, *P. argentatum*, and *T. brevicorniculatum*, as well as alignments of REF/SRPP amino acid sequences from *Hevea* species, were conducted using the same tool.

**Supplementary Text S6. Protein extraction, identification and quantitative analysis**

Latex samples were homogenised in a buffer containing 100 mM Tris (pH 8.0), 4% SDS, and 20 mM NaCl. The mixtures were sonicated for 30 min, rotated for 24 h, and this process of sonication and rotation was repeated once. Protein concentration was determined using the bicinchoninic acid (BCA) assay and adjusted to 0.115 µg/µL with the same buffer as above. Disulfide bonds were reduced with 200 mM tris(2-carboxyethyl)phosphine at 80 °C for 10 min, followed by alkylation with 30 mM iodoacetamide at room temperature in the dark for 30 min. The alkylated samples were mixed with single-pot solid-phase-enhanced sample preparation beads and ethanol [15], incubated for 20 min and digested overnight with trypsin/Lys-C (Promega) at 37 °C. The reaction was quenched with 5% trifluoroacetic acid (TFA) and the peptides were desalted using a reversed-phase spin column (GL-Tip SDB). The peptides were reconstituted in 2% acetonitrile and 0.1% TFA, and the concentration was measured by BCA assay. A total of 500 ng of extracted peptides from each sample were subjected to LC-MS/MS analysis.

In this study, 40,641 peptides were identified from the latex proteome, including 29,599 unique peptides, with a false discovery rate of 1%. These peptides matched 6,690 protein groups annotated from the *H. brasiliensis* reference genome. On average, each protein was matched by 4.4 peptides, with only 6.2% of proteins being identified by a single peptide. Among the most abundant proteins identified in latex included superoxide dismutase (SOD), glutathione *S*-transferase (GST) and profilin-6 (Supplementary Table S20). Reactive oxygen species (ROS)-scavenging enzymes, such as SOD and GST, are crucial for breaking down the harmful by-products of oxidative stress [16, 17]. Specifically, SOD is suggested to play a role in maintaining the lutoid integrity and promoting latex flow by preventing damage from superoxide radicals [17]. Profilin, a cytoskeletal actin-binding protein, has been identified as one of the allergens present in latex [18]. The most abundant proteins in our latex proteome have also been consistently reported as abundant in other latex transcriptome and proteome studies, underscoring the reliability and reproducibility of our dataset [19-21].

Differential abundance analysis was performed using the limma package (v3.58.1), which fits a linear model with empirical Bayes moderation. Limma is one of the preferred differential expression analysis tool on proteomics data for high-performing workflows reviewed in Peng et al. [22]. Protein abundance data were normalized using the normalizeBetweenArrays function in limma, and the normalized values were log_2_-transformed prior to statistical analysis. Missing values were imputed with the minimum observed value in the dataset. *P*-values were adjusted for multiple testing using the Benjamini-Hochberg method. Fold changes were calculated as the ratio of protein abundances in *H. brasiliensis* RRIM 600 compared to other *Hevea* species (*H. guianensis, H. pauciflora, H. spruceana, H. collina,* and *H. confusa*), with an adjusted *p*-value < 0.05 and a log_2_ fold change greater than 0.2. Among the 6,690 quantifiable proteins, 1,082 (16.2%) were significantly upregulated, and 783 (11.7%) were downregulated in *H. brasiliensis* (Supplementary Fig. S11). Of these, 1,479 proteins exhibited a log_2_ fold change greater than 0.2, with 696 upregulated and 783 downregulated in *H. brasiliensis*.

**Supplementary Text S7. Analysis of *ICE*-*CBF*-*COR* genes in *Hevea* species.**

The inducer of CBF expression (ICE)-C-repeat binding factor (CBF) transcriptional cascade is one of the most extensively studied cold regulatory pathways in *A. thaliana* [23]. Under cold stress. CBF transcription factors are induced and bind to the promoters of *cold-regulated* (*COR*) genes to activate their expression, thereby conferring cold tolerance [24-26]. Inducer of CBF expression 1 (ICE1), the best-characterised upstream regulator in this pathway, acts as a master transcriptional activator by directly binding to CBF promoters and promoting their expression.

In *Hevea brasiliensis*, the ICE–CBF–COR pathway has been shown to be functional, with key components such as HbICE1 [27] and HbCOR47 [28] previously characterised. In this study, we annotated *ICE*, *CBF* and *COR* genes in *Hevea* species using the following method.

First, a Hidden Markov Model (HMM) profile was constructed based on protein sequences from *A. thaliana*. Candidate genes were identified using a threshold of *E*-value < 1 × 10⁻⁵ and ≥ 50% sequence identity. Predicted protein sequences were then checked for conserved domains using the NCBI Conserved Domain Database (http://www.ncbi.nlm.nih.gov/Structure/cdd/wrpsb.cgi).


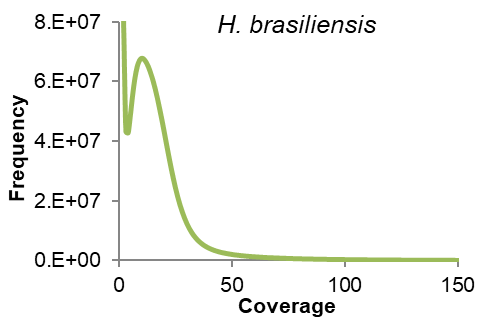

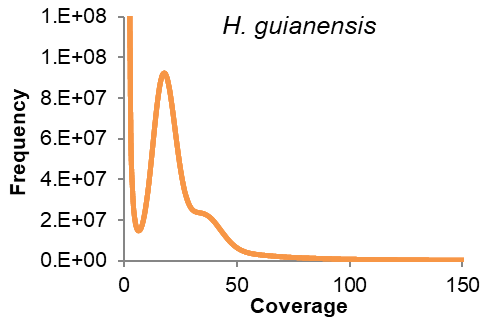

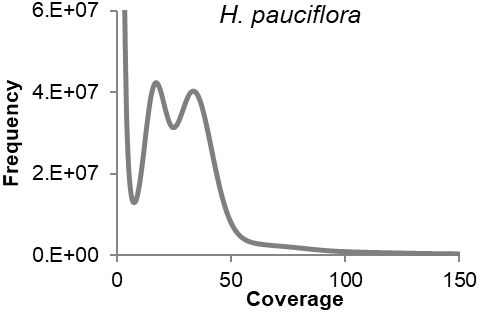

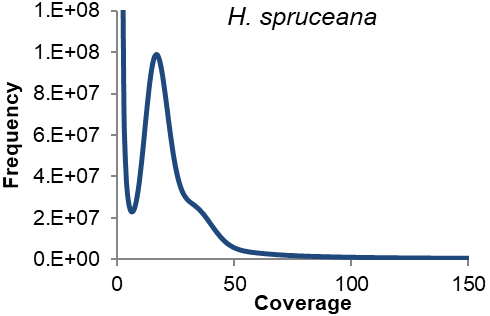

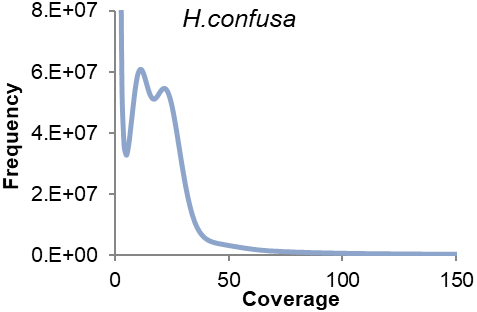

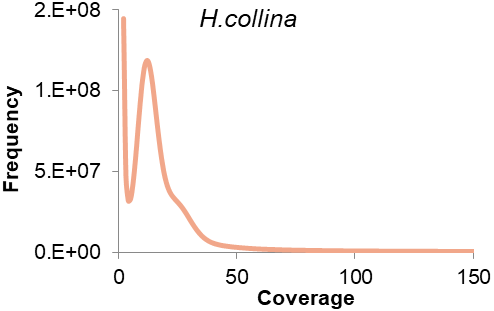


| Species | Genome size (Gb) | Repeat length (Gb) | Unique length (Gb) | Heterozygosity (%) | Model Fit (%) |
| --- | --- | --- | --- | --- | --- |
| *H*. *brasiliensis* | 2.036 | 1.069 | 0.967 | 1.11 | 98.94 |
| *H*. *guianensis* | 1.696 | 0.769 | 0.927 | 3.65 | 97.37 |
| *H*. *pauciflora* | 1.760 | 0.774 | 0.986 | 1.15 | 98.63 |
| *H*. *spruceana* | 1.755 | 0.754 | 1.001 | 3.76 | 96.78 |
| *H*. *collina* | 1.782 | 0.875 | 0.907 | 3.65 | 98.50 |
| *H*. *confusa* | 1.885 | 0.902 | 0.983 | 1.22 | 99.28 |

**Figure S1**: Genome size estimation of *Hevea* species based on *k*-mer analysis. The distributions of unique 25 *k*-mer counts were calculated from paired-end short reads of *H. guianensis, H. pauciflora, H. spruceana, H. collina* and *H. confusa* generated in this study, as well as from *H. brasiliensis* reads sequenced in a previous study [3].


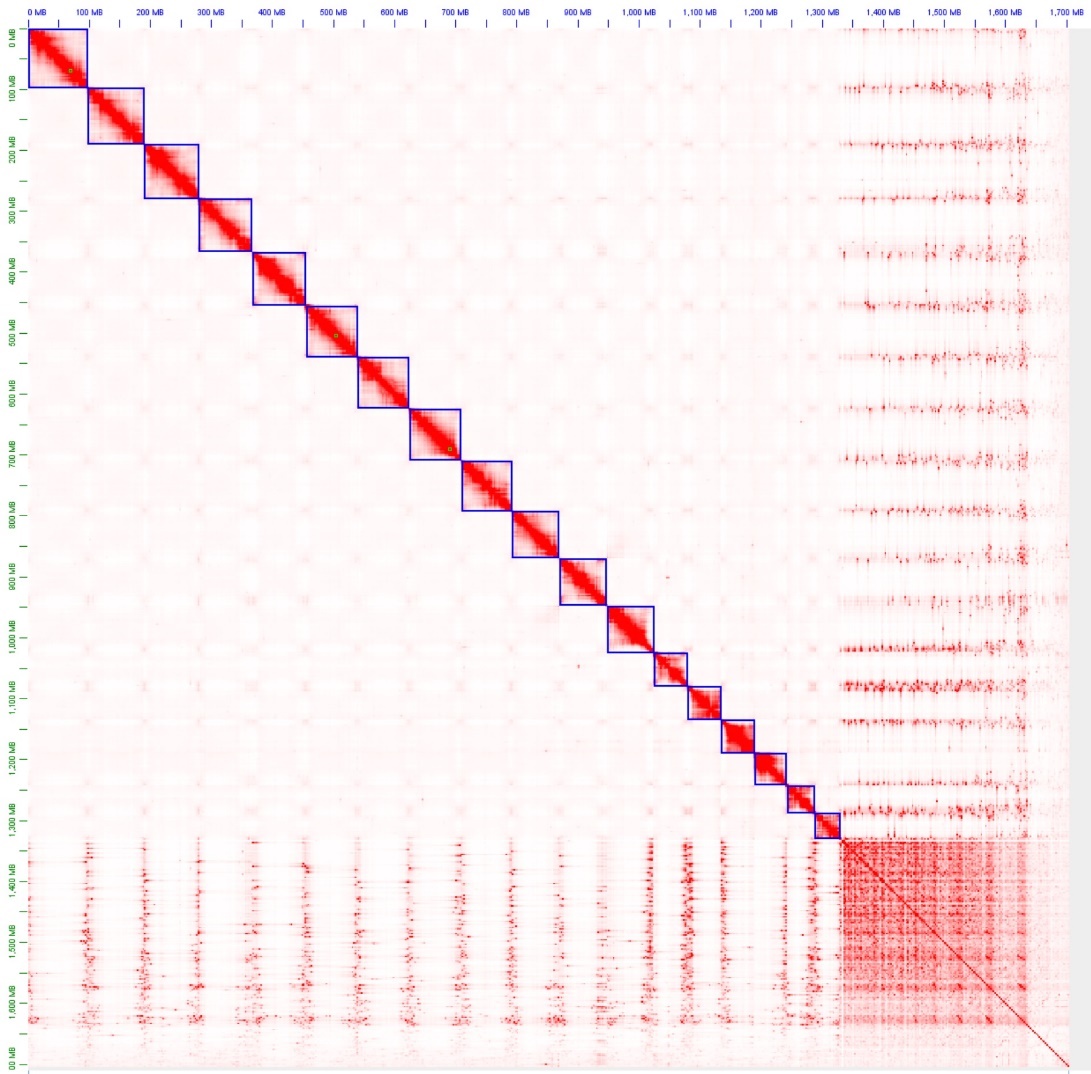


**Figure S2:** Hi-C interaction heatmap of *H. brasiliensis* genome. The visualisation was plotted in Juicebox (v1.11.08).

**
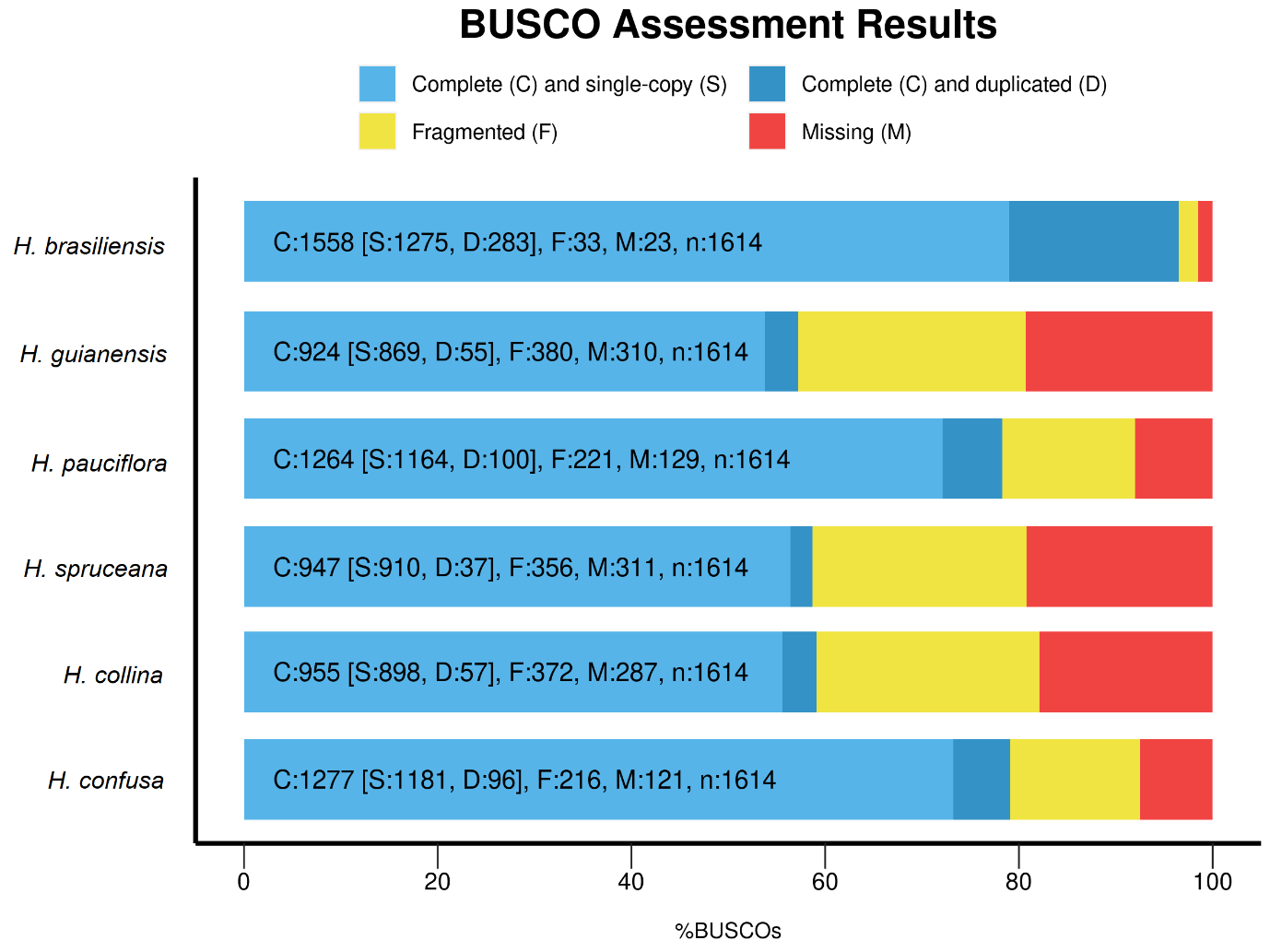
**

**Figure S3**: Assessment of genome completeness using BUSCO software.

**Figure S4**: Distribution of GC contents in *Hevea* species genomes. The GC contents were plotted using a 20Kb sliding window.


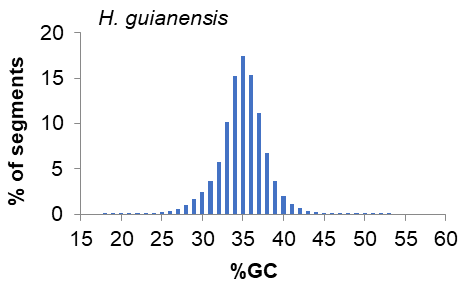

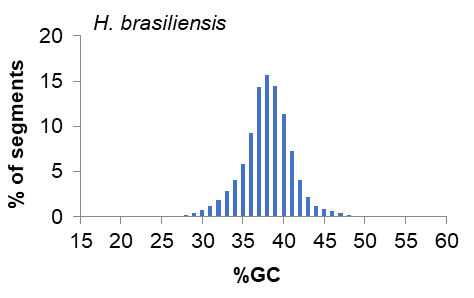

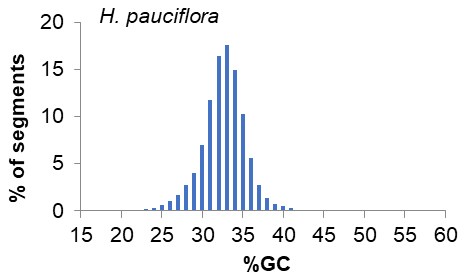

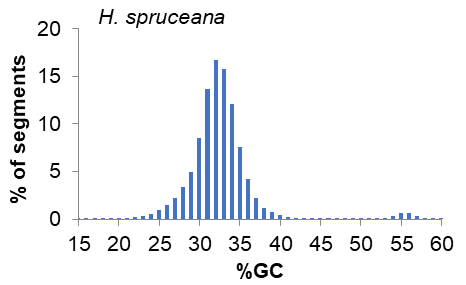

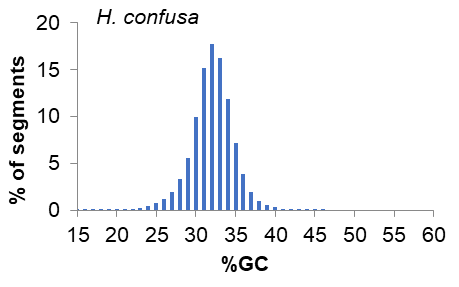

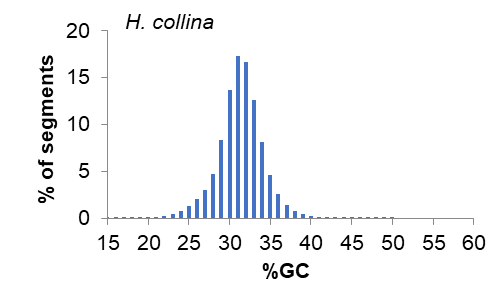

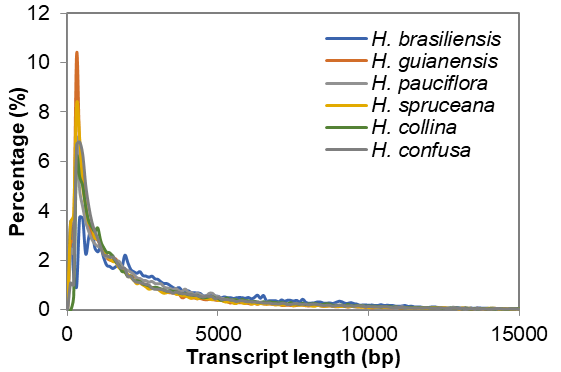

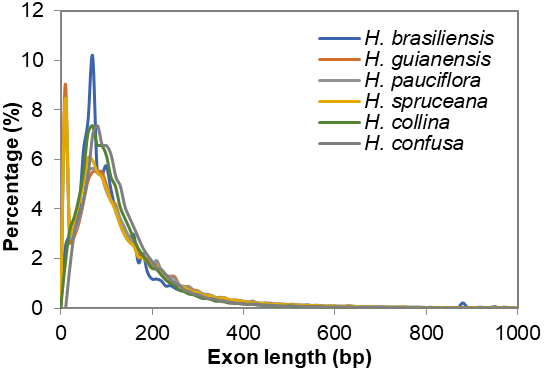

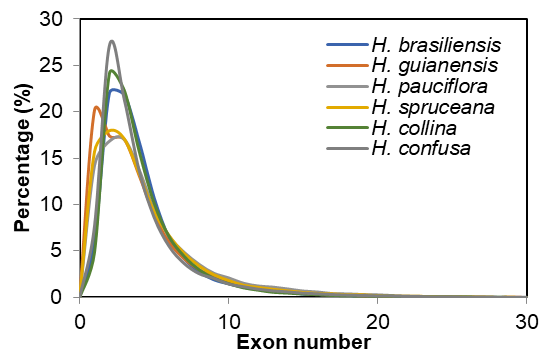

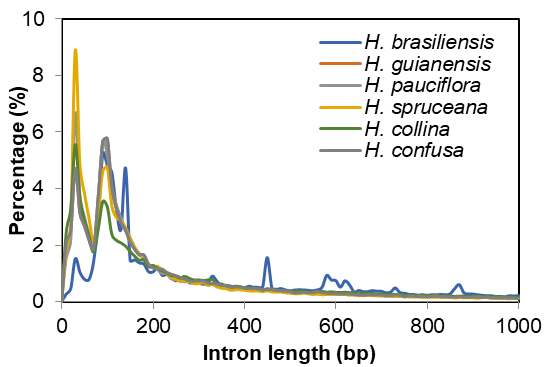

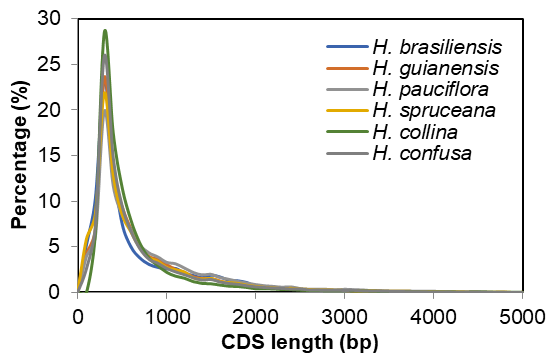


**Figure S5**: Comparison of gene structure between *Hevea* species.


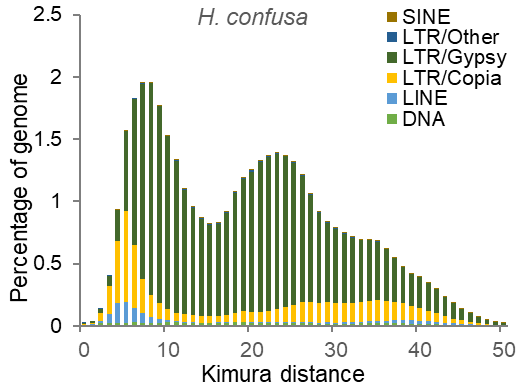

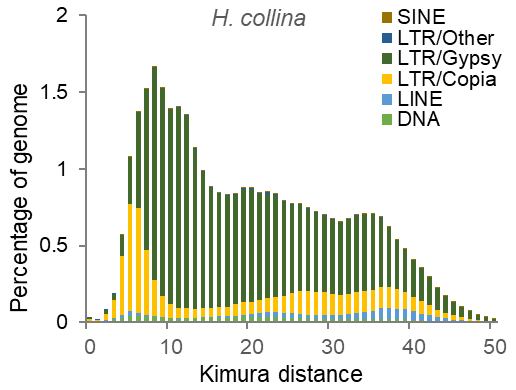

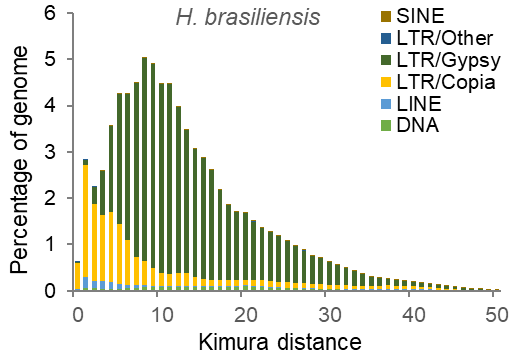

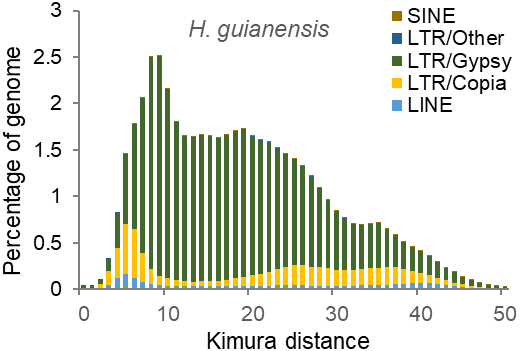

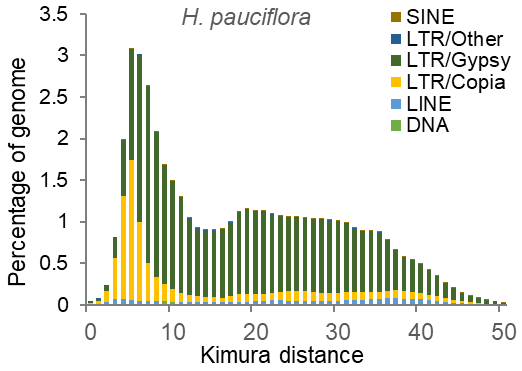

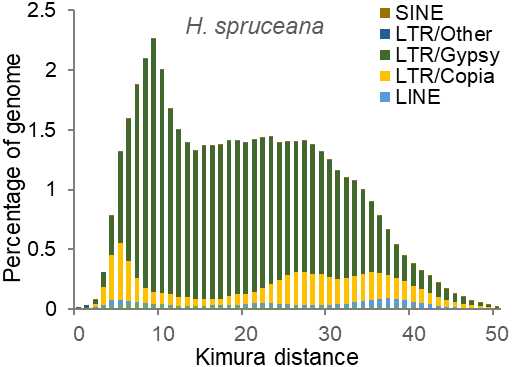


**Figure S6**: Age distribution of transposable elements in the *Hevea* species genomes. The Kimura divergence rates of transposable elements were estimated using the ‘calcDivergenceFromalign.pl’ script from the RepeatMasker package, and the TE landscapes were visualised with ‘createRepeatLandscape.pl’.


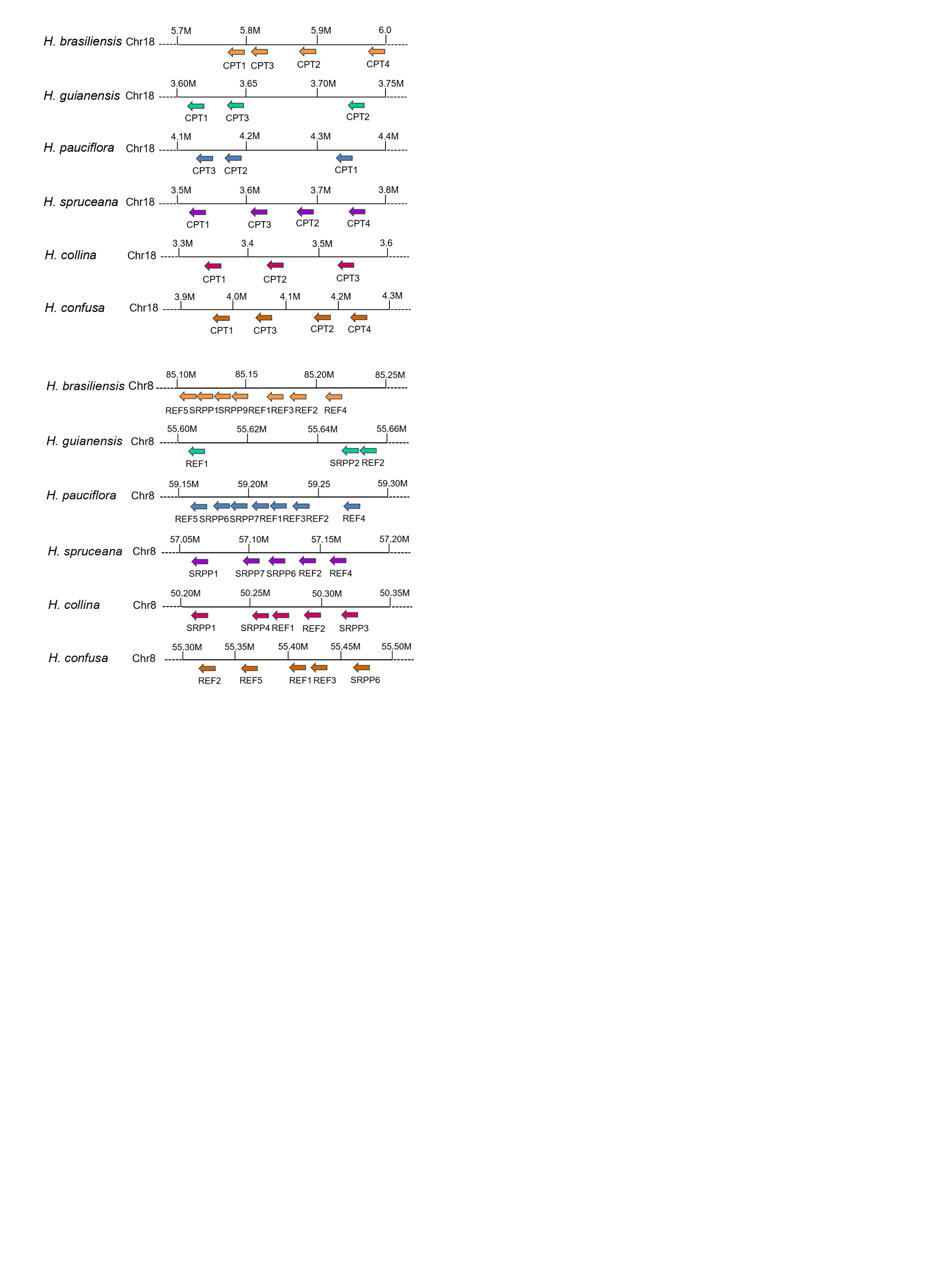


**Figure S7**: Genomic clustering of CPT genes on chromosome 18 and REF and SRPP genes on chromosome 8 across *Hevea* species.

I

II

III


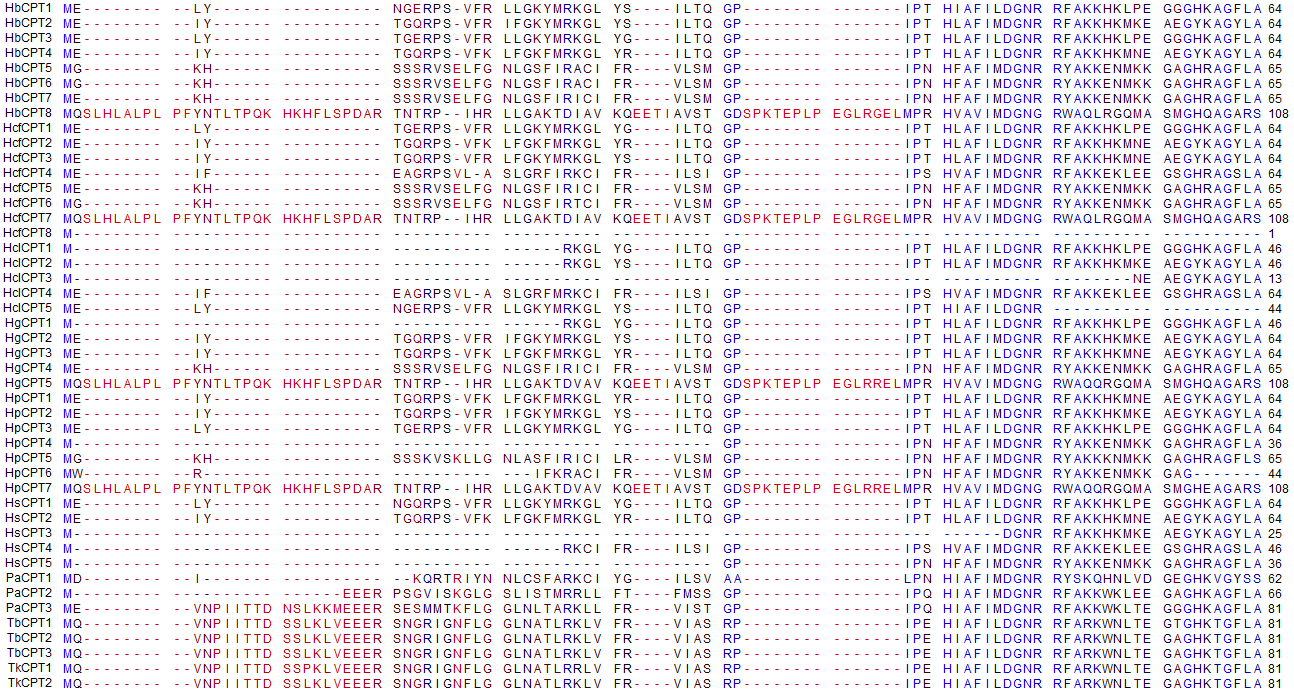

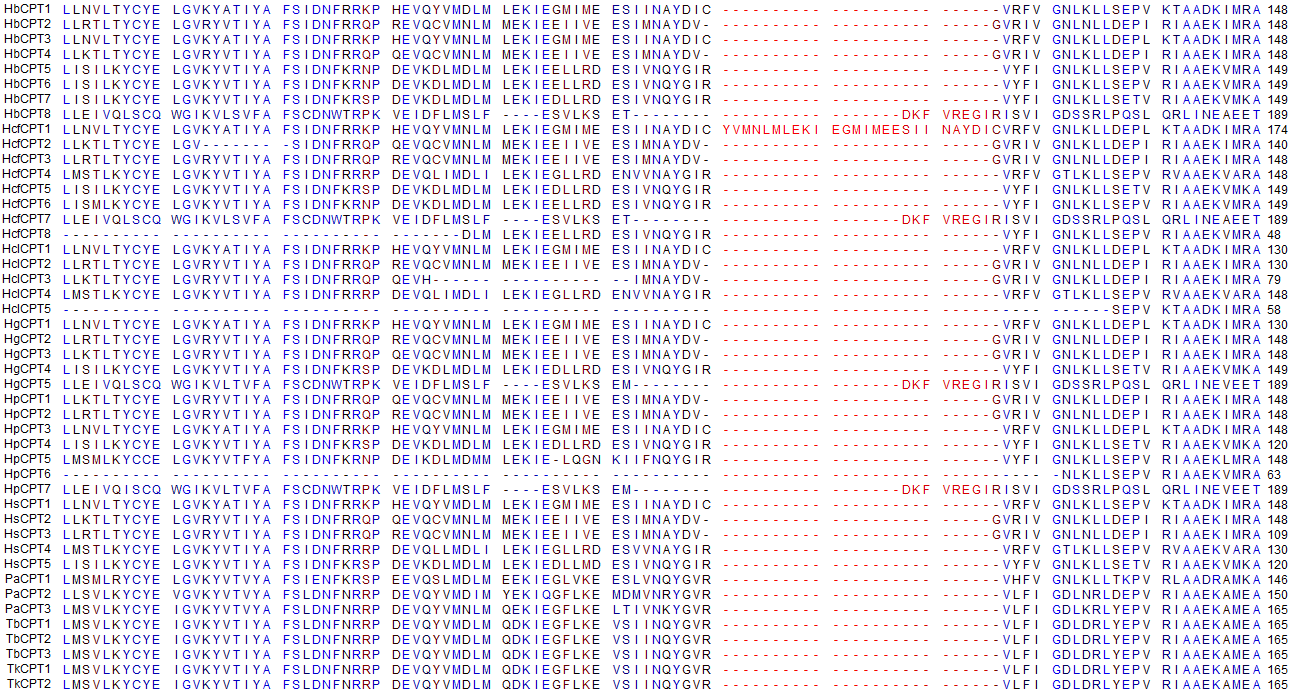


IV

V


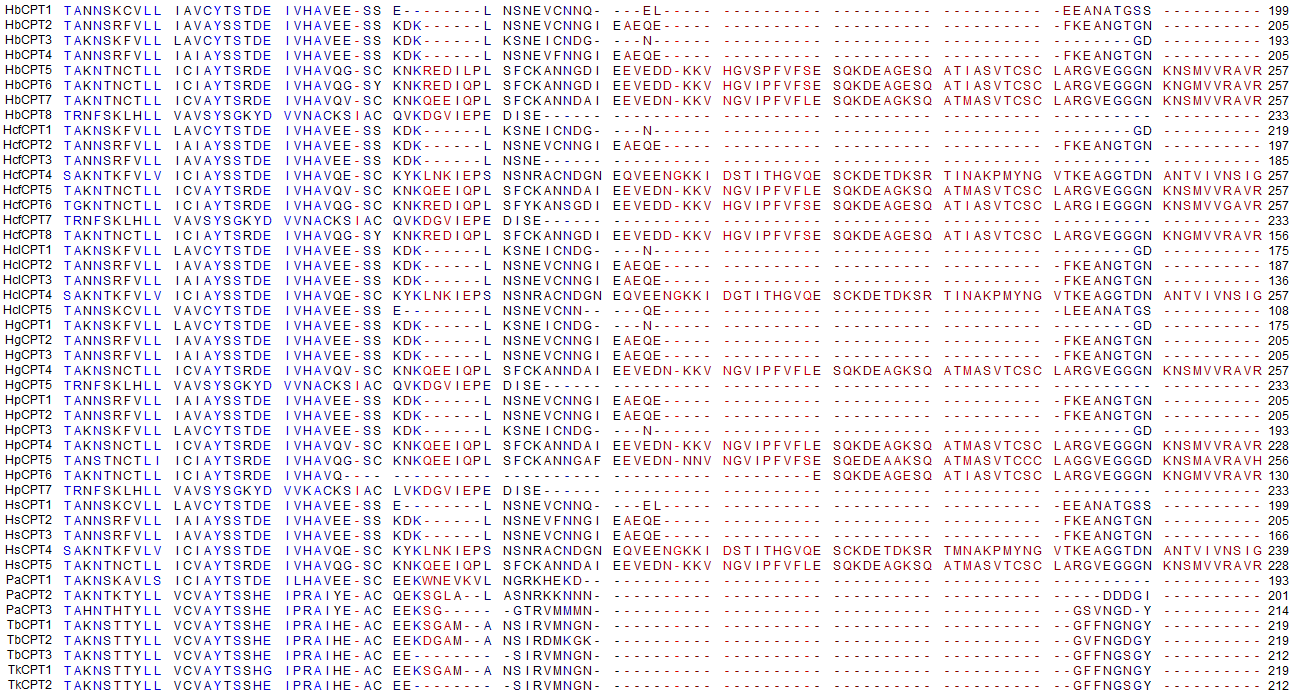

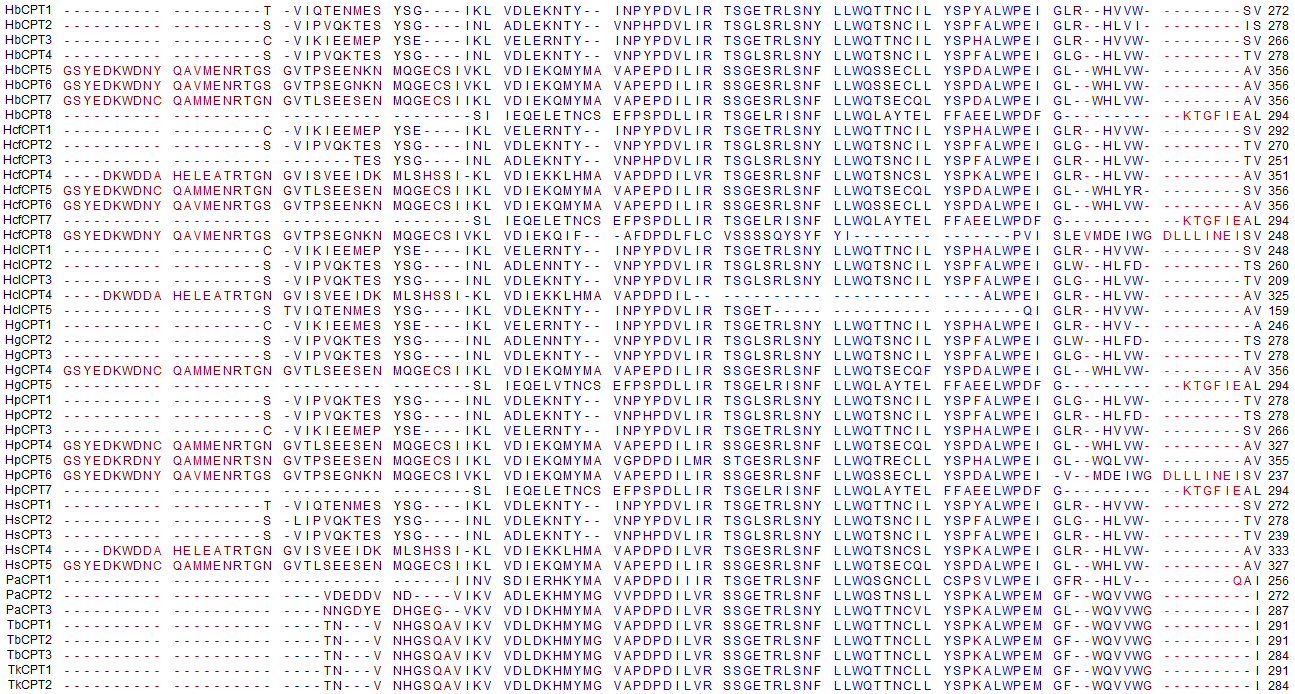


**Figure S8**: Multiple alignment of amino sequences of CPTs from *Hevea* species and other rubber-producing plants. Only regions containing conserved CPT domains are shown. Hb, *H. brasiliensis*; Hcf, *H. confusa*; Hcl, *H. collina*; Hg, *H. guianensis*; Hp, *H. pauciflora*; Hs, *H. spruceana*; Pa, *P. argentatum*; Tb, *T. brevicorniculatum,* and Tk, *T. koksaghyz*.


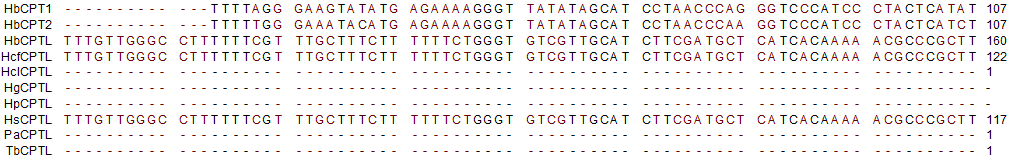

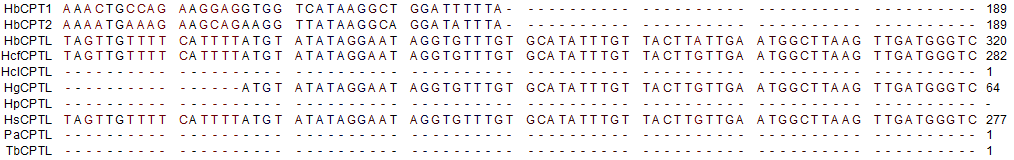

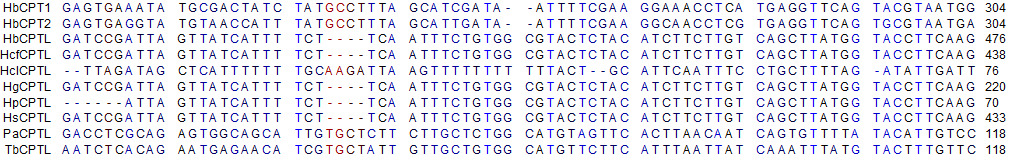

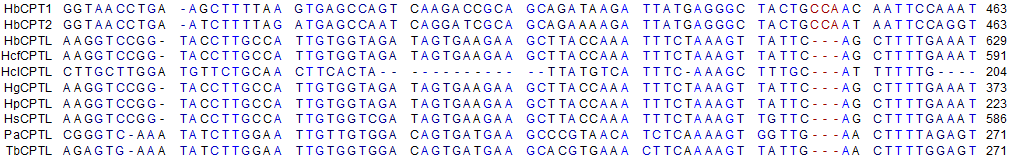

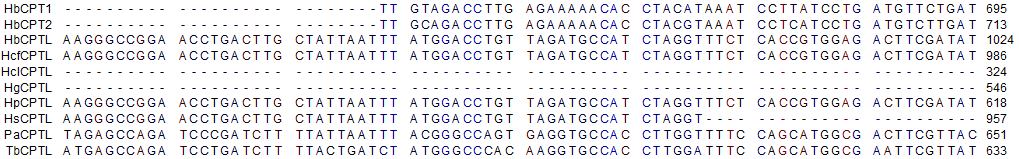


**Figure S9:** Multiple alignment comparing CPTLs from *Hevea* species, *P. argentatum* and *T. brevicorniculatum* with *H. brasiliensis* CPT1. Only regions showing the conserved domains of *H. brasiliensis* CPT1 are presented. The arrow indicates the start of *H. brasiliensis* CPT1 conserved domains. Hb, *H. brasiliensis*; Hcf, *H. confusa*; Hcl, *H. collina*; Hg, *H. guianensis*; Hp, *H. pauciflora*; Hs, *H. spruceana*; Pa, *P. argentatum*; and Tb, *T. brevicorniculatum*.


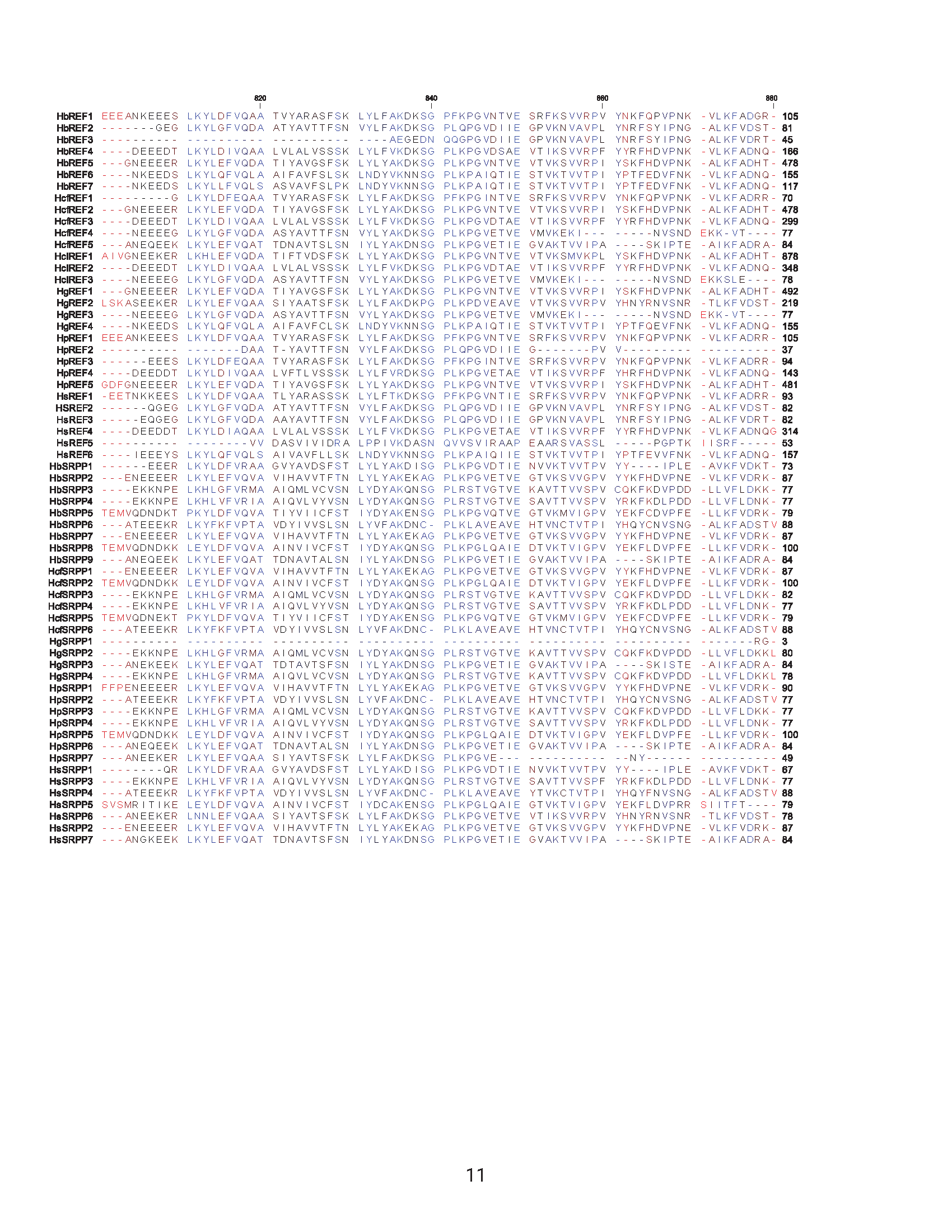


REF domain


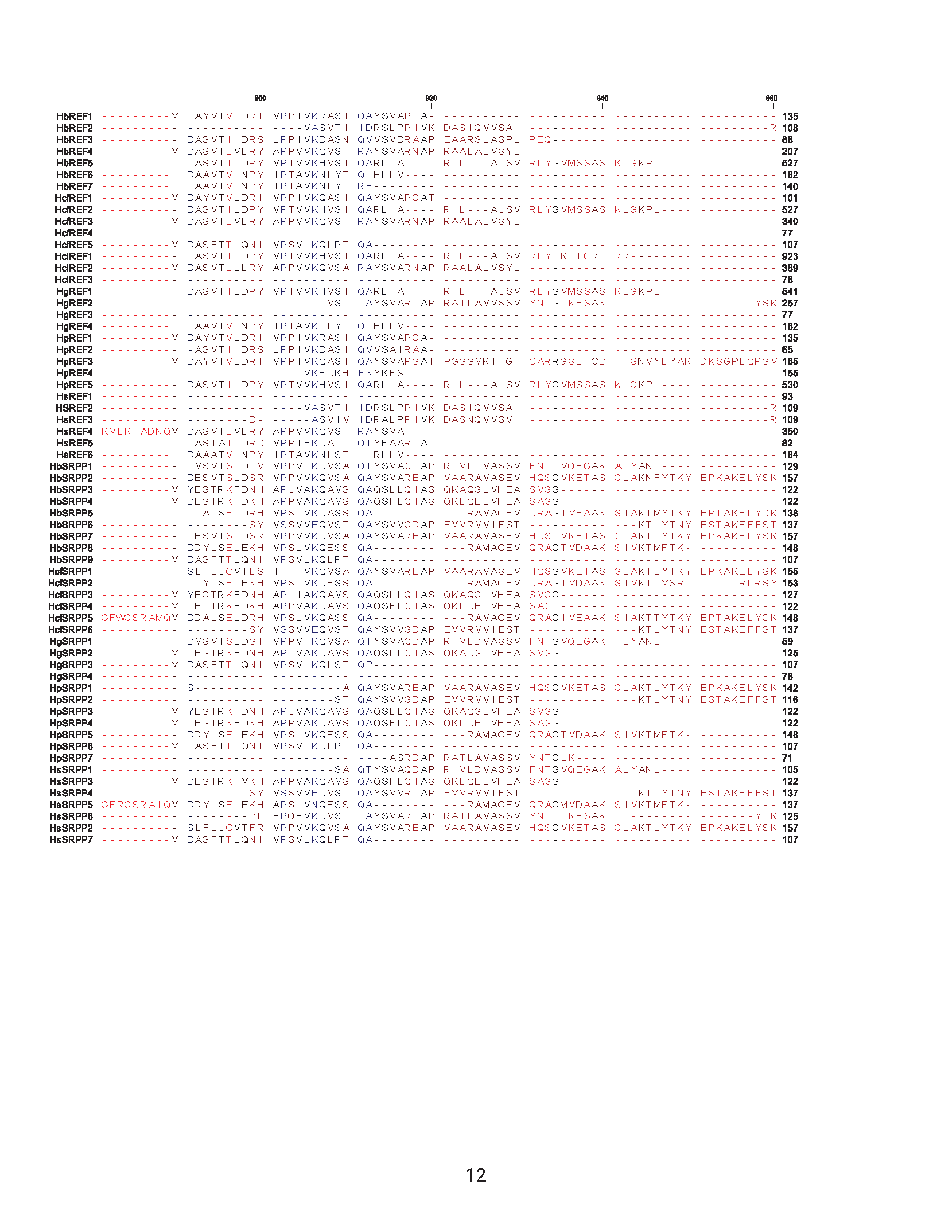


C-terminal

**Figure S10**: Multiple alignment of amino sequences of REF/SRPP from *Hevea* species. Only regions containing conserved REF domains and variable C-terminals are shown. Hb, *H. brasiliensis*; Hcf, *H. confusa*; Hcl, *H. collina*; Hg, *H. guianensis*; Hp, *H. pauciflora*; and Hs, *H. spruceana*.

**
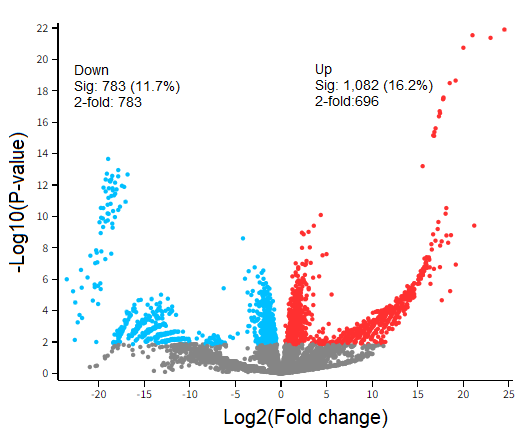
**

**Figure S11**: Volcano plot showing upregulated (red) and downregulated proteins (blue) in *H. brasiliensis*. The x-axis represents log2 fold change, and the y-axis shows -log10 of the *p*-value.

**References**

1. Priyadarshan PM, Gonçalves PS and Omokhafe KO. Breeding *Hevea* rubber. In: Jain SM and Priyadarshan PM, editors. Breeding plantation tree crops: Tropical species. New York, NY: Springer New York; 2009. p. 469-522.

2. Kadota M, Nishimura O, Miura H, Tanaka K, Hiratani I and Kuraku S. Multifaceted Hi-C benchmarking: what makes a difference in chromosome-scale genome scaffolding? Gigascience. 2020;9 https://doi.org/10.1093/gigascience/giz158.

3. Lau NS, Makita Y, Kawashima M, Taylor TD, Kondo S, Othman AS, et al. The rubber tree genome shows expansion of gene family associated with rubber biosynthesis. Sci Rep. 2016;6:28594. https://doi.org/10.1038/srep28594.

4. Fang Y, Xiao X, Lin J, Lin Q, Wang J, Liu K, et al. Pan-genome and phylogenomic analyses highlight *Hevea* species delineation and rubber trait evolution. Nat Commun. 2024;15:7232. https://doi.org/10.1038/s41467-024-51031-3.

5. Chao J, Wu S, Shi M, Xu X, Gao Q, Du H, et al. Genomic insight into domestication of rubber tree. Nat Commun. 2023;14:4651. https://doi.org/10.1038/s41467-023-40304-y.

6. Cheng H, Song X, Hu Y, Wu T, Yang Q, An Z, et al. Chromosome-level wild *Hevea brasiliensis* genome provides new tools for genomic-assisted breeding and valuable loci to elevate rubber yield. Plant Biotechnol J. 2023;21:1058-72. https://doi.org/10.1111/pbi.14018.

7. Makita Y, Ng KK, Veera Singham G, Kawashima M, Hirakawa H, Sato S, et al. Large-scale collection of full-length cDNA and transcriptome analysis in *Hevea brasiliensis*. DNA Res. 2017;24:159-67. https://doi.org/10.1093/dnares/dsw056.

8. Schultes RE. A new infrageneric classification of *Hevea*. Botanical Museum Leaflets, Harvard University. 1977;25:243-57.

9. Mueller-Argoviensis J. Euphorbiaceae. De Candolle, Prodr. Syst Nat Reg Veg. 1862;15:716-9.

10. Huber J. Observações sôbre as arvores de borracha da região amazônica. Bol Mus Paraense3. 1902:345-69.

11. Seibert RJ. A study of *Hevea* (with its economic aspects) in the Republic of Peru. Ann Mo Bot Gard. 1947;34:261-353.

12. Priyadarshan PM. Genetic resources. Biology of *Hevea* rubber. Springer Cham; 2017. p. 83-105.

13. Wycherley PR. CHAPTER 3 - The Genus *Hevea* - Botanical Aspects. In: Sethuraj MR and Mathew NM, editors. Developments in Crop Science. Elsevier; 1992. p. 50-66.

14. Schultes RE. The history of taxonomic studies in *Hevea*. Bot Rev. 1970;36:197-276. https://doi.org/10.1007/BF02858879.

15. Hughes CS, Moggridge S, Müller T, Sorensen PH, Morin GB and Krijgsveld J. Single-pot, solid-phase-enhanced sample preparation for proteomics experiments. Nat Protoc. 2019;14:68-85. https://doi.org/10.1038/s41596-018-0082-x.

16. Balabaskaran S and Muniandy N. Glutathione s-transferase from *Hevea brasiliensis*. Phytochemistry. 1984;23:251-6. https://doi.org/https://doi.org/10.1016/S0031-9422(00)80312-X.

17. Zhang Y, Leclercq J and Montoro P. Reactive oxygen species in *Hevea brasiliensis* latex and relevance to Tapping Panel Dryness. Tree Physiol. 2017;37:261-9. https://doi.org/10.1093/treephys/tpw106.

18. Sussman GL, Beezhold DH and Kurup VP. Allergens and natural rubber proteins. J Allergy Clin Immunol. 2002;110:S33-9. https://doi.org/10.1067/mai.2002.124969.

19. Berthelot K, Lecomte S, Estevez Y and Peruch F. *Hevea brasiliensis* REF (Hev b 1) and SRPP (Hev b 3): An overview on rubber particle proteins. Biochimie. 2014;106:1-9. https://doi.org/10.1016/j.biochi.2014.07.002.

20. Chao J, Yang S, Chen Y and Tian WM. Transcript profiling of *Hevea brasiliensis* during latex flow. Front Plant Sci. 2017;8:1904. https://doi.org/10.3389/fpls.2017.01904.

21. Wang X, Shi M, Wang D, Chen Y, Cai F, Zhang S, et al. Comparative proteomics of primary and secondary lutoids reveals that chitinase and glucanase play a crucial combined role in rubber particle aggregation in *Hevea brasiliensis*. J Proteome Res. 2013;12:5146-59. https://doi.org/10.1021/pr400378c.

22. Peng H, Wang H, Kong W, Li J and Goh WWB. Optimizing differential expression analysis for proteomics data via high-performing rules and ensemble inference. Nat Commun. 2024;15:3922. https://doi.org/10.1038/s41467-024-47899-w.

23. Liu Y, Dang P, Liu L and He C. Cold acclimation by the CBF-*COR* pathway in a changing climate: Lessons from *Arabidopsis thaliana*. Plant Cell Rep. 2019;38:511-9. https://doi.org/10.1007/s00299-019-02376-3.

24. Liu Q, Kasuga M, Sakuma Y, Abe H, Miura S, Yamaguchi-Shinozaki K, et al. Two transcription factors, DREB1 and DREB2, with an EREBP/AP2 DNA binding domain separate two cellular signal transduction pathways in drought- and low-temperature-responsive gene expression, respectively, in *Arabidopsis*. Plant Cell. 1998;10:1391-406. https://doi.org/10.1105/tpc.10.8.1391.

25. Stockinger EJ, Gilmour SJ and Thomashow MF. *Arabidopsis thaliana* CBF1 encodes an AP2 domain-containing transcriptional activator that binds to the C-repeat/DRE, a cis-acting DNA regulatory element that stimulates transcription in response to low temperature and water deficit. Proc Natl Acad Sci U S A. 1997;94:1035-40. https://doi.org/10.1073/pnas.94.3.1035.

26. Shi Y, Ding Y and Yang S. Molecular regulation of CBF signaling in cold acclimation. Trends Plant Sci. 2018;23:623-37. https://doi.org/10.1016/j.tplants.2018.04.002.

27. Yuan HM, Sheng Y, Chen WJ, Lu YQ, Tang X, Ou-Yang M, et al. Overexpression of *Hevea brasiliensis* HbICE1 enhances cold tolerance in *Arabidopsis*. Front Plant Sci. 2017;8:1462. https://doi.org/10.3389/fpls.2017.01462.

28. Cheng H, Chen X and Huang H. The identification and expression analysis of cold responsive HbCOR47 gene from *Hevea brasiliensis*. J Chin J Trop Crops. 2016;37:1924-30.
