# Supplementary material for: Comparative genomics and multiomics analyses reveal the evolution and physiological basis of rubber biosynthesis in Hevea species
Source: Gigascience. 2025 Oct 10;14:giaf115. doi: 10.1093/gigascience/giaf115 (PMC12512020; doi:10.1093/gigascience/giaf115)
Supplement: giaf115_GIGA-D-25-00195_Original_Submission [file giaf115_giga-d-25-00195_original_submission.pdf]

GigaScience

Comparative genomics and multi-omics analyses reveal the evolution and physiological basis of rubber biosynthesis in Hevea species

--Manuscript Draft--

**Manuscript Number:**	GIGA-D-25-00195
**Full Title:**	Comparative genomics and multi-omics analyses reveal the evolution and physiological basis of rubber biosynthesis in Hevea species
**Article Type:**	Research
**Funding Information:**	Science and Technology Research Partnership for Sustainable Development — Prof. Minami Matsui
**Abstract:**	There are multiple species within the Hevea genus, each exhibiting distinct characteristics, but many remain underexplored due to their lower latex productivity. While Hevea brasiliensis is the primary source of natural rubber, other Hevea species represent valuable gene pools that could be leveraged in breeding programs to enhance latex yield, biosynthesis efficiency, and the physicochemical properties of latex. With increasing interest in enhancing natural rubber traits, these lesser-known species are being revisited for their underexplored genetic diversity. In this study, we performed a pangene analysis of six Hevea species and varieties, integrating proteomic and lipidomic data to investigate genetic and metabolic variation related to rubber biosynthesis and latex composition. The pangene analysis revealed conserved and expanded ATP-related functions, underscoring ATP's role in latex production. The proteomic data identified key enzymes involved in rubber biosynthesis and differentially abundant proteins related to latex regeneration, suggesting that regeneration capacity may influence yield efficiency. Lipidomic profiling uncovered species-specific lipid compositions associated with membrane dynamics and rubber particle stability, which may contribute to latex properties. These findings provide valuable insights into Hevea's genomic and metabolic diversity, supporting future breeding programs aimed at improving natural rubber production and its performance in various applications.
**Corresponding Author:**	Minami Matsui<br>RIKEN Center for Sustainable Resource Science<br>JAPAN
**Corresponding Author Secondary Information:**	
**Corresponding Author's Institution:**	RIKEN Center for Sustainable Resource Science
**Corresponding Author's Secondary Institution:**	
**First Author:**	Nyok Sean Lau
**First Author Secondary Information:**	
**Order of Authors:**	Nyok Sean Lau<br>Emiko Ohkubo-Kurihara<br>Yuko Makita Nakai<br>Fetrina Oktavia<br>Tomoko Kuriyama<br>Yukio Kurihara<br>Hidefumi Hamasaki<br>Yuki Nakamura<br>Mitsutaka Kadota<br>Osamu Nishimura

Powered by Editorial Manager® and ProduXion Manager® from Aries Systems Corporation

	Shigehiro Kuraku
	Ahmad Sofiman Othman
	Minami Matsui
**Order of Authors Secondary Information:**	
**Additional Information:**	
**Question**	**Response**
Are you submitting this manuscript to a special series or article collection?	No
**Experimental design and statistics**<br><br>Full details of the experimental design and statistical methods used should be given in the Methods section, as detailed in our Minimum Standards Reporting Checklist. Information essential to interpreting the data presented should be made available in the figure legends.<br><br>Have you included all the information requested in your manuscript?	Yes
**Resources**<br><br>A description of all resources used, including antibodies, cell lines, animals and software tools, with enough information to allow them to be uniquely identified, should be included in the Methods section. Authors are strongly encouraged to cite Research Resource Identifiers (RRIDs) for antibodies, model organisms and tools, where possible.<br><br>Have you included the information requested as detailed in our Minimum Standards Reporting Checklist?	Yes
**Availability of data and materials**<br><br>All datasets and code on which the conclusions of the paper rely must be either included in your submission or deposited in publicly available repositories (where available and ethically	Yes

appropriate), referencing such data using a unique identifier in the references and in the “Availability of Data and Materials” section of your manuscript.<br><br>Have you have met the above requirement as detailed in our Minimum Standards Reporting Checklist?	
GigaScience has policies and guidelines in place for the use of generative AI-writing tools such as ChatGPT. If you have used such writing tools to assist with writing the manuscript this must be declared and cited in the text. Authors should not list AI-writing tools and other AI-assisted technologies as an author or co-author and should acknowledge that they are fully responsible for text generated or refined by AI-writing tools.<p><br><br>A summary of use (particularly in the introduction or among methods) needs to be included at the end of the paper, and the outputs should also be included as a supplementary file hosted in GigaDB or other open repositories. Please read our guidelines for more information. <p><br><br>By submitting to GigaScience, you are aware of the journal's AI-writing tools policy, and if you have declared use of such tools below, you have acknowledged this where appropriate in your manuscript and have made a summary of use and outputs available. <p><br><b>AI-assisted writing tools have been used in the preparation of this manuscript?	No

Click here to access/download;Manuscript;Manuscript_Comparative

Comparative genomics and multi-omics analyses reveal the evolution and physiological basis of rubber biosynthesis in *Hevea* species

Nyok-Sean Lau[1,2†], Emiko Okubo-Kurihara[1,3†], Yuko Makita Nakai[1,4†], Fetrina Oktavia[5], Tomoko Kuriyama[1], Yukio Kurihara[1], Hidefumi Hamasaki[1], Yuki Nakamura[6], Mitsutaka Kadota[7], Osamu Nishimura[7], Shigehiro Kuraku[7], Ahmad Sofiman Othman[2,8], Minami Matsui[1,9*]

[1]RIKEN Center for Sustainable Resource Science, Yokohama 230-0045, Japan

[2]Centre for Chemical Biology, Universiti Sains Malaysia, Bayan Lepas, 11900, Penang, Malaysia

[3]Department of Biology, Keio University, Yokohama 223-8522, Japan

[4]Mebashi Institute of Technology, 460-1, Kamisadori, Maebashi, Gunma, Japan

[5]Indonesian Rubber Research Institute, 15 Jln. Raya Palembang Pangkalan Balai Km. 29, Sembawa, Banyuasin 30953 Indonesia

[6]Plant Lipid Research Team, RIKEN Center for Sustainable Resource Science, Yokohama 230-0045, Japan

[7]Laboratory for Phyloinformatics, RIKEN Center for Biosystems Dynamics Research (BDR), Kobe 650-0047, Japan

[8]School of Biological Sciences, Universiti Sains Malaysia, Minden, 11800, Penang, Malaysia

[9]Yokohama City University, Kihara Institute for Biological Research, Totsuka, Yokohama, Kanagawa, Japan

[*]Correspondence address. Minami Matsui, RIKEN Center for Sustainable Resource Science, Yokohama 230-0045, Japan. Email: minami@riken.jp

[†]These authors contributed equally.

Abstract

There are multiple species within the *Hevea* genus, each exhibiting distinct characteristics, but many remain underexplored due to their lower latex productivity. While *Hevea brasiliensis* is the primary source of natural rubber, other *Hevea* species represent valuable gene pools that could be leveraged in breeding programs to enhance latex yield, biosynthesis efficiency, and the physicochemical properties of latex. With increasing interest in enhancing natural rubber traits, these lesser-known species are being revisited for their underexplored genetic diversity. In this study, we performed a pangene analysis of six *Hevea* species and varieties, integrating proteomic and lipidomic data to investigate genetic and metabolic variation related to rubber biosynthesis and latex composition. The pangene analysis revealed conserved and expanded ATP-related functions, underscoring ATP's role in latex production. The proteomic data identified key enzymes involved in rubber biosynthesis and differentially abundant proteins related to latex regeneration, suggesting that regeneration capacity may influence yield efficiency. Lipidomic profiling uncovered species-specific lipid compositions associated with membrane dynamics and rubber particle stability, which may contribute to latex properties. These findings provide valuable insights into *Hevea*'s genomic and metabolic diversity, supporting future breeding programs aimed at improving natural rubber production and its performance in various applications.

Keywords: *Hevea* species, comparative genomics, proteomics, lipidomics, rubber biosynthesis, latex metabolism

Introduction

Natural rubber stands as an indispensable natural polymer, integral to the manufacture of diverse industrial products, owing to its unparalleled physical properties. Despite extensive research, no synthetic polymer has yet been developed that can replicate the physical attributes of natural rubber. Although more than 2500 plant species are known to biosynthesize natural rubber [1, 2], commercial natural rubber production depends exclusively on latex derived from the Pará rubber tree, *Hevea brasiliensis* Muell. Arg. The genus *Hevea*, belonging to the family Euphorbiaceae, comprises eleven species including *H. brasiliensis, Hevea bethamiana, Hevea camporum, Hevea camargoana, Hevea guianensis*, *Hevea microphylla*, *Hevea nitida*, *Hevea pauciflora*, *Hevea paludosa*, *Hevea rigidifolia* and *Hevea spruceana* [3, 4]. Although the genus is diverse, *H. brasiliensis* is the primary source of natural rubber, accounting for over 90% of global production, estimated at 14 million tons annually (http://www.fao.org/faostat/). Originally from the Amazon basin, *H. brasiliensis* has seen its cultivation shift mainly to Southeast Asia since Henry Wickham introduced rubber tree seeds to the region in 1876 [5]. As a result, global rubber production is now largely dependent on a limited genetic pool from a small collection of seeds. Over the past decades, global rubber production has increased due to the expansion of cultivated areas and a significant increase in productivity, with genetic improvement playing a crucial role.

Natural rubber, a *cis*-1,4-polyisoprene polymer, is synthesised and stored in specialised phloem cells known as laticifers, specifically on the surfaces of rubber particles [6]. The elongation of rubber chains is catalysed by rubber transferase, a member of the cis-prenyltransferase (CPT) family, through the sequential addition of isopentenyl pyrophosphate (IPP) to prenyl groups [7]. A CPT-like (CPTL) protein is thought to be associated with the rubber transferase complex, where it interacts with and activates CPT [8, 9]. In plants, IPP is synthesised via the cytosolic mevalonate (MVA) and plastidic methylerythritol (MEP)

pathways, with evidence suggesting the MVA pathway as the primary source for natural rubber production in *H. brasiliensis* [10]. The synthesis of natural rubber is also supported by rubber elongation factors (REFs) and small rubber particle proteins (SRPPs), the two most abundant proteins on rubber particles, which are essential for rubber particle stability and rubber biosynthesis [11, 12]. Latex coagulation, a crucial process for rubber production, is mediated by lutoid-derived proteins such as hevamine/chitinase, β-1,3-glucanase and hevein, which are released upon lutoid disruption during tapping and promote rubber particle aggregation [13]. Latex biosynthesis is regulated by hormonal signalling pathways, particularly ethylene and jasmonates, which can be produced in response to wounding or applied exogenously. Jasmonates are known to play a role in secondary laticifer differentiation, while ethylene has been shown to prolong the duration of latex flow [14, 15]. Understanding the genetic basis of the rubber biosynthetic pathway is integral to optimising rubber yield and improving production efforts.

Although multiple genome assemblies have been published for *H. brasiliensis*, genomic analyses of other *Hevea* species have only recently become available [16-23]. Genetic information beyond *H. brasiliensis* remains limited, restricting the gene space accessible for comparative studies and breeding. To help address this gap and improve our understanding of diversity within the genus, we performed a pangene analysis of five *Hevea* species or varieties alongside the *H. brasiliensis* reference genome. Our comparative genomic analyses, integrated with proteomics and lipidomics data, enabled the exploration of key agronomic traits, including core components of rubber biosynthesis, factors influencing its capacity, and lipid composition relevant to latex properties. This multi-omics approach offers a broader view of molecular variation related to rubber biosynthesis and latex traits in *Hevea*, providing a useful resource to inform future efforts in genetic improvement and species conservation.

Results

Hevea species genome sequences as a resource for diversity studies

We previously reported a draft genome sequence of *H. brasiliensis* RRIM 600, a historically important clone widely planted during the early expansion of rubber cultivation in East Asia [22] (Supplementary Text S1). To support comparative analyses, RRIM600 was selected for high-quality genome reconstruction. A chromosome-level genome was constructed using a hybrid approach integrating PacBio SMRT and Hi-C sequencing (Supplementary Table S1). The initial PacBio assembly, based on 169.94 Gb of reads, yielded a 1.71 Gb genome with an N50 of 550.35 Kb (Supplementary Table S2). Refinement with Hi-C reads (105-fold coverage) produced a final assembly of 1.71 Gb, with a scaffold N50 of 78.35 Mb (Fig. 1a, Supplementary Text S2 and Table S3). More than 78% of the assembly was anchored onto 18 pseudo-chromosomes, covering 91% of the estimated 1.88 Gb genome size (Fig. 1b, Supplementary Fig. S1-2 and Table S4). Benchmarking Universal Single-Copy Orthologs (BUSCO) assessment showed 98.7% completeness, and over 90% of PacBio Iso-Seq transcripts aligned to the assembly, indicating high contiguity and sequence quality (Supplementary Text S3, Fig. S3 and Table S5-6). Centromeric regions were identified in all 18 chromosomes, while telomeric sequences were detected at both ends of most chromosomes, with only one telomeric end identified in four chromosomes (Fig. 1c, Supplementary Table S7-S8). To explore genomic diversity in *Hevea*, we sequenced five additional *Hevea*: *H. guianensis*, *H. pauciflora*, *H. spruceana*, *H. confusa* (*H. pauciflora* var. *confusa*) and *H. collina* (*H. guianensis* var. *collina*) (Supplementary Text S4). Their genomes were *de novo* assembled using 56.9 to 95.6× Illumina reads and synteny scaffolded with *H. brasiliensis* as reference. Assembly sizes ranged from 1.35 to 1.77 Gb, with N50 values of 0.29 to 38.51 Mb and GC content between 33.80 and 34.48% (Supplementary Fig. S4).

Annotation using the MAKER pipeline predicted an average of ~65,000 high-confidence gene models, with *H. brasiliensis* containing the most (84,076) and *H. guianensis* the fewest (44,254). Predicted protein-coding genes were mapped to functional databases, including NCBI NR, SwissProt, TrEMBL, InterPro, Gene Ontology (GO), and Kyoto Encyclopedia of Genes and Genomes (KEGG) (Supplementary Table S9). The gene models averaged 3,044 to 3,874 bp in transcript length, 599 to 798 bp in coding sequence length, and four exons per gene (Supplementary Fig. S5). Noncoding RNA genes, including tRNAs, rRNAs, miRNAs, and snoRNAs, were also identified (Supplementary Table S10). Repeat annotation using *de novo* and homology-based approaches revealed variability across *Hevea* genomes. *H. brasiliensis* exhibited the highest repeat content (74.1%), while *H. collina* had the lowest (33.3%) (Supplementary Table S11). Long terminal repeats (LTRs) dominated, constituting 31.21-70.54% of the genomes, with Gypsy elements being particularly abundant (31.45-54.92%). In contrast, long interspersed nuclear elements (LINEs) accounted for only 1.04-1.60%, and DNA transposons ranged from 0.79-1.94%. The Gypsy-to-Copia ratio was consistently high across species. Divergence analysis of transposable elements based on Kimura distances revealed a peak divergence rate of around 6 for *H. pauciflora* and 9-10 for other species, with the Gypsy superfamily being the major component of the peak (Supplementary Fig. S6).

Comparative genomics of *Hevea* species

To study the genomic landscape of the *Hevea* genus, we conducted comparative analyses on the assembled genomes of *H. brasiliensis*, *H. guianensis*, *H. pauciflora*, *H. spruceana*, *H. collina*, and *H. confusa* (Fig. 2a-c). Orthologous gene analysis classified all genes from these six genomes into 239,660 families. Our findings revealed that 2% of the gene families were present in the core-cluster, 4% in the soft-core, 7% in the shell and 87% in the cloud clusters. The core and soft-core clusters harboured highly conserved genes shared across all analysed

genomes, while the shell and cloud clusters contained flexible genes that may serve as reservoirs of genetic variability contributing to adaptive potential. GO enrichment analysis of the core and soft-core gene clusters identified significantly enriched GO terms, with the largest gene members included in ATP binding, ATP hydrolysis activity, and apoplast (Fig. 2d-e, Supplementary Table S12-13). In the shell gene cluster, enriched pathways also encompassed ATP binding, and ATP hydrolysis activity, but also plasmodesma (Fig. 2f, Supplementary Table S14). Functional annotation of the cloud genes revealed significant enrichment of GO terms associated with DNA integration, U5 snRNA binding, and U6 snRNA binding (Fig. 2g, Supplementary table S15).

Modelling of pangene size showed that the total number of gene families increased with the addition of each new genome, indicating that *Hevea* genomes exhibit genetic variation, and the analysed genomes may not fully capture the diversity of the genus. Furthermore, species-specific genes in *H. brasiliensis* were annotated, revealing an abundance of pathways related to binding, cellular anatomical structures, and cellular processes (Fig. 2h). These fundamental function genes have likely evolved independently to support the distinct biological characteristics of *H. brasiliensis*.

To trace the evolutionary history of *Hevea*, gene family clustering was performed using gene sets from 13 plant species, including six *Hevea*, six representative Malpighiales species, and the outgroup *Arabidopsis thaliana*. This analysis identified 1,156 single-copy orthologs shared between *Hevea* and the other plants, which were used for phylogenetic reconstruction and divergence time estimation (Fig. 3a). Our results indicate that all *Hevea* species share a common ancestor around 6.46 million years ago (mya), with *Hevea* diverging from *Manihot esculenta* approximately 26.54 mya. *H. brasiliensis* exhibited high conservation of orthologous genes with *M. esculenta*, *Ricinus communis*, and *Jatropha curcas*, particularly with *M. esculenta* (Fig. 1b). The phylogenomic analysis revealed two

distinct clades within *Hevea*: one containing *H. brasiliensis, H. spruceana, H. pauciflora,* and its variety *H. confusa*; the other comprising *H. guianensis* and its variety *H. collina*.

To investigate changes in gene family size during evolution, we analysed expansions and contractions of gene families in *Hevea* relative to the most recent common ancestor of the 13 plant species, as well as in *H. brasiliensis* relative to the common ancestor of *Hevea* (Fig. 3a). Expanded gene families in *Hevea* species were enriched for functions related to ATP binding, plasmodesma, and ATP hydrolysis activity (Fig. 3b, Supplementary table S16). In *H. brasiliensis,* expanded gene families also showed enrichment in ATP binding, plasmodesma, and zinc ion binding (Fig. 3c, Supplementary table S17). Conversely, contracted gene families in *Hevea* species exhibited an overrepresentation of functions associated with the negative regulation of macromolecule metabolic process, defence response to fungus, and ubiquitin protein ligase activity (Supplementary table S18). In *H. brasiliensis,* contracted gene families were overrepresented with gene expression, RNA metabolic process, and the regulation of cellular metabolic process (Supplementary table S19).

For dating the whole-genome duplication event in *Hevea*, a genomic analysis based on self-comparison of *H. brasiliensis, H. guianensis, H. pauciflora, H. spruceana, H. collina* and *H. confusa* paralogous genes was carried out. All *Hevea* genomes exhibited a major left peak in the distributions of synonymous substitutions per synonymous site (K_s) and transversions at four-fold degenerate sites (4DTv) frequency, indicating a recent species-specific duplication (Fig. 3d-e). K_s estimates for paralogs in *H. brasiliensis, H. guianensis, H. pauciflora, H. spruceana, H. collina,* and *H. confusa* suggested that this *Hevea*-specific duplication event may date back to ~12.3-28.7 mya. Additionally, a minor right peak in the 4DTv distribution and a right smooth peak in the K_s distribution correspond to the whole-genome triplication event across core eudicots.

Evolution of rubber biosynthesis potential in *Hevea* species

The biosynthesis of natural rubber in *Hevea* involves a network of metabolic pathways and key proteins. Genes linked to rubber biosynthesis were identified through genome annotation across *Hevea* species, and their activity in latex was confirmed by proteomics (Fig. 4-5, Supplementary Text S5). The relative abundances of proteins in latex were quantified using data-independent acquisition coupled with high-resolution mass spectrometry (Supplementary Text S6 and Table S20).

IPP monomer supplying pathways

At the core of this process are the pathways that supply IPP, the fundamental building block for natural rubber**.** IPP is produced via two compartmentalised metabolic routes: the MVA pathway in the cytosol and the MEP pathway in the plastids. In *Hevea*, 14–18 genes are annotated in the MVA pathway, while 14–22 are associated with the MEP pathway (Supplementary Table S21). Proteomic analysis identified 14 of the 18 MVA pathway proteins in latex but only seven from the MEP pathway (Fig. 5a, Supplementary table S22). Notably, MEP pathway proteins were detected at much lower levels, and 1-deoxy-D- xylulose 5-phosphate synthase (DXS), which catalyses the first MEP pathway step, was absent from the latex proteome. In the MVA pathway, all six key enzymes, acetyl-CoA acetyltransferase (AACT), 3-hydroxy-3-methylglutaryl-coenzyme A synthase (HMGS), HMG-CoA reductase (HMGR), mevalonate kinase (MVK), phosphomevalonate kinase (PMVK), and mevalonate diphosphate decarboxylase (DPMD), were detected in latex, represented by two to four isoforms. AACT3, HMGS1, and DPMD were among the most abundant isoforms.

During rubber elongation, IPP and its isomer, dimethylallyl diphosphate, undergo sequential condensations to form geranyl pyrophosphate (GPP), farnesyl pyrophosphate (FPP), and geranylgeranyl pyrophosphate (GGPP), catalysed by trans-prenyltransferases such as geranyl pyrophosphate synthase (GPS), farnesyl pyrophosphate synthase (FPS), and

geranylgeranyl pyrophosphate synthase (GGPS). These short-chain prenyl pyrophosphates serve as initiators for further IPP condensation, leading to the production of high molecular weight natural rubber. Among these enzymes, FPS was the most abundant in latex, indicating its role as the major trans-prenyltransferase in *Hevea*, as its product, FPP, has been identified as the main initiator of natural rubber biosynthesis [24].

Rubber particle−associated proteins

The key enzymes responsible for natural rubber biosynthesis, CPTs, were annotated in *Hevea* genomes: CPT1-8 in *H. brasiliensis*, CPT1-5 in *H. guianensis*, CPT1-7 in *H. pauciflora*, CPT1-5 in *H. spruceana*, CPT1-5 in *H. collina*, and CPT1-8 in *H. confusa* (Figure 4a, Supplementary Table S23). Chromosome 18 emerged as a hotspot for CPT genes, with several clustered together, suggesting coordinated regulation, supported by the observation of topologically associating domain structures for CPT1-4 in the *H. brasiliensis* leaf Hi-C analysis (Fig. 4b. Supplementary Fig. S7). Comparative analyses of CPT orthologs across multiple species, including *Linum usitatissimum*, *P. trichocarpa*, and *Salix purpurea* from Malpighiales, and *J. curcas, M. esculenta*, *R. communis*, and *Hevea* species from Euphorbiaceae, revealed a higher CPT gene count in Euphorbiaceae (average of seven) compared to non-Euphorbiaceae Malpighiales (average of four), indicating a lineage-specific duplication event in Euphorbiaceae (Supplementary Table S24).

Phylogenetic analysis, including rubber-producing species such as *Taraxacum koksaghyz*, *Taraxacum brevicorniculatum*, and *Parthenium argentatum*, revealed two CPT clades: clade I comprising chloroplastic CPTs, and clade II consisting of cytosolic CPTs, consistent with previous studies (Fig. 5a) [8]. Within clade II, specific *Hevea* CPTs formed subclades distinct from other rubber-producing plants. Proteomic analysis of *Hevea* latex showed that CPTs1-3 from clade II were exclusively expressed in latex, reinforcing their role

in rubber biosynthesis. Multiple alignment of CPT sequences identified conserved regions I-V characteristic of known CPTs structure in *Hevea* [25] (Supplementary Fig. S8).

The rubber transferase complex, comprising CPTs and CPT-like (CPTL) proteins, has been characterised in *Hevea* and other rubber-producing species, where CPTLs anchor, activate, and stabilise CPTs on rubber particles without directly catalysing polymerisation [8, 9, 26, 27]. Genome annotation and proteomic detection confirm CPTLs are present across *Hevea* species beyond *H. brasiliensis*, supporting their involvement in rubber biosynthesis. Congruent with earlier observations, *Hevea* CPTLs exhibit low homology to CPTs, lacking conserved CPT motifs [26] (Supplementary Fig. S9). Phylogenetic analysis showed *Hevea* CPTLs forming a distinct cluster from those in *P. argentatum, T. brevicorniculatum, L. sativa, S. lycopersicum,* and *A. thaliana* (Fig. 4c).

REF and SRPP proteins, which are abundant in rubber particles, contribute positively to rubber biosynthesis by stabilising particle structure and preventing coagulation, with REF levels in latex correlating with rubber content [9, 11, 12, 28]. Genome annotation revealed an average of 12 REF/SRPP genes across *Hevea*, with *H. brasiliensis* containing the most (16) and *H. collina* the fewest (7) (Supplementary Table S25). Non-*Hevea* species in Malpighiales and Euphorbiaceae have an average of four REF/SRPP genes. Phylogenetic analysis delineated REF/SRPP genes into five evolutionary branches, with distinct clusters for *Hevea* genes, particularly in branch I, III, IV, and V (Fig. 4d). Sequence alignments confirmed the presence of the REF domain and noted C-terminal differences between SRPP and REF, consistent with previous reports [12, 28] (Supplementary Fig. S10). Proteomic analysis identified *Hevea* REF1-3 and SRPP1-4 in latex, with REF2 and SRPP1 being the most abundant among latex biosynthesis-related proteins.

Rubber particle aggregation

In addition to rubber particles, lutoids of vacuolar origin are major components of latex, containing proteins essential for latex coagulation. Upon tapping, latex flow alters turgor pressure, causing lutoid rupture and the release of hevamine/chitinase and β-1,3-glucanase—proteins that promote rubber particle aggregation and latex coagulation [13, 29]. Concurrently, hevein contributes to particle aggregation, ultimately plugging latex vessels and stopping flow. Our study demonstrates a high abundance of chitinase and β-1,3-glucanase in *Hevea* latex proteomes, consistent with their proposed role in rubber coagulation (Supplementary Table S26).

Jasmonate and ethylene signalling in rubber biosynthesis

Tapping induces a wounding response in phloem tissues that activates the jasmonate and ethylene signalling pathways. Jasmonate signalling is crucial for laticifer differentiation and stimulates the expression of rubber biosynthesis-related genes (e.g., *FPS* and *SRPP*), leading to increased rubber yield [14]. The coronatine-insensitive 1 (COI1)–jasmonate ZIM-domain (JAZ)–MYC transcription factor module is central to this pathway: jasmonate perception leads to the degradation of JAZ repressors via the COI1 complex, releasing MYC2 and activating jasmonate-responsive genes [30]. Components of the COI1–JAZ–MYC2 regulatory module were annotated across *Hevea* species (Fig. 5a). Latex proteomes showed high levels of COI1 and absence of JAZ, suggesting JAZ degradation and activation of jasmonate-responsive genes.

Ethylene treatment prolongs latex flow by promoting sucrose allocation, water transport, glycolysis, and C3 carbon fixation [15]. While key ethylene signalling components, ethylene receptors, constitutive triple response 1 (CTR1), and ethylene-insensitive 2/3, are annotated in *Hevea* genomes, their low abundance in latex proteomes (Fig. 5a) suggests that ethylene signalling is not the primary response to tapping-induced wounding.

Differential proteomics reveal potential factors influencing high-capacity rubber biosynthesis

We next identified proteins differentially expressed between *H. brasiliensis* and other *Hevea* species latex (Fig. 5b, Supplementary Fig. S11 and Table S27). Notably, glutamine synthetase exhibited the largest fold change, increasing 24.51-fold in *H. brasiliensis* compared with other species. This observation aligns with investigations into ethylene treatment on latex yield, which suggest that the glutamine synthetase/glutamate synthase cycle may constitute the major pathway for protein synthesis involved in latex regeneration [31]. Metallothionein, enriched 18.65-fold in *H. brasiliensis*, was also among the top differentially expressed proteins. While thiol, ascorbate, and glutathione are established as the major ROS scavengers in latex [32], the marked upregulation of metallothionein highlights its likely complementary role in maintaining ROS homeostasis under oxidative stress during tapping and regeneration [33]. Additionally, the bark storage protein, a nitrogen storage protein not previously linked to latex production, was upregulated by 18.34-fold. In *J. curcas*, it has been implicated in nitrogen cycling [34], and in *H. brasiliensis*, it may contribute to meeting the nitrogen demands associated with latex regeneration. Interestingly, a eukaryotic translation initiation factor (eIF), fundamental for protein synthesis initiation, was enriched 17.80-fold in *H. brasiliensis.* Within the eIF family, eIF5A, also known as rubber biosynthesis stimulator protein, promotes IPP incorporation into rubber during *in vitro* assays, revealing its potential in enhancing rubber biosynthesis and yield trait [35]. Furthermore, proteins linked to rubber biosynthesis, including those involved in IPP formation, rubber biosynthesis, rubber particle aggregation, and hormone signalling, were analysed for differential expression (Supplementary Table S28). However, only SRPP2 (2.19-fold), MVK2 (6.22-fold), AACT4 (10.74-fold), and CTR1a (12.4-fold) showed significant enrichment in *H. brasiliensis*.

Lipidome profiling of *Hevea* species latex

In addition to *cis*-polyisoprene, *Hevea* latex comprises proteins, lipids, and carbohydrates, with lipids known to influence natural rubber's physical properties [36-38]. To explore this further, a targeted metabolomic analysis was performed to profile the lipid composition of latex from *Hevea* species within the same age range. The lipidomic profiles were analysed using LC-MS/MS, identifying 53 lipid molecules classified into five main categories: fatty acyls, glycerolipids, glycerophospholipids, sphingolipids, and sterol lipids, which were further divided into 16 classes (Supplementary Table S29-S30). Triacylglycerol (TG), acylhexosyl sitosterol (AHexSIS), and phosphatidic acid (PA) were the most abundant lipid classes across species (Fig. 6a). *H. guianensis* and *H. confusa* exhibited lipid profiles similar to *H. brasiliensis*, characterised by high PA but lower TG. In contrast, *H. pauciflora*, *H. spruceana*, and *H. collina* showed a distinct profile with elevated TG and reduced PA. The lipid composition differed from that reported previously [39], as our samples reflected field-to-factory timelines without chemical preservation.

A supervised orthogonal partial least-squares discriminative analysis (OPLS-DA) was performed to compare the latex lipidomes of *H. brasiliensis* and other *Hevea* species. The OPLS-DA score plots showed clear separation between the two groups, with R^2X(cum), R^2Y(cum), and Q^2(cum) values of 0.80, 0.97, and 0.801, respectively (Fig. 6b). Differential lipid species were identified using OPLS-DA and univariate analysis, selecting those with variable importance in projection (VIP) > 1 and $p < 0.05$. This analysis identified 18 differential lipid molecules, comprising six acylated steryl glucosides (ASG), one ceramide (Cer), two diacylglycerols (DG), five hexosylceramides (HexCer), three PA, and one sterol lipid (ST) (Fig. 6c, Supplementary Table S31). Among these, DG 36:1, DG 36:2, and PA 36:1 exhibited the highest upregulated fold changes, whereas HexCer 41:1, HexCer 43:1, and HexCer 42:1 were the most downregulated in *H. brasiliensis* relative to other *Hevea* species.

Discussion

The genetic diversity among *Hevea* species is crucial for breeding and conservation, offering a reservoir of traits that could be harnessed to improve rubber yield, latex regeneration capacity, and physiological adaptability. However, the limited genetic information available for species other than *H. brasiliensis* poses challenges to fully understanding the evolutionary relationships and functional diversity within the genus. This highlights the importance of identifying shared and species-specific genes across *Hevea* species to better explore and utilise the genetic potential of this economically significant genus.

A pangene analysis of *H. brasiliensis*, *H. guianensis, H. pauciflora, H. spruceana, H. collina,* and *H. confusa* revealed conservation of ATP binding and ATP hydrolysis activity functions within core and soft-core gene clusters. Simultaneously, gene family size evolution analysis showed expansion in ATP binding and ATP hydrolysis activity gene families in *Hevea*. These findings, demonstrating that ATP-related functions are conserved in core genes and expanded relative to other plants, underscore ATP's role in *Hevea* physiology and metabolism. ATP plays a pivotal role in latex production of *H. brasiliensis* by functioning as a physiological regulator directly in the metabolic pathways and indirectly through the H^+-ATPase activity. Efficient adenylate turnover supports latex regeneration and rubber yield by driving sucrose conversion to polyisoprene and regulating cytosolic pH [40]. While inorganic phosphorus is an established indicator in latex diagnosis [41], previous studies have also reported a strong correlation between latex ATP levels and rubber yield [42, 43], suggesting that ATP may contribute to yield-associated physiological processes. The conservation and expansion of ATP metabolism-related genes across *Hevea* species may thus reflect genomic adaptations supporting the metabolic demands of latex production.

The taxonomy of *Hevea* species has long relied on morphological classification, which has not been updated for some time [3, 44]. Morphological approaches are limited by

geographic accessibility and confounded by convergent evolution and phenotypic plasticity. Sampling of native *Hevea* species, aside from *H. brasiliensis*, has historically been confined to the Amazon basin [45]. In this study, genomic resources beyond *H. brasiliensis* provide a more robust framework for delineating taxonomic relationships based on genetic relatedness. Phylogenomic analysis of single-copy orthologs revealed two distinct *Hevea* subgroups: one comprising *H. brasiliensis*, *H. pauciflora*, *H. confusa*, and *H. spruceana*, and the other containing *H. guianensis* and *H. collina*. The divergence between these subgroups, estimated at 6.46 mya, predates the introduction of *H. brasiliensis* into cultivation outside its native range. This divergence may reflect ecological differentiation, as *H. guianensis* typically grows at higher elevations, while the others thrive in lowland areas [45]. Further genomic and ecological data from additional representative species of the genus are needed to substantiate this speculation.

Characterisation of the genetic basis of latex biosynthesis across rubber-producing plants suggests polyphyletic evolution, with convergent mechanisms and enzymes emerging independently in distinct clades [46, 47]. Our phylogenetic analyses of rubber biosynthesis genes in *Hevea* support this, showing *Hevea* CPT genes formed a distinct clade compared to other rubber-producing plants. Similarly, the *Hevea* CPTL and REF/SRPP genes clustered separately in their respective phylogenies. Comparative analyses of CPT and REF/SRPP gene counts indicated that the Euphorbiaceae including *Hevea*, have undergone CPT gene duplication, whereas REF/SRPP gene duplication occurred exclusively in *Hevea*. The *Hevea* CPT genes have expanded into two subclades, with CPT1-3 detected in the latex proteome, clustering within one subclade in the phylogeny. This observation suggests that the two *Hevea* CPT subclades may have diverged through neofunctionalization, with the CPT1-3 likely playing a role in natural rubber metabolism. Similarly, *Hevea* REF/SRPP genes are separated into three subgroups, with REF1-3 and SRPP1-4 exclusively detected in the latex proteome.

This phylogenetic divergence suggests functional specialisation driven by selection, with the latex-specific REF/SRPP genes adapted for rubber particle function.

Proteomic analysis provides evidence for the presence and relative abundance of proteins, offering complementary insights into the potential activity of metabolic pathways. In this study, proteomics of latex across *Hevea* highlighted key enzymes involved in rubber biosynthesis, spanning IPP formation, rubber elongation, particle aggregation, and hormone signalling. The latex proteome confirmed the MVA pathway as the primary IPP source, FPS as the main trans-prenyltransferase, and CPT1-3, REF1-3, and SRPP1-4 as essential for latex synthesis. Hevamine/chitinase and β-1,3-glucanase are implicated in the rubber particle aggregation, while jasmonate signalling is activated in response to tapping. These proteins represent core rubber biosynthesis components across *Hevea* species. To explore the factors contributing to efficient rubber biosynthesis in *H. brasiliensis*, differential proteomics analysis revealed several key enriched proteins. These include glutamine synthetase, which is linked to protein synthesis for latex regeneration [31]; metallothionein, which plays a role in maintaining ROS homeostasis [33]; and a eukaryotic translation initiation factor that is essential for overall protein synthesis [35]. Among the core components linked to rubber biosynthesis, only SRPP2, MVK2, AACT4, and CTR1a showed significant enrichment. Given the continuous harvesting of latex, which entails a high demand for latex regeneration to replenish carbon, nitrogen, and energy reserves, the substantial enrichment of proteins involved in nitrogen metabolism, ROS scavenging, and protein synthesis suggests that the efficiency of rubber biosynthesis in *H. brasiliensis* relies on the capacity for latex regeneration. Notably, only four out of the 51 proteins linked to rubber biosynthesis detected in latex were significantly enriched, indicating that while the biosynthetic machinery may be efficient, the overall process could be constrained by the regeneration of latex components. Our proteomics finding contributes to a deeper understanding of the molecular mechanisms

underlying rubber biosynthesis and highlights the importance of focusing on latex regeneration as a potential strategy for improving latex yield, beyond targeting the biosynthetic pathway itself.

Latex lipidomes further revealed distinct compositional features across *Hevea* species, with TG, AHexSIS, and PA identified as dominant lipid classes. TG functions as a lipid reservoir supporting regeneration [48], while AHexSIS modulates membrane fluidity [49], potentially influencing latex colloidal stability during storage or coagulation. PA, associated with membrane curvature and vesicle formation [50], may influence interparticle interaction during transport and processing. Latex from *H. brasiliensis* exhibited elevated levels of DG 36:1, DG 36:2, and PA 36:1 compared to other *Hevea* species. DG acts as a biosynthetic precursor and signalling molecule [51], and its enrichment suggests more active membrane turnover and stress adaptation. Likewise, higher PA levels may enhance colloidal stability and particle interactions during storage and processing. Since PA is also involved in plant stress signalling, particularly in response to wounding [52], its abundance may reflect an adaptive response to tapping-induced stress. Differences in rubber particle size between species [53] likely contribute to these compositional differences, affecting surface-area-dependent lipid interactions and processing behaviour. Overall, the distinct lipid profile of *H. brasiliensis* suggests adaptive metabolic features that support rubber stability, membrane integrity, and flow resilience—traits crucial for high-quality rubber production.

Our study highlights the genetic diversity within *Hevea* species, providing insights into rubber yield, latex physiology, and potential species-specific adaptation. Pangene analysis revealed conserved and expanded ATP-related functions, underscoring ATP's role in latex regeneration, which supports high rubber yield. Proteomic profiling uncovered differentially abundant proteins, including those regulating nitrogen balance, ROS homeostasis, and protein synthesis, linking latex yield to regeneration potential. Lipidomic

analysis showed species-specific lipid compositions that may influence latex properties, with adaptations in *H. brasiliensis* linked to membrane remodelling and rubber particle stability. While further functional analyses are required to confirm these roles, our integrative multi-omics framework offers valuable genomic and biochemical insights to guide future research and breeding strategies for improved rubber production.

Methods

DNA extraction and genome sequencing

The sequenced individuals of *H. brasiliensis, H. guianensis, H. pauciflora, H. spruceana, H. collina,* and *H. confusa* were maintained at the Rubber Research Institute of Indonesia (Supplementary Text S1). High molecular weight genomic DNA was extracted from young leaves of *H. brasiliensis* using the Genomic-tip 100/G kit (Qiagen, Germany). A PacBio library was prepared using the SMRTbell Express Template Prep Kit 2.0 (Pacific Biosciences, USA) according to the manufacturer’s instructions. Sequencing was performed on PacBio Sequel II with a SMRT Cell 8M. Raw PacBio subreads were obtained from the SMRT Link v9.0 pipeline. For Hi-C sequencing of *H. brasiliensis,* fresh young leaves were ground in liquid nitrogen and fixed with formaldehyde. The Hi-C library was constructed using the Arima Hi-C kit with DpnII and HinfI restriction enzymes, following Kadota et al. [54] (Supplementary Text S2). The library was sequenced on a HiSeq X Ten (Illumina, USA). For short-read sequencing of *H. guianensis, H. pauciflora, H. spruceana, H. collina,* and *H. confusa,* genomic DNA was extracted from leaf tissues using a DNeasy Plant Mini Kit (Qiagen). Sequencing libraries were prepared using the TruSeq DNA Sample Preparation Kit (Illumina) and sequenced on a DNBSEQ platform at BGI-Shenzen (BGI Co. Ltd., China).

RNA sequencing

Total RNA was extracted from the bark, latex, leaf and petiole of *H. brasiliensis* following the method previously published [55], using a CTAB buffer and lithium chloride precipitation. RNA was quantified using a NanoDrop spectrophotometer (ThermoFisher Scientific, USA) and assessed with a Bioanalyzer 2100 (Agilent Technologies, USA). For Iso-seq, cDNA synthesis was performed using the SMARTer PCR cDNA Synthesis Kit (Clontech, Japan). Size fractionation and selection were carried out using the BluePippin Size Selection System (Sage Science, USA). SMRT libraries were prepared using the SMRTbell Express Template Prep Kit 2.0 (Pacific Biosciences) and sequenced on the PacBio Sequel II. Iso-Seq data were analysed using SMRT Link (v9.0), which incorporates read quality filtering, read clustering, consensus calling, and Quiver polishing steps to assemble the Iso-Seq reads into high quality, full-length transcripts (Supplementary Text S3).

Genome assembly and annotation

For *H. brasiliensis*, the long-read assembler Flye [56] (v2.8.2) was used for the assembly and polishing of PacBio reads with the parameter -pacbio-raw. The raw Hi-C reads were adapter and quality trimmed using TrimGalore (v0.6.0). The clean Hi-C reads were mapped to the PacBio assembly using Juicer pipeline [57] (v1.6). Scaffolds were grouped, ordered, and orientated into pseudo-chromosomes using 3d-dna (v.180922) with parameters i=10000, and r=4. For *H. guianensis, H. pauciflora, H. spruceana, H. collina,* and *H. confusa*, Illumina reads were trimmed using Platanus_trim (v1.0.7) and assembled using Platanus (v1.2.4) [58]. The Platanus assemblies were synteny scaffolded with RagTag [59] (v2.1.0) using *H. brasiliensis* as reference.

Genome size was estimated based on *k*-mer counting using Jellyfish (v2.3.0). For *H. brasiliensis*, previously sequenced Illumina paired-end reads [22] were used, while for *H. guianensis*, *H. pauciflora*, *H. spruceana, H. collina*, and *H. confusa*, short reads generated in

this study were analysed. Genome size was estimated based on 25 *k*-mer with GenomeScope 2.0. Completeness of the genome assembly was assessed using BUSCO (v5) [60] against the Embryophyta odb10 database.

Telomeric and centromeric sequences in *H. brasiliensis* chromosomes were analysed using quarTeT (v1.2.5). Within quarTeT, the TeloExplorer module, which utilises the Telomere Identification Toolkit (tidk), was used for telomere detection. Meanwhile, the CentroMiner module incorporated transposable element annotations from the Extensive De Novo TE Annotator (EDTA) as a complementary approach for centromere identification.

Repetitive elements in the *Hevea* species genome were identified by RepeatMasker (v4.1.0) [61] using the *de novo* repeat library and known Viridiplantae repetitive sequences in Dfam and RepBase. The *de novo* repeat library was constructed using RepeatModeler, integrating RECON (v1.0.8), RepeatScout (v1.0.6), and Tandem Repeat Finder (v4.0.9). Genome annotation was performed using the MAKER (v3.01.03) [62] pipeline with *ab initio* gene predictions Augustus (v3.2.2), Snap (v2013-11-29), GeneMark-ES (v3.61), and Fgenesh (v8.0.0b). Iso-Seq dataset and assembled transcripts from previously generated RNA-seq [63] were input to MAKER as expressed sequence tag evidence. The functional annotation of the protein-coding genes against NCBI NR, SwissProt, TrEMBL and KEGG was performed with BLASTP at an *E*-value of $1e^{-5}$. GO terms and InterPro entries were assigned via OmicsBox (v3.1.9) and InterProScan (v5.52-86.0). Non-coding RNAs were annotated using INFERNAL (v1.1.4) against the Rfam database.

Clustering of pangene

Pangene across *Hevea*, including *H. brasiliensis, H. guianensis, H. spruceana, H. collina*, and *H. confusa* were analysed using GET_HOMOLOGUES-EST (v3.6.2) [64]. All-against-all comparisons were performed using BLAST, followed by clustering with the OrthoMCL algorithm at an inflation value of 1.5. Genes were clustered into core, soft-core, shell and

cloud clusters based on orthogroup frequency. GO enrichment analysis for genes in each cluster was performed using the OmicsBox. The curves describing pangene and core gene sizes were fitted to the Tettelin model.

Construction of gene families

We downloaded the protein sequences of *A. thaliana, L. usitatissimum, M. esculenta, O. sativa, P. trichocarpa, R. communis*, and *S. purpurea* from Phytozome 13 (https://phytozome-next.jgi.doe.gov/), and the protein sequences of *J. curcas* and *Vitis vinifera* from Ensembl Plants (https://plants.ensembl.org/). Gene families based on all-against-all BLASTP alignment among the 13 plant species were constructed using OrthoFinder (v2.5.5) [65]. Single-copy orthologs for each species within a given orthogroup as analysed by OrthoFinder were aligned using MAFFT (v7.520), and the resulting alignments were concatenated to create a super alignment matrix. Phylogenetic analysis on the concatenated alignment was conducted using IQ-TREE (v2.2.6) [66] with maximum likelihood and 1000 ultrafast bootstraps, employing the JTTF+I+R6 model selected by ModelFinder according to the Bayesian Information Criterion. The phylogenetic tree was visualised using FigTree (v1.4.5). The divergence times in the phylogenetic tree were inferred using the least square dating method implemented in IQ-TREE. For divergence time estimation, we calibrated the model using the divergence times between *A. thaliana* and *H. brasiliensis* (108 Mya), *L. usitatissimum* and *H. brasiliensis* (88 Mya), and *P. trichocarpa* and *H. brasiliensis* (72 Mya) obtained from the TimeTree database.

Gene family size evolution

The gene family expansion and contraction across 13 plant species were inferred using CAFE5 [67], with the phylogenetic tree constructed by IQ-TREE based on single-copy orthologs as the input tree. The separate birth and death rates of gene families across the

phylogeny were estimated using maximum likelihood, and the *P*-value threshold of 0.01 was applied to identify significantly expanded or contracted gene families for a given species. These expanded and contracted gene families were subjected to enrichment analysis using OmicsBox with Fisher's exact test.

Genome duplication analysis

The genome-wide duplications in the *Hevea* species genomes were analysed with Tree2GD (v1.0.40) [68]. All-against-all protein alignments were performed with Diamond (v2.1.9), and hierarchical orthogroups were predicted with PhyloMCL (v2.0). Gene trees were reconciled with a reference species tree using the default parameters in Tree2GD. Paralogous gene pairs for each species were identified, and their coding sequences were aligned using MUSCLE (v3.8.31) and pal2nal (v13). The K_s values of the gene pairs were calculated using KaKs_calculator (v2.0), and the 4DTv values were determined using the Perl script Calculate_4DTV_correction.pl. The genome duplication events times of the *Hevea* species were estimated using the formula $T = K_s/2r$, where the rate of synonymous substitutions per site per year (r) is 7.5×10^{-9}.

Proteome characterisation

Proteins from the latex samples were extracted as detailed in Note S5. The peptides were injected onto a 75 µm × 120 mm nanoLC column (Nikkyo Technos) at 50 °C and then separated with a gradient (A = 0.1% formic acid (FA) in water, B = 0.1% FA in 80% acetonitrile) consisting of 0-50 min 8% B, 50-57 min 36% B, 57-60 min 70% B using an UltiMate 3000 RSLCnano LC system (Thermo Fisher Scientific). The eluted peptides were analysed on an Orbitrap Exploris 480 (Thermo Fisher Scientific) operated in positive ion mode with data-independent acquisition (DIA-MS). MS1 spectra were collected in the range of 495 to 745 m/z at a 15,000 resolution, and MS2 spectra were collected in the range of 200

m/z and above at a 30,000 resolution with normalised collision energy at 26%. Data were processed using DIA-NN [69] (v1.8.1) for protein and peptide identification and quantification. A spectral library was generated using deep learning-based spectral prediction, and the MS/MS data were searched against the *H. brasiliensis* annotated protein sequences. Parameters were set as follows: enzyme, trypsin; maximum missed cleavage site, 1; static modification, carbamidomethylation; and FDR thresholds for precursor and protein identification, 1%.

Lipid extraction and LC-MS/MS analysis

The latex samples for lipidome analysis were collected following the same method as those for proteome analysis, as detailed in Note S5. To simulate real field-to-factory conditions, latex samples were transported without chemical preservation before lipidomic analysis. This approach reflects the time point resembling rubber processing, which differs from the profile reported under controlled laboratory conditions.

100 µL of chloroform was added to 1 mg of latex samples, followed by shaking, sonication, and centrifugation. Methanol was then added to 50 µL of the supernatant, stirred, and centrifuged again, and the supernatant was collected. Lysophosphatidylcholine (18:1-d7) was added as an internal standard at a final concentration of 500 ng/mL, and the sample was analysed using LC-MS/MS. Operational blanks (prepared without samples) and quality control samples (prepared by equally mixing aliquots from each sample solution) were analysed in the same manner. LC-MS analysis was performed using an UltiMate 3000 BioRS system coupled to an LTQ Orbitrap XL mass spectrometer (Thermo Fisher Scientific). Chromatographic separation was achieved using an L-column3 C18 metal-free column (2.0 mm I.D. × 100 mm, 2 µm particle size; CERI). Data analysis, including peak detection, lipid species estimation within each class, and sample alignment, was conducted using MS-DIAL (v4.80). Lipid peaks were considered detected if they met the following criteria: peak height

in QC samples ≥10,000; peak area at least twice that of the operational blank; average signal-to-noise ratio ≥3; and consistent detection across all QC measurements with a coefficient of variation <20%. The lipid content was determined relative to an internal standard by comparing the analyte peak area. OPLS-DA was performed using the R package ropls (v1.39.0) to estimate lipid differences between *H. brasiliensis* and other *Hevea* species. Lipid abundances were log-transformed prior to analysis. Variable importance in projection values were obtained from ropls, and *p*-values were calculated using a two-sample t-test.

Abbreviations

AACT: acetyl-CoA C-acetyltransferase; ADGGA: acyl diacylglyceryl glucuronide; AHexSIS: acylhexosyl sitosterol; AHexSTS: acylhexosyl stigmasterol; Cer_AP: ceramide alpha-hydroxy fatty acid-phytospingosine; CL: cardiolipin; CME: 4-(cytidine 5′-diphospho)-2-C-methyl-D-erythritol; CMEC: 2-C-methyl-D-erythritol-2,4-cyclodiphosphate; CMK: 2-C-methyl-d-erythritol 4-phosphate kinase; CMS: 2-C-methyl-D-erythritol 4-phosphate cytidylyltransferase; COI1: coronatine-insensitive 1; CPT: cis-prenyltransferase; CPTL: CPT-like; CTR1: constitutive triple response 1; DG: diacylglycerol; DGDG: digalactosyldiacylglycerol; DGGA: diacylglyceryl glucuronide; DMAPP: dimethylallyl pyrophosphate; DPMD: diphosphomevalonate decarboxylase; DXP: 1-deoxy-d-xylulose 5-phosphate; DXR: DXP reductoisomerase; DXS: 1-deoxy-d-xylulose 5-phosphate synthase; EIL1: ethylene insensitive-like 1; EIN2/3: ethylene-insensitive 2/3; ETR: ethylene receptors; FPS: farnesyl pyrophosphate synthase; G3P: glyceraldehyde 3-phosphate; GGPP: geranylgeranyl pyrophosphate; GGPS: geranylgeranyl pyrophosphate synthase; GPP: geranyl pyrophosphate; GPS: geranyl pyrophosphate synthase; HDR: 4-hydroxy-3-methylbut-2-enyl diphosphate reductase; HDS: 4-hydroxy-3-methylbut-2-enyl-diphosphate synthase; HMBD: 1-hydroxy-2-methyl-2-butenyl 4-diphosphate; HMGR: hydroxymethylglutaryl-CoA

reductase; HMGS: hydroxymethylglutaryl-CoA synthase; IPI: isopentenyl diphosphate isomerase; IPP: isopentenyl diphosphate; JAZ: jasmonate ZIM-domain; MCS: 2-C-methyl-D-erythritol 2,4-cyclodiphosphate synthase; MEP: 2-C-methyl-d-erythritol 4-phosphate; MVK: mevalonate kinase; MYC: MYC transcription factor; MVA-5-p: mevalonate-5-phopshate; MVA-5-pp: MVA 5-diphosphate; OxFA: oxidized fatty acid; PA: phosphatidic acid; PCME: 2-phospho-4-(cytidine 5′-diphospho)-2-C-methyl-D-erythritol; PI: phosphatidylinositol; PMK: phospho-MVA kinase; REF: rubber elongation factor; SRPP: small rubber particle protein; ST: sulfatide; TG: triacylglycerol.

Acknowledgements

We thank Dr. Thomas Wijaya (IRRI, Indonesia), Marliana Sheny (JICA) and Satoru Mitani (JICA) for coordination of our international research. We thank Kapus Suroso Rahutomo for access to Natural rubber plantation. This research was partially supported by the Science and Technology Research Partnership for Sustainable Development (SATREPS).

Author contributions

N-S.L., E.O-K, Y.M-N, A.S.O. and M.M. designed the experiments and summarised the data. N-S.L. and Y.N-M. performed bioinformatic analysis of genomic, transcriptomic, proteomic and lipidomic data. Sampling and material preparation were done by F.O., T. K., E.O-K. and H.H. M.K., O.N. and S.K. performed Hi-C genome sequencing and assembly. N-S.L. and M.M. wrote the manuscript.

Data Availability

The genome data were deposited in the DDBJ/EMBL/GenBank under the accession number of SRR31189420-SRR31189422 (*H. brasiliensis*), SRR31191093 (*H. guianensis*),

SRR31191092 (*H. collina*), SRR31192722 (*H. pauciflora*), SRR31192721 (*H. confusa*), and SRR31192751 (*H. spruceana*). The Iso-seq data are available from SRR31201725 (latex), SRR31201726 (leaf), SRR31203224 (petiole), and SRR31203225(bark). Proteomic data have been deposited in the ProteomeXchange repository with identifier PXD057483.

Competing interests

The authors declare that they have no competing interests.

References

1. Metcalfe CR. Distribution of latex in the plant kingdom. Econ Bot. 1967;21:115-27. https://doi.org/.10.1007/BF02897859.

2. van Beilen JB and Poirier Y. Establishment of new crops for the production of natural rubber. Trends Biotechnol. 2007;25:522-9. https://doi.org/.10.1016/j.tibtech.2007.08.009.

3. Gonçalves PdS, Cardoso M and Ortolani A. Origin, variability and domestication of *Hevea*-a review. Pesq Agropec Brasileira. 1990;25:135–56.

4. Priyadarshan P and Goncalves PdS. Use of *Hevea* gene pool in rubber tree (*Hevea brasiliensis* Muell.-Arg) breeding. Planter. 2002;78:123-38.

5. Priyadarshan PM and Clément-Demange A. Breeding *Hevea* rubber: formal and molecular genetics. Adv Genet. 2004;52:51-115. https://doi.org/.10.1016/s0065-2660(04)52003-5.

6. Cornish K, Wood DF and Windle JJ. Rubber particles from four different species, examined by transmission electron microscopy and electron-paramagnetic-resonance spin labeling, are found to consist of a homogeneous rubber core enclosed by a

contiguous, monolayer biomembrane. Planta. 1999;210:85-96. https://doi.org/.10.1007/s004250050657.

7. Takahashi S and Koyama T. Structure and function of *cis*-prenyl chain elongating enzymes. Chem Rec. 2006;6:194-205. https://doi.org/.10.1002/tcr.20083.

8. Lakusta AM, Kwon M, Kwon EG, Stonebloom S, Scheller HV and Ro DK. Molecular studies of the protein complexes involving *cis*-prenyltransferase in guayule (*Parthenium argentatum*), an alternative rubber-producing plant. Front Plant Sci. 2019;10:165. https://doi.org/.10.3389/fpls.2019.00165.

9. Yamashita S, Yamaguchi H, Waki T, Aoki Y, Mizuno M, Yanbe F, et al. Identification and reconstitution of the rubber biosynthetic machinery on rubber particles from *Hevea brasiliensis*. Elife. 2016;5. https://doi.org/.10.7554/eLife.19022.

10. Chow K-S, Mat-Isa MN, Bahari A, Ghazali A-K, Alias H, Mohd.-Zainuddin Z, et al. Metabolic routes affecting rubber biosynthesis in *Hevea brasiliensis* latex. J Exp Bot. 2012;63:1863-71. https://doi.org/.10.1093/jxb/err363.

11. Dennis MS and Light DR. Rubber elongation factor from *Hevea brasiliensis*. Identification, characterization, and role in rubber biosynthesis. J Biol Chem. 1989;264:18608-17.

12. Berthelot K, Lecomte S, Estevez Y and Peruch F. *Hevea brasiliensis* REF (Hev b 1) and SRPP (Hev b 3): An overview on rubber particle proteins. Biochimie. 2014;106:1-9. https://doi.org/.10.1016/j.biochi.2014.07.002.

13. Wang X, Shi M, Wang D, Chen Y, Cai F, Zhang S, et al. Comparative proteomics of primary and secondary lutoids reveals that chitinase and glucanase play a crucial combined role in rubber particle aggregation in *Hevea brasiliensis*. J Proteome Res. 2013;12:5146-59. https://doi.org/.10.1021/pr400378c.

14. Deng X, Guo D, Yang S, Shi M, Chao J, Li H, et al. Jasmonate signalling in the regulation of rubber biosynthesis in laticifer cells of rubber tree, *Hevea brasiliensis*. J Exp Bot. 2018;69:3559-71. https://doi.org/.10.1093/jxb/ery169.

15. Liu JP, Zhuang YF, Guo XL and Li YJ. Molecular mechanism of ethylene stimulation of latex yield in rubber tree (*Hevea brasiliensis*) revealed by de novo sequencing and transcriptome analysis. BMC Genomics. 2016;17:257. https://doi.org/.10.1186/s12864-016-2587-4.

16. Chao J, Wu S, Shi M, Xu X, Gao Q, Du H, et al. Genomic insight into domestication of rubber tree. Nat Commun. 2023;14:4651. https://doi.org/.10.1038/s41467-023-40304-y.

17. Cheng H, Song X, Hu Y, Wu T, Yang Q, An Z, et al. Chromosome-level wild *Hevea brasiliensis* genome provides new tools for genomic-assisted breeding and valuable loci to elevate rubber yield. Plant Biotechnol J. 2023;21:1058-72. https://doi.org/.10.1111/pbi.14018.

18. Pootakham W, Sonthirod C, Naktang C, Ruang-Areerate P, Yoocha T, Sangsrakru D, et al. *De novo* hybrid assembly of the rubber tree genome reveals evidence of paleotetraploidy in *Hevea* species. Sci Rep. 2017;7:41457. https://doi.org/.10.1038/srep41457.

19. Rahman AY, Usharraj AO, Misra BB, Thottathil GP, Jayasekaran K, Feng Y, et al. Draft genome sequence of the rubber tree *Hevea brasiliensis*. BMC Genomics. 2013;14:75. https://doi.org/.10.1186/1471-2164-14-75.

20. Tang C, Yang M, Fang Y, Luo Y, Gao S, Xiao X, et al. The rubber tree genome reveals new insights into rubber production and species adaptation. Nat Plants. 2016;2:16073. https://doi.org/.10.1038/nplants.2016.73.

21. Fang Y, Xiao X, Lin J, Lin Q, Wang J, Liu K, et al. Pan-genome and phylogenomic analyses highlight *Hevea* species delineation and rubber trait evolution. Nat Commun. 2024;15:7232. https://doi.org/.10.1038/s41467-024-51031-3.

22. Lau NS, Makita Y, Kawashima M, Taylor TD, Kondo S, Othman AS, et al. The rubber tree genome shows expansion of gene family associated with rubber biosynthesis. Sci Rep. 2016;6:28594. https://doi.org/.10.1038/srep28594.

23. Liu J, Shi C, Shi CC, Li W, Zhang QJ, Zhang Y, et al. The chromosome-based rubber tree genome provides new insights into spurge genome evolution and rubber biosynthesis. Mol Plant. 2020;13:336-50. https://doi.org/.10.1016/j.molp.2019.10.017.

24. Cornish K. Similarities and differences in rubber biochemistry among plant species. Phytochemistry. 2001;57:1123-34. https://doi.org/.https://doi.org/10.1016/S0031-9422(01)00097-8.

25. Asawatreratanakul K, Zhang YW, Wititsuwannakul D, Wititsuwannakul R, Takahashi S, Rattanapittayaporn A, et al. Molecular cloning, expression and characterization of cDNA encoding cis-prenyltransferases from *Hevea brasiliensis*. A key factor participating in natural rubber biosynthesis. Eur J Biochem. 2003;270:4671-80. https://doi.org/.10.1046/j.1432-1033.2003.03863.x.

26. Epping J, van Deenen N, Niephaus E, Stolze A, Fricke J, Huber C, et al. A rubber transferase activator is necessary for natural rubber biosynthesis in dandelion. Nat Plants. 2015;1:15048. https://doi.org/.10.1038/nplants.2015.48.

27. Niephaus E, Müller B, van Deenen N, Lassowskat I, Bonin M, Finkemeier I, et al. Uncovering mechanisms of rubber biosynthesis in *Taraxacum koksaghyz* - role of cis-prenyltransferase-like 1 protein. Plant J. 2019;100:591-609. https://doi.org/.10.1111/tpj.14471.

28. Oh SK, Kang H, Shin DH, Yang J, Chow KS, Yeang HY, et al. Isolation, characterization, and functional analysis of a novel cDNA clone encoding a small rubber particle protein from *Hevea brasiliensis*. J Biol Chem. 1999;274:17132-8. https://doi.org/.10.1074/jbc.274.24.17132.
29. Chao J, Chen Y, Wu S and Tian W-M. Comparative transcriptome analysis of latex from rubber tree clone CATAS8-79 and PR107 reveals new cues for the regulation of latex regeneration and duration of latex flow. BMC Plant Biol. 2015;15:104. https://doi.org/.10.1186/s12870-015-0488-3.
30. Florez-Velasco N, Ramos VF, Magnitskiy S and Balaguera-López H. Ethylene and jasmonate as stimulants of latex yield in rubber trees (*Hevea brasiliensis*): Molecular and physiological mechanisms. A systematic approximation review. Advanced Agrochem. 2024;3:279-88. https://doi.org/.https://doi.org/10.1016/j.aac.2024.07.003.
31. Pujade-Renaud V, Clement A, Perrot-Rechenmann C, Prevot JC, Chrestin H, Jacob JL, et al. Ethylene-induced increase in glutamine synthetase activity and mRNA levels in *Hevea brasiliensis* latex cells. Plant Physiol. 1994;105:127-32. https://doi.org/.10.1104/pp.105.1.127.
32. Zhang Y, Leclercq J and Montoro P. Reactive oxygen species in *Hevea brasiliensis* latex and relevance to Tapping Panel Dryness. Tree Physiol. 2016;37:261-9. https://doi.org/.10.1093/treephys/tpw106.
33. Huang Y, Fang Y, Long X, Liu L, Wang J, Zhu J, et al. Characterization of the rubber tree metallothionein family reveals a role in mitigating the effects of reactive oxygen species associated with physiological stress. Tree Physiol. 2018;38:911-24. https://doi.org/.10.1093/treephys/tpy003.
34. Zhang MJ, Fu Q, Chen MS, He H, Tang M, Ni J, et al. Characterization of the bark storage protein gene (*JcBSP*) family in the perennial woody plant *Jatropha curcas* and

the function of *JcBSP1* in *Arabidopsis thaliana*. PeerJ. 2022;10:e12938. https://doi.org/.10.7717/peerj.12938.

35. Yusof F, Chow K-S, Ward MA and Walker JMJJoRR. A stimulator protein of rubber biosynthesis from *Hevea brasiliensis* latex. J Rubber Res. 2000;3:232-49.

36. Asghari Barzegar Z, Taghvaei Ganjali S, Malekzadeh M and Motiee F. Correlations between lipid contents of natural rubber and tensile properties of natural rubber-based compound, using attenuated total reflection Fourier transform infrared spectroscopy. Spectrosc Lett. 2023;56:1-13. https://doi.org/.10.1080/00387010.2022.2153143.

37. Yu H, Wang Q, Li J, Liu Y, He D, Gao X, et al. Effect of lipids on the stability of natural rubber latex and tensile properties of its films. J Rubber Res. 2017;20:213-22. https://doi.org/.10.1007/BF03449153.

38. Zhang BL, Huang HH, Wang YZ, Ding L and Liang Y. Study on molecular structure and property of highly purified natural rubber. J Anal Appl Pyrol. 2018;134:130-5. https://doi.org/.10.1016/j.jaap.2018.05.018.

39. Bae SW, Jung S, Choi SC, Kim MY and Ryu SB. Lipid composition of latex and rubber particles in *Hevea brasiliensis* and *Taraxacum kok-saghyz*. Molecules. 2020;25 https://doi.org/.10.3390/molecules25215110.

40. d'Auzac J, Jacob JL and Chrestin H. Physiology of rubber tree latex. Boca Raton, FL: CRC Press; 1989.

41. Chotiphan R, Vaysse L, Lacote R, Gohet E, Thaler P, Sajjaphan K, et al. Can fertilization be a driver of rubber plantation intensification? Ind Crop Prod. 2019;141:111813. https://doi.org/.https://doi.org/10.1016/j.indcrop.2019.111813.

42. Sreelatha S, Simon SP and Jacob JJJoRR. On the possibility of using ATP concentration in latex as an indicator of high yield in *Hevea brasiliensis*. J Rubb Res. 2004;7:71-8.

43. Sreelatha S, Jacob J, Mercykutty VC, Simon SP, Krishnakumar R and Annamalainathan K. ATP concentration in latex as an indicator for early evaluation of yield in *Hevea brasiliensis*. J Plant Crops. 2014;42:48-53.

44. Schultes RE. A brief taxonomic view of the genus *Hevea*. Kuala Lumpur: Malaysian Rubber Research and Development Board; 1990.

45. Priyadarshan PM. Genetic resources. Biology of *Hevea* rubber. Springer Cham; 2017. p. 83-105.

46. Lin T, Xu X, Ruan J, Liu S, Wu S, Shao X, et al. Genome analysis of *Taraxacum kok-saghyz* Rodin provides new insights into rubber biosynthesis. Natl Sci Rev. 2018;5:78-87. https://doi.org/.10.1093/nsr/nwx101.

47. Wuyun TN, Wang L, Liu H, Wang X, Zhang L, Bennetzen JL, et al. The hardy rubber tree genome provides insights into the evolution of polyisoprene biosynthesis. Mol Plant. 2018;11:429-42. https://doi.org/.10.1016/j.molp.2017.11.014.

48. Yang Y and Benning C. Functions of triacylglycerols during plant development and stress. Curr Opin Biotechnol. 2018;49:191-8. https://doi.org/.https://doi.org/10.1016/j.copbio.2017.09.003.

49. Dufourc EJ. Sterols and membrane dynamics. J Chem Biol. 2008;1:63-77. https://doi.org/.10.1007/s12154-008-0010-6.

50. Kooijman EE, Chupin V, de Kruijff B and Burger KNJ. Modulation of membrane curvature by phosphatidic acid and lysophosphatidic acid. Traffic. 2003;4:162-74. https://doi.org/.https://doi.org/10.1034/j.1600-0854.2003.00086.x.

51. Carrasco S and Mérida I. Diacylglycerol, when simplicity becomes complex. Trends Biochem Sci. 2007;32:27-36.

52. Testerink C and Munnik T. Phosphatidic acid: a multifunctional stress signaling lipid in plants. Trends Plant Sci. 2005;10:368-75.

53. Ong CW and Shamsul Bahri AR. Rubber particles: Size, molecular weight and their distributions detected in wild *Hevea* Species. J Biol Agric Healthc. 2016;6:98-103.

54. Kadota M, Nishimura O, Miura H, Tanaka K, Hiratani I and Kuraku S. Multifaceted Hi-C benchmarking: what makes a difference in chromosome-scale genome scaffolding? Gigascience. 2020;9. https://doi.org/.10.1093/gigascience/giz158.

55. Deng LH, Luo MW, Zhang CF and Zeng HC. Extraction of high-quality RNA from rubber tree leaves. Biosci Biotechnol Biochem. 2012;76:1394-6. https://doi.org/.10.1271/bbb.120014.

56. Kolmogorov M, Yuan J, Lin Y and Pevzner PA. Assembly of long, error-prone reads using repeat graphs. Nat Biotechnol. 2019;37:540-6. https://doi.org/.10.1038/s41587-019-0072-8.

57. Durand NC, Shamim MS, Machol I, Rao SS, Huntley MH, Lander ES, et al. Juicer provides a one-click system for analyzing loop-resolution Hi-C experiments. Cell Syst. 2016;3:95-8. https://doi.org/.10.1016/j.cels.2016.07.002.

58. Kajitani R, Toshimoto K, Noguchi H, Toyoda A, Ogura Y, Okuno M, et al. Efficient de novo assembly of highly heterozygous genomes from whole-genome shotgun short reads. Genome Res. 2014;24:1384-95. https://doi.org/.10.1101/gr.170720.113.

59. Alonge M, Lebeigle L, Kirsche M, Jenike K, Ou S, Aganezov S, et al. Automated assembly scaffolding using RagTag elevates a new tomato system for high-throughput genome editing. Genome Biol. 2022;23:258. https://doi.org/.10.1186/s13059-022-02823-7.

60. Manni M, Berkeley MR, Seppey M, Simão FA and Zdobnov EM. BUSCO update: Novel and streamlined workflows along with broader and deeper phylogenetic coverage for scoring of eukaryotic, prokaryotic, and viral genomes. Mol Biol Evol. 2021;38:4647-54. https://doi.org/.10.1093/molbev/msab199.

61. Tarailo-Graovac M and Chen N. Using RepeatMasker to identify repetitive elements in genomic sequences. Curr Protoc Bioinformatics. 2009;25:Unit 4.10. https://doi.org/.10.1002/0471250953.bi0410s25.

62. Campbell MS, Law M, Holt C, Stein JC, Moghe GD, Hufnagel DE, et al. MAKER-P: a tool kit for the rapid creation, management, and quality control of plant genome annotations. Plant Physiol. 2014;164:513-24. https://doi.org/.10.1104/pp.113.230144.

63. Makita Y, Ng KK, Veera Singham G, Kawashima M, Hirakawa H, Sato S, et al. Large-scale collection of full-length cDNA and transcriptome analysis in *Hevea brasiliensis*. DNA Res. 2017;24:159-67. https://doi.org/.10.1093/dnares/dsw056.

64. Contreras-Moreira B, Cantalapiedra CP, García-Pereira MJ, Gordon SP, Vogel JP, Igartua E, et al. Analysis of plant pan-genomes and transcriptomes with GET_HOMOLOGUES-EST, a clustering solution for sequences of the same species. Front Plant Sci. 2017;8:184. https://doi.org/.10.3389/fpls.2017.00184.

65. Emms DM and Kelly S. OrthoFinder: phylogenetic orthology inference for comparative genomics. Genome Biol. 2019;20:238. https://doi.org/.10.1186/s13059-019-1832-y.

66. Minh BQ, Schmidt HA, Chernomor O, Schrempf D, Woodhams MD, von Haeseler A, et al. IQ-TREE 2: New models and efficient methods for phylogenetic inference in the genomic era. Mol Biol Evol. 2020;37:1530-4. https://doi.org/.10.1093/molbev/msaa015.

67. Mendes FK, Vanderpool D, Fulton B and Hahn MW. CAFE 5 models variation in evolutionary rates among gene families. Bioinformatics. 2021;36:5516-8. https://doi.org/.10.1093/bioinformatics/btaa1022.

68. Chen D, Zhang T, Chen Y, Ma H and Qi J. Tree2GD: a phylogenomic method to detect large-scale gene duplication events. Bioinformatics. 2022;38:5317-21. https://doi.org/.10.1093/bioinformatics/btac669.

69. Demichev V, Messner CB, Vernardis SI, Lilley KS and Ralser M. DIA-NN: neural networks and interference correction enable deep proteome coverage in high throughput. Nat Methods. 2020;17:41-4. https://doi.org/.10.1038/s41592-019-0638-x.

Figure legends

Figure 1: Genome assembly and annotation of *Hevea* species. (A) Summary of genome assembly and annotation statistics across *Hevea* species. (B) Circos plot showing the chromosomal organisation of *Hevea brasiliensis* (outermost ring) and comparative features across *Hevea* species. From outside to inside: proteome abundance in *H. confusa*, *H. collina*, *H. spruceana*, *H. guianensis*, and *H. brasiliensis*, with blue indicating high abundance and red indicating low abundance; rubber biosynthesis-related genes are labelled; orthologous gene relationships between *H. brasiliensis* and *A. thaliana*, *M. esculenta*, *R. communis*, and *J. curcas*; GC content across chromosomes; and the innermost links represent gene duplications within *H. brasiliensis*. (C) Chromosomal distribution of telomeric regions in *H. brasiliensis*, with telomeric regions marked by blue triangles and gene density represented by a heatmap, where red indicates gene-rich regions and blue indicates gene-poor regions.

Figure 2: Pangene analysis of *Hevea*. Simulations of the (A) increase in the pangene size and (B) decrease in the core gene size. (C) Composition of the *Hevea* pangene. Gene ontology (GO) enrichment of the (D) core, (E) soft-core, (F) shell and (G) cloud genes relative to the pangene. (H) GO annotation of the *H. brasiliensis*-specific genes.

Figure 3: Comparative genomics of *Hevea* species. (A) Maximum likelihood phylogeny inferred from single-copy orthologues. The column on the right represents the number of expanded and contracted gene families. Bar chart shows the distribution of single-copy, multiple-copy and, and unique orthologs in the 11 plant species. Gene ontology enrichment of genes expanded (blue) and contracted (pink) in (B) *Hevea* species and (C) *H. brasiliensis*. The distribution of (D). K_s and (E) 4DTv values of the syntenic gene pairs in the *H. brasiliensis, H. guianensis, H. pauciflora, H. collina*, and *H. confusa* genomes.

Figure 4: Analysis of rubber biosynthesis-related genes. (A) Maximum likelihood tree of CPT amino acid sequences from *Hevea* and other plants. (b) Hi-C plot showing TAD-likes

structure for CPT1-4 on chromosome 18. (C) Maximum likelihood tree of CPTL nucleotide sequences from *Hevea* and other plants. (D) Maximum likelihood tree of REF and SRPP amino acid sequences from *Hevea* and other plants. At, *A. thaliana*; Hb, *H. brasiliensis*; Hcf, *H. confusa*; Hcl, *H. collina*; Hg, *H. guianensis*; Hp, *H. pauciflora*; Hs, *H. spruceana*; Ls, *L. sativa*; Pa, *P. argentatum*; Sl, *S. lycopersicum*; and Tb, *T. brevicorniculatum*.

Figure 5: Rubber biosynthesis pathway and proteomic profiles in *Hevea* species. Heatmap showing protein abundance (log2), with colour coding in which yellow indicates high abundance and blue signifies low abundance. MVA pathway: AACT, HMGS, HMGR, MVK, MVA-5-p, MVA-5-pp, PMK, DPMD. MEP pathway: G3P, DXS, DXP, DXS, DXR, MEP, CMS, CME, CMK, PCME, MCS, CMEC, HDS, HMBD, HDR. Formation of isoprenoid precursors: IPP, IPI, DMAPP, GPS, GGPP, FPS, GGPS, GPP. Rubber particle associated proteins: CPT, REF, SRPP, CPTL. Jasmonic acid signalling: COI1, JAZ, MYC. Ethylene signalling: ETR, CTR1,EIN2/3, EIL1. (B) Differentially abundant proteins between *H. brasiliensis* and other *Hevea* species samples. Bubble plot showing proteins with significantly different abundance levels, where bubble size is proportional to statistical significance value.

Figure 6: Lipidome profiles across *Hevea* species. (A) Bar plot showing the composition of lipid classes in the *Hevea* latex lipidome. Hb, *H. brasiliensis*; Hg, *H. guianensis*; Hs, *H. spruceana*; Hcl, *H. collina*; Hcf, *H. confusa*. (B) Orthogonal partial least squares discriminant analysis score plot showing the separation between *H. brasiliensis* and other *Hevea* species samples. (C) Heatmap showing lipid abundance (log10), with colour coding at which yellow indicates high abundance and blue signifies low abundance. Only differential abundant lipids with VIP > 1 and $p < 0.05$ are shown.

Click here to access/download;Figure;Fig. 1.tif

A

	H. brasiliensis	*H. guianensis*	*H. pauciflora*	*H. spruceana*	*H. collina*	*H. confusa*
No. of scaffolds	14,722	545,786	558,352	577,834	488,774	574,685
Longest scaffold (Mb)	99.10	65.93	71.52	66.96	59.61	65.79
Scaffold N50 (Mb)	78.35	31.28	49.81	0.29	29.06	38.51
Assembly size (Gb)	1.71	1.63	1.41	1.77	1.42	1.35
GC content (%)	34.48	34.01	34.17	34.33	33.80	34.44
No. of gene models	84,076	44,254	53,110	52,848	73,123	82,122
Mean transcript length (bp)	3,874	3,135	3,358	3,044	3,447	2,987
Mean coding sequence length (bp)	706	677	798	693	599	678
Average no. of exons per transcript	4.4	4.3	4.9	4.6	4.4	4.4
Mean exon length (bp)	160	159	164	152	136	156
Mean intron length (bp)	790	691	591	609	809	637

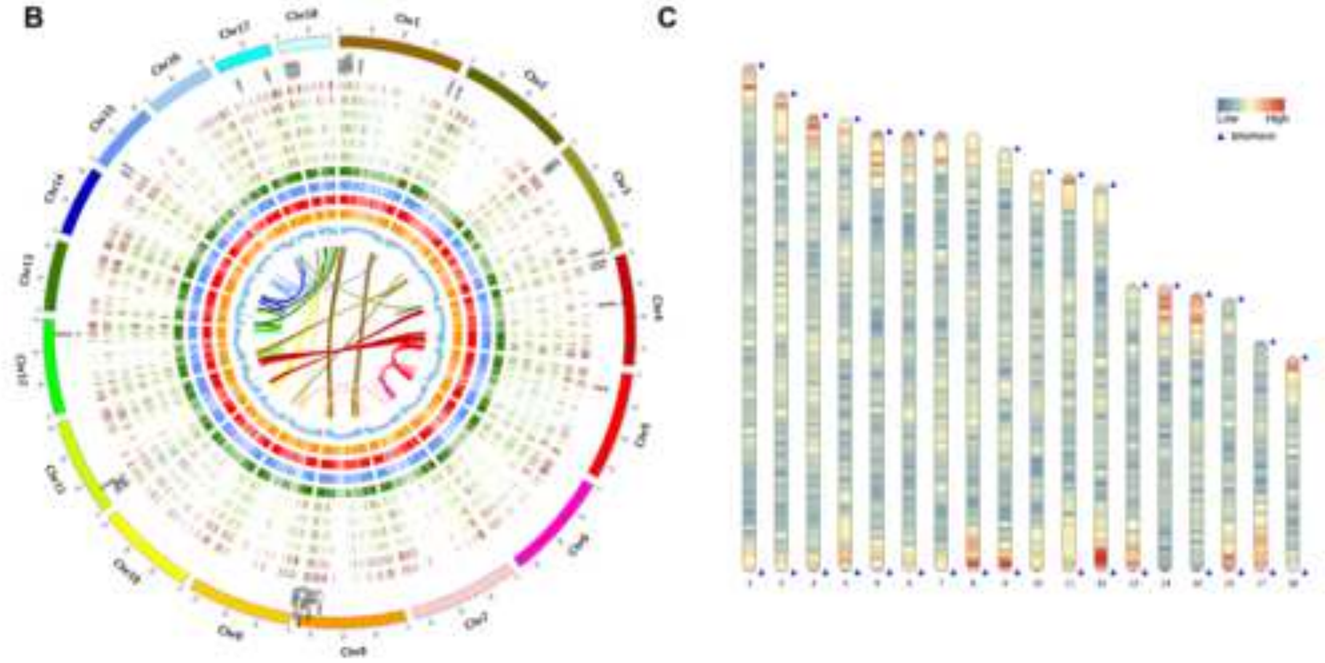

Figure 2

Click here to access/download;Figure;Fig. 2.tif

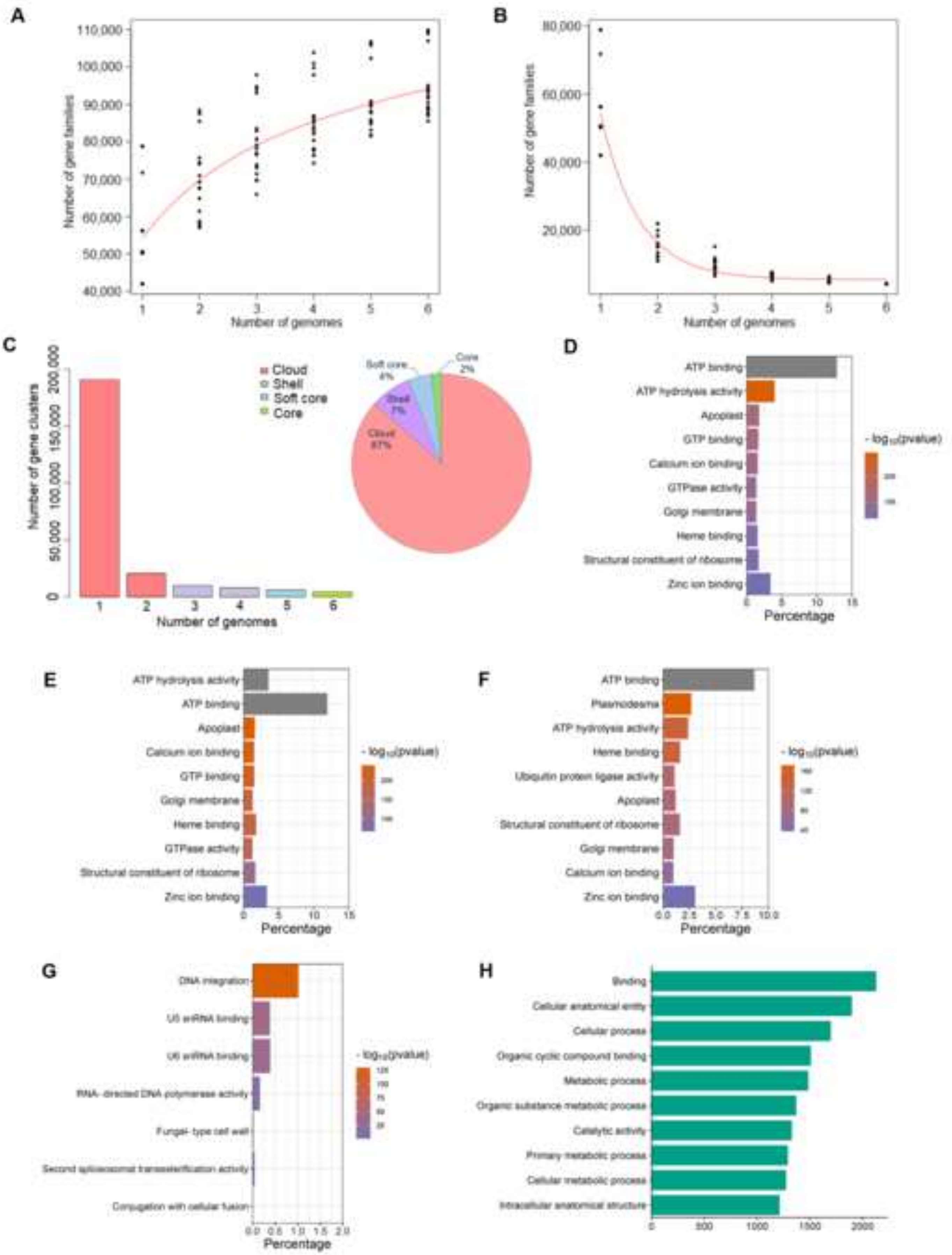

Figure 3

Click here to access/download;Figure;Fig. 3.tif

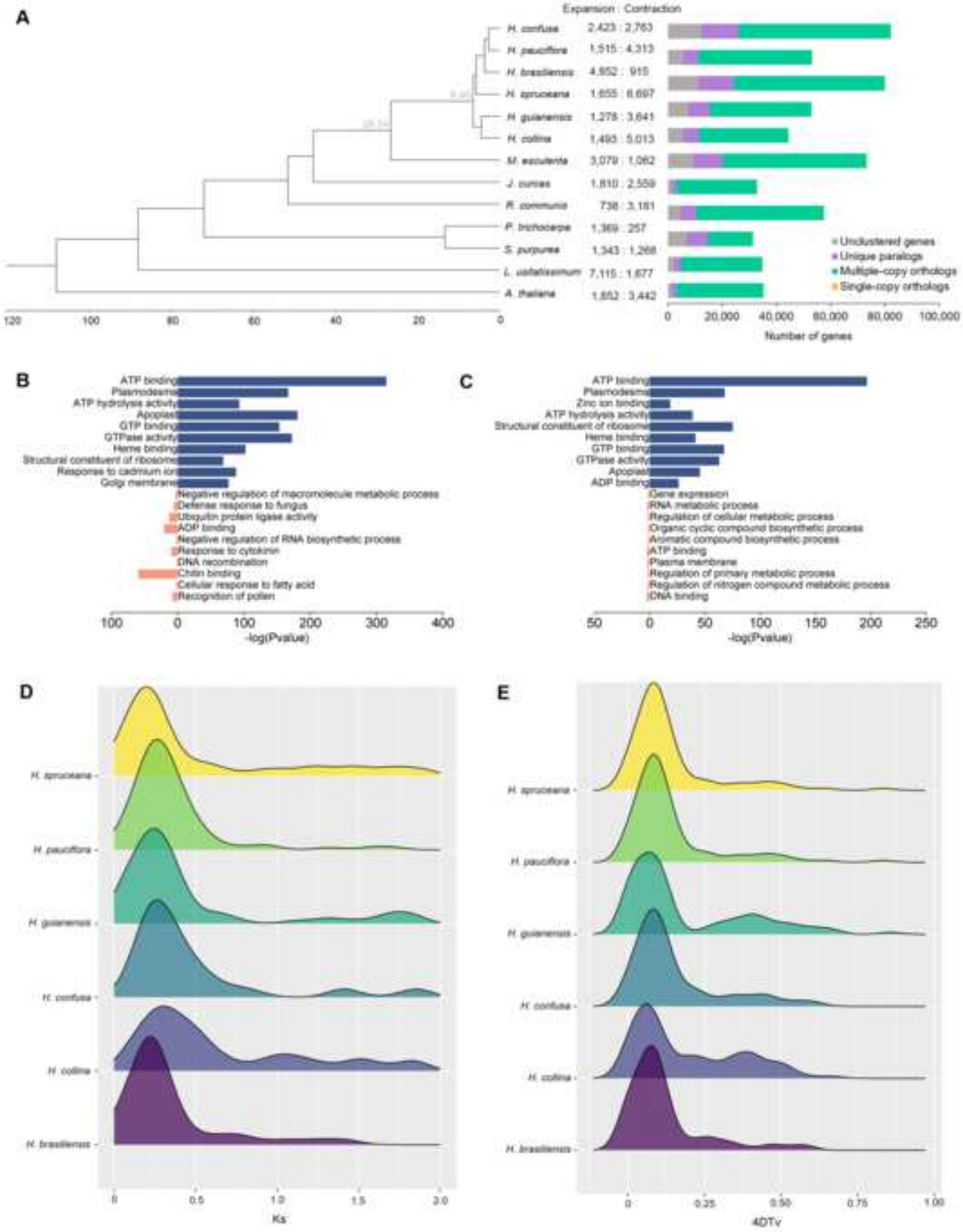

Figure 4 Click here to access/download;Figure;Fig. 4.tif

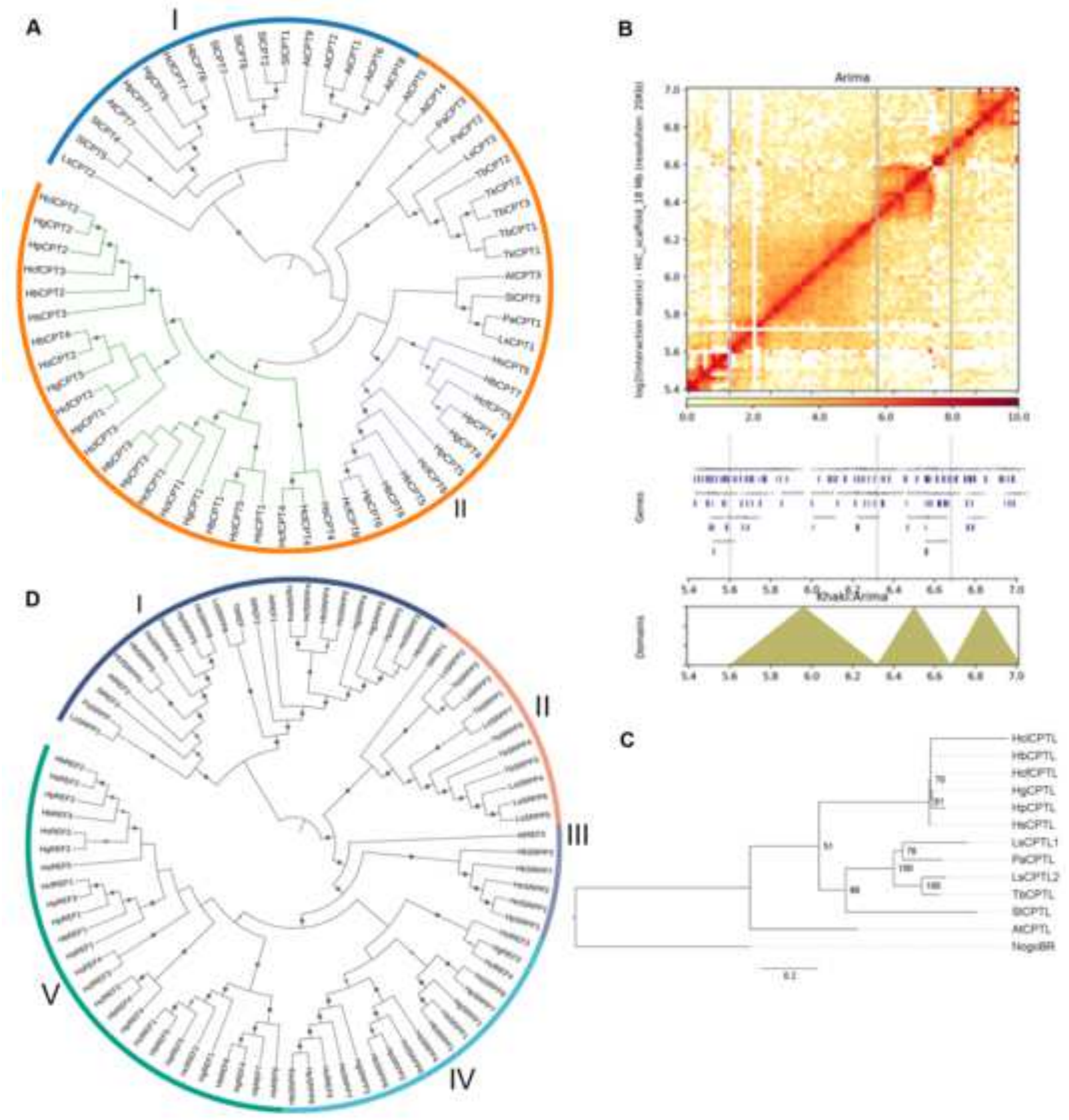

Figure 5 Click here to access/download;Figure;Fig. 5.tif

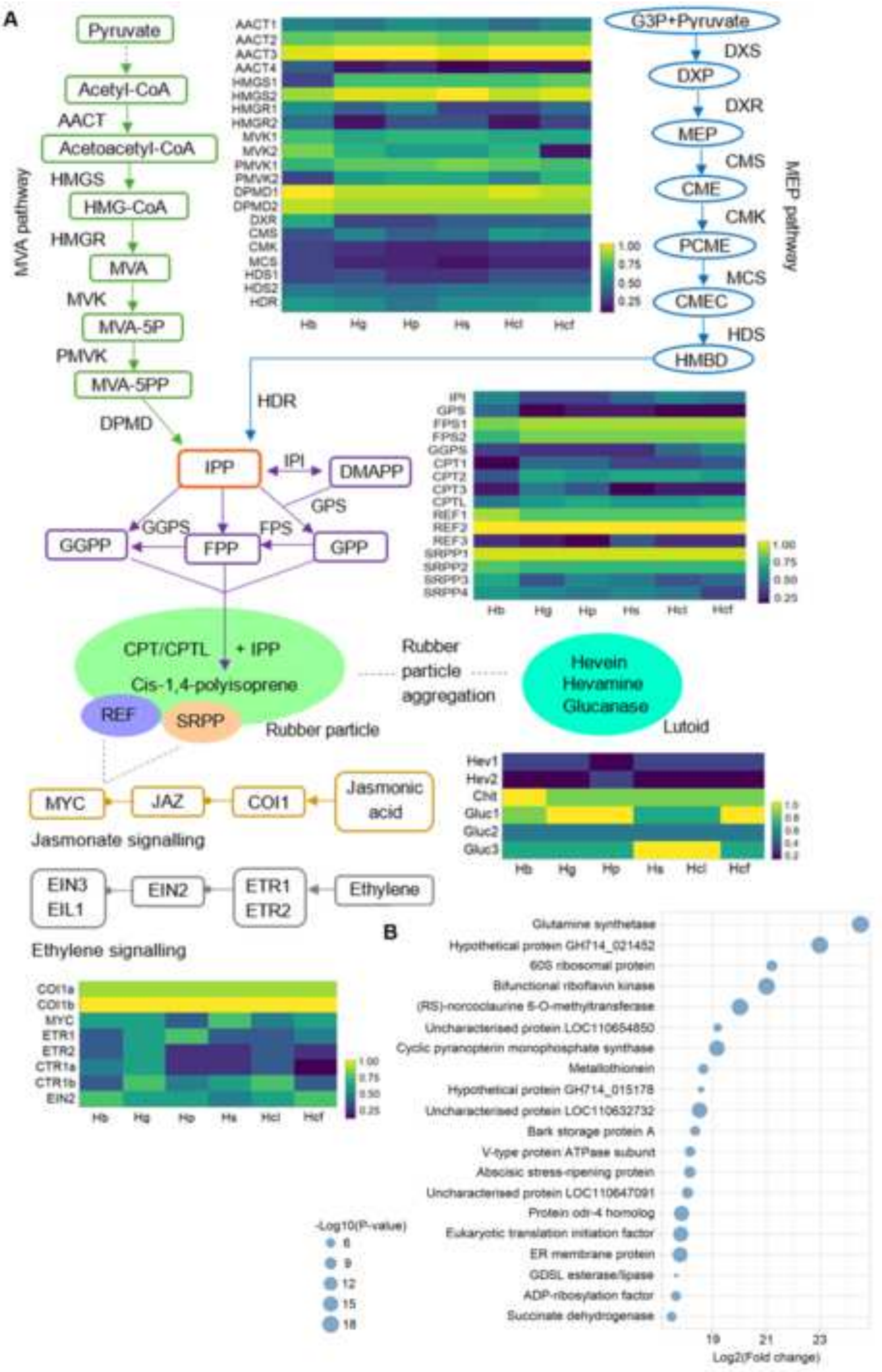

Figure 6

Click here to access/download;Figure;Fig. 6.tif

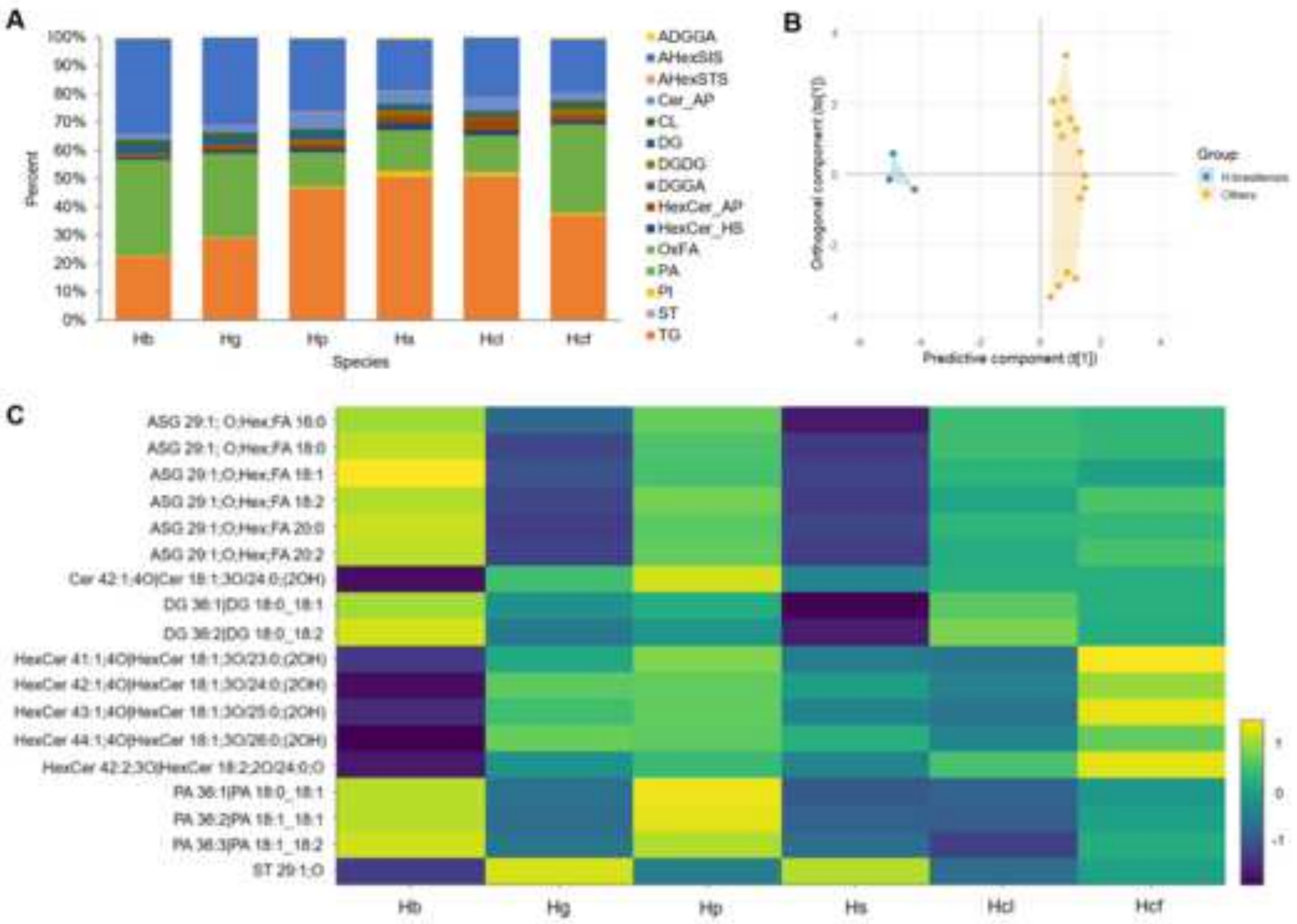

Click here to access/download
Supplementary Material
Supplementary Material_Figures_Comparative genomics Hevea.docx

Click here to access/download
Supplementary Material
Supplementary Table_Comparative genomics Hevea.xlsx

Personal Cover Click here to access/download;Personal Cover;Cover letter Hevea_190525.docx

Editor-in-Chief
GigaScience Date: 20th May 2025

Dear Editor,

We are pleased to submit our manuscript titled "Comparative genomics and multi-omics analyses reveal the evolution and physiological basis of rubber biosynthesis in *Hevea* species", for consideration in GigaScience. This study presents a comprehensive comparative analysis of six *Hevea* species and varieties, integrating genomics, proteomics, and lipidomics to elucidate the evolutionary and physiological underpinnings of rubber biosynthesis.

Our work is aligned with GigaScience's focus on data-intensive research and reproducibility. By constructing *Hevea* pangene and performing comparative genomic analyses, we identified conserved and expanded ATP-related gene functions potentially supporting metabolic demands of latex biosynthesis. Proteomics revealed species-specific protein abundance patterns in latex that may explain differences in regeneration and yield. Lipidomics further uncovered variation in lipid composition among species, which may influence rubber particle stability and latex characteristics. These multi-omics data resources, alongside our evolutionary insights, provide a valuable foundation for further functional studies and guiding breeding strategies for rubber-producing species.

All authors have approved the manuscript for submission. We confirm that the content has not been published or submitted elsewhere. There are no competing interests to declare.

We believe that our data-rich, open science approach and the integrated multi-omics findings presented in this study fit well with the scope of GigaScience, and we hope that you will consider the manuscript for review.

We would like to suggest the following potential reviewers:

<u>Potential Reviewers</u>

1. Name: Dr. Christian Schulze Gronover
   Affiliation: Fraunhofer Institute for Molecular Biology and Applied Ecology IME, 48149, Münster, Germany.
   Email: christian.schulze.gronover@ime.fraunhofer.de
2. Name: Dr. Wirulda Pootakham
   Affiliation: National Center for Genetic Engineering and Biotechnology (BIOTEC), National Science and Technology Development Agency, Pathum Thani, Thailand.
   Email: wirulda.poo@biotec.or.th
3. Name: Dr. Margaret H Frank

Affiliation: School of Integrative Plant Science, Cornell University, Ithaca, NY 14850, USA.
Email: mhf47@cornell.edu

4. Name: Dr. Marcin Nowicki
Affiliation: Department of Entomology and Plant Pathology, The University of Tennessee, Knoxville, TN, USA.
Email: mnowicki@utk.edu
5. Name: Prof. Satoshi Tabata,
Affiliation: Kazusa DNA Research Institute, Japan.
E-mail: tabata@kazusa.or.jp

With best regards,

Minami Matsui, PhD (Corresponding author)

Group Director

Synthetic Genomics Research Group,

RIKEN Center for Sustainable Resource Science

1-7-22 Suehiro, Tsurumi, Yokohama,

Kanagawa 230-0045 Japan.

Email: minami@riken.jp
