# Supplementary material for: Comparative genomics and multiomics analyses reveal the evolution and physiological basis of rubber biosynthesis in Hevea species
Source: Gigascience. 2025 Oct 10;14:giaf115. doi: 10.1093/gigascience/giaf115 (PMC12512020; doi:10.1093/gigascience/giaf115)
Supplement: giaf115_GIGA-D-25-00195_Revision_1 [file giaf115_giga-d-25-00195_revision_1.pdf]

GigaScience

Comparative genomics and multi-omics analyses reveal the evolution and physiological basis of rubber biosynthesis in Hevea species

--Manuscript Draft--

**Manuscript Number:**	GIGA-D-25-00195R1
**Full Title:**	Comparative genomics and multi-omics analyses reveal the evolution and physiological basis of rubber biosynthesis in Hevea species
**Article Type:**	Research
**Funding Information:**	Science and Technology Research Partnership for Sustainable Development — Prof. Minami Matsui
**Abstract:**	There are multiple species within the Hevea genus, each exhibiting distinct characteristics, but many remain underexplored due to their lower latex productivity. While Hevea brasiliensis is the primary source of natural rubber, other Hevea species represent valuable gene pools that could be leveraged in breeding programs to enhance latex yield, biosynthesis efficiency, and the physicochemical properties of latex. With increasing interest in enhancing natural rubber traits, these lesser-known species are being revisited for their underexplored genetic diversity. In this study, we performed a pangene analysis of six Hevea species and varieties, integrating proteomic and lipidomic data to investigate genetic and metabolic variation related to rubber biosynthesis and latex composition. The pangene analysis revealed conserved and expanded ATP-related functions, underscoring ATP's role in latex production. The proteomic data identified key enzymes involved in rubber biosynthesis and differentially abundant proteins related to latex regeneration, suggesting that regeneration capacity may influence yield efficiency. Lipidomic profiling uncovered species-specific lipid compositions associated with membrane dynamics and rubber particle stability, which may contribute to latex properties. These findings provide valuable insights into Hevea's genomic and metabolic diversity, supporting future breeding programs aimed at improving natural rubber production and its performance in various applications.
**Corresponding Author:**	Minami Matsui<br>RIKEN Center for Sustainable Resource Science<br>JAPAN
**Corresponding Author Secondary Information:**	
**Corresponding Author's Institution:**	RIKEN Center for Sustainable Resource Science
**Corresponding Author's Secondary Institution:**	
**First Author:**	Nyok Sean Lau
**First Author Secondary Information:**	
**Order of Authors:**	Nyok Sean Lau
	Emiko Ohkubo-Kurihara
	Yuko Makita Nakai
	Fetrina Oktavia
	Tomoko Kuriyama
	Yukio Kurihara
	Hidefumi Hamasaki
	Yuki Nakamura
	Mitsutaka Kadota
	Osamu Nishimura

Powered by Editorial Manager® and ProduXion Manager® from Aries Systems Corporation

	Shigehiro Kuraku
	Ahmad Sofiman Othman
	Minami Matsui
**Order of Authors Secondary Information:**	
**Response to Reviewers:**	Responses to reviewer 1’s comments:<br>Response 1: Thank you for your thorough and constructive comments. We are pleased that you found the manuscript to be a valuable and robust contribution to plant genomics and rubber research.<br>Most of the remaining Hevea species are endemic to Brazil, especially in the Amazon region. Over the past few years, we attempted to obtain samples through a research collaboration agreement with the Brazilian authorities. Despite our efforts, logistical and regulatory challenges prevented the acquisition of genomic DNA or viable tissues from those species. Consequently, we focused on six Hevea from the Indonesian national germplasm collection, which provided a representative subset for comparative genomic analysis. While this does not encompass the entire genus, we believe the current dataset offers meaningful insights into Hevea evolution and rubber biosynthesis.<br>Functional study in another rubber-producing species, Taraxacum brevicorniculatum showed that overexpression of ATP-dependent enzymes in the mevalonate pathway enhanced isoprenoid flux. This evidence supports the functional relevance of ATP metabolism-related gene expansion in Hevea, and we have incorporated this discussion into the revised manuscript (page 16).<br>For differentially abundant proteins, we have also expanded our discussion on the overexpression of glutamine synthetase in Arabidopsis thaliana and functional studies on metallothionein to support our proteomics findings (page 18).<br>Response 2: We have revised the Methods section (page 26) to include a more detailed explanation of our sampling approach and how it differs from previous studies that used chemical inhibitors to prevent lipid degradation. We also provide a rationale for our experimental design and explain how these methodological differences may have influenced the lipidomic profiles observed (page 14).<br>Response 3 To explore potential links between genomic traits and environmental adaptation, we analysed cold stress regulatory genes in Hevea species, the ICE–CBF–COR pathway. We observed an expansion of ICE and CBF genes in the highland H. guianensis, which may reflect genomic adaptation to low temperature stress at higher altitude. This analysis has been incorporated into the Discussion (page 17), with further details provided in Supplementary Text S7 and Supplementary Table S33.<br>Response 4: We have performed correlation analyses between the proteome and lipidome profiles, and the results have been included in Figure 6D and Supplementary Table S32. The corresponding results have been added to page 15, and the discussion has been included on page 19.<br>Response 5: Details on the parameters used for differential protein abundance analysis and missing data handling have been added to Supplementary Text S6 (page 5), and the details for differential lipid abundance are included on page 27 of the main manuscript.<br>Response 6: Orthologous gene analysis using GET_HOMOLOGUES-EST classified the genes into 239,600 families. The resulting gene cluster list is available and will be uploaded to GigaDB. However, the previous login to Gigadb is not accessible, we will complete the data upload once access is provided.<br>Response 7: Information on the number of biological replicates for the proteomic and lipidomic analyses have been added to the Methods section on page 25-26.<br>Response 8: The description of p-value adjustment for multiple testing in the differential protein abundance analysis has been added to Supplementary Text S6. The heatmap of protein abundance in Fig. 5, the bar plot of lipid classes in Fig. 6A, and the lipid abundance heatmap in Fig. 6C were all plotted using the mean values from three biological replicates. These details have been incorporated into the respective figure legends.<br>Response 9: The sample preparation method for proteomic analysis by LC-MS/MS has been deposited on protocols.io, and the DOI is cited in the Methods section (page 25).<br>Response 10: We have revised the conclusion to present a broader perspective, as suggested by the reviewer.<br>Response 11: We have added RRIDs accessions for the tools described in the Methods section.

Powered by Editorial Manager® and ProduXion Manager® from Aries Systems Corporation

	Responses to reviewer 2's comments:<br>Response 1: Thank you for the positive comments and suggestions.<br>As recommended, we have revised the sentence on page 18 "The substantial enrichment of proteins involved in nitrogen metabolism, ROS (reactive oxygen species) homeostasis, and protein synthesis suggests that the efficiency of rubber biosynthesis in H. brasiliensis relies on the capacity for latex regeneration" to frame it more cautiously as a hypothesis to be tested.<br>Response 2: Thank you for pointing this out. We have corrected the author's name to "Yuko Makita" and updated the "Author Contributions" abbreviation to "Y.M." accordingly.<br>Response 3: We have written out ATP in full as adenosine triphosphate upon its first mention on page 7.<br>Response 4: As suggested, we have replaced "at" with "in" in the Figure 5 legend.<br>Response 5: We have revised LC-MS/MS to its full form, liquid chromatography–tandem mass spectrometry, at its first mention on page 14.<br>Response 6: We have revised "isopentyl pyrophosphate" to "isopentyl diphosphate" on page 3 for consistency.
Additional Information:	
Question	Response
Are you submitting this manuscript to a special series or article collection?	No
**Experimental design and statistics**<br><br>Full details of the experimental design and statistical methods used should be given in the Methods section, as detailed in our Minimum Standards Reporting Checklist. Information essential to interpreting the data presented should be made available in the figure legends.<br><br>Have you included all the information requested in your manuscript?	Yes
**Resources**<br><br>A description of all resources used, including antibodies, cell lines, animals and software tools, with enough information to allow them to be uniquely identified, should be included in the Methods section. Authors are strongly encouraged to cite Research Resource Identifiers (RRIDs) for antibodies, model organisms and tools, where possible.<br><br>Have you included the information requested as detailed in our Minimum Standards Reporting Checklist?	Yes

Powered by Editorial Manager® and ProduXion Manager® from Aries Systems Corporation

**Availability of data and materials**<br><br>All datasets and code on which the conclusions of the paper rely must be either included in your submission or deposited in publicly available repositories (where available and ethically appropriate), referencing such data using a unique identifier in the references and in the "Availability of Data and Materials" section of your manuscript.<br><br>Have you have met the above requirement as detailed in our Minimum Standards Reporting Checklist?	Yes
GigaScience has policies and guidelines in place for the use of generative AI-writing tools such as ChatGPT. If you have used such writing tools to assist with writing the manuscript this must be declared and cited in the text. Authors should not list AI-writing tools and other AI-assisted technologies as an author or co-author and should acknowledge that they are fully responsible for text generated or refined by AI-writing tools.<p><br><br>A summary of use (particularly in the introduction or among methods) needs to be included at the end of the paper, and the outputs should also be included as a supplementary file hosted in GigaDB or other open repositories. Please read our guidelines for more information. <p><br><br>By submitting to GigaScience, you are aware of the journal's AI-writing tools policy, and if you have declared use of such tools below, you have acknowledged this where appropriate in your manuscript and have made a summary of use and outputs available. <p><br><b>AI-assisted writing tools have been used in the preparation of this manuscript?	No

Powered by Editorial Manager® and ProduXion Manager® from Aries Systems Corporation

Manuscript

Click here to access/download;Manuscript;Manuscript_Comparative_genom

Comparative genomics and multi-omics analyses reveal the evolution and physiological basis of rubber biosynthesis in *Hevea* species

Nyok-Sean Lau[1,2†], Emiko Okubo-Kurihara[1,3†], Yuko Makita[1,4†], Fetrina Oktavia[5], Tomoko Kuriyama[1], Yukio Kurihara[1], Hidefumi Hamasaki[1], Yuki Nakamura[6], Mitsutaka Kadota[7], Osamu Nishimura[7], Shigehiro Kuraku[7], Ahmad Sofiman Othman[2,8], Minami Matsui[1,9*]

[1]RIKEN Center for Sustainable Resource Science, Yokohama 230-0045, Japan

[2]Centre for Chemical Biology, Universiti Sains Malaysia, Bayan Lepas, 11900, Penang, Malaysia

[3]Department of Biology, Keio University, Yokohama 223-8522, Japan

[4]Mebashi Institute of Technology, 460-1, Kamisadori, Maebashi, Gunma, Japan

[5]Indonesian Rubber Research Institute, 15 Jln. Raya Palembang Pangkalan Balai Km. 29, Sembawa, Banyuasin 30953 Indonesia

[6]Plant Lipid Research Team, RIKEN Center for Sustainable Resource Science, Yokohama 230-0045, Japan

[7]Laboratory for Phyloinformatics, RIKEN Center for Biosystems Dynamics Research (BDR), Kobe 650-0047, Japan

[8]School of Biological Sciences, Universiti Sains Malaysia, Minden, 11800, Penang, Malaysia

[9]Yokohama City University, Kihara Institute for Biological Research, Totsuka, Yokohama, Kanagawa, Japan

[*]Correspondence address. Minami Matsui, RIKEN Center for Sustainable Resource Science, Yokohama 230-0045, Japan. Email: minami@riken.jp

[†]These authors contributed equally.

Nyok Sean Lau [0000-0002-0708-5970]; Emiko Ohkubo-Kurihara [0000-0002-7171-7346]; Yuko Makita Nakai [0000-0001-5745-6007]; Fetrina Oktavia [0000-0002-0271-6200]; Yukio Kurihara [0000-0002-3149-8097]; Hidefumi Hamasaki [0009-0001-8888-7448]; Yuki Nakamura [0000-0003-2897-4301]; Mitsutaka Kadota [0000-0002-1674-6697]; Osamu Nishimura [0000-0003-1969-2580]; Shigehiro Kuraku [0000-0003-1464-8388]; Ahmad Sofiman Othman [0000-0002-4944-4177]; Minami Matsui [0000-0001-5162-2668];

Abstract

There are multiple species within the *Hevea* genus, each exhibiting distinct characteristics, but many remain underexplored due to their lower latex productivity. While *Hevea brasiliensis* is the primary source of natural rubber, other *Hevea* species represent valuable gene pools that could be leveraged in breeding programs to enhance latex yield, biosynthesis efficiency, and the physicochemical properties of latex. With increasing interest in enhancing natural rubber traits, these lesser-known species are being revisited for their underexplored genetic diversity. In this study, we performed a pangene analysis of six *Hevea* species and varieties, integrating proteomic and lipidomic data to investigate genetic and metabolic variation related to rubber biosynthesis and latex composition. The pangene analysis revealed conserved and expanded ATP-related functions, underscoring ATP's role in latex production. The proteomic data identified key enzymes involved in rubber biosynthesis and differentially abundant proteins related to latex regeneration, suggesting that regeneration capacity may influence yield efficiency. Lipidomic profiling uncovered species-specific lipid compositions associated with membrane dynamics and rubber particle stability, which may contribute to latex properties. These findings provide valuable insights into *Hevea*'s genomic and metabolic diversity, supporting future breeding programs aimed at improving natural rubber production and its performance in various applications.

Keywords: *Hevea* species, comparative genomics, proteomics, lipidomics, rubber biosynthesis, latex metabolism

Introduction

Natural rubber stands as an indispensable natural polymer, integral to the manufacture of diverse industrial products, owing to its unparalleled physical properties. Despite extensive research, no synthetic polymer has yet been developed that can replicate the physical attributes of natural rubber. Although more than 2500 plant species are known to biosynthesize natural rubber [1, 2], commercial natural rubber production depends exclusively on latex derived from the Pará rubber tree, *Hevea brasiliensis* Muell. Arg. The genus *Hevea*, belonging to the family Euphorbiaceae, comprises eleven species including *H. brasiliensis*, *Hevea bethamiana*, *Hevea camporum*, *Hevea camargoana*, *Hevea guianensis*, *Hevea microphylla*, *Hevea nitida*, *Hevea pauciflora*, *Hevea paludosa*, *Hevea rigidifolia* and *Hevea spruceana* [3, 4]. Although the genus is diverse, *H. brasiliensis* is the primary source of natural rubber, accounting for over 90% of global production, estimated at 14 million tons annually according to the website of Food and Agriculture Organization of the United Nations. Originally from the Amazon basin, *H. brasiliensis* has seen its cultivation shift mainly to Southeast Asia since Henry Wickham introduced rubber tree seeds to the region in 1876 [5]. As a result, global rubber production is now largely dependent on a limited genetic pool from a small collection of seeds. Over the past decades, global rubber production has increased due to the expansion of cultivated areas and a significant increase in productivity, with genetic improvement playing a crucial role.

Natural rubber, a *cis*-1,4-polyisoprene polymer, is synthesised and stored in specialised phloem cells known as laticifers, specifically on the surfaces of rubber particles [6]. The elongation of rubber chains is catalysed by rubber transferase, a member of the *cis*-prenyltransferase (CPT) family, through the sequential addition of isopentenyl diphosphate (IPP) to prenyl groups [7]. A CPT-like (CPTL) protein is thought to be associated with the rubber transferase complex, where it interacts with and activates CPT [8, 9]. In plants, IPP is synthesised via the cytosolic mevalonate (MVA) and plastidic methylerythritol (MEP) pathways, with evidence suggesting the MVA pathway as the primary source for natural rubber production in *H. brasiliensis* [10]. The synthesis of natural rubber is also supported by rubber elongation factors (REFs) and small rubber particle proteins (SRPPs), the two most abundant proteins on rubber particles, which are essential for rubber particle stability and rubber biosynthesis [11, 12]. Latex coagulation, a crucial process for rubber production, is mediated by lutoid-derived proteins such as hevamine/chitinase, β-1,3-glucanase and hevein, which are released upon lutoid disruption during tapping and promote rubber particle aggregation [13]. Latex biosynthesis is regulated by hormonal signalling pathways, particularly ethylene and jasmonates, which can be produced in response to wounding or applied exogenously. Jasmonates are known to play a role in secondary laticifer differentiation, while ethylene has been shown to prolong the duration of latex flow [14, 15]. Understanding the genetic basis of the rubber biosynthetic pathway is integral to optimising rubber yield and improving production efforts.

Although multiple genome assemblies have been published for *H. brasiliensis*, genomic analyses of other *Hevea* species have only recently become available [16-23]. Genetic information beyond *H. brasiliensis* remains limited, restricting the gene space accessible for comparative studies and breeding. To help address this gap and improve our understanding of diversity within the genus, we performed a pangene analysis of five *Hevea*

species or varieties alongside the *H. brasiliensis* reference genome. Our comparative genomic analyses, integrated with proteomics and lipidomics data, enabled the exploration of key agronomic traits, including core components of rubber biosynthesis, factors influencing its capacity, and lipid composition relevant to latex properties. This multi-omics approach offers a broader view of molecular variation related to rubber biosynthesis and latex traits in *Hevea*, providing a useful resource to inform future efforts in genetic improvement and species conservation.

Results

Hevea species genome sequences as a resource for diversity studies

We previously reported a draft genome sequence of *H. brasiliensis* RRIM 600, a historically important clone widely planted during the early expansion of rubber cultivation in East Asia [22] (Supplementary Text S1). To support comparative analyses, RRIM600 was selected for high-quality genome reconstruction. A chromosome-level genome was constructed using a hybrid approach integrating PacBio SMRT and Hi-C sequencing (Supplementary Table S1). The initial PacBio assembly, based on 169.94 Gb of reads, yielded a 1.71 Gb genome with an N50 of 550.35 Kb (Supplementary Table S2). Refinement with Hi-C reads (105-fold coverage) produced a final assembly of 1.71 Gb, with a scaffold N50 of 78.35 Mb (Fig. 1a, Supplementary Text S2 and Table S3). More than 78% of the assembly was anchored onto 18 pseudo-chromosomes, covering 91% of the estimated 1.88 Gb genome size (Fig. 1b, Supplementary Fig. S1-2 and Table S4). Benchmarking Universal Single-Copy Orthologs (BUSCO) assessment showed 98.7% completeness, and over 90% of PacBio Iso-Seq transcripts aligned to the assembly, indicating high contiguity and sequence quality (Supplementary Text S3, Fig. S3 and Table S5-6). Centromeric regions were identified in all 18 chromosomes, while telomeric sequences were detected at both ends of most chromosomes, with only one telomeric end identified in four chromosomes (Fig. 1c,

Supplementary Table S7-S8). To explore genomic diversity in *Hevea*, we sequenced five additional *Hevea*: *H. guianensis*, *H. pauciflora*, *H. spruceana*, *H. confusa* (*H. pauciflora* var. *confusa*) and *H. collina* (*H. guianensis* var. *collina*) (Supplementary Text S4). Their genomes were *de novo* assembled using 56.9 to 95.6× Illumina reads and synteny scaffolded with *H. brasiliensis* as reference. Assembly sizes ranged from 1.35 to 1.77 Gb, with N50 values of 0.29 to 38.51 Mb and GC content between 33.80 and 34.48% (Supplementary Fig. S4).

Annotation using the MAKER pipeline predicted an average of ~65,000 high-confidence gene models, with *H. brasiliensis* containing the most (84,076) and *H. guianensis* the fewest (44,254). Predicted protein-coding genes were mapped to functional databases, including NCBI NR, SwissProt, TrEMBL, InterPro, Gene Ontology (GO), and Kyoto Encyclopedia of Genes and Genomes (KEGG) (Supplementary Table S9). The gene models averaged 3,044 to 3,874 bp in transcript length, 599 to 798 bp in coding sequence length, and four exons per gene (Supplementary Fig. S5). Noncoding RNA genes, including tRNAs, rRNAs, miRNAs, and snoRNAs, were also identified (Supplementary Table S10). Repeat annotation using *de novo* and homology-based approaches revealed variability across *Hevea* genomes. *H. brasiliensis* exhibited the highest repeat content (74.1%), while *H. collina* had the lowest (33.3%) (Supplementary Table S11). Long terminal repeats (LTRs) dominated, constituting 31.21-70.54% of the genomes, with Gypsy elements being particularly abundant (31.45-54.92%). In contrast, long interspersed nuclear elements (LINEs) accounted for only 1.04-1.60%, and DNA transposons ranged from 0.79-1.94%. The Gypsy-to-Copia ratio was consistently high across species. Divergence analysis of transposable elements based on Kimura distances revealed a peak divergence rate of around 6 for *H. pauciflora* and 9-10 for other species, with the Gypsy superfamily being the major component of the peak (Supplementary Fig. S6).

Comparative genomics of *Hevea* species

To study the genomic landscape of the *Hevea* genus, we conducted comparative analyses on the assembled genomes of *H. brasiliensis*, *H. guianensis*, *H. pauciflora*, *H. spruceana*, *H. collina*, and *H. confusa* (Fig. 2a-c). Orthologous gene analysis classified all genes from these six genomes into 239,660 families. Our findings revealed that 2% of the gene families were present in the core-cluster, 4% in the soft-core, 7% in the shell and 87% in the cloud clusters. The core and soft-core clusters harboured highly conserved genes shared across all analysed genomes, while the shell and cloud clusters contained flexible genes that may serve as reservoirs of genetic variability contributing to adaptive potential. GO enrichment analysis of the core and soft-core gene clusters identified significantly enriched GO terms, with the largest gene members included in adenosine triphosphate (ATP) binding, ATP hydrolysis activity, and apoplast (Fig. 2d-e, Supplementary Table S12-13).In the shell gene cluster, enriched pathways also encompassed ATP binding, and ATP hydrolysis activity, but also plasmodesma (Fig. 2f, Supplementary Table S14). Functional annotation of the cloud genes revealed significant enrichment of GO terms associated with DNA integration, U5 snRNA binding, and U6 snRNA binding (Fig. 2g, Supplementary table S15).

Modelling of pangene size showed that the total number of gene families increased with the addition of each new genome, indicating that *Hevea* genomes exhibit genetic variation, and the analysed genomes may not fully capture the diversity of the genus. Furthermore, species-specific genes in *H. brasiliensis* were annotated, revealing an abundance of pathways related to binding, cellular anatomical structures, and cellular processes (Fig. 2h). These fundamental function genes have likely evolved independently to support the distinct biological characteristics of *H. brasiliensis*.

To trace the evolutionary history of *Hevea*, gene family clustering was performed using gene sets from 13 plant species, including six *Hevea*, six representative Malpighiales species, and the outgroup *Arabidopsis thaliana*. This analysis identified 1,156 single-copy

orthologs shared between *Hevea* and the other plants, which were used for phylogenetic reconstruction and divergence time estimation (Fig. 3a). Our results indicate that all *Hevea* species share a common ancestor around 6.46 million years ago (mya), with *Hevea* diverging from *Manihot esculenta* approximately 26.54 mya. *H. brasiliensis* exhibited high conservation of orthologous genes with *M. esculenta*, *Ricinus communis*, and *Jatropha curcas*, particularly with *M. esculenta* (Fig. 1b). The phylogenomic analysis revealed two distinct clades within *Hevea*: one containing *H. brasiliensis, H. spruceana, H. pauciflora*, and its variety *H. confusa*; the other comprising *H. guianensis* and its variety *H. collina*.

To investigate changes in gene family size during evolution, we analysed expansions and contractions of gene families in *Hevea* relative to the most recent common ancestor of the 13 plant species, as well as in *H. brasiliensis* relative to the common ancestor of *Hevea* (Fig. 3a). Expanded gene families in *Hevea* species were enriched for functions related to ATP binding, plasmodesma, and ATP hydrolysis activity (Fig. 3b, Supplementary table S16). In *H. brasiliensis*, expanded gene families also showed enrichment in ATP binding, plasmodesma, and zinc ion binding (Fig. 3c, Supplementary table S17). Conversely, contracted gene families in *Hevea* species exhibited an overrepresentation of functions associated with the negative regulation of macromolecule metabolic process, defence response to fungus, and ubiquitin protein ligase activity (Supplementary table S18). In *H. brasiliensis*, contracted gene families were overrepresented with gene expression, RNA metabolic process, and the regulation of cellular metabolic process (Supplementary table S19).

For dating the whole-genome duplication event in *Hevea*, a genomic analysis based on self-comparison of *H. brasiliensis, H. guianensis, H. pauciflora, H. spruceana, H. collina* and *H. confusa* paralogous genes was carried out. All *Hevea* genomes exhibited a major left peak in the distributions of synonymous substitutions per synonymous site (K_s) and transversions at four-fold degenerate sites (4DTv) frequency, indicating a recent species-specific duplication

(Fig. 3d-e). K_s estimates for paralogs in *H. brasiliensis, H. guianensis, H. pauciflora, H. spruceana, H. collina*, and *H. confusa* suggested that this *Hevea*-specific duplication event may date back to ~12.3-28.7 mya. Additionally, a minor right peak in the 4DTv distribution and a right smooth peak in the K_s distribution correspond to the whole-genome triplication event across core eudicots.

Evolution of rubber biosynthesis potential in *Hevea* species

The biosynthesis of natural rubber in *Hevea* involves a network of metabolic pathways and key proteins. Genes linked to rubber biosynthesis were identified through genome annotation across *Hevea* species, and their activity in latex was confirmed by proteomics (Fig. 4-5, Supplementary Text S5). The relative abundances of proteins in latex were quantified using data-independent acquisition coupled with high-resolution mass spectrometry (Supplementary Text S6 and Table S20).

IPP monomer supplying pathways

At the core of this process are the pathways that supply IPP, the fundamental building block for natural rubber**.** IPP is produced via two compartmentalised metabolic routes: the MVA pathway in the cytosol and the MEP pathway in the plastids. In *Hevea*, 14–18 genes are annotated in the MVA pathway, while 14–22 are associated with the MEP pathway (Supplementary Table S21). Proteomic analysis identified 14 of the 18 MVA pathway proteins in latex but only seven from the MEP pathway (Fig. 5a, Supplementary table S22). Notably, MEP pathway proteins were detected at much lower levels, and 1-deoxy-D- xylulose 5-phosphate synthase (DXS), which catalyses the first MEP pathway step, was absent from the latex proteome. In the MVA pathway, all six key enzymes, acetyl-CoA acetyltransferase (AACT), 3-hydroxy-3-methylglutaryl-coenzyme A synthase (HMGS), HMG-CoA reductase

(HMGR), mevalonate kinase (MVK), phosphomevalonate kinase (PMVK), and mevalonate diphosphate decarboxylase (DPMD), were detected in latex, represented by two to four isoforms. AACT3, HMGS1, and DPMD were among the most abundant isoforms.

During rubber elongation, IPP and its isomer, dimethylallyl diphosphate, undergo sequential condensations to form geranyl pyrophosphate (GPP), farnesyl pyrophosphate (FPP), and geranylgeranyl pyrophosphate (GGPP), catalysed by trans-prenyltransferases such as geranyl pyrophosphate synthase (GPS), farnesyl pyrophosphate synthase (FPS), and geranylgeranyl pyrophosphate synthase (GGPS). These short-chain prenyl pyrophosphates serve as initiators for further IPP condensation, leading to the production of high molecular weight natural rubber. Among these enzymes, FPS was the most abundant in latex, indicating its role as the major trans-prenyltransferase in *Hevea*, as its product, FPP, has been identified as the main initiator of natural rubber biosynthesis [24].

Rubber particle−associated proteins

The key enzymes responsible for natural rubber biosynthesis, CPTs, were annotated in *Hevea* genomes: CPT1-8 in *H. brasiliensis*, CPT1-5 in *H. guianensis*, CPT1-7 in *H. pauciflora*, CPT1-5 in *H. spruceana*, CPT1-5 in *H. collina*, and CPT1-8 in *H. confusa* (Figure 4a, Supplementary Table S23). Chromosome 18 emerged as a hotspot for CPT genes, with several clustered together, suggesting coordinated regulation, supported by the observation of topologically associating domain structures for CPT1-4 in the *H. brasiliensis* leaf Hi-C analysis (Fig. 4b. Supplementary Fig. S7). Comparative analyses of CPT orthologs across multiple species, including *Linum usitatissimum*, *P. trichocarpa*, and *Salix purpurea* from Malpighiales, and *J. curcas*, *M. esculenta*, *R. communis*, and *Hevea* species from Euphorbiaceae, revealed a higher CPT gene count in Euphorbiaceae (average of seven) compared to non-Euphorbiaceae Malpighiales (average of four), indicating a lineage-specific duplication event in Euphorbiaceae (Supplementary Table S24).

Phylogenetic analysis, including rubber-producing species such as *Taraxacum koksaghyz, Taraxacum brevicorniculatum*, and *Parthenium argentatum*, revealed two CPT clades: clade I comprising chloroplastic CPTs, and clade II consisting of cytosolic CPTs, consistent with previous studies (Fig. 5a) [8]. Within clade II, specific *Hevea* CPTs formed subclades distinct from other rubber-producing plants. Proteomic analysis of *Hevea* latex showed that CPTs1-3 from clade II were exclusively expressed in latex, reinforcing their role in rubber biosynthesis. Multiple alignment of CPT sequences identified conserved regions I-V characteristic of known CPTs structure in *Hevea* [25] (Supplementary Fig. S8).

The rubber transferase complex, comprising CPTs and CPT-like (CPTL) proteins, has been characterised in *Hevea* and other rubber-producing species, where CPTLs anchor, activate, and stabilise CPTs on rubber particles without directly catalysing polymerisation [8, 9, 26, 27]. Genome annotation and proteomic detection confirm CPTLs are present across *Hevea* species beyond *H. brasiliensis*, supporting their involvement in rubber biosynthesis. Congruent with earlier observations, *Hevea* CPTLs exhibit low homology to CPTs, lacking conserved CPT motifs [26] (Supplementary Fig. S9). Phylogenetic analysis showed *Hevea* CPTLs forming a distinct cluster from those in *P. argentatum, T. brevicorniculatum, L. sativa, S. lycopersicum*, and *A. thaliana* (Fig. 4c).

REF and SRPP proteins, which are abundant in rubber particles, contribute positively to rubber biosynthesis by stabilising particle structure and preventing coagulation, with REF levels in latex correlating with rubber content [9, 11, 12, 28]. Genome annotation revealed an average of 12 REF/SRPP genes across *Hevea*, with *H. brasiliensis* containing the most (16) and *H. collina* the fewest (7) (Supplementary Table S25). Non-*Hevea* species in Malpighiales and Euphorbiaceae have an average of four REF/SRPP genes. Phylogenetic analysis delineated REF/SRPP genes into five evolutionary branches, with distinct clusters for *Hevea* genes, particularly in branch I, III, IV, and V (Fig. 4d). Sequence alignments confirmed the

presence of the REF domain and noted C-terminal differences between SRPP and REF, consistent with previous reports [12, 28] (Supplementary Fig. S10). Proteomic analysis identified *Hevea* REF1-3 and SRPP1-4 in latex, with REF2 and SRPP1 being the most abundant among latex biosynthesis-related proteins.

Rubber particle aggregation

In addition to rubber particles, lutoids of vacuolar origin are major components of latex, containing proteins essential for latex coagulation. Upon tapping, latex flow alters turgor pressure, causing lutoid rupture and the release of hevamine/chitinase and β-1,3-glucanase—proteins that promote rubber particle aggregation and latex coagulation [13, 29]. Concurrently, hevein contributes to particle aggregation, ultimately plugging latex vessels and stopping flow. Our study demonstrates a high abundance of chitinase and β-1,3-glucanase in *Hevea* latex proteomes, consistent with their proposed role in rubber coagulation (Supplementary Table S26).

Jasmonate and ethylene signalling in rubber biosynthesis

Tapping induces a wounding response in phloem tissues that activates the jasmonate and ethylene signalling pathways. Jasmonate signalling is crucial for laticifer differentiation and stimulates the expression of rubber biosynthesis-related genes (e.g., *FPS* and *SRPP*), leading to increased rubber yield [14]. The coronatine-insensitive 1 (COI1)–jasmonate ZIM-domain (JAZ)–MYC transcription factor module is central to this pathway: jasmonate perception leads to the degradation of JAZ repressors via the COI1 complex, releasing MYC2 and activating jasmonate-responsive genes [30]. Components of the COI1–JAZ–MYC2 regulatory module were annotated across *Hevea* species (Fig. 5a). Latex proteomes showed high levels

of COI1 and absence of JAZ, suggesting JAZ degradation and activation of jasmonate-responsive genes.

Ethylene treatment prolongs latex flow by promoting sucrose allocation, water transport, glycolysis, and C3 carbon fixation [15]. While key ethylene signalling components, ethylene receptors, constitutive triple response 1 (CTR1), and ethylene-insensitive 2/3, are annotated in *Hevea* genomes, their low abundance in latex proteomes (Fig. 5a) suggests that ethylene signalling is not the primary response to tapping-induced wounding.

Differential proteomics reveal potential factors influencing high-capacity rubber biosynthesis

We next identified proteins differentially expressed between *H. brasiliensis* and other *Hevea* species latex (Fig. 5b, Supplementary Fig. S11 and Table S27). Glutamine synthetase exhibited the largest fold change, increasing 24.51-fold in *H. brasiliensis* compared with other species. This observation aligns with study on ethylene treatment, which suggest that the glutamine synthetase/glutamate synthase cycle may constitute the major pathway for protein synthesis during latex regeneration [31]. Metallothionein, enriched 18.65-fold in *H. brasiliensis*, was also among the top differentially expressed proteins. While thiol, ascorbate, and glutathione are major reactive oxygen species (ROS) scavengers in latex [32], metallothionein expression has also been associated with maintaining ROS homeostasis under oxidative stress during tapping and regeneration [33]. Bark storage protein, a nitrogen storage protein not previously linked to latex production, was upregulated by 18.34-fold. The eukaryotic translation initiation factor eIF5A, a member of the broader eIF family involved in protein synthesis initiation, was enriched 17.80-fold in *H. brasiliensis*. Additionally, proteins linked to rubber biosynthesis, including those involved in IPP formation, rubber biosynthesis, rubber particle aggregation, and hormone signalling, were analysed for differential expression (Supplementary Table S28). Among these, only SRPP2 (2.19-fold), MVK2 (6.22-fold),

AACT4 (10.74-fold), and CTR1a (12.4-fold) showed significant enrichment in *H. brasiliensis*.

Lipidome profiling of *Hevea* species latex

In addition to *cis*-polyisoprene, *Hevea* latex comprises proteins, lipids, and carbohydrates, with lipids known to influence natural rubber's physical properties [34-36]. To explore this further, a targeted metabolomic analysis was performed to profile the lipid composition of latex from *Hevea* species within the same age range. The lipidomic profiles were analysed using liquid chromatography-tandem mass spectrometry (LC-MS/MS), identifying 53 lipid molecules classified into five main categories: fatty acyls, glycerolipids, glycerophospholipids, sphingolipids, and sterol lipids, which were further divided into 16 classes (Supplementary Table S29-S30). Triacylglycerol (TG), acylhexosyl sitosterol (AHexSIS), and phosphatidic acid (PA) were the most abundant lipid classes across species (Fig. 6a). *H. guianensis* and *H. confusa* exhibited lipid profiles similar to *H. brasiliensis*, characterised by high PA but lower TG. In contrast, *H. pauciflora*, *H. spruceana*, and *H. collina* showed a distinct profile with elevated TG and reduced PA. The lipid composition observed in this study differs from that reported previously [37], likely due to differences in sampling and processing protocols. While the prior study used inhibitors to prevent lipid degradation, our samples were collected and transported without chemical preservatives to reflect field-to-factory conditions.

A supervised orthogonal partial least-squares discriminative analysis (OPLS-DA) was performed to compare the latex lipidomes of *H. brasiliensis* and other *Hevea* species. The OPLS-DA score plots showed clear separation between the two groups, with R^2X(cum), R^2Y(cum), and Q^2(cum) values of 0.80, 0.97, and 0.801, respectively (Fig. 6b). Differential lipid species were identified using OPLS-DA and univariate analysis, selecting those with variable importance in projection (VIP) > 1 and $p < 0.05$. This analysis identified 18

differential lipid molecules, comprising six acylated steryl glucosides (ASG), one ceramide (Cer), two diacylglycerols (DG), five hexosylceramides (HexCer), three PA, and one sterol lipid (ST) (Fig. 6c, Supplementary Table S31). Among these, DG 36:1, DG 36:2, and PA 36:1 exhibited the highest upregulated fold changes, whereas HexCer 41:1, HexCer 43:1, and HexCer 42:1 were the most downregulated in *H. brasiliensis* relative to other *Hevea* species.

To investigate potential functional relationships between proteins and lipids in *Hevea* latex, we performed a correlation analysis between proteome and lipidome profiles across species. Using Pearson correlation with a threshold of $r > 0.75$ and $p < 0.01$, a total of 828 significant positive protein–lipid associations were identified (Supplementary Table S32). From these, the top 50 highly correlated protein–lipid pairs were selected for network visualisation using Cytoscape. Notably, several significant correlations involved cardiolipin (CL), a mitochondrial phospholipid, and mitochondrial-associated proteins such as NifU-like, succinate-CoA ligase, and glycine cleavage H. In addition, proteins involved in lipid-related pathways such as long-chain acyl-CoA synthetase, squalene synthase-like, and shaggy-related protein kinase showed significant correlations with digalactosyldiacylglycerol (DGDG) and diacylglycerol (DG). These correlations indicate potential coordination between lipid and protein abundance patterns, with several protein-lipid pairs showing strong associations that may reflect shared pathways or subcellular localisation.

Discussion

The genetic diversity among *Hevea* species is crucial for breeding and conservation, offering a reservoir of traits that could be harnessed to improve rubber yield, latex regeneration capacity, and physiological adaptability. However, the limited genetic information available for species other than *H. brasiliensis* poses challenges to fully understanding the evolutionary relationships and functional diversity within the genus. This highlights the importance of

identifying shared and species-specific genes across *Hevea* species to better explore and utilise the genetic potential of this economically significant genus.

A pangene analysis of *H. brasiliensis*, *H. guianensis*, *H. pauciflora, H. spruceana, H. collina*, and *H. confusa* revealed conservation of ATP binding and ATP hydrolysis activity functions within core and soft-core gene clusters. Simultaneously, gene family size evolution analysis showed expansion in ATP binding and ATP hydrolysis activity gene families in *Hevea*. In *H. brasiliensis*, ATP plays a crucial role in latex production by functioning as a physiological regulator directly in the metabolic pathways and indirectly through the H^+-ATPase activity. Efficient adenylate turnover supports latex regeneration and rubber yield by driving sucrose conversion to polyisoprene and regulating cytosolic pH [38]. While inorganic phosphorus is known indicator in latex diagnosis [39], ATP levels in latex also correlate strongly with rubber yield [40, 41], implicating ATP in yield-associated physiological processes. Further evidence supporting the role of ATP in isoprenoid biosynthesis is provided by *T. brevicorniculatum*, a rubber-producing species that serves as a suitable model for functional studies related to latex production. In this species, overexpression of ATP-dependent enzymes in the MVA pathway (acetyl-CoA acetyltransferase and ATP-citrate lyase) resulted in increased accumulation of isoprenoid end products [42]. This prior finding supports the notion that ATP-dependent steps influence isoprenoid flux, consistent with the hypothesis that expansion of ATP-related genes in *Hevea* reflects adaptation to the energetic demands of latex biosynthesis.

The taxonomy of *Hevea* species has long relied on morphological classification, which has not been updated for some time [3, 43]. Morphological approaches are limited by geographic accessibility and confounded by convergent evolution and phenotypic plasticity. Sampling of native *Hevea* species, aside from *H. brasiliensis*, has historically been confined to the Amazon basin [44]. In this study, genomic resources beyond *H. brasiliensis* provide a

more robust framework for delineating taxonomic relationships based on genetic relatedness. Phylogenomic analysis of single-copy orthologs revealed two distinct *Hevea* subgroups: one comprising *H. brasiliensis, H. pauciflora, H. confusa,* and *H. spruceana,* and the other containing *H. guianensis* and *H. collina*. The divergence between these subgroups, estimated at 6.46 mya, predates the introduction of *H. brasiliensis* into cultivation outside its native range. This divergence may reflect ecological differentiation, as *H. guianensis* typically grows at higher elevations, while the others thrive in lowland areas [44]. To investigate potential molecular adaptations to this environment, we annotated genes involved in the cold stress regulatory pathway, focusing on the inducer of CBF Expression (ICE)–C-repeat Binding Factor (CBF) transcriptional cascade (Supplementary Text S7). Comparative analysis revealed that *H. guianensis* possesses additional copies of *ICE* and *CBF* relative to *H. brasiliensis* (Supplementary Table S33). This gene expansion may reflect a genomic adaptation to cold stress, supporting the hypothesis that specific genetic changes underpin ecological differentiation.

Characterisation of the genetic basis of latex biosynthesis across rubber-producing plants suggests polyphyletic evolution, with convergent mechanisms and enzymes emerging independently in distinct clades [45, 46]. Our phylogenetic analyses of rubber biosynthesis genes in *Hevea* support this, showing *Hevea* CPT genes formed a distinct clade compared to other rubber-producing plants. Similarly, the *Hevea* CPTL and REF/SRPP genes clustered separately in their respective phylogenies. Comparative analyses of CPT and REF/SRPP gene counts indicated that the Euphorbiaceae including *Hevea*, have undergone CPT gene duplication, whereas REF/SRPP gene duplication occurred exclusively in *Hevea*. The *Hevea* CPT genes have expanded into two subclades, with CPT1-3 detected in the latex proteome, clustering within one subclade in the phylogeny. This observation suggests that the two *Hevea* CPT subclades may have diverged through neofunctionalization, with the CPT1-3 likely

playing a role in natural rubber metabolism. Similarly, *Hevea* REF/SRPP genes are separated into three subgroups, with REF1-3 and SRPP1-4 exclusively detected in the latex proteome. This phylogenetic divergence suggests functional specialisation driven by selection, with the latex-specific REF/SRPP genes adapted for rubber particle function.

Proteomic analysis provides complementary insights into metabolic activity by revealing the presence and relative abundance of proteins. In this study, latex proteomics across *Hevea* highlighted key enzymes involved in rubber biosynthesis, spanning IPP formation, rubber chain elongation, particle aggregation, and hormone signalling. The results confirmed the MVA pathway as the primary IPP source, FPS as the main trans-prenyltransferase, and CPT1-3, REF1-3, and SRPP1-4 as essential for latex synthesis. Hevamine/chitinase and β-1,3-glucanase are implicated in the rubber particle aggregation, while jasmonate signalling is activated in response to tapping. These proteins represent core rubber biosynthesis components across *Hevea* species. To explore the factors contributing to enhanced rubber biosynthesis in *H. brasiliensis*, differential proteomics analysis revealed several key enriched proteins. These include glutamine synthetase, whose overexpression in *A. thaliana* promotes nitrogen assimilation [47], with nitrogen content positively correlated with latex yield in *H. brasiliensis* [48], suggesting a regulatory link to rubber biosynthesis. Metallothionein, another top-enriched protein, is encoded by a gene induced by ethephon and has been associated with ROS scavenging in tapping panel dryness [49]. The bark storage protein, implicated in nitrogen cycling in *J. curcas* [50], is speculated to support nitrogen demands associated with latex regeneration. The eukaryotic translation initiation factor eIF5A, also known as the rubber biosynthesis stimulator protein, was also enriched and has been shown to promote IPP incorporation into rubber *in vitro*[51]. Given the continuous harvesting of latex and the resulting demand to replenish carbon, nitrogen, and energy reserves, the observed enrichment of proteins involved in nitrogen metabolism, ROS

scavenging, and protein synthesis could reflect an adaptive response supporting latex regeneration. These findings raise the hypothesis that the efficiency of rubber biosynthesis in *H. brasiliensis* may be influenced by the molecular capacity for latex regeneration. Among the 51 detected proteins linked to rubber biosynthesis, only SRPP2, MVK2, AACT4, and CTR1a were significantly enriched, suggesting that while the core biosynthetic pathway may be well conserved, latex production could be constrained by regeneration processes. Our proteomic data highlight the importance of latex renewal in sustaining high rubber yield and suggest that future improvements may benefit from targeting upstream physiological capacities rather than the biosynthetic steps alone.

Latex lipidomes further revealed distinct compositional features across *Hevea* species, with TG, AHexSIS, and PA identified as dominant lipid classes. TG functions as a lipid reservoir supporting regeneration [52], while AHexSIS modulates membrane fluidity [53], potentially influencing latex colloidal stability during storage or coagulation. PA, associated with membrane curvature and vesicle formation [54], may influence interparticle interaction during transport and processing. Latex from *H. brasiliensis* exhibited elevated levels of DG 36:1, DG 36:2, and PA 36:1 compared to other *Hevea* species. DG acts as a biosynthetic precursor and signalling molecule [55], and its enrichment suggests more active membrane turnover and stress adaptation. Likewise, higher PA levels may enhance colloidal stability and particle interactions during storage and processing. Since PA is also involved in plant stress signalling, particularly in response to wounding [56], its abundance may reflect an adaptive response to tapping-induced stress. Differences in rubber particle size between species [57] likely contribute to these compositional differences, affecting surface-area-dependent lipid interactions and processing behaviour.

In addition to compositional differences, correlation analysis between proteome and lipidome profiles provided insights into potential functional interactions. Notably, significant

positive correlations were observed between the mitochondrial phospholipid cardiolipin and and proteins of mitochondrial origin or function. For instance, NifU-like and succinate-CoA ligase, both implicated in mitochondrial iron-sulfur cluster assembly and energy metabolism [58, 59], showed robust correlations with cardiolipin. These associations reflect coordinated roles in mitochondrial activity in latex-producing cells. Additionally, proteins involved in lipid biosynthesis (long-chain acyl-CoA synthetase) [60], sterol and isoprenoid biosynthesis (squalene synthase-like) [61], and lipid-related signaling (shaggy-related protein kinase alpha) [62] showed significant correlations with glycerolipids, including diacylglycerol and digalactosyldiacylglycerol. These associations raise the possibility of coordinated roles in lipid remodeling, membrane biogenesis, or signaling processes that may contribute to rubber particle formation or function.

Together, the distinct lipid profiles of *Hevea*, alongside its protein–lipid interactions, may underpin adaptive metabolic features that support rubber stability, membrane integrity, and flow resilience, traits crucial for high-quality rubber production.

This study demonstrates how comparative multi-omics can be applied to investigate the biological basis of agronomic traits such as latex production in *Hevea* species. By integrating genomic, proteomic, and lipidomic data, we highlight the genetic and biochemical diversity among *Hevea* and speculate on molecular features that may contribute to rubber yield and latex regeneration. Our integrative analyses support the hypothesis that latex productivity and quality in *Hevea* may be influenced by a combination of factors, including an expanded ATP-related functions, differential protein abundance related to nitrogen balance, ROS homeostasis, and protein synthesis, as well as distinct lipid profiles associated with membrane remodelling and rubber particle stability. These insights open new avenues for understanding species-specific adaptations and lay the groundwork for targeted functional

studies. Importantly, our findings provide a foundational resource to inform future research and breeding strategies aimed at improving rubber production.

Methods

DNA extraction and genome sequencing

The sequenced individuals of *H. brasiliensis* (NCBI:txid605986)*, H. guianensis* (NCBI:txid882917)*, H. pauciflora* (NCBI:txid1318717)*, H. spruceana* (NCBI:txid1318718)*, H. collina*, and *H. confusa* were maintained at the Rubber Research Institute of Indonesia (Supplementary Text S1). High molecular weight genomic DNA was extracted from young leaves of *H. brasiliensis* using the Genomic-tip 100/G kit (Qiagen, Germany). A PacBio library was prepared using the SMRTbell Express Template Prep Kit 2.0 (Pacific Biosciences, USA) according to the manufacturer's instructions. Sequencing was performed on PacBio Sequel II with a SMRT Cell 8M. Raw PacBio subreads were obtained from the SMRT Link v9.0 pipeline (RRID:SCR_002942). For Hi-C sequencing of *H. brasiliensis*, fresh young leaves were ground in liquid nitrogen and fixed with formaldehyde. The Hi-C library was constructed using the Arima Hi-C kit with DpnII and HinfI restriction enzymes, following Kadota et al. [63] (Supplementary Text S2). The library was sequenced on a HiSeq X Ten (Illumina, USA). For short-read sequencing of *H. guianensis, H. pauciflora, H. spruceana, H. collina*, and *H. confusa*, genomic DNA was extracted from leaf tissues using a DNeasy Plant Mini Kit (Qiagen). Sequencing libraries were prepared using the TruSeq DNA Sample Preparation Kit (Illumina) and sequenced on a DNBSEQ platform at BGI-Shenzen (BGI Co. Ltd., China).

RNA sequencing

Total RNA was extracted from the bark, latex, leaf and petiole of *H. brasiliensis* following the method previously published [64], using a CTAB buffer and lithium chloride precipitation.

RNA was quantified using a NanoDrop spectrophotometer (ThermoFisher Scientific, USA) and assessed with a Bioanalyzer 2100 (Agilent Technologies, USA). For Iso-seq, cDNA synthesis was performed using the SMARTer PCR cDNA Synthesis Kit (Clontech, Japan). Size fractionation and selection were carried out using the BluePippin Size Selection System (Sage Science, USA). SMRT libraries were prepared using the SMRTbell Express Template Prep Kit 2.0 (Pacific Biosciences) and sequenced on the PacBio Sequel II. Iso-Seq data were analysed using SMRT Link (v9.0), which incorporates read quality filtering, read clustering, consensus calling, and Quiver polishing steps to assemble the Iso-Seq reads into high quality, full-length transcripts (Supplementary Text S3).

Genome assembly and annotation

For *H. brasiliensis,* the long-read assembler Flye (v2.8.2) (RRID:SCR_017016) [65] was used for the assembly and polishing of PacBio reads with the parameter -pacbio-raw. The raw Hi-C reads were adapter and quality trimmed using TrimGalore (v0.6.0). The clean Hi-C reads were mapped to the PacBio assembly using Juicer pipeline (v1.6) (RRID:SCR_017226) [66]. Scaffolds were grouped, ordered, and orientated into pseudo-chromosomes using 3d-dna (v.180922) (RRID:SCR_017227) with parameters i=10000, and r=4. For *H. guianensis, H. pauciflora, H. spruceana, H. collina*, and *H. confusa*, Illumina reads were trimmed using Platanus_trim (v1.0.7) and assembled using Platanus (v1.2.4) [67]. The Platanus assemblies were synteny scaffolded with RagTag [68] (v2.1.0) using *H. brasiliensis* as reference.

Genome size was estimated based on *k*-mer counting using Jellyfish (v2.3.0) (RRID:SCR_005491). For *H. brasiliensis*, previously sequenced Illumina paired-end reads [22] were used, while for *H. guianensis*, *H. pauciflora*, *H. spruceana*, *H. collina*, and *H. confusa*, short reads generated in this study were analysed. Genome size was estimated based on 25 *k*-mer with GenomeScope 2.0. Completeness of the genome assembly was assessed using BUSCO (v5) (RRID:SCR_015008) [69] against the Embryophyta odb10 database.

Telomeric and centromeric sequences in *H. brasiliensis* chromosomes were analysed using quarTeT (v1.2.5). Within quarTeT, the TeloExplorer module, which utilises the Telomere Identification Toolkit (tidk), was used for telomere detection. Meanwhile, the CentroMiner module incorporated transposable element annotations from the Extensive De Novo TE Annotator (EDTA) as a complementary approach for centromere identification.

Repetitive elements in the *Hevea* species genome were identified by RepeatMasker (v4.1.0) (RRID:SCR_012954) [70] using the *de novo* repeat library and known Viridiplantae repetitive sequences in Dfam and RepBase. The *de novo* repeat library was constructed using RepeatModeler, integrating RECON (v1.0.8), RepeatScout (v1.0.6), and Tandem Repeat Finder (v4.0.9). Genome annotation was performed using the MAKER (v3.01.03) (RRID:SCR_005309) [71] pipeline with *ab initio* gene predictions Augustus (v3.2.2), Snap (v2013-11-29), GeneMark-ES (v3.61), and Fgenesh (v8.0.0b). Iso-Seq dataset and assembled transcripts from previously generated RNA-seq [72] were input to MAKER as expressed sequence tag evidence. The functional annotation of the protein-coding genes against NCBI NR, SwissProt, TrEMBL and KEGG was performed with BLASTP at an *E*-value of $1e^{-5}$. GO terms and InterPro entries were assigned via OmicsBox (v3.1.9) and InterProScan (v5.52-86.0). Non-coding RNAs were annotated using INFERNAL (v1.1.4) against the Rfam database.

Clustering of pangene

Pangene across *Hevea*, including *H. brasiliensis, H. guianensis, H. spruceana, H. collina*, and *H. confusa* were analysed using GET_HOMOLOGUES-EST (v3.6.2) (RRID:SCR_015705) [73]. All-against-all comparisons were performed using BLAST, followed by clustering with the OrthoMCL algorithm at an inflation value of 1.5. Genes were clustered into core, soft-core, shell and cloud clusters based on orthogroup frequency. GO enrichment analysis for

genes in each cluster was performed using the OmicsBox. The curves describing pangene and core gene sizes were fitted to the Tettelin model.

Construction of gene families

We downloaded the protein sequences of *A. thaliana, L. usitatissimum, M. esculenta, O. sativa, P. trichocarpa, R. communis*, and *S. purpurea* from Phytozome 13, and the protein sequences of *J. curcas* from Ensembl Plants. Gene families based on all-against-all BLASTP alignment among the 13 plant species were constructed using OrthoFinder (v2.5.5) (RRID:SCR_017118) [74]. Single-copy orthologs for each species within a given orthogroup as analysed by OrthoFinder were aligned using MAFFT (v7.520), and the resulting alignments were concatenated to create a super alignment matrix. Phylogenetic analysis on the concatenated alignment was conducted using IQ-TREE (v2.2.6) (RRID:SCR_017254) [75] with maximum likelihood and 1000 ultrafast bootstraps, employing the JTTF+I+R6 model selected by ModelFinder according to the Bayesian Information Criterion. The phylogenetic tree was visualised using FigTree (v1.4.5). The divergence times in the phylogenetic tree were inferred using the least square dating method implemented in IQ-TREE. For divergence time estimation, we calibrated the model using the divergence times between *A. thaliana* and *H. brasiliensis* (108 Mya), *L. usitatissimum* and *H. brasiliensis* (88 Mya), and *P. trichocarpa* and *H. brasiliensis* (72 Mya) obtained from the TimeTree database [76].

Gene family size evolution

The gene family expansion and contraction across 13 plant species were inferred using CAFE5 (RRID:SCR_018924) [77], with the phylogenetic tree constructed by IQ-TREE based on single-copy orthologs as the input tree. The separate birth and death rates of gene families across the phylogeny were estimated using maximum likelihood, and the *P*-value threshold of

0.01 was applied to identify significantly expanded or contracted gene families for a given species. These expanded and contracted gene families were subjected to enrichment analysis using OmicsBox with Fisher's exact test.

Genome duplication analysis

The genome-wide duplications in the *Hevea* species genomes were analysed with Tree2GD (v1.0.40) [78]. All-against-all protein alignments were performed with Diamond (v2.1.9), and hierarchical orthogroups were predicted with PhyloMCL (v2.0). Gene trees were reconciled with a reference species tree using the default parameters in Tree2GD. Paralogous gene pairs for each species were identified, and their coding sequences were aligned using MUSCLE (v3.8.31) and pal2nal (v13). The K_s values of the gene pairs were calculated using KaKs_calculator (v2.0), and the 4DTv values were determined using the Perl script Calculate_4DTV_correction.pl from Github. The genome duplication events times of the *Hevea* species were estimated using the formula $T = K_s/2r$, where the rate of synonymous substitutions per site per year (r) is 7.5×10^{-9}.

Proteome characterisation

Proteins from the latex samples were extracted as detailed in Supplementary Text S6 and according to the protocol available at protocols.io [79]. Three biological replicates were collected and analysed for each sample. The peptides were injected onto a 75 µm × 120 mm nanoLC column (Nikkyo Technos) at 50 °C and then separated with a gradient (A = 0.1% formic acid (FA) in water, B = 0.1% FA in 80% acetonitrile) consisting of 0-50 min 8% B, 50-57 min 36% B, 57-60 min 70% B using an UltiMate 3000 RSLCnano LC system (Thermo Fisher Scientific). The eluted peptides were analysed on an Orbitrap Exploris 480 (Thermo Fisher Scientific) operated in positive ion mode with data-independent acquisition (DIA-MS). MS1 spectra were collected in the range of 495 to 745 m/z at a 15,000 resolution, and MS2

spectra were collected in the range of 200 m/z and above at a 30,000 resolution with normalised collision energy at 26%. Data were processed using DIA-NN (v1.8.1) (RRID:SCR_022865) [80] for protein and peptide identification and quantification. A spectral library was generated using deep learning-based spectral prediction, and the MS/MS data were searched against the *H. brasiliensis* annotated protein sequences. Parameters were set as follows: enzyme, trypsin; maximum missed cleavage site, 1; static modification, carbamidomethylation; and FDR thresholds for precursor and protein identification, 1%.

Lipid extraction and LC-MS/MS analysis

Latex samples for lipidomic analysis were collected following the same method as for proteomic analysis (Supplementary Text S1), with three biological replicates per sample. To simulate real field-to-factory conditions, samples were transported without chemical preservation prior to lipid extraction and LC-MS/MS analysis.

In previous study, phospholipase A inhibitors such as manoalide, ONO (2-[*p*-amylcinnamoyl]amino-4-chlorobenzoic acid), BEL (bromoenol lactone), and AACOCF3 (trifluoromethyl ketone), along with Triton X-100 were used to prevent lipid degradation [37]. In comparison, our sampling strategy aimed to capture a lipid profile representative of the natural processing timeline, which may differ from profiles obtained under controlled laboratory conditions.

100 µL of chloroform was added to 1 mg of latex samples, followed by shaking, sonication, and centrifugation. Methanol was then added to 50 µL of the supernatant, stirred, and centrifuged again, and the supernatant was collected. Lysophosphatidylcholine (18:1-d7) was added as an internal standard at a final concentration of 500 ng/mL, and the sample was analysed using LC-MS/MS. Operational blanks (prepared without samples) and quality control samples (prepared by equally mixing aliquots from each sample solution) were analysed in the same manner. LC-MS analysis was performed using an UltiMate 3000 BioRS

system coupled to an LTQ Orbitrap XL mass spectrometer (Thermo Fisher Scientific). Chromatographic separation was achieved using an L-column3 C18 metal-free column (2.0 mm I.D. × 100 mm, 2 μm particle size; CERI). Data analysis, including peak detection, lipid species estimation within each class, and sample alignment, was conducted using MS-DIAL (v4.80). Lipid peaks were considered detected if they met the following criteria: peak height in QC samples ≥10,000; peak area at least twice that of the operational blank; average signal-to-noise ratio ≥3; and consistent detection across all QC measurements with a coefficient of variation <20%. The lipid content was determined relative to an internal standard by comparing the analyte peak area. OPLS-DA was performed using the R package ropls (v1.39.0) (RRID:SCR_016888) to estimate lipid differences between *H. brasiliensis* and other *Hevea* species. Lipid abundances were transformed using $\log_{10}(x+1)$ to reduce data skewness and accommodate zero values. Variable importance in projection (VIP) scores were obtained from ropls, and *p*-values were calculated using a two-sample t-test. Lipids with $p < 0.05$ and VIP > 1.0 were considered differentially abundant.

Abbreviations

AACT: acetyl-CoA C-acetyltransferase; ADGGA: acyl diacylglyceryl glucuronide; AHexSIS: acylhexosyl sitosterol; AHexSTS: acylhexosyl stigmasterol; Cer_AP: ceramide alpha-hydroxy fatty acid-phytospingosine; CL: cardiolipin; CME: 4-(cytidine 5′-diphospho)-2-C-methyl-D-erythritol; CMEC: 2-C-methyl-D-erythritol-2,4-cyclodiphosphate; CMK: 2-C-methyl-d-erythritol 4-phosphate kinase; CMS: 2-C-methyl-D-erythritol 4-phosphate cytidylyltransferase; COI1: coronatine-insensitive 1; CPT: cis-prenyltransferase; CPTL: CPT-like; CTR1: constitutive triple response 1; DG: diacylglycerol; DGDG: digalactosyldiacylglycerol; DGGA: diacylglyceryl glucuronide; DMAPP: dimethylallyl pyrophosphate; DPMD: diphosphomevalonate decarboxylase; DXP: 1-deoxy-d-xylulose 5-

phosphate; DXR: DXP reductoisomerase; DXS: 1-deoxy-d-xylulose 5-phosphate synthase; EIL1: ethylene insensitive-like 1; EIN2/3: ethylene-insensitive 2/3; ETR: ethylene receptors; FPS: farnesyl pyrophosphate synthase; G3P: glyceraldehyde 3-phosphate; GGPP: geranylgeranyl pyrophosphate; GGPS: geranylgeranyl pyrophosphate synthase; GPP: geranyl pyrophosphate; GPS: geranyl pyrophosphate synthase; HDR: 4-hydroxy-3-methylbut-2-enyl diphosphate reductase; HDS: 4-hydroxy-3-methylbut-2-enyl-diphosphate synthase; HMBD: 1-hydroxy-2-methyl-2-butenyl 4-diphosphate; HMGR: hydroxymethylglutaryl-CoA reductase; HMGS: hydroxymethylglutaryl-CoA synthase; IPI: isopentenyl diphosphate isomerase; IPP: isopentenyl diphosphate; JAZ: jasmonate ZIM-domain; MCS: 2-C-methyl-D-erythritol 2,4-cyclodiphosphate synthase; MEP: 2-C-methyl-d-erythritol 4-phosphate; MVK: mevalonate kinase; MYC: MYC transcription factor; MVA-5-p: mevalonate-5-phopshate; MVA-5-pp: MVA 5-diphosphate; OxFA: oxidized fatty acid; PA: phosphatidic acid; PCME: 2-phospho-4-(cytidine 5′-diphospho)-2-C-methyl-D-erythritol; PI: phosphatidylinositol; PMK: phospho-MVA kinase; REF: rubber elongation factor; SRPP: small rubber particle protein; ST: sulfatide; TG: triacylglycerol.

Acknowledgements

We thank Dr. Thomas Wijaya (IRRI, Indonesia), Marliana Sheny (JICA) and Satoru Mitani (JICA) for coordination of our international research. We thank Kapus Suroso Rahutomo for access to Natural rubber plantation. This research was partially supported by the Science and Technology Research Partnership for Sustainable Development (SATREPS).

Author contributions

N-S.L., E.O-K., Y.M., A.S.O. and M.M. designed the experiments and summarised the data. N-S.L. and Y.M. performed bioinformatic analysis of genomic, transcriptomic, proteomic and lipidomic data. Sampling and material preparation were done by F.O., T. K., E.O-K. and H.H.

M.K., O.N. and S.K. performed Hi-C genome sequencing and assembly. N-S.L. and M.M. wrote the manuscript.

Data Availability

The data have been submitted under BioProject accession PRJNA1180688. A previously published RNA-seq dataset used in this study is available under BioProject accession PRJDB4387. Genome data are available in the DDBJ/EMBL/GenBank under accession numbers SRR31189420–SRR31189422 (*H. brasiliensis*), SRR31191093 (*H. guianensis*), SRR31191092 (*H. collina*), SRR31192722 (*H. pauciflora*), SRR31192721 (*H. confusa*), and SRR31192751 (*H. spruceana*). Iso-Seq data are available under accession numbers SRR31201725 (latex), SRR31201726 (leaf), SRR31203224 (petiole), and SRR31203225 (bark). Proteomic data have been deposited in the ProteomeXchange repository under the accession number PXD057483. Lipidomic data have been submitted to the Metabolomics Workbench under accession ST003958. All additional supporting data are available in the *GigaScience* repository, GigaDB [81].

Competing interests

The authors declare that they have no competing interests.

References

1. Metcalfe CR. Distribution of latex in the plant kingdom. Econ Bot. 1967;21:115-27. https://doi.org/10.1007/BF02897859.
2. van Beilen JB and Poirier Y. Establishment of new crops for the production of natural rubber. Trends Biotechnol. 2007;25:522-9. https://doi.org/10.1016/j.tibtech.2007.08.009.
3. Gonçalves PdS, Cardoso M and Ortolani A. Origin, variability and domestication of *Hevea*-a review. Pesq Agropec Brasileira. 1990;25:135–56.

4. Priyadarshan P and Goncalves PdS. Use of *Hevea* gene pool in rubber tree (*Hevea brasiliensis* Muell.-Arg) breeding. Planter. 2002;78:123-38.

5. Priyadarshan PM and Clément-Demange A. Breeding *Hevea* rubber: formal and molecular genetics. Adv Genet. 2004;52:51-115. https://doi.org/10.1016/s0065-2660(04)52003-5.

6. Cornish K, Wood DF and Windle JJ. Rubber particles from four different species, examined by transmission electron microscopy and electron-paramagnetic-resonance spin labeling, are found to consist of a homogeneous rubber core enclosed by a contiguous, monolayer biomembrane. Planta. 1999;210:85-96. https://doi.org/10.1007/s004250050657.

7. Takahashi S and Koyama T. Structure and function of *cis*-prenyl chain elongating enzymes. Chem Rec. 2006;6:194-205. https://doi.org/10.1002/tcr.20083.

8. Lakusta AM, Kwon M, Kwon EG, Stonebloom S, Scheller HV and Ro DK. Molecular studies of the protein complexes involving *cis*-prenyltransferase in guayule (*Parthenium argentatum*), an alternative rubber-producing plant. Front Plant Sci. 2019;10:165. https://doi.org/10.3389/fpls.2019.00165.

9. Yamashita S, Yamaguchi H, Waki T, Aoki Y, Mizuno M, Yanbe F, et al. Identification and reconstitution of the rubber biosynthetic machinery on rubber particles from *Hevea brasiliensis*. Elife. 2016;5. https://doi.org/10.7554/eLife.19022.

10. Chow K-S, Mat-Isa MN, Bahari A, Ghazali A-K, Alias H, Mohd.-Zainuddin Z, et al. Metabolic routes affecting rubber biosynthesis in *Hevea brasiliensis* latex. J Exp Bot. 2012;63:1863-71. https://doi.org/10.1093/jxb/err363.

11. Dennis MS and Light DR. Rubber elongation factor from *Hevea brasiliensis*. Identification, characterization, and role in rubber biosynthesis. J Biol Chem. 1989;264:18608-17.

12. Berthelot K, Lecomte S, Estevez Y and Peruch F. *Hevea brasiliensis* REF (Hev b 1) and SRPP (Hev b 3): An overview on rubber particle proteins. Biochimie. 2014;106:1-9. https://doi.org/10.1016/j.biochi.2014.07.002.

13. Wang X, Shi M, Wang D, Chen Y, Cai F, Zhang S, et al. Comparative proteomics of primary and secondary lutoids reveals that chitinase and glucanase play a crucial combined role in rubber particle aggregation in *Hevea brasiliensis*. J Proteome Res. 2013;12:5146-59. https://doi.org/10.1021/pr400378c.

14. Deng X, Guo D, Yang S, Shi M, Chao J, Li H, et al. Jasmonate signalling in the regulation of rubber biosynthesis in laticifer cells of rubber tree, *Hevea brasiliensis*. J Exp Bot. 2018;69:3559-71. https://doi.org/10.1093/jxb/ery169.

15. Liu JP, Zhuang YF, Guo XL and Li YJ. Molecular mechanism of ethylene stimulation of latex yield in rubber tree (*Hevea brasiliensis*) revealed by de novo sequencing and transcriptome analysis. BMC Genomics. 2016;17:257. https://doi.org/10.1186/s12864-016-2587-4.

16. Chao J, Wu S, Shi M, Xu X, Gao Q, Du H, et al. Genomic insight into domestication of rubber tree. Nat Commun. 2023;14:4651. https://doi.org/10.1038/s41467-023-40304-y.

17. Cheng H, Song X, Hu Y, Wu T, Yang Q, An Z, et al. Chromosome-level wild *Hevea brasiliensis* genome provides new tools for genomic-assisted breeding and valuable loci to elevate rubber yield. Plant Biotechnol J. 2023;21:1058-72. https://doi.org/10.1111/pbi.14018.

18. Pootakham W, Sonthirod C, Naktang C, Ruang-Areerate P, Yoocha T, Sangsrakru D, et al. *De novo* hybrid assembly of the rubber tree genome reveals evidence of paleotetraploidy in *Hevea* species. Sci Rep. 2017;7:41457. https://doi.org/10.1038/srep41457.

19. Rahman AY, Usharraj AO, Misra BB, Thottathil GP, Jayasekaran K, Feng Y, et al. Draft genome sequence of the rubber tree *Hevea brasiliensis*. BMC Genomics. 2013;14:75. https://doi.org/10.1186/1471-2164-14-75.

20. Tang C, Yang M, Fang Y, Luo Y, Gao S, Xiao X, et al. The rubber tree genome reveals new insights into rubber production and species adaptation. Nat Plants. 2016;2:16073. https://doi.org/10.1038/nplants.2016.73.

21. Fang Y, Xiao X, Lin J, Lin Q, Wang J, Liu K, et al. Pan-genome and phylogenomic analyses highlight *Hevea* species delineation and rubber trait evolution. Nat Commun. 2024;15:7232. https://doi.org/10.1038/s41467-024-51031-3.

22. Lau NS, Makita Y, Kawashima M, Taylor TD, Kondo S, Othman AS, et al. The rubber tree genome shows expansion of gene family associated with rubber biosynthesis. Sci Rep. 2016;6:28594. https://doi.org/10.1038/srep28594.

23. Liu J, Shi C, Shi CC, Li W, Zhang QJ, Zhang Y, et al. The chromosome-based rubber tree genome provides new insights into spurge genome evolution and rubber biosynthesis. Mol Plant. 2020;13:336-50. https://doi.org/10.1016/j.molp.2019.10.017.

24. Cornish K. Similarities and differences in rubber biochemistry among plant species. Phytochemistry. 2001;57:1123-34. https://doi.org/https://doi.org/10.1016/S0031-9422(01)00097-8.

25. Asawatreratanakul K, Zhang YW, Wititsuwannakul D, Wititsuwannakul R, Takahashi S, Rattanapittayaporn A, et al. Molecular cloning, expression and characterization of cDNA encoding cis-prenyltransferases from *Hevea brasiliensis*. A key factor participating in natural rubber biosynthesis. Eur J Biochem. 2003;270:4671-80. https://doi.org/10.1046/j.1432-1033.2003.03863.x.

26. Epping J, van Deenen N, Niephaus E, Stolze A, Fricke J, Huber C, et al. A rubber transferase activator is necessary for natural rubber biosynthesis in dandelion. Nat Plants. 2015;1:15048. https://doi.org/10.1038/nplants.2015.48.

27. Niephaus E, Müller B, van Deenen N, Lassowskat I, Bonin M, Finkemeier I, et al. Uncovering mechanisms of rubber biosynthesis in *Taraxacum koksaghyz* - role of cis-prenyltransferase-like 1 protein. Plant J. 2019;100:591-609. https://doi.org/10.1111/tpj.14471.

28. Oh SK, Kang H, Shin DH, Yang J, Chow KS, Yeang HY, et al. Isolation, characterization, and functional analysis of a novel cDNA clone encoding a small rubber particle protein from *Hevea brasiliensis*. J Biol Chem. 1999;274:17132-8. https://doi.org/10.1074/jbc.274.24.17132.

29. Chao J, Chen Y, Wu S and Tian W-M. Comparative transcriptome analysis of latex from rubber tree clone CATAS8-79 and PR107 reveals new cues for the regulation of latex regeneration and duration of latex flow. BMC Plant Biol. 2015;15:104. https://doi.org/10.1186/s12870-015-0488-3.

30. Florez-Velasco N, Ramos VF, Magnitskiy S and Balaguera-López H. Ethylene and jasmonate as stimulants of latex yield in rubber trees (*Hevea brasiliensis*): Molecular and physiological mechanisms. A systematic approximation review. Advanced Agrochem. 2024;3:279-88. https://doi.org/https://doi.org/10.1016/j.aac.2024.07.003.

31. Pujade-Renaud V, Clement A, Perrot-Rechenmann C, Prevot JC, Chrestin H, Jacob JL, et al. Ethylene-induced increase in glutamine synthetase activity and mRNA levels in *Hevea brasiliensis* latex cells. Plant Physiol. 1994;105:127-32. https://doi.org/10.1104/pp.105.1.127.

32. Zhang Y, Leclercq J and Montoro P. Reactive oxygen species in *Hevea brasiliensis* latex and relevance to Tapping Panel Dryness. Tree Physiol. 2016;37:261-9. https://doi.org/10.1093/treephys/tpw106.

33. Huang Y, Fang Y, Long X, Liu L, Wang J, Zhu J, et al. Characterization of the rubber tree metallothionein family reveals a role in mitigating the effects of reactive oxygen species associated with physiological stress. Tree Physiol. 2018;38:911-24. https://doi.org/10.1093/treephys/tpy003.

34. Asghari Barzegar Z, Taghvaei Ganjali S, Malekzadeh M and Motiee F. Correlations between lipid contents of natural rubber and tensile properties of natural rubber-based compound, using attenuated total reflection Fourier transform infrared spectroscopy. Spectrosc Lett. 2023;56:1-13. https://doi.org/10.1080/00387010.2022.2153143.

35. Yu H, Wang Q, Li J, Liu Y, He D, Gao X, et al. Effect of lipids on the stability of natural rubber latex and tensile properties of its films. J Rubber Res. 2017;20:213-22. https://doi.org/10.1007/BF03449153.

36. Zhang BL, Huang HH, Wang YZ, Ding L and Liang Y. Study on molecular structure and property of highly purified natural rubber. J Anal Appl Pyrol. 2018;134:130-5. https://doi.org/10.1016/j.jaap.2018.05.018.

37. Bae SW, Jung S, Choi SC, Kim MY and Ryu SB. Lipid composition of latex and rubber particles in *Hevea brasiliensis* and *Taraxacum kok-saghyz*. Molecules. 2020;25 https://doi.org/10.3390/molecules25215110.

38. d'Auzac J, Jacob JL and Chrestin H. Physiology of rubber tree latex. Boca Raton, FL: CRC Press; 1989.

39. Chotiphan R, Vaysse L, Lacote R, Gohet E, Thaler P, Sajjaphan K, et al. Can fertilization be a driver of rubber plantation intensification? Ind Crop Prod. 2019;141:111813. https://doi.org/10.1016/j.indcrop.2019.111813.

40. Sreelatha S, Simon SP and Jacob JJJoRR. On the possibility of using ATP concentration in latex as an indicator of high yield in *Hevea brasiliensis*. J Rubb Res. 2004;7:71-8.

41. Sreelatha S, Jacob J, Mercykutty VC, Simon SP, Krishnakumar R and Annamalainathan K. ATP concentration in latex as an indicator for early evaluation of yield in *Hevea brasiliensis*. J Plant Crops. 2014;42:48-53.

42. Pütter KM, van Deenen N, Unland K, Prüfer D and Schulze Gronover C. Isoprenoid biosynthesis in dandelion latex is enhanced by the overexpression of three key enzymes involved in the mevalonate pathway. BMC Plant Biol. 2017;17:88. https://doi.org/10.1186/s12870-017-1036-0.

43. Schultes RE. A brief taxonomic view of the genus *Hevea*. Kuala Lumpur: Malaysian Rubber Research and Development Board; 1990.

44. Priyadarshan PM. Genetic resources. Biology of *Hevea* rubber. Springer Cham; 2017. p. 83-105.

45. Lin T, Xu X, Ruan J, Liu S, Wu S, Shao X, et al. Genome analysis of *Taraxacum kok-saghyz* Rodin provides new insights into rubber biosynthesis. Natl Sci Rev. 2018;5:78-87. https://doi.org/10.1093/nsr/nwx101.

46. Wuyun TN, Wang L, Liu H, Wang X, Zhang L, Bennetzen JL, et al. The hardy rubber tree genome provides insights into the evolution of polyisoprene biosynthesis. Mol Plant. 2018;11:429-42. https://doi.org/10.1016/j.molp.2017.11.014.

47. Zhu C, Zhang G, Shen C, Chen S, Tang Y, Mei B, et al. Expression of bacterial glutamine synthetase gene in *Arabidopsis thaliana* increases the plant biomass and level of nitrogen utilization. Biologia. 2015;70:1586-96. https://doi.org/10.1515/biolog-2015-0183.

48. An F, Xie G, Jiang J, Lin W, Xie Y, Pan Y, et al. Nutrient conditions of rubber olantations and their relationship with latex yield under stimulated tapping system in Xishuangbanna. Chin J Trop Crop. 2005;26:1-6.

49. Zhu J, Zhang Q, Wu R and Zhang Z. *HbMT2*, an ethephon-induced metallothionein gene from *Hevea brasiliensis* responds to H_2O_2 stress. Plant Physiol Biochem. 2010;48:710-5. https://doi.org/10.1016/j.plaphy.2010.04.004.

50. Zhang MJ, Fu Q, Chen MS, He H, Tang M, Ni J, et al. Characterization of the bark storage protein gene (*JcBSP*) family in the perennial woody plant *Jatropha curcas* and the function of *JcBSP1* in *Arabidopsis thaliana*. PeerJ. 2022;10:e12938. https://doi.org/10.7717/peerj.12938.

51. Yusof F, Chow K-S, Ward MA and Walker JMJJoRR. A stimulator protein of rubber biosynthesis from *Hevea brasiliensis* latex. J Rubber Res. 2000;3:232-49.

52. Yang Y and Benning C. Functions of triacylglycerols during plant development and stress. Curr Opin Biotechnol. 2018;49:191-8. https://doi.org/10.1016/j.copbio.2017.09.003.

53. Dufourc EJ. Sterols and membrane dynamics. J Chem Biol. 2008;1:63-77. https://doi.org/10.1007/s12154-008-0010-6.

54. Kooijman EE, Chupin V, de Kruijff B and Burger KNJ. Modulation of membrane curvature by phosphatidic acid and lysophosphatidic acid. Traffic. 2003;4:162-74. https://doi.org/10.1034/j.1600-0854.2003.00086.x.

55. Carrasco S and Mérida I. Diacylglycerol, when simplicity becomes complex. Trends Biochem Sci. 2007;32:27-36.

56. Testerink C and Munnik T. Phosphatidic acid: a multifunctional stress signaling lipid in plants. Trends Plant Sci. 2005;10:368-75.

57. Ong CW and Shamsul Bahri AR. Rubber particles: Size, molecular weight and their distributions detected in wild *Hevea* Species. J Biol Agric Healthc. 2016;6:98-103.

58. Couturier J, Touraine B, Briat JF, Gaymard F and Rouhier N. The iron-sulfur cluster assembly machineries in plants: current knowledge and open questions. Front Plant Sci. 2013;4:259. https://doi.org/10.3389/fpls.2013.00259.

59. Zhang Y and Fernie AR. The role of TCA cycle enzymes in plants. Adv Biol. 2023;7:e2200238. https://doi.org/10.1002/adbi.202200238.

60. Zhao H, Kosma DK and Lü S. Functional role of long-chain acyl-coA synthetases in plant development and stress responses. Front Plant Sci. 2021;12:640996. https://doi.org/10.3389/fpls.2021.640996.

61. Unland K, Pütter KM, Vorwerk K, van Deenen N, Twyman RM, Prüfer D, et al. Functional characterization of squalene synthase and squalene epoxidase in *Taraxacum koksaghyz*. Plant Direct. 2018;2:e00063. https://doi.org/10.1002/pld3.63.

62. Dornelas MC, Van Lammeren AA and Kreis M. *Arabidopsis thaliana* SHAGGY-related protein kinases (AtSK11 and 12) function in perianth and gynoecium development. Plant J. 2000;21:419-29. https://doi.org/10.1046/j.1365-313x.2000.00691.x.

63. Kadota M, Nishimura O, Miura H, Tanaka K, Hiratani I and Kuraku S. Multifaceted Hi-C benchmarking: what makes a difference in chromosome-scale genome scaffolding? Gigascience. 2020;9. https://doi.org/10.1093/gigascience/giz158.

64. Deng LH, Luo MW, Zhang CF and Zeng HC. Extraction of high-quality RNA from rubber tree leaves. Biosci Biotechnol Biochem. 2012;76:1394-6. https://doi.org/10.1271/bbb.120014.

65. Kolmogorov M, Yuan J, Lin Y and Pevzner PA. Assembly of long, error-prone reads using repeat graphs. Nat Biotechnol. 2019;37:540-6. https://doi.org/10.1038/s41587-019-0072-8.

66. Durand NC, Shamim MS, Machol I, Rao SS, Huntley MH, Lander ES, et al. Juicer provides a one-click system for analyzing loop-resolution Hi-C experiments. Cell Syst. 2016;3:95-8. https://doi.org/10.1016/j.cels.2016.07.002.

67. Kajitani R, Toshimoto K, Noguchi H, Toyoda A, Ogura Y, Okuno M, et al. Efficient de novo assembly of highly heterozygous genomes from whole-genome shotgun short reads. Genome Res. 2014;24:1384-95. https://doi.org/10.1101/gr.170720.113.

68. Alonge M, Lebeigle L, Kirsche M, Jenike K, Ou S, Aganezov S, et al. Automated assembly scaffolding using RagTag elevates a new tomato system for high-throughput genome editing. Genome Biol. 2022;23:258. https://doi.org/10.1186/s13059-022-02823-7.

69. Manni M, Berkeley MR, Seppey M, Simão FA and Zdobnov EM. BUSCO update: Novel and streamlined workflows along with broader and deeper phylogenetic coverage for scoring of eukaryotic, prokaryotic, and viral genomes. Mol Biol Evol. 2021;38:4647-54. https://doi.org/10.1093/molbev/msab199.

70. Tarailo-Graovac M and Chen N. Using RepeatMasker to identify repetitive elements in genomic sequences. Curr Protoc Bioinformatics. 2009;25:Unit 4.10. https://doi.org/10.1002/0471250953.bi0410s25.

71. Campbell MS, Law M, Holt C, Stein JC, Moghe GD, Hufnagel DE, et al. MAKER-P: a tool kit for the rapid creation, management, and quality control of plant genome annotations. Plant Physiol. 2014;164:513-24. https://doi.org/10.1104/pp.113.230144.

72. Makita Y, Ng KK, Veera Singham G, Kawashima M, Hirakawa H, Sato S, et al. Large-scale collection of full-length cDNA and transcriptome analysis in *Hevea brasiliensis*. DNA Res. 2017;24:159-67. https://doi.org/10.1093/dnares/dsw056.

73. Contreras-Moreira B, Cantalapiedra CP, García-Pereira MJ, Gordon SP, Vogel JP, Igartua E, et al. Analysis of plant pan-genomes and transcriptomes with GET_HOMOLOGUES-EST, a clustering solution for sequences of the same species. Front Plant Sci. 2017;8:184. https://doi.org/10.3389/fpls.2017.00184.

74. Emms DM and Kelly S. OrthoFinder: phylogenetic orthology inference for comparative genomics. Genome Biol. 2019;20:238. https://doi.org/10.1186/s13059-019-1832-y.

75. Minh BQ, Schmidt HA, Chernomor O, Schrempf D, Woodhams MD, von Haeseler A, et al. IQ-TREE 2: New models and efficient methods for phylogenetic inference in the genomic era. Mol Biol Evol. 2020;37:1530-4. https://doi.org/10.1093/molbev/msaa015.

76. Kumar S, Suleski M, Craig JM, Kasprowicz AE, Sanderford M, Li M, et al. TimeTree 5: An expanded resource for species divergence times. Mol Biol Evol. 2022;39. https://doi.org/10.1093/molbev/msac174.

77. Mendes FK, Vanderpool D, Fulton B and Hahn MW. CAFE 5 models variation in evolutionary rates among gene families. Bioinformatics. 2021;36:5516-8. https://doi.org/10.1093/bioinformatics/btaa1022.

78. Chen D, Zhang T, Chen Y, Ma H and Qi J. Tree2GD: a phylogenomic method to detect large-scale gene duplication events. Bioinformatics. 2022;38:5317-21. https://doi.org/10.1093/bioinformatics/btac669.

79. Lau N, Okubo-Kurihara E, Makita Y, Matsui M. Sample Preparation of Hevea brasiliensis for Proteomic Analysis by LC-MS/MS. protocols.io. 2025. dx.doi.org/10.17504/protocols.io.ewov1mwr7vr2/v1

80. Demichev V, Messner CB, Vernardis SI, Lilley KS and Ralser M. DIA-NN: neural networks and interference correction enable deep proteome coverage in high throughput. Nat Methods. 2020;17:41-4. https://doi.org/10.1038/s41592-019-0638-x.

81. Lau N, Okubo-Kurihara E, Makita Y, Oktavia F, Kuriyama T, Kurihara Y, et al. Supporting data for "Comparative genomics and multi-omics analyses reveal the evolution and physiological basis of rubber biosynthesis in Hevea species" GigaScience Database. 2025. https://doi.org/10.5524/102755

Figure legends

Figure 1: Genome assembly and annotation of *Hevea* species. (A) Summary of genome assembly and annotation statistics across *Hevea* species. (B) Circos plot showing the chromosomal organisation of *Hevea brasiliensis* (outermost ring) and comparative features across *Hevea* species. From outside to inside: proteome abundance in *H. confusa*, *H. collina*, *H. spruceana*, *H. guianensis*, and *H. brasiliensis,* with blue indicating high abundance and red indicating low abundance; rubber biosynthesis-related genes are labelled; orthologous gene relationships between *H. brasiliensis* and *A. thaliana*, *M. esculenta*, *R. communis,* and *J. curcas*; GC content across chromosomes; and the innermost links represent gene duplications within *H. brasiliensis*. (C) Chromosomal distribution of telomeric regions in *H. brasiliensis,* with telomeric regions marked by blue triangles and gene density represented by a heatmap, where red indicates gene-rich regions and blue indicates gene-poor regions.

Figure 2: Pangene analysis of *Hevea*. Simulations of the (A) increase in the pangene size and (B) decrease in the core gene size. (C) Composition of the *Hevea* pangene. Gene ontology

(GO) enrichment of the (D) core, (E) soft-core, (F) shell and (G) cloud genes relative to the pangene. (H) GO annotation of the *H. brasiliensis*-specific genes.

Figure 3: Comparative genomics of *Hevea* species. (A) Maximum likelihood phylogeny inferred from single-copy orthologues. The column on the right represents the number of expanded and contracted gene families. Bar chart shows the distribution of single-copy, multiple-copy and, and unique orthologs in the 11 plant species. Gene ontology enrichment of genes expanded (blue) and contracted (pink) in (B) *Hevea* species and (C) *H. brasiliensis*. The distribution of (D). K_s and (E) 4DTv values of the syntenic gene pairs in the *H. brasiliensis, H. guianensis, H. pauciflora, H. collina,* and *H. confusa* genomes.

Figure 4: Analysis of rubber biosynthesis-related genes. (A) Maximum likelihood tree of CPT amino acid sequences from *Hevea* and other plants. (b) Hi-C plot showing TAD-likes structure for CPT1-4 on chromosome 18. (C) Maximum likelihood tree of CPTL nucleotide sequences from *Hevea* and other plants. (D) Maximum likelihood tree of REF and SRPP amino acid sequences from *Hevea* and other plants. At, *A. thaliana*; Hb, *H. brasiliensis*; Hcf, *H. confusa*; Hcl, *H. collina*; Hg, *H. guianensis*; Hp, *H. pauciflora*; Hs, *H. spruceana*; Ls, *L. sativa*; Pa, *P. argentatum*; Sl, *S. lycopersicum*; and Tb, *T. brevicorniculatum*.

Figure 5: Rubber biosynthesis pathway and proteomic profiles in *Hevea* species. Heatmap showing $\log_2$-transformed average protein abundance across three biological replicates per sample, with colour coding at which yellow indicates high abundance and blue signifies low abundance. MVA pathway: AACT, HMGS, HMGR, MVK, MVA-5-p, MVA-5-pp, PMK, DPMD. MEP pathway: G3P, DXS, DXP, DXS, DXR, MEP, CMS, CME, CMK, PCME, MCS, CMEC, HDS, HMBD, HDR. Formation of isoprenoid precursors: IPP, IPI, DMAPP, GPS, GGPP, FPS, GGPS, GPP. Rubber particle associated proteins: CPT, REF, SRPP, CPTL. Jasmonic acid signalling: COI1, JAZ, MYC. Ethylene signalling: ETR, CTR1,EIN2/3, EIL1. (B) Differentially abundant proteins between *H. brasiliensis* and other *Hevea* species samples.

Bubble plot showing proteins with significantly different abundance levels, where bubble size is proportional to statistical significance value.

Figure 6: Lipidome profiles across *Hevea* species. (A) Bar plot showing the average composition of lipid classes in the *Hevea* latex lipidome across three biological replicates per sample. Hb, *H. brasiliensis*; Hg, *H. guianensis*; Hs, *H. spruceana*; Hcl, *H. collina*; Hcf, *H. confusa*. (B) Orthogonal partial least squares discriminant analysis score plot showing the separation between *H. brasiliensis* and other *Hevea* species samples. (C) Heatmap showing $\log_{10}$-transformed average lipid abundance (based on three biological replicates), with colour coding at which yellow indicates high abundance and blue signifies low abundance. Only differential abundant lipids with VIP > 1 and $p < 0.05$ are shown. (D) Correlation network of proteome and lipidome profiles in *Hevea* latex. Nodes represent proteins (blue) and lipids (orange), and edges represent significant positive correlations (Pearson's $r > 0.75$, $p < 0.01$). The network illustrates the top 50 most significant protein–lipid associations**.**

Click here to access/download;Figure;Fig. 1.tif

A

	H. brasiliensis	*H. guianensis*	*H. pauciflora*	*H. spruceana*	*H. collina*	*H. confusa*
No. of scaffolds	14,722	545,786	558,352	577,834	488,774	574,685
Longest scaffold (Mb)	99.10	65.93	71.52	66.96	59.61	65.79
Scaffold N50 (Mb)	78.35	31.28	49.81	0.29	29.06	38.51
Assembly size (Gb)	1.71	1.63	1.41	1.77	1.42	1.35
GC content (%)	34.48	34.01	34.17	34.33	33.80	34.44
No. of gene models	84,076	44,254	53,110	52,848	73,123	82,122
Mean transcript length (bp)	3,874	3,135	3,358	3,044	3,447	2,987
Mean coding sequence length (bp)	706	677	798	693	599	678
Average no. of exons per transcript	4.4	4.3	4.9	4.6	4.4	4.4
Mean exon length (bp)	160	159	164	152	136	156
Mean intron length (bp)	790	691	591	609	809	637

B

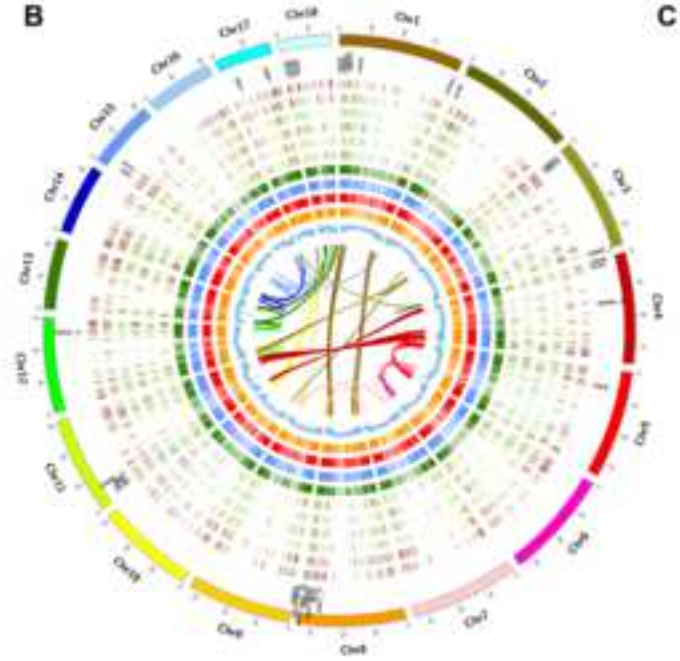

C

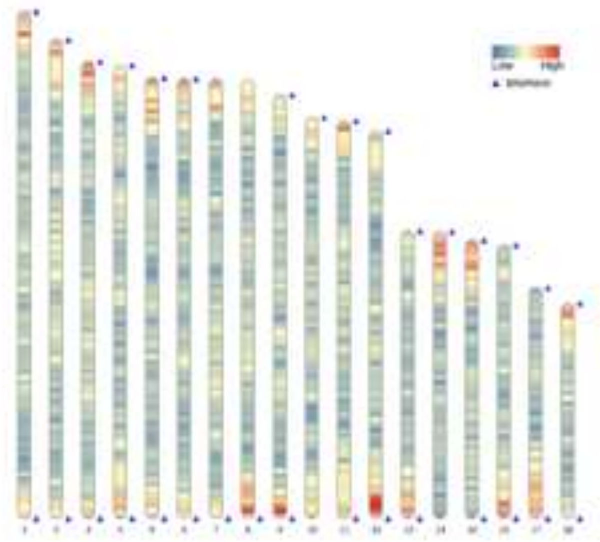

Figure 2 Click here to access/download;Figure;Fig. 2.tif

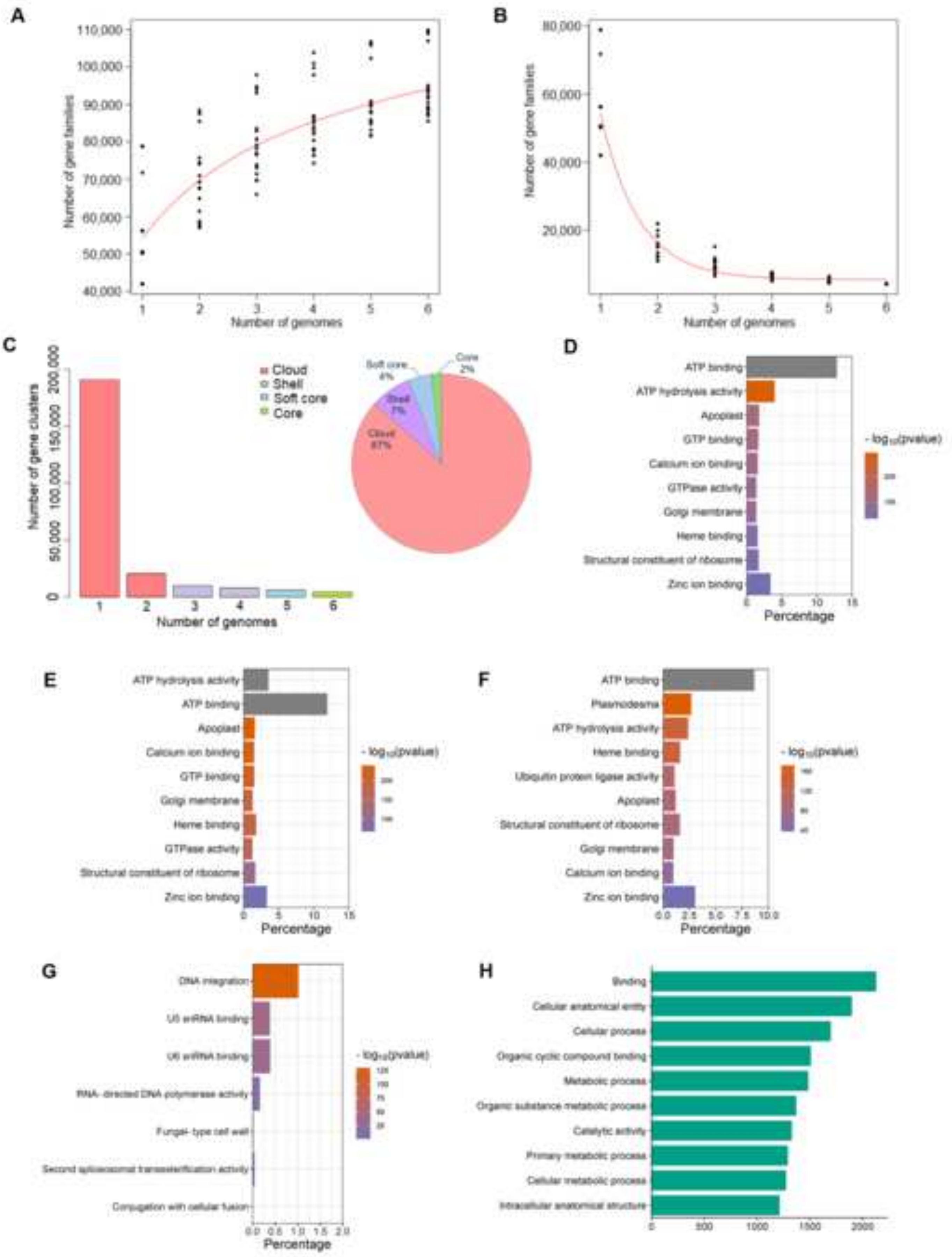

Figure 3 Click here to access/download;Figure;Fig. 3.tif

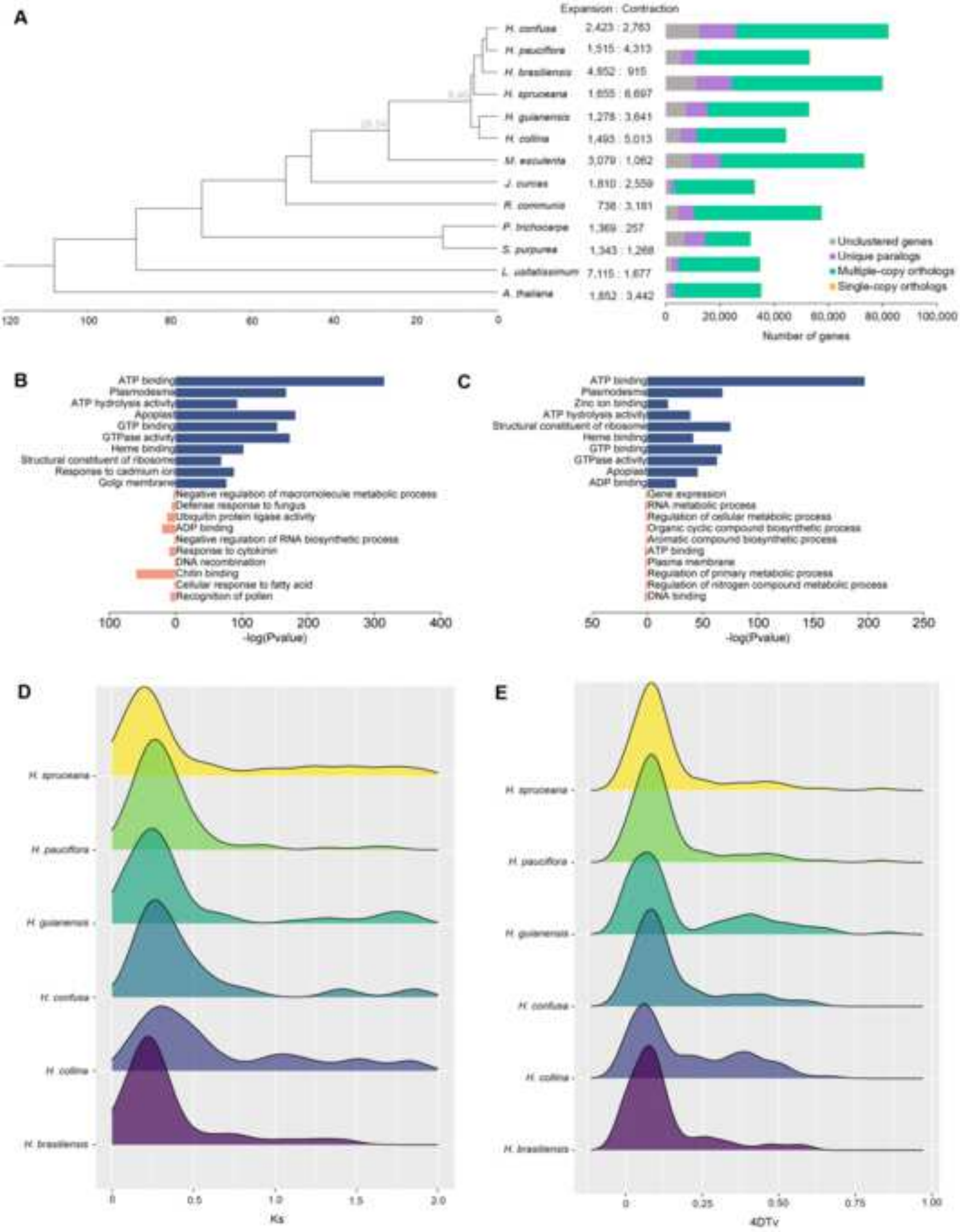

Figure 4

Click here to access/download;Figure;Fig. 4.tif

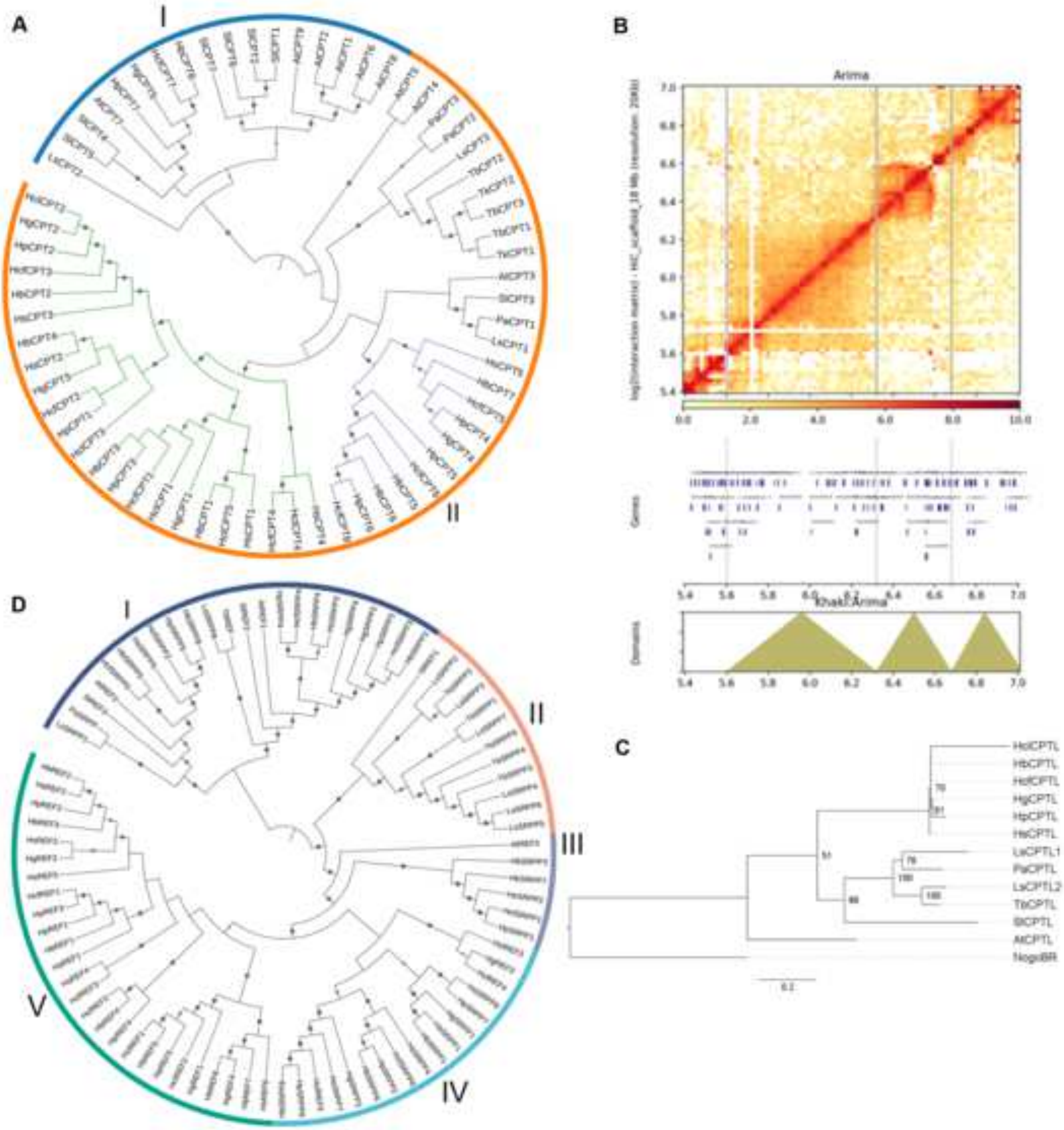

Figure 5 Click here to access/download;Figure;Fig. 5.tif

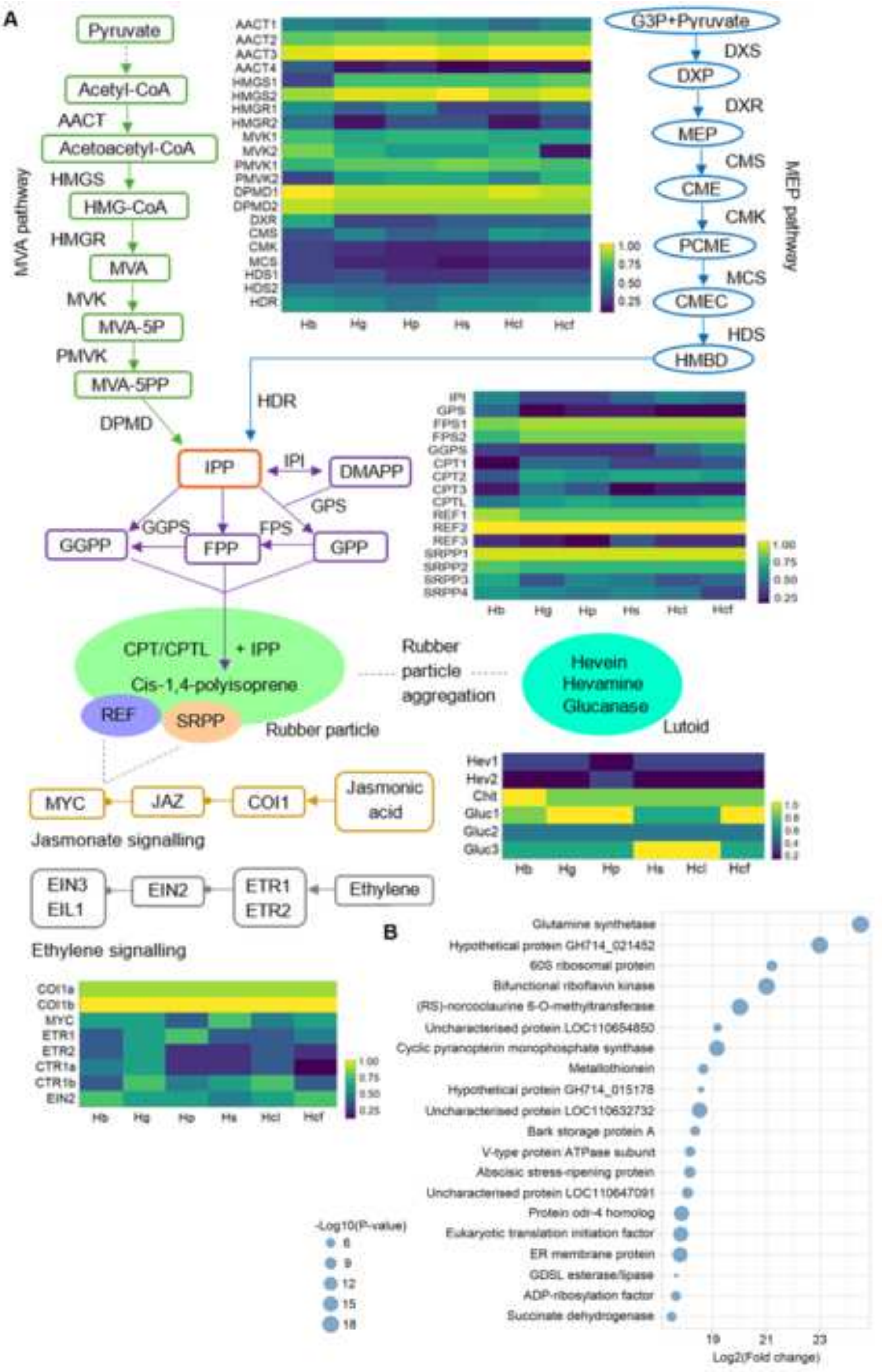

Figure 6

Click here to access/download;Figure;Fig. 6.tif

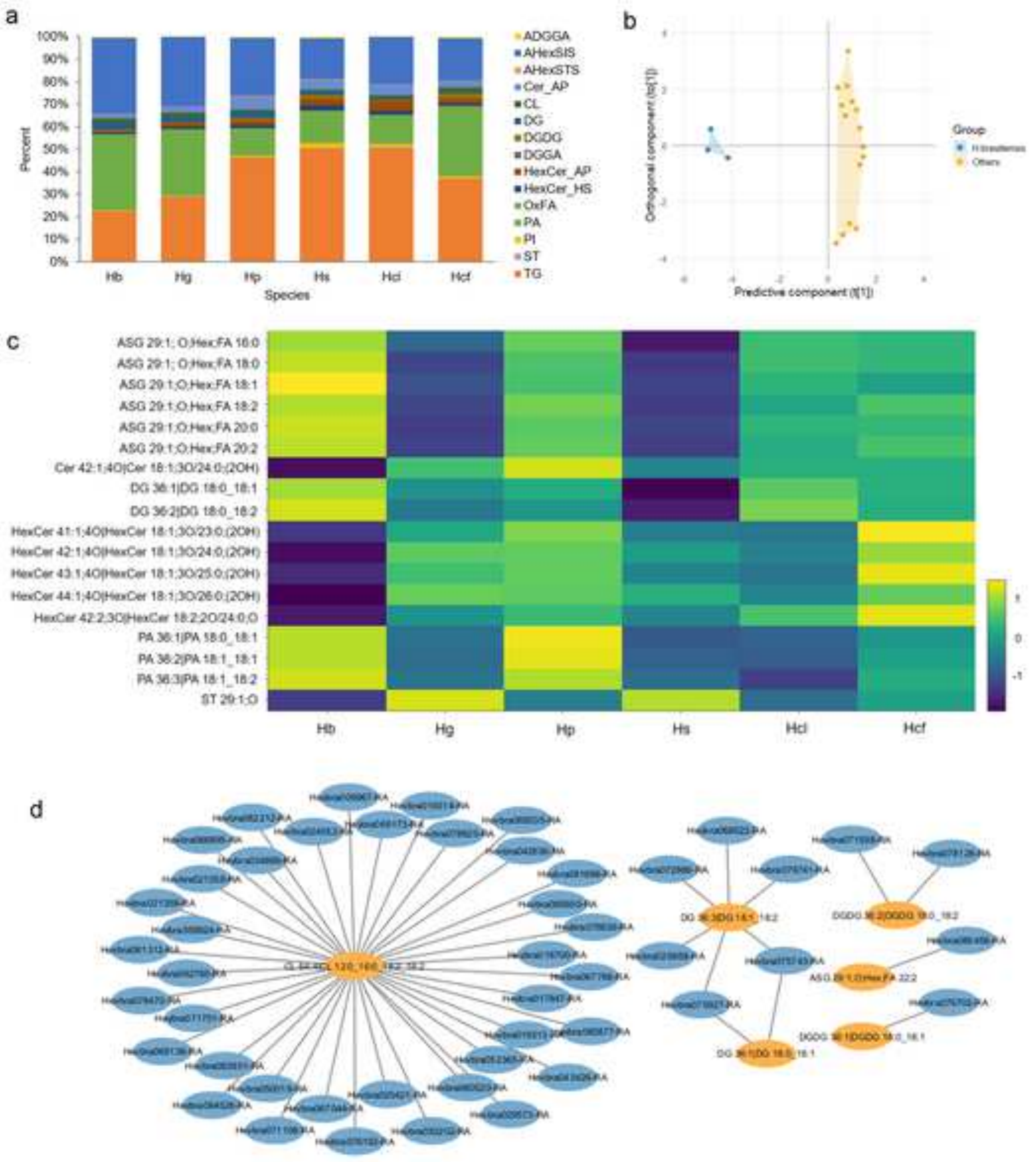

Click here to access/download
Supplementary Material
Supplementary Material_Figures_Comparative genomics Hevea.docx

Supplementary Table

Click here to access/download
Supplementary Material
Supplementary Table_Comparative genomics Hevea.xlsx

Responses to reviewers' comments

Reviewers' comments	Authors responses
Reviewer #1: The manuscript "Comparative genomics and multi-omics analyses reveal the evolution and physiological basis of rubber biosynthesis in Hevea species" is a substantial, timely, and methodologically robust contribution to plant genomics and natural rubber research. Its integration of comparative genomics, proteomics, and lipidomics across six Hevea species provides a comprehensive, multi-layered view of the genetic and metabolic diversity that underpins rubber biosynthesis, with clear implications for breeding and crop improvement. The study's strengths are manifold: the high-quality genome assemblies, including a chromosome-level assembly for H. brasiliensis and de novo assemblies for five additional species, represent a valuable resource for the research community. The pangene analysis, distinguishing core, soft-core, shell, and cloud gene clusters, offers new insights into conserved and variable genetic elements within the genus. Notably, the conservation and expansion of ATP-related functions highlight a previously underappreciated aspect of latex physiology and yield, which reinforces the importance of metabolic regulation for crop productivity.<br><br>Proteomic profiling of latex further distinguishes this work by confirming the presence and abundance of key enzymes involved in rubber biosynthesis—such as CPTs and REF/SRPPs—but also identifying differentially expressed proteins, including glutamine synthetase, metallothionein, and eukaryotic translation initiation factors, which may underpin the superior latex regeneration capacity of H. brasiliensis. This mechanistic link between molecular diversity and agronomic traits is crucial for ongoing and future targeted breeding strategies. The lipidomic analysis, which revealed species-specific	Thank you for your thorough and constructive comments. We are pleased that you found the manuscript to be a valuable and robust contribution to plant genomics and rubber research.<br><br>Most of the remaining *Hevea* species are endemic to Brazil, especially in the Amazon region. Over the past few years, we attempted to obtain samples through a research collaboration agreement with the Brazilian authorities. Despite our efforts, logistical and regulatory challenges prevented the acquisition of genomic DNA or viable tissues from those species. Consequently, we focused on six *Hevea* from the Indonesian national germplasm collection, which provided a representative subset for comparative genomic analysis. While this does not encompass the entire genus, we believe the current dataset offers meaningful insights into *Hevea* evolution and rubber biosynthesis.<br><br>Functional study in another rubber-producing species, *Taraxacum brevicorniculatum* showed that overexpression of ATP-dependent enzymes in the mevalonate pathway enhanced isoprenoid flux.<br>This evidence supports the functional relevance of ATP metabolism-related gene expansion in *Hevea*, and we have incorporated this discussion into the revised manuscript (page 16).<br><br>For differentially abundant proteins, we have also expanded our discussion on the overexpression of glutamine synthetase in *Arabidopsis thaliana* and functional studies on metallothionein to support our proteomics findings (page 18).

lipid compositions that may influence membrane dynamics, rubber particle stability, and ultimately, latex properties, adds a new dimension to our understanding of latex biology. Collectively, these multi-omics approaches advance fundamental knowledge of Hevea evolution and rubber biosynthesis and additionally establish a solid foundation for future functional studies and breeding aimed at improving rubber yield and quality.<br><br>Despite these strengths, there are areas where the manuscript could be improved or expanded. Although the comparative genomic analysis is thorough, the inclusion of only six species out of eleven recognized in the genus limits the generalizability of conclusions regarding genetic diversity and evolutionary relationships. Future studies incorporating additional species, particularly those with distinct ecological adaptations, would strengthen the comparative framework. The functional implications of observed gene family expansions and contractions—especially those related to ATP metabolism—are largely inferred from enrichment analyses and previous literature. Direct functional validation, such as transgenic or gene editing experiments, would help establish causality and further solidify the biological relevance of these findings. Similarly, despite the roles of differentially abundant proteins being discussed in the context of existing studies, experimental validation through knockdown or overexpression in model systems is absent.	
The lipidomic analysis, though comprehensive, is based on field-to-factory samples without chemical preservation, which, although reflective of industrial conditions, complicates direct comparison with previous laboratory-based studies. The manuscript notes discrepancies with earlier reports but could more fully explore the potential impact of	We have revised the Methods section (page 26) to include a more detailed explanation of our sampling approach and how it differs from previous studies that used chemical inhibitors to prevent lipid degradation. We also provide a rationale for our experimental design and explain how these methodological differences may

sampling and processing differences on lipid composition. A more systematic comparison or discussion of methodological variability would enhance the interpretability and reproducibility of these findings. Additionally, the manuscript could further develop or at least hypothesize the ecological and physiological context of observed molecular diversity. For instance, although the divergence between highland and lowland Hevea species is mentioned, the link between specific genomic or metabolic traits and environmental adaptation or stress tolerance is not fully explored. Integrating ecological data or hypothesizing about possible adaptive significance would add depth to the evolutionary narrative.	have influenced the lipidomic profiles observed (page 14).<br><br>To explore potential links between genomic traits and environmental adaptation, we analysed cold stress regulatory genes in *Hevea* species, the ICE–CBF–COR pathway. We observed an expansion of *ICE* and *CBF* genes in the highland *H. guianensis*, which may reflect genomic adaptation to low temperature stress at higher altitude. This analysis has been incorporated into the Discussion (page 17), with further details provided in Supplementary Text S7 and Supplementary Table S33.
The multi-omics approach is a clear strength, yet the integration between datasets could be made more explicit. Although the manuscript discusses proteomic and lipidomic findings in relation to genomic data, a more formal systems biology analysis—such as network modeling or correlation analyses between gene/protein/lipid abundance and phenotypic traits—could yield additional insights and generate testable hypotheses for future work. Furthermore, despite the methods being generally well described, some details could be expanded for full reproducibility. For example, the criteria for differential protein and lipid abundance, the handling of missing data, and the statistical methods for multi-omics integration could be more clearly stated. The manuscript does reference public data repositories and provides accession numbers, which aligns with best practices for data sharing and reproducibility.	We have performed correlation analyses between the proteome and lipidome profiles, and the results have been included in Figure 6D and Supplementary Table S32. The corresponding results have been added to page 15, and the discussion has been included on page 19.<br><br>Details on the parameters used for differential protein abundance analysis and missing data handling have been added to Supplementary Text S6 (page 5), and the details for differential lipid abundance are included on page 27 of the main manuscript.
Regarding adherence to GigaScience's Minimum Standards of Reporting Checklist, the manuscript is largely compliant, with comprehensive methods, clear data availability (except the gene calculation of 239,660 gene families), and appropriate resource descriptions. Yet, explicit details on sample size, replicates,	Orthologous gene analysis using GET_HOMOLOGUES-EST classified the genes into 239,600 families. The resulting gene cluster list is available and will be uploaded to GigaDB. However, the previous login to Gigadb is not accessible, we will complete the data upload once access is provided.

randomization, blinding, statistical assumptions, and protocol deposition would further enhance transparency and reproducibility. The manuscript should specify the exact sample size (n) for each experimental group or condition, clarify whether samples are technical or biological replicates, and state inclusion/exclusion criteria. Details on sample allocation to experimental groups and any randomization procedures should be provided, and if human investigators were involved in sample assessment, a statement on blinding should be included. The manuscript should also clarify how many times each experiment was replicated and indicate the extent of variation between<br>replicates. Statistical methods should be described in greater detail, including whether tests are one- or two-sided, whether adjustments for multiple comparisons were made, and whether means or medians are reported. A justification for the appropriateness of statistical tests and whether data meet test assumptions should be included. The manuscript lists kits and software but could further clarify validation of antibodies or other reagents if used, and consider citing RRIDs for tools and resources to enhance traceability. Despite detailed protocols are referenced in supplementary materials, GigaScience encourages deposition of protocols in open repositories such as protocols.io, with a DOI cited in the Methods. If any code was central to the results, a statement on its availability and licensing should be included. The manuscript is well written and does not require heavy editing for language or clarity, although minor improvements could be made to further clarify<br>statistical reporting and experimental design. One notable change should be considered: To recast the Conclusions statement as a larger-picture, unlike that currently being largely a repetition of results' summary.	Information on the number of biological replicates for the proteomic and lipidomic analyses have been added to the Methods section on page 25-26.<br><br>The description of *p*-value adjustment for multiple testing in the differential protein abundance analysis has been added to Supplementary Text S6. The heatmap of protein abundance in Fig. 5, the bar plot of lipid classes in Fig. 6A, and the lipid abundance heatmap in Fig. 6C were all plotted using the mean values from three biological replicates. These details have been incorporated into the respective figure legends.<br><br>The sample preparation method for proteomic analysis by LC-MS/MS has been deposited on protocols.io, and the DOI is cited in the Methods section (page 25).<br><br>We have revised the conclusion to present a broader perspective, as suggested by the reviewer.<br><br>We have added RRIDs accessions for the tools described in the Methods section.

Reviewer #2: Review of:<br>"Comparative genomics and multi-omics analyses reveal the evolution and physiological basis of rubber biosynthesis in Hevea species"<br><br>I like this paper. Its major strength is its integrative approach, which combines comparative genomics, proteomics, and lipidomics to provide a comprehensive understanding of rubber biosynthesis and latex production across six Hevea species. The study identifies conserved and expanded ATP-related functions, links differentially abundant proteins, such as glutamine synthetase and metallothionein, to latex regeneration, and suggests that species-specific lipid compositions influence the stability of rubber particles.<br><br>The manuscript makes several quite bold claims such as: "The substantial enrichment of proteins involved in nitrogen metabolism, ROS [reactive oxygen species] homeostasis, and protein synthesis suggests that the efficiency of rubber biosynthesis in H. brasiliensis relies on the capacity for latex regeneration" (p17), which argues for a direct link between the enrichment of specific proteins (e.g., glutamine synthetase, metallothionein, and eukaryotic translation initiation factor) and the efficiency of rubber biosynthesis, specifically through enhanced latex regeneration. However, the authors have no functional information to support any of the claims made in the paper. That in itself is not a problem (this is a genomics paper), but the authors should be very tentative about making such claims and frame them as hypotheses to be tested, rather than using words like "likely", "suggests", etc.	Thank you for the positive comments and suggestions.<br><br><br><br><br><br><br><br><br><br><br><br><br><br><br><br><br><br><br>As recommended, we have revised the sentence on page 18 "The substantial enrichment of proteins involved in nitrogen metabolism, ROS (reactive oxygen species) homeostasis, and protein synthesis suggests that the efficiency of rubber biosynthesis in *H. brasiliensis* relies on the capacity for latex regeneration" to frame it more cautiously as a hypothesis to be tested.
Minor issues<br>(1) In the author list, "Yuko Makita Nakai" is listed, but in the "Author Contributions" section (Page 29), she is referred to as "Y.N-M" (likely "Yuko Nakai-Makita"). The hyphenation and order of the name appear inconsistent.	 Thank you for pointing this out. We have corrected the author's name to "Yuko Makita" and updated the "Author Contributions" abbreviation to "Y.M." accordingly.

(2) The abbreviation "ATP" is used heavily throughout. ATP is a well-known term, but for clarity, it should still be spelt out at first use as "adenosine triphosphate"	We have written out ATP in full as *adenosine triphosphate* upon its first mention on page 7.
(3) Figure 5 legend: the phrase "colour coding at which yellow indicates high ..." replace "at" with "in"	As suggested, we have replaced "at" with "in" in the Figure 5 legend.
(4) The abbreviation "LC-MS/MS" is often used; explain it as "liquid chromatography-tandem mass spectrometry (LC-MS/MS)" on first mention.	We have revised LC-MS/MS to its full form, liquid chromatography–tandem mass spectrometry, at its first mention on page 14.
(5) "IPP" is given as "isopentenyl pyrophosphate (IPP)" on Page 3 but as "isopentenyl diphosphate (IPP)" in the Abbreviations section (Page 28).	We have revised "isopentyl pyrophosphate" to "isopentyl diphosphate" on page 3 for consistency.
