# Supplementary material for: Comparative genomics and multiomics analyses reveal the evolution and physiological basis of rubber biosynthesis in Hevea species
Source: Gigascience. 2025 Oct 10;14:giaf115. doi: 10.1093/gigascience/giaf115 (PMC12512020; doi:10.1093/gigascience/giaf115)
Supplement: giaf115_Authors_Response_To_Reviewer_Comments_Original_Submission [file giaf115_authors_response_to_reviewer_comments_original_submission.pdf]

Author's Response To Reviewer Comments

Responses to reviewer 1's comments:
Response 1: Thank you for your thorough and constructive comments. We are pleased that you found the manuscript to be a valuable and robust contribution to plant genomics and rubber research.
Most of the remaining Hevea species are endemic to Brazil, especially in the Amazon region. Over the past few years, we attempted to obtain samples through a research collaboration agreement with the Brazilian authorities. Despite our efforts, logistical and regulatory challenges prevented the acquisition of genomic DNA or viable tissues from those species. Consequently, we focused on six Hevea from the Indonesian national germplasm collection, which provided a representative subset for comparative genomic analysis. While this does not encompass the entire genus, we believe the current dataset offers meaningful insights into Hevea evolution and rubber biosynthesis.
Functional study in another rubber-producing species, Taraxacum brevicorniculatum showed that overexpression of ATP-dependent enzymes in the mevalonate pathway enhanced isoprenoid flux. This evidence supports the functional relevance of ATP metabolism-related gene expansion in Hevea, and we have incorporated this discussion into the revised manuscript (page 16).
For differentially abundant proteins, we have also expanded our discussion on the overexpression of glutamine synthetase in Arabidopsis thaliana and functional studies on metallothionein to support our proteomics findings (page 18).
Response 2: We have revised the Methods section (page 26) to include a more detailed explanation of our sampling approach and how it differs from previous studies that used chemical inhibitors to prevent lipid degradation. We also provide a rationale for our experimental design and explain how these methodological differences may have influenced the lipidomic profiles observed (page 14).
Response 3 To explore potential links between genomic traits and environmental adaptation, we analysed cold stress regulatory genes in Hevea species, the ICE–CBF–COR pathway. We observed an expansion of ICE and CBF genes in the highland H. guianensis, which may reflect genomic adaptation to low temperature stress at higher altitude. This analysis has been incorporated into the Discussion (page 17), with further details provided in Supplementary Text S7 and Supplementary Table S33.
Response 4: We have performed correlation analyses between the proteome and lipidome profiles, and the results have been included in Figure 6D and Supplementary Table S32. The corresponding results have been added to page 15, and the discussion has been included on page 19.
Response 5: Details on the parameters used for differential protein abundance analysis and missing data handling have been added to Supplementary Text S6 (page 5), and the details for differential lipid abundance are included on page 27 of the main manuscript.
Response 6: Orthologous gene analysis using GET_HOMOLOGUES-EST classified the genes into 239,600 families. The resulting gene cluster list is available and will be uploaded to GigaDB. However, the previous login to Gigadb is not accessible, we will complete the data upload once access is provided.
Response 7: Information on the number of biological replicates for the proteomic and lipidomic analyses have been added to the Methods section on page 25-26.
Response 8: The description of p-value adjustment for multiple testing in the differential protein abundance analysis has been added to Supplementary Text S6. The heatmap of protein abundance in Fig. 5, the bar plot of lipid classes in Fig. 6A, and the lipid abundance heatmap in Fig. 6C were all plotted using the mean values from three biological replicates. These details have been incorporated into the respective figure legends.
Response 9: The sample preparation method for proteomic analysis by LC-MS/MS has been deposited on protocols.io, and the DOI is cited in the Methods section (page 25).
Response 10: We have revised the conclusion to present a broader perspective, as suggested by the reviewer.
Response 11: We have added RRIDs accessions for the tools described in the Methods section.
Responses to reviewer 2's comments:
Response 1: Thank you for the positive comments and suggestions.
As recommended, we have revised the sentence on page 18 "The substantial enrichment of proteins involved in nitrogen metabolism, ROS (reactive oxygen species) homeostasis, and protein synthesis suggests that the efficiency of rubber biosynthesis in H. brasiliensis relies on the capacity for latex regeneration" to frame it more cautiously as a hypothesis to be tested.

Response 2: Thank you for pointing this out. We have corrected the author's name to "Yuko Makita" and updated the "Author Contributions" abbreviation to "Y.M." accordingly.
Response 3: We have written out ATP in full as adenosine triphosphate upon its first mention on page 7.
Response 4: As suggested, we have replaced "at" with "in" in the Figure 5 legend.
Response 5: We have revised LC-MS/MS to its full form, liquid chromatography–tandem mass spectrometry, at its first mention on page 14.
Response 6: We have revised "isopentyl pyrophosphate" to "isopentyl diphosphate" on page 3 for consistency.

Editorial Manager®

Cookie Preference Center

We use cookies which are necessary to make our site work. We may also use additional cookies to analyze, improve and personalize our content and your digital experience. For more information, see our Cookie Policy.

You may choose not to allow some types of cookies. However, blocking some types may impact your experience of our site and the services we are able to offer. See the different category headings below to find out more or change your settings.

Allow all

Manage Consent Preferences

Strictly Necessary Cookies

Always active

These cookies are necessary for the website to function and cannot be switched off in our systems. They are usually only set in response to actions made by you which amount to a request for services, such as setting your privacy preferences, logging in or filling in forms. You can set your browser to block or alert you about these cookies, but some parts of the site will not then work.

Functional Cookies

These cookies enable the website to provide enhanced functionality and personalisation. They may be set by us or by third party providers whose services we have added to our pages. If you do not allow these cookies then some or all of these services may not function properly.

Performance Cookies

These cookies allow us to count visits and traffic sources so we can measure and improve the performance of our site. They help us to know which pages are the most and least popular and see how visitors move around the site.

Targeting Cookies

These cookies may be set through our site by our advertising partners. They may be used by those companies to build a profile of your interests and show you relevant adverts on other sites. If you do not allow these cookies, you will experience less targeted advertising.

Cookie List

Clear

Apply Cancel

Consent Leg.Interest

Confirm my choices

Powered by onetrus
