# Supplementary material for: Comparative genomics and multiomics analyses reveal the evolution and physiological basis of rubber biosynthesis in Hevea species
Source: Gigascience. 2025 Oct 10;14:giaf115. doi: 10.1093/gigascience/giaf115 (PMC12512020; doi:10.1093/gigascience/giaf115)
Supplement: giaf115_Reviewer_1_Report_Original_Submission — Marcin Nowicki -- 6/15/2025 [file giaf115_reviewer_1_report_original_submission.pdf]

Reviewer Report

Title: Comparative genomics and multi-omics analyses reveal the evolution and physiological basis of rubber biosynthesis in Hevea species

Version: Original Submission **Date:** 6/15/2025

Reviewer name: Marcin Nowicki

Reviewer Comments to Author:

The manuscript "Comparative genomics and multi-omics analyses reveal the evolution and physiological basis of rubber biosynthesis in Hevea species" is a substantial, timely, and methodologically robust contribution to plant genomics and natural rubber research. Its integration of comparative genomics, proteomics, and lipidomics across six Hevea species provides a comprehensive, multi-layered view of the genetic and metabolic diversity that underpins rubber biosynthesis, with clear implications for breeding and crop improvement. The study's strengths are manifold: the high-quality genome assemblies, including a chromosome-level assembly for H. brasiliensis and de novo assemblies for five additional species, represent a valuable resource for the research community. The pangene analysis, distinguishing core, soft-core, shell, and cloud gene clusters, offers new insights into conserved and variable genetic elements within the genus. Notably, the conservation and expansion of ATP-related functions highlight a previously underappreciated aspect of latex physiology and yield, which reinforces the importance of metabolic regulation for crop productivity.

Proteomic profiling of latex further distinguishes this work by confirming the presence and abundance of key enzymes involved in rubber biosynthesisâ€”such as CPTs and REF/SRPPsâ€”but also identifying differentially expressed proteins, including glutamine synthetase, metallothionein, and eukaryotic translation initiation factors, which may underpin the superior latex regeneration capacity of H. brasiliensis. This mechanistic link between molecular diversity and agronomic traits is crucial for ongoing and future targeted breeding strategies. The lipidomic analysis, which revealed species-specific lipid compositions that may influence membrane dynamics, rubber particle stability, and ultimately, latex properties, adds a new dimension to our understanding of latex biology. Collectively, these multi-omics approaches advance fundamental knowledge of Hevea evolution and rubber biosynthesis and additionally establish a solid foundation for future functional studies and breeding aimed at improving rubber yield and quality.

Despite these strengths, there are areas where the manuscript could be improved or expanded. Although the comparative genomic analysis is thorough, the inclusion of only six species out of eleven recognized in the genus limits the generalizability of conclusions regarding genetic diversity and evolutionary relationships. Future studies incorporating additional species, particularly those with distinct ecological adaptations, would strengthen the comparative framework. The functional implications of observed gene family expansions and contractionsâ€”especially those related to ATP metabolismâ€”are largely inferred from enrichment analyses and previous literature. Direct functional validation, such as transgenic or gene editing experiments, would help establish causality and further solidify the biological relevance of these findings. Similarly, despite the roles of differentially abundant

proteins being discussed in the context of existing studies, experimental validation through knockdown or overexpression in model systems is absent.

The lipidomic analysis, though comprehensive, is based on field-to-factory samples without chemical preservation, which, although reflective of industrial conditions, complicates direct comparison with previous laboratory-based studies. The manuscript notes discrepancies with earlier reports but could more fully explore the potential impact of sampling and processing differences on lipid composition. A more systematic comparison or discussion of methodological variability would enhance the interpretability and reproducibility of these findings. Additionally, the manuscript could further develop or at least hypothesize the ecological and physiological context of observed molecular diversity. For instance, although the divergence between highland and lowland Hevea species is mentioned, the link between specific genomic or metabolic traits and environmental adaptation or stress tolerance is not fully explored. Integrating ecological data or hypothesizing about possible adaptive significance would add depth to the evolutionary narrative.

The multi-omics approach is a clear strength, yet the integration between datasets could be made more explicit. Although the manuscript discusses proteomic and lipidomic findings in relation to genomic data, a more formal systems biology analysisâ€"such as network modeling or correlation analyses between gene/protein/lipid abundance and phenotypic traitsâ€"could yield additional insights and generate testable hypotheses for future work. Furthermore, despite the methods being generally well described, some details could be expanded for full reproducibility. For example, the criteria for differential protein and lipid abundance, the handling of missing data, and the statistical methods for multi-omics integration could be more clearly stated. The manuscript does reference public data repositories and provides accession numbers, which aligns with best practices for data sharing and reproducibility. Regarding adherence to GigaScience's Minimum Standards of Reporting Checklist, the manuscript is largely compliant, with comprehensive methods, clear data availability (except the gene calculation of 239,660 gene families), and appropriate resource descriptions. Yet, explicit details on sample size, replicates, randomization, blinding, statistical assumptions, and protocol deposition would further enhance transparency and reproducibility. The manuscript should specify the exact sample size (n) for each experimental group or condition, clarify whether samples are technical or biological replicates, and state inclusion/exclusion criteria. Details on sample allocation to experimental groups and any randomization procedures should be provided, and if human investigators were involved in sample assessment, a statement on blinding should be included. The manuscript should also clarify how many times each experiment was replicated and indicate the extent of variation between replicates. Statistical methods should be described in greater detail, including whether tests are one- or two-sided, whether adjustments for multiple comparisons were made, and whether means or medians are reported. A justification for the appropriateness of statistical tests and whether data meet test assumptions should be included. The manuscript lists kits and software but could further clarify validation of antibodies or other reagents if used, and consider citing RRIDs for tools and resources to enhance traceability. Despite detailed protocols are referenced in supplementary materials, GigaScience encourages deposition of protocols in open repositories such as protocols.io, with a DOI cited in the Methods. If any code was central to the results, a statement on its availability and licensing should be included. The manuscript is well written and does not require heavy editing for language or clarity, although minor improvements could be made to further clarify statistical reporting and experimental design. One notable change

should be considered: To recast the Conclusions statement as a larger-picture, unlike that currently being largely a repetition of results' summary.

Methods

Are the methods appropriate to the aims of the study, are they well described, and are necessary controls included? Choose an item.

Conclusions

Are the conclusions adequately supported by the data shown? Choose an item.

Reporting Standards

Does the manuscript adhere to the journal's guidelines on minimum standards of reporting? Choose an item.

Choose an item.

Statistics

Are you able to assess all statistics in the manuscript, including the appropriateness of statistical tests used? Choose an item.

Quality of Written English

Please indicate the quality of language in the manuscript: Choose an item.

Declaration of Competing Interests

Please complete a declaration of competing interests, considering the following questions:

- Have you in the past five years received reimbursements, fees, funding, or salary from an organisation that may in any way gain or lose financially from the publication of this manuscript, either now or in the future?
- Do you hold any stocks or shares in an organisation that may in any way gain or lose financially from the publication of this manuscript, either now or in the future?
- Do you hold or are you currently applying for any patents relating to the content of the manuscript?
- Have you received reimbursements, fees, funding, or salary from an organization that holds or has applied for patents relating to the content of the manuscript?
- Do you have any other financial competing interests?
- Do you have any non-financial competing interests in relation to this paper?

If you can answer no to all of the above, write 'I declare that I have no competing interests' below. If your reply is yes to any, please give details below.

I declare that I have no competing interests

I agree to the open peer review policy of the journal. I understand that my name will be included on my report to the authors and, if the manuscript is accepted for publication, my named report including any attachments I upload will be posted on the website along with the authors' responses. I agree for my report to be made available under an Open Access Creative Commons CC-BY license (http://creativecommons.org/licenses/by/4.0/). I understand that any comments which I do not wish to be included in my named report can be included as confidential comments to the editors, which will not be published.

Choose an item.

To further support our reviewers, we have joined with Publons, where you can gain additional credit to further highlight your hard work (see: https://publons.com/journal/530/gigascience). On publication of this paper, your review will be automatically added to Publons, you can then choose whether or not to claim your Publons credit. I understand this statement.

Yes Choose an item.
