# Supplementary material for: Comparative genomics and multiomics analyses reveal the evolution and physiological basis of rubber biosynthesis in Hevea species
Source: Gigascience. 2025 Oct 10;14:giaf115. doi: 10.1093/gigascience/giaf115 (PMC12512020; doi:10.1093/gigascience/giaf115)
Supplement: giaf115_Reviewer_2_Report_Original_Submission — Quentin Cronk -- 6/26/2025 [file giaf115_reviewer_2_report_original_submission.pdf]

Reviewer Report

Title: Comparative genomics and multi-omics analyses reveal the evolution and physiological basis of rubber biosynthesis in Hevea species

Version: Original Submission **Date:** 6/26/2025

Reviewer name: Quentin Cronk

Reviewer Comments to Author:

Review of:
"Comparative genomics and multi-omics analyses reveal the evolution and physiological basis of rubber biosynthesis in Hevea species"
I like this paper. Its major strength is its integrative approach, which combines comparative genomics, proteomics, and lipidomics to provide a comprehensive understanding of rubber biosynthesis and latex production across six Hevea species. The study identifies conserved and expanded ATP-related functions, links differentially abundant proteins, such as glutamine synthetase and metallothionein, to latex regeneration, and suggests that species-specific lipid compositions influence the stability of rubber particles.
The manuscript makes several quite bold claims such as: "The substantial enrichment of proteins involved in nitrogen metabolism, ROS [reactive oxygen species] homeostasis, and protein synthesis suggests that the efficiency of rubber biosynthesis in H. brasiliensis relies on the capacity for latex regeneration" (p17), which argues for a direct link between the enrichment of specific proteins (e.g., glutamine synthetase, metallothionein, and eukaryotic translation initiation factor) and the efficiency of rubber biosynthesis, specifically through enhanced latex regeneration. However, the authors have no functional information to support any of the claims made in the paper. That in itself is not a problem (this is a genomics paper), but the authors should be very tentative about making such claims and frame them as hypotheses to be tested, rather than using words like "likely", "suggests", etc.
Minor issues
(1) In the author list, "Yuko Makita Nakai" is listed, but in the "Author Contributions" section (Page 29), she is referred to as "Y.N-M" (likely "Yuko Nakai-Makita"). The hyphenation and order of the name appear inconsistent.
(2) The abbreviation "ATP" is used heavily throughout. ATP is a well-known term, but for clarity, it should still be spelt out at first use as "adenosine triphosphate"
(3) Figure 5 legend: the phrase "colour coding at which yellow indicates high ..." replace "at" with "in"
(4) The abbreviation "LC-MS/MS" is often used; explain it as "liquid chromatography-tandem mass spectrometry (LC-MS/MS)" on first mention.
(5) "IPP" is given as "isopentenyl pyrophosphate (IPP)" on Page 3 but as "isopentenyl diphosphate (IPP)" in the Abbreviations section (Page 28).

Methods

Are the methods appropriate to the aims of the study, are they well described, and are necessary controls included? Choose an item.

Conclusions

Are the conclusions adequately supported by the data shown? Choose an item.

Reporting Standards

Does the manuscript adhere to the journal's guidelines on minimum standards of reporting? Choose an item.

Choose an item.

Statistics

Are you able to assess all statistics in the manuscript, including the appropriateness of statistical tests used? Choose an item.

Quality of Written English

Please indicate the quality of language in the manuscript: Choose an item.

Declaration of Competing Interests

Please complete a declaration of competing interests, considering the following questions:

- Have you in the past five years received reimbursements, fees, funding, or salary from an organisation that may in any way gain or lose financially from the publication of this manuscript, either now or in the future?
- Do you hold any stocks or shares in an organisation that may in any way gain or lose financially from the publication of this manuscript, either now or in the future?
- Do you hold or are you currently applying for any patents relating to the content of the manuscript?
- Have you received reimbursements, fees, funding, or salary from an organization that holds or has applied for patents relating to the content of the manuscript?
- Do you have any other financial competing interests?
- Do you have any non-financial competing interests in relation to this paper?

If you can answer no to all of the above, write 'I declare that I have no competing interests' below. If your reply is yes to any, please give details below.

'I declare that I have no competing interests'

I agree to the open peer review policy of the journal. I understand that my name will be included on my report to the authors and, if the manuscript is accepted for publication, my named report including any attachments I upload will be posted on the website along with the authors' responses. I agree for my

report to be made available under an Open Access Creative Commons CC-BY license (http://creativecommons.org/licenses/by/4.0/). I understand that any comments which I do not wish to be included in my named report can be included as confidential comments to the editors, which will not be published.

Choose an item.

To further support our reviewers, we have joined with Publons, where you can gain additional credit to further highlight your hard work (see: https://publons.com/journal/530/gigascience). On publication of this paper, your review will be automatically added to Publons, you can then choose whether or not to claim your Publons credit. I understand this statement.

Yes Choose an item.
